# New α- and β-cyclodextrin derivatives with cinchona alkaloids used in asymmetric organocatalytic reactions

**DOI:** 10.3762/bjoc.15.80

**Published:** 2019-04-01

**Authors:** Iveta Chena Tichá, Simona Hybelbauerová, Jindřich Jindřich

**Affiliations:** 1 Department of Organic Chemistry, Faculty of Science, Charles University, Hlavova 8, 128 43, Prague 2, Czech Republic; 2 Department of Teaching and Didactics of Chemistry, Faculty of Science, Charles University, Hlavova 8, 128 43, Prague 2, Czech Republic

**Keywords:** asymmetric allylic amination, cinchona alkaloids, CuAAC click reaction, cyclodextrin, organocatalysts

## Abstract

The preparation of new organocatalysts for asymmetric syntheses has become a key stage of enantioselective catalysis. In particular, the development of new cyclodextrin (CD)-based organocatalysts allowed to perform enantioselective reactions in water and to recycle catalysts. However, only a limited number of organocatalytic moieties and functional groups have been attached to CD scaffolds so far. Cinchona alkaloids are commonly used to catalyze a wide range of enantioselective reactions. Thus, in this study, we report the preparation of new α- and β-CD derivatives monosubstituted with cinchona alkaloids (cinchonine, cinchonidine, quinine and quinidine) on the primary rim through a CuAAC click reaction. Subsequently, permethylated analogs of these cinchona alkaloid–CD derivatives also were synthesized and the catalytic activity of all derivatives was evaluated in several enantioselective reactions, specifically in the asymmetric allylic amination (AAA), which showed a promising enantiomeric excess of up to 75% ee. Furthermore, a new disubstituted α-CD catalyst was prepared as a pure AD regioisomer and also tested in the AAA. Our results indicate that (i) the cinchona alkaloid moiety can be successfully attached to CD scaffolds through a CuAAC reaction, (ii) the permethylated cinchona alkaloid–CD catalysts showed better results than the non-methylated CDs analogues in the AAA reaction, (iii) promising enantiomeric excesses are achieved, and (iv) the disubstituted CD derivatives performed similarly to monosubstituted CDs. Therefore, these new CD derivatives with cinchona alkaloids effectively catalyze asymmetric allylic aminations and have the potential to be successfully applied in other enantioselective reactions.

## Introduction

Cyclodextrins (CDs) [1], cyclic oligosaccharides consisting of α-D-glucopyranoside units, and their derivatives are widely used in many industrial and research areas for their ability to form supramolecular inclusion complexes [2]. CD derivatives have been increasingly applied in catalysis and biomimetic reactions [3–4] thanks to host–guest interactions and to the non-toxic, chiral skeleton of CDs. More specifically, CDs applied in reactions involving metal catalysis [5], organocatalysis [6] and artificial enzymes [7] have been recently studied, thus highlighting their high potential as effective catalysts.

CDs represent an ideal skeleton with a cavity-containing structure for catalysts. Moreover, using native or modified CDs, organic reactions can be performed under green conditions [8–10]. In addition, CDs improve the rate and modulate the regioselectivity and enantioselectivity of reactions [11]. For example, metal-based CD catalytic systems and CD derivatives for organocatalysis have already shown promising results in the studies by Hapiot and Monflier [12], Armspach [13] and others [14–15].

**Figure 1:**
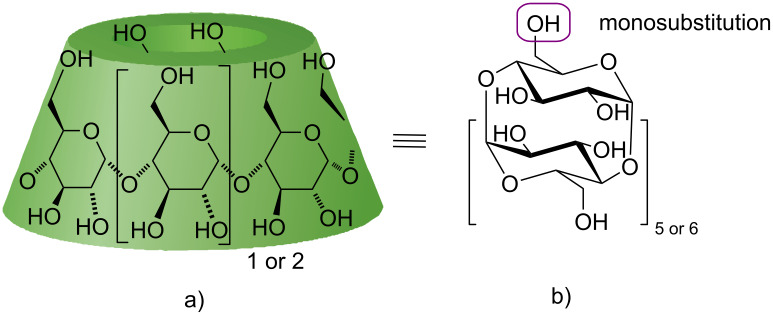
Schematic cone-shaped (a) and structure representations (b) of α-CD (six glucopyranoside units) and β-CD (seven glucopyranoside units).

The chemical modification of native CD skeletons with new functional groups enhances the application of CDs and provides access to new organic chemistry transformations and catalytic systems. Among the approaches used for chemical derivatization of CD skeletons, monosubstitution on the primary rim of CD (Figure 1) is a well-explored strategy [2] which can be used to prepare various types of CD derivatives.

Several examples of modified-CD derivatives with a catalytic nucleophilic center have been reported in the area of organocatalytic asymmetric reactions [11]. Initially, Kanagaraj et al. [16] used per-6-amino-β-CD as the promoter (not in a catalytic amount) of a Henry reaction and obtained the product with 99% ee. Subsequently, Doyagüez et al. [17] attached L-proline to β-CD via different linkers (including a triazole linker) and used the resulting organocatalysts in an aldol reaction in water, albeit with a lower enantiomeric excess (54% ee). Conversely, Shen et al. [18] performed an aldol reaction in a buffer using L- and D-proline-derived CDs connected through a pyrrolidine skeleton as catalysts and observed 94% ee. More recently, Liu et al. [19] reported the excellent enantioselectivity of 99% ee in an aldol reaction catalyzed by β-CD with L-proline attached through a urea moiety. Therefore, mainly proline-derived CDs have been previously tested as organocatalysts and mainly in aldol-type reactions.

**Figure 2:**
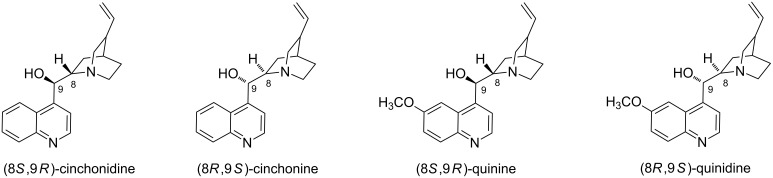
Common cinchona alkaloids (cinchonine, cinchonidine, quinine, quinidine).

The limited number of functional groups attached to CD comprising mainly L-proline restricts the potential of asymmetric organocatalytic reactions using CD derivatives. However, a wide range of catalytic groups, especially cinchona alkaloids (Figure 2), have been used in organocatalysis with excellent results. These naturally occurring compounds and their derivatives are commonly applied in various enantioselective reactions (mainly because of the nucleophilic center on the chiral quinuclidine skeleton) [20]. More importantly, they are a privileged class of chiral catalysts, which are well known for their use in Michael additions [21], Morita–Baylis–Hillman reactions [22], and aldol reactions [23], among others [24]. Hence, combining cinchona alkaloids with CDs has the potential to widen the applications of CD derivatives in asymmetric organocatalysis.

The combination of cinchona alkaloids with CDs was first reported by Liu et al. [25] who prepared inclusion complexes of cinchona alkaloids and organoselenium-bridged bis-β-CDs. Subsequently, the same research group [26] investigated the performance of inclusion complexes of native and permethylated β-CDs and cinchona alkaloids as pH-responsive binding systems. Nevertheless, to the best of our knowledge, CD derivatives with covalently bonded cinchona alkaloids have never been prepared and tested in asymmetric organocatalysis. Thus, in this study, we investigated methods for attaching cinchona alkaloids to CD skeletons, and we assessed the enantiomeric excess of the resulting CD derivatives as organocatalysts in asymmetric reactions, specifically in the asymmetric allylic amination (AAA).

We successfully prepared a series of monosubstituted α- and β-CDs derivatives with the cinchona alkaloids cinchonine, cinchonidine, quinine, and quinidine with up to 95% isolated yield through CuAAC click reactions. By this simple, high-yielding and quick method we synthesized eight new CD derivatives, four based on the α-CD and four based on β-CD skeleton. In addition, to widen the usability and to improve the solubility of the prepared CD derivatives, the corresponding eight permethylated analogs were also synthesized. Furthermore, to test more advanced types of catalysts, a disubstituted α-CD derivative as a pure AD regioisomer with two identical cinchona alkaloid residues was prepared and tested in the AAA reaction.

Our study shows that the CuAAC reaction is a good and high-yielding method for the functionalization of the CD skeleton when attaching sterically demanding cinchona groups. Additionally, some of these new CD derivatives showed promising results of up to 75% ee in the AAA reactions of Morita–Baylis–Hillman (MBH) carbamates and significant differences depending on the attached cinchona alkaloid (cinchonine, cinchonidine, quinine, quinidine) as well as on the size of the cavity, i.e., β-CD or α-CD derivatives). Thus, this study showed that cinchona-substituted CD catalysts are active in organocatalytic reactions.

## Results and Discussion

### Synthesis of monosubstituted non-methylated CD derivatives

Initially, the method for attaching cinchona alkaloids to non-methylated CDs was developed. For our purposes of using the derivatives as catalysts for enantioselective reactions, we focused on α- and β-CD skeletons.

**Scheme 1:**
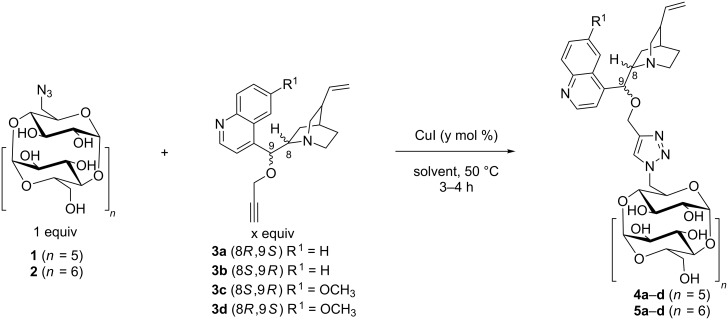
CuAAC click reaction of propargylated cinchona alkaloids **3a–d** with 6^I^-azido-6^I^-deoxy-α-CD (**1**) and 6^I^-azido-6^I^-deoxy-β-CD (**2**).

Our successful approach consisted of attaching these molecules through copper-catalyzed alkyne–azide cycloaddition (CuAAC). First, the required starting materials 6^I^-azido-6^I^-deoxy-α-CD (**1**) [27] and 6^I^-azido-6^I^-deoxy-β-CD (**2**) [27] and 9-*O*-propargylated cinchona alkaloid derivatives **3a–d** [28] were synthesized followed by optimization of the conditions for the CuAAC click reaction (Scheme 1).

The CuAAC click reaction conditions were initially optimized using α-CD (Table 1). Initially, the reaction was performed in a THF/H_2_O mixture with 1.5 equiv of 9-*O*-propargylated cinchonine (**3a**) and 50 mol % CuI affording the product in 78% yield (Table 1, entry 1). Reducing both the amount of propargylated cinchona alkaloid **3a** to 1.3 equiv and the amount of the Cu salt to 20 mol % resulted in virtually the same yield of the product (Table 1, entry 2). Conversely, the further decreasing the amount of propargylated cinchonine (**3a**) to 1.05 equiv led to a significantly lower conversion to the product (56%, Table 1, entry 3). Thus, based on the optimal conditions identified for the α-CD skeleton (Table 1, entry 2), the subsequent syntheses were performed using 1.3 equiv of cinchona alkaloids **3b–d** in a mixture of THF/H_2_O with 20 mol % CuI. The corresponding α-CD products (**4a–d**, **5a–d**) were isolated in high yields of up to 86% yield (Table 1, entries 2 and 4**–**6).

**Table 1:** Optimized conditions of the CuAAC click reactions of non-methylated azido-CDs with propargylated cinchona alkaloids depicted in Scheme 1.

Entry	CD	Alkaloid	R^1^	x (equiv)	y (mol %)	Solvent	Yield^a^ in % (product)

1	**1**	**3a** (8*R*,9*S*)	H	1.5	50	THF/H_2_O 1:1	78 (**4a**)
2	**1**	**3a** (8*R*,9*S*)	H	1.3	20	THF/H_2_O 1:1	77 (**4a**)
3	**1**	**3a** (8*R*,9*S*)	H	1.05	20	THF/H_2_O 1:1	56 (**4a**)
4	**1**	**3b** (8*S*,9*R*)	H	1.3	20	THF/H_2_O 1:1	86 (**4b**)
5	**1**	**3c** (8*S*,9*R*)	OCH_3_	1.3	20	THF/H_2_O 1:1	72 (**4c**)
6	**1**	**3d** (8*R*,9*S*)	OCH_3_	1.3	20	THF/H_2_O 1:1	74 (**4d**)
7^b^	**2**	**3a** (8*R*,9*S*)	H	1.3	20	THF/H_2_O	20 (**5a**)
8	**2**	**3a** (8*R*,9*S*)	H	1.3	20	DMF	89 (**5a**)
9	**2**	**3b** (8*S*,9*R*)	H	1.3	20	DMF	70 (**5b**)
10	**2**	**3c** (8*S*,9*R*)	OCH_3_	1.3	20	DMF	80 (**5c**)
11	**2**	**3d** (8*R*,9*S*)	OCH_3_	1.3	20	DMF	95 (**5d**)
12^b^	**1**	**3a** (8*R*,9*S*)	H	1.3	20	DMF	38 (**4a**)

^a^Isolated yield. ^b^After 48 hours.

In the reactions with β-CD (**2**, Table 1, entries 7–11), no full conversion into the product could be achieved in the solvent mixture THF/H_2_O even after 48 hours of reaction (Table 1, entry 7). However, when changing the solvent to DMF a full conversion into the product was observed within 2 hours of reaction affording the products with high to excellent yields (95%, **5a–d**, Table 1, entries 8**–**11). Conversely, the product yield was low when using DMF for a CuAAC reaction with α-CD resulting in only 38% of product **4a** after 48 hours (Table 1, entry 12).

### Synthesis of monosubstituted methylated CD derivatives

**Scheme 2:**
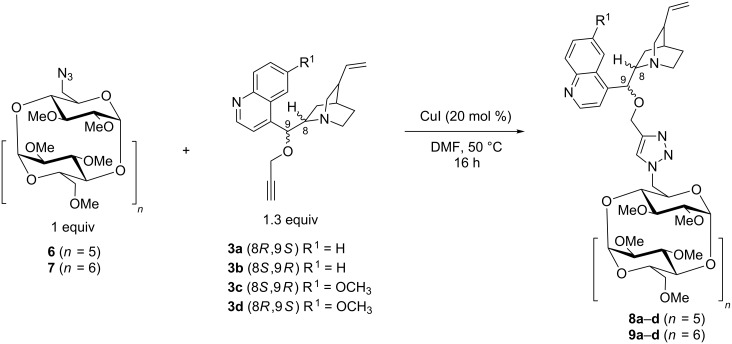
CuAAC click reaction of per-Me-N_3_-α-CD (**6**) or per-Me-N_3_-β-CD (**7**) and propargylated cinchona alkaloids **3a–d**.

After developing the approach for attaching of cinchona alkaloids to non-methylated CD skeletons, we next focused on the functionalization of permethylated CD derivatives. First, we prepared the starting CD compounds, per-Me-N_3_-α-CD (**6**) [29] and per-Me-N_3_-β-CD (**7**) [30], and subjected them to the previously optimized conditions of the CuAAC click reaction with propargylated cinchona alkaloids (**3a–d**). The resulting permethylated CD derivatives **8a–d**, **9a–d** were isolated in high yields of up to 69% (Scheme 2).

As outlined in Table 2, the conditions assessed using the non-methylated CDs were applied to prepare per-Me-α-CD (**6**) analogs. Thus, reaction **6** with 1.3 molar equivalents of the propargylated cinchona alkaloid **3a** in the presence of 20 mol % CuI in DMF afforded product **8a** in 59% yield (Table 2, entry 1). Subsequently, we prepared the other permethylated cinchona–α-CD derivatives **8b–d** with moderate yields (42–49% yield, Table 2, entries 2**–**4). In the case of per-Me-β-CD **7**, following the same procedure, the products **9a–d** were also isolated with good to high yields (up to 69% yield, Table 2, entries 6**–**9). Concomitantly, the reaction was investigated in the THF/H_2_O solvent mixture (Table 2, entry 5), however, the reaction in DMF afforded a higher isolated yield (Table 2, entry 3).

**Table 2:** Yields for optimized conditions of CuAAC click reaction of permethylated azido-CDs with propargylated cinchona alkaloids from Scheme 2.

Entry	CD	Alkaloid	R^1^	Yield^a^ in % (product)

1	**6**	**3a** (8*R*,9*S*)	H	59 (**8a**)
2	**6**	**3b** (8*S*,9*R*)	H	48 (**8b**)
3	**6**	**3c** (8*S*,9*R*)	OCH_3_	49 (**8c**)
4	**6**	**3d** (8*R*,9*S*)	OCH_3_	42 (**8d**)
5^b^	**6**	**3c** (8*S*,9*R*)	OCH_3_	34 (**8c**)
6	**7**	**3a** (8*R*,9*S*)	H	64 (**9a**)
7	**7**	**3b** (8*S*,9*R*)	H	69 (**9b**)
8	**7**	**3c** (8*S*,9*R*)	OCH_3_	48 (**9c**)
9	**7**	**3d** (8*R*,9*S*)	OCH_3_	63 (**9d**)

^a^Isolated yield. ^b^THF/H_2_O solvent mixture.

### Synthesis of disubstituted CD derivatives

To open the way for the preparation of more versatile types of enantioselective organocatalysts containing a CD skeleton and cinchona alkaloids, a method for the synthesis of a disubstituted derivative of cinchona alkaloid–non-methylated CD was developed. The prepared derivative was subsequently tested in an AAA reaction. In contrast to the monosubstituted derivatives, disubstituted CDs should be considered as possible mixtures of regioisomers and pseudoenantiomers [31–32]. Because of the results published by our group previously [33], we chose an AD regioisomer (as a pure regioisomer) on an α-CD skeleton for the preparation of the catalyst.

**Scheme 3:**
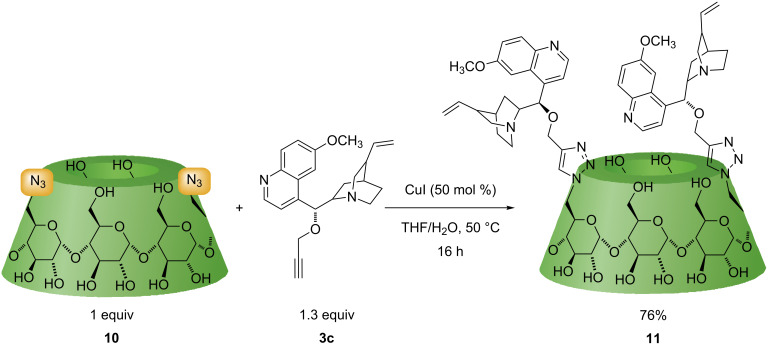
Synthesis of difunctionalized α-CD **11** with quinine moieties.

Initially, we synthesized the starting material, 6^A^,6^D^-diazido-6^A^,6^D^-dideoxy-α-CD (**10**) [34–36] and reacted it with propargylated cinchona alkaloid **3c**. The disubstituted product **11** with a quinine moiety (**3c**) at position 1,4 on the primary rim of the α-CD skeleton was isolated in 76% yield (Scheme 3).

### NMR elucidation of the prepared cinchona–CD derivatives

**Figure 3:**
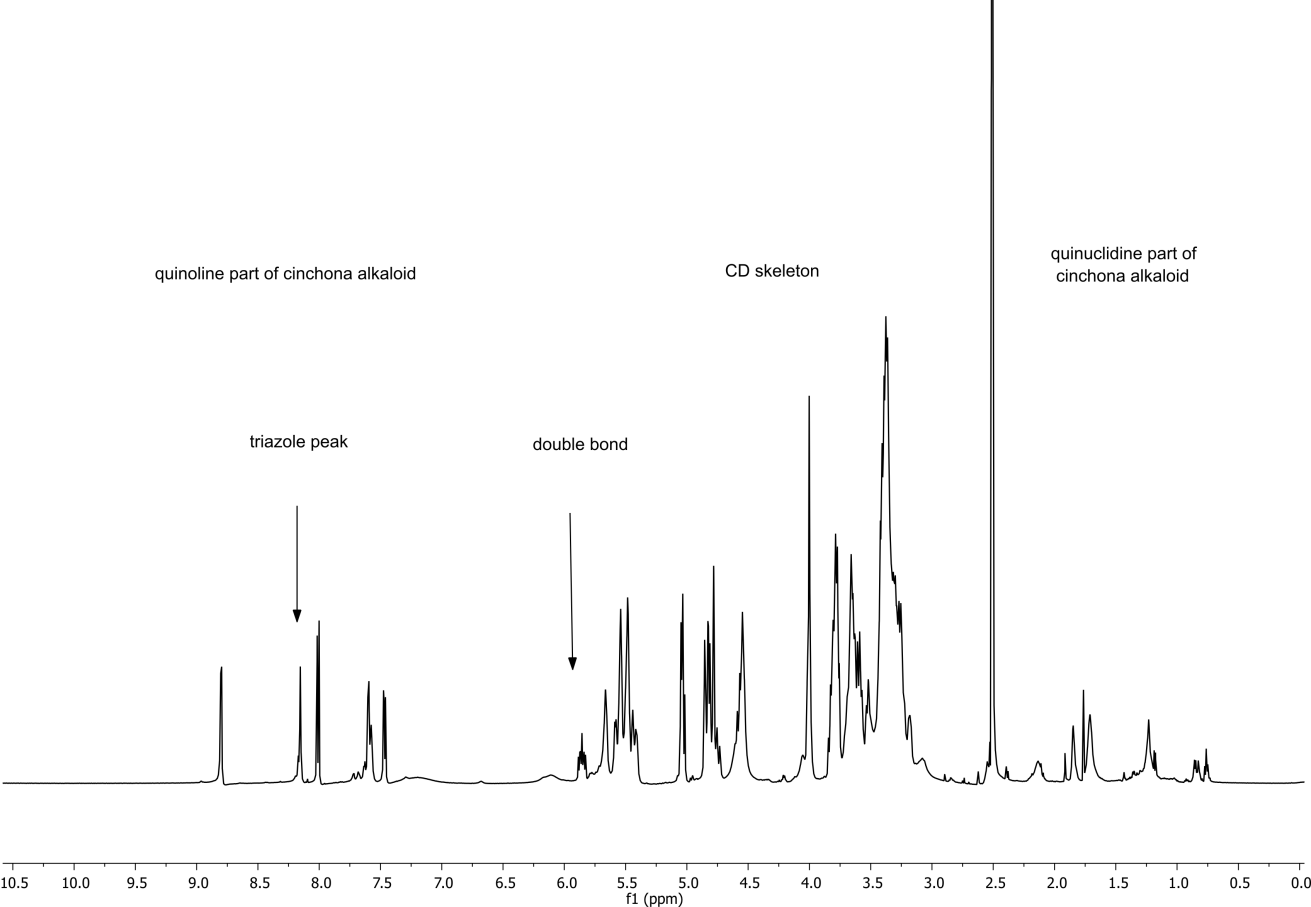
Representative ^1^H NMR spectrum of the non-methylated quinidine–α-CD derivative **4d**.

The structures of mono- (**4a–d**, **5a–d**) and disubstituted (**11**) non-methylated CDs and permethylated (**8a–d**, **9a–d**) CD derivatives were unambiguously confirmed by NMR measurements. As representative example of the prepared CD derivatives, we chose the α-CD derivative substituted with quinidine **4d**. The ^1^H NMR spectra of the non-methylated CD derivatives in DMSO-*d*_6_ are in accordance with monosubstituted derivatives at position 6 on the primary rim (Figure 3). Generally, we observed four different regions with the typical signals: the first, well-resolved aromatic region belongs to the quinoline part of the cinchona alkaloid (9.00**–**7.55 ppm) and to the hydrogen signal of the triazole (8.21 ppm), thus confirming the successful attachment of the cinchona alkaloid to the CD skeleton through the CuAAC click reaction. The second part of the ^1^H NMR spectrum comprises the resolved signal for the double bond on the quinuclidine skeleton of the cinchona alkaloid (5.93 ppm). The third part of the spectrum consists of the CD region (5.50**–**3.20 ppm) with H-1 atoms of unsubstituted glucose units (4.80 ppm) and H-1^I^ (5.03 ppm) for the substituted glucose. The signals of the H-2, H-3, H-4 and H-6 atoms of unsubstituted units are observed at around 4.00**–**3.00 ppm; on the other hand, the signal for H-6^I^ is separately visible around 4.75 ppm (especially in the HSQC and ^1^H,^1^H COSY spectra). This part of the spectrum also includes the primary rim OH groups (4.49**–**4.34 ppm) and secondary rim OH groups (5.91**–**5.53 ppm). Finally, the quinuclidine skeleton part of the cinchona alkaloid is identified in the region around 2.00**–**1.20 ppm.

**Figure 4:**
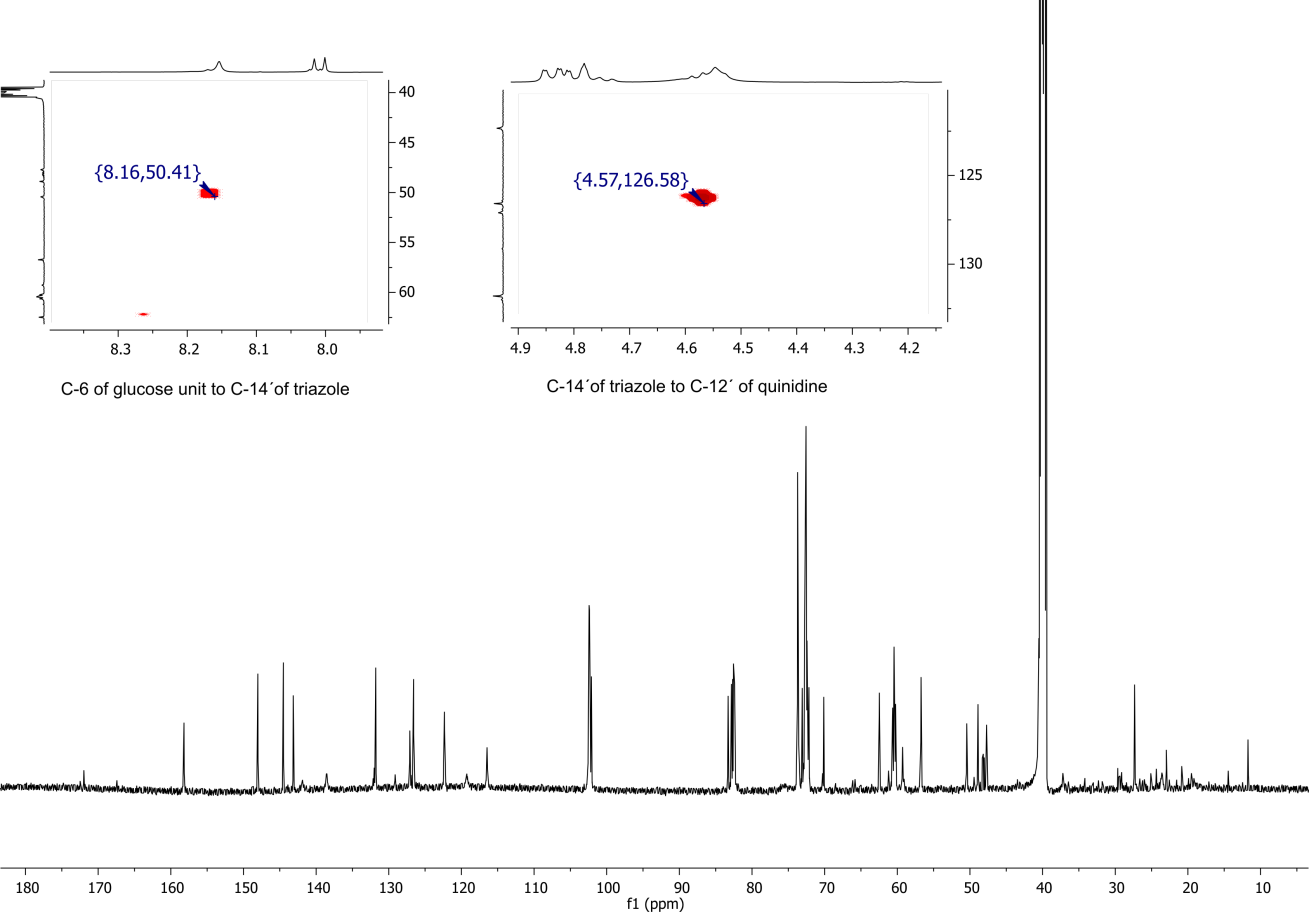
Representative ^13^C NMR spectrum and parts of the HMBC spectrum of the non-methylated quinidine–α-CD derivative **4d**.

Subsequently, ^13^C NMR, DEPT-edited HSQC and HMBC spectra also confirmed the substitution on the primary rim of the CD skeleton (Figure 4). The C-6 atom of the substituted glucose unit is correlated with the hydrogen signal of the triazole ring (50.41 ppm of C-6^I^ to 8.16 ppm of H-14' of triazole) and 126.13 ppm of C-14' triazole is correlated to the signal at 4.57 ppm of the quinidine part. Additional 2D NMR spectra (COSY, HSQC, HMBC, ROESY) are included in Supporting Information File S2 and Supporting Information File S3.

Moreover, we also investigated a possible inclusion of the cinchona alkaloid substituent in the CD cavity in the case of cinchonine–β-CD **5a** in D_2_O. The 2D ROESY spectrum showed cross-peaks between the substituent (hydrogen atoms of the double bond of the quinuclidine skeleton) and the inner H-3 atoms of the β-CD cavity. However, the low solubility of non-methylated β-CDs in H_2_O (1 mg/1 mL) did not allow us to further investigate the nature of the inclusion, e.g., by concentration dependency measurements. Thus, the observed cross-peaks could be caused by intermolecular interactions (inclusion of the part of the cinchonine moiety into the second CD cavity) or by intramolecular interactions. Nevertheless, the rotation of the substituted glucopyranoside unit as discussed by Hapiot and Monflier [37], leading to the formation of the *in* isomer, is not very probable in our case, due to the large steric demand of the cinchona substituent. Moreover, the CD inner hydrogens showed no cross-peaks with the triazole ring hydrogen as well as with no cinchona hydrogens which are close to the triazole. The results of these NMR measurements in D_2_O are collected in Supporting Information File 1.

In conclusion, we unambiguously confirmed the cinchona alkaloid attachment to the CD skeleton through the triazole by 2D NMR measurements. This thorough investigation revealed no triple bond and a new triazole hydrogen signal while correlating carbon C6 of the substituted glucose unit with the triazole. Therefore, the prepared CD derivatives are substituted on the primary side. Further characterization data are included in Supporting Information Files 1–3.

### Catalytic activity of cinchona–CD derivatives

**Scheme 4:**
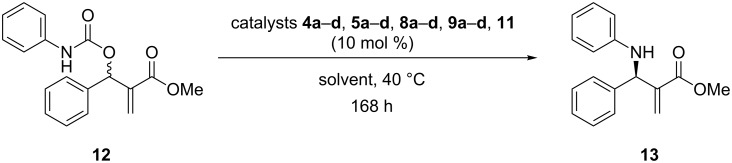
AAA reaction of MBH carbamate **12** catalyzed by the prepared CD derivatives **4a–d**, **5a–d**, **8a–d**, **9a–d**, **11**.

Lastly, the activity of all prepared CD derivatives was tested in asymmetric organocatalytic reactions. After unsuccessful application in Morita–Baylis–Hillman and aldol-type reactions, we focused on their application in the decarboxylative asymmetric allylic amination (AAA) [38] of MBH carbamate **12** affording the product **13** with enantiomeric excesses of up to 75% (Scheme 4).

However, compared with the published procedure [38] (up to 97% ee, aromatic solvent, 40 °C, and 168 hours), the solvent of the reaction had to be changed in the case of non-methylated CDs due to their lower solubility in organic solvents. The reaction conditions and results are summed up in Table 3.

**Table 3:** Catalytic activity of the CD derivatives in the AAA reaction of MBH carbamate **12**.^a^

Entry	Catalyst	Solvent	Yield^b^ (%)	ee (%)

1	DABCO	toluene	89	–
2^c^	per-Me-α-CD	toluene	n.d.	–
3^c^	per-Me-β-CD	toluene	n.d.	–
4	**8a**	toluene	62	13
5	**8b**	toluene	42	74
6	**8c**	toluene	76	25
7	**8d**	toluene	47	27
8	**9a**	toluene	37	15
9	**9b**	toluene	55	69
10	**9c**	toluene	12	15
11	**9d**	toluene	44	25
12	**9b**	CHCl_3_	63	69
13	**9b**	MTBE	63	69
14	**9b**	MeOH	73	33
15^d^	**9b**	toluene	15	75
16^c^	α-CD	ACN/H_2_O	n.d.	–
17^c^	β-CD	ACN/H_2_O	n.d.	–
18	**4a**	ACN/H_2_O	10	3
19^c^	**4b**	DMF	n.d.	–
20	**4b**	ACN/H_2_O	5	0
21	**4c**	ACN/H_2_O	12	0
22	**4d**	ACN/H_2_O	18	21
23	**5a**	ACN/H_2_O	26	5
24	**5b**	DMF	9	25
25	**5b**	DMSO	19	23
26	**5b**	ACN/H_2_O	19	19
27	**5c**	ACN/H_2_O	5	0
28	**5d**	ACN/H_2_O	21	13
29^c^	**11**	ACN/H_2_O	n.d.	–
30^c,e^	**11**	ACN/H_2_O	n.d.	–

^a^Standard conditions: 10 mol % catalyst, 0.4 M solution, solvent, 40 °C, 168 hours. ^b^Isolated yield. ^c^n.d. = not detected, – = not measured. ^d^Temperature 25 °C. ^e^With 5 mol % (1*S*)-CSA.

First, the racemic version of this reaction with DABCO gave 89% yield of the product (Table 3, entry 1). Pure permethylated α- and β-CDs without cinchona alkaloid modification were also tested (Table 3, entries 2 and 3) as blank catalysts but completely failed. Promising results were achieved with permethylated CD–cinchonidine derivatives **8b** and **9b** affording the product in 74 and 69% ee, respectively (Table 3, entries 5 and 9). Other permethylated CD derivatives **8a**, **8c**, **8d**, **9a**, **9c**, **9d** resulted in low ee (Table 3, entries 4, 6**–**8, 10 and 11). Based on these results, the promising catalyst **9b** was selected and investigated under different conditions (solvents and temperature, Table 3, entries 12–15) with similar results. Lastly, native α- and β-CDs were also tested as blank catalysts to confirm that the CD molecule without any modification has no influence on the reaction (Table 3, entries 16 and 17). Furthermore, the non-methylated monosubstituted CD derivatives **4a–d**, **5a–d** afforded on one side lower enantiomer excesses (Table 3, entries 18**–**28), on the other hand, they showed some catalytic activity in the solvent mixture acetonitrile/H_2_O, which could be promising for future applications of these catalysts in water. The disubstituted CD derivative **11** was not active in this enantioselective reaction (Table 3, entry 29) and this derivative was also tested in the AAA reaction with (1*S*)-10-camphorsulfonic acid (CSA) according to the original procedure [38], in which the cocatalyst (1*S*)-CSA enhanced the efficiency of dimeric cinchona alkaloids (Table 3, entry 30). However, there was no difference observable under these conditions.

## Conclusion

We prepared a series of new 6-monosubstituted α-CD and β-CD derivatives modified with four different cinchona alkaloids, i.e., cinchonine, cinchonidine, quinine, and quinidine. The products were obtained in high yields through the CuAAC reaction and subsequently applied as catalysts in enantioselective reactions. We fully characterized the series of new 16 cinchona–CD derivatives including non-methylated and permethylated CDs by 2D NMR, MS, IR spectroscopy and we optimized their preparation (less than 3 h and up to 95% isolated yield). We applied them in the decarboxylative asymmetric allylic amination of a Morita–Baylis–Hillman carbamate (10 mol % of catalyst, up to 75% ee, up to 76% isolated yield). We believe that these new CD derivatives comprising cinchona alkaloids will be suitable catalysts of other asymmetric reactions using them under green chemistry conditions.

## Supporting Information

File 1 Experimental procedures, characterization data, copies of NMR spectra and chiral HPLC analysis.

File 2 2D NMR spectra of compounds **4a**–**d** and **5a**–**d**.

File 3 2D NMR spectra of compounds **8a**–**d**, **9a**–**d** and **11**.
