# Supplementary material for: New α- and β-cyclodextrin derivatives with cinchona alkaloids used in asymmetric organocatalytic reactions
Source: Beilstein J Org Chem. 2019 Apr 1;15:830–9. doi: 10.3762/bjoc.15.80 (PMC6466772; doi:10.3762/bjoc.15.80)
Supplement: File 1 — Experimental procedures, characterization data, copies of NMR spectra and chiral HPLC analysis. [file Beilstein_J_Org_Chem-15-830-s001.pdf]

BEILSTEIN JOURNAL OF ORGANIC CHEMISTRY

Supporting Information

for

New α- and β-cyclodextrin derivatives with cinchona alkaloids used in asymmetric organocatalytic reactions

Iveta Chena Tichá, Simona Hybelbauerová and Jindřich Jindřich

Beilstein J. Org. Chem. **2019,** *15,* 830–839. doi:10.3762/bjoc.15.80

Experimental procedures, characterization data, copies of NMR spectra and chiral HPLC analysis

License and Terms: This is a supporting information file under the terms of the Creative Commons Attribution License (http://creativecommons.org/licenses/by/4.0). Please note that the reuse, redistribution and reproduction in particular requires that the authors and source are credited.
The license is subject to the *Beilstein Journal of Organic Chemistry* terms and conditions: (https://www.beilstein-journals.org/bjoc)

Table of contents

Instruments and materials S2

General information for NMR elucidation of CD catalysts S3

General procedure for the preparation of monosubstituted non-methylated CD derivatives (GP1) S4

General procedure for the preparation of monosubstituted permethylated CD derivatives (GP2) S11

Preparation of disubstituted α-CD derivatives S18

Catalytic activity of CD derivatives S19

References S20

^{1}H and ^{13}C NMR spectra of prepared CD derivatives S21

HRMS spectra of prepared CD compounds S38

Chiral HPLC chromatograms S44

Instruments and materials

α and β-CD were purchased from Wacker Chemie, Germany. (−)-Cinchonidine (96%), cinchonine (98%), quinine (90%), quinidine (98%), propargyl bromide (80% in toluene), copper(I) iodide, and *p*-toluenesulfonyl chloride were purchased from Sigma-Aldrich. Sodium azide, *N*,*N*-dimethylformamide, tetrahydrofuran, acetonitrile (ACN), and pyridine were purchased from Penta Chemicals, Czech Republic.

Silica gel 60 (0.040–0.063 mm) was used for column chromatography and TLC was performed on aluminum sheets with a layer of Silica gel 60 F_{254}, both purchased from Merck, Germany. Ratio of solvents in elution mixtures is given as volume/volume. Argon was used as an inert gas. Spots on TLC plates were detected by using an UV lamp (λ = 254 nm for cinchonine and cinchonidine derivatives and λ = 365 nm for quinine and quinidine derivatives) and by dipping the TLC plates in sulfuric acid (50% aqueous solution) followed by heating with a heat gun. Plates were developed in a saturated chamber in eluent A: ACN/H_2O/conc. aq. NH_3 10:5:1 or in eluent B: $CHCl_3$/MeOH/conc. aq. NH_3 20:1:0.1.

For IR measurements, samples were mixed with KBr and measured on a Thermo Nicolet AVATAR 370 FT-IR spectrometer using DRIFT method. Specific optical rotation was done on Rudolph Research AUTOPOL III polarimeter at 589 nm (sodium D line) and values of $[\alpha]^{25}_{D}$ are reported together with used concentration (*c*, g/100 mL) and solvent.

Mass spectra (HRMS) were measured on Agilent 6530Q-TOF MS spectrometer. Samples were dissolved in a mixture of ACN/H_2O.

Chiral HPLC measurements were carried out using a LC20AD Shimadzu liquid chromatograph with SPD-M20A diode array detector with column Daicel Chiralpak® IB.

NMR spectra were recorded on a Varian VNMRS 300 ($v(^{1}H)$ = 299.94 MHz, $v(^{13}C)$ = 75.43 MHz) (for non-CD derivatives) or on a Bruker Avance III ($v(^{1}H)$ = 600.17 MHz, $v(^{13}C)$ = 150.04 MHz) in deuterated solvents and are referenced to residual solvent peak. Chemical shifts are given on the δ-scale; coupling constants *J* are given in Hz. NMR characterization was done by using ^{1}H, ^{13}C, DEPT and 2D NMR experiments such as COSY, HSQC and HMBC. The most common numbering of cinchona alkaloids atoms [1] for NMR spectra transcription was used (Figure S1).

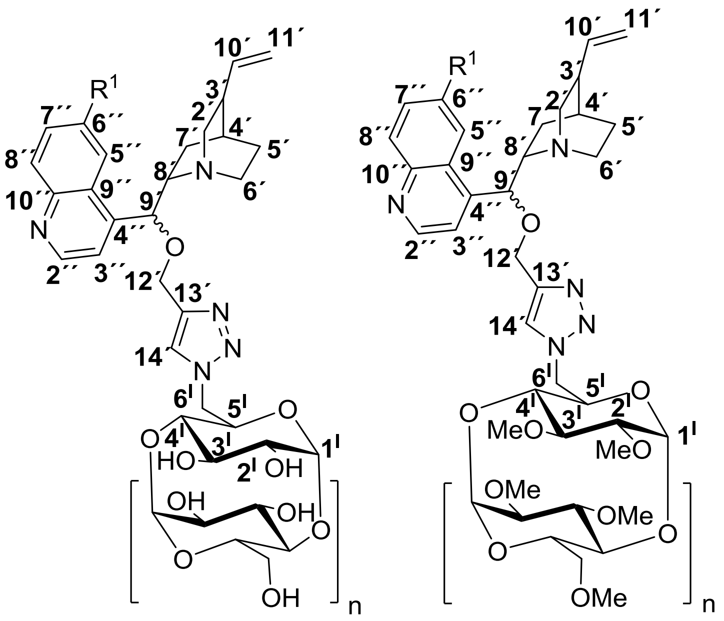

Figure S1: Numbering of atoms in cyclodextrin derivative structures used in NMR.

General information for NMR elucidation of CD catalysts

NMR elucidation of new CD derivatives was done using 2D NMR techniques; nevertheless, the exact positions of some peaks were problematic, particularly on the quinuclidine skeleton (5´, 6´, 7´, 8´, 9´). Especially, H-9´ (C-9´) signals are usually not seen in ^{1}H NMR or ^{13}C NMR, not even in ^{1}H-^{1}H COSY, HSQC or HMBC and also C-7´and C-5´ signals are not fully seen in some ^{13}C NMR spectra especially in case of non-methylated CDs.

General procedure for the preparation of monosubstituted non-methylated CD derivatives (GP1)

For α-CD derivatives: The starting material, 9-*O*-propargyl cinchona alkaloid **3a–d** (0.13 mmol), was dissolved in distilled (10 min-sonicated under Ar atmosphere) THF (0.5 mL). Then, 6^{I}-azido-6^{I}-deoxy-α-CD (**1**, 0.10 mmol), prepared according to the literature [2], was dissolved in distilled (10 min-sonicated under Ar atmosphere) H_2O (0.4 mL) and added to the stirred solution of the cinchona alkaloid. Finally, copper iodide (0.02 mmol), suspended in H_2O (0.1 mL), was added and the reaction mixture stirred at 50 °C for 2–3 h.

For β-CD derivatives: The starting material, 9-*O*-propargyl cinchona alkaloid **3a–3d** (0.11 mmol) was dissolved in distilled (10 min-sonicated under Ar atmosphere) DMF (0.5 mL). The second starting material, 6^{I}-azido-6^{I}-deoxy-β-CD [2] (**2**, 0.09 mmol), was dissolved in distilled (10 min-sonicated under Ar atmosphere) DMF (0.5 mL) and added to the stirring solution of the cinchona alkaloid. Finally, copper iodide (0.02 mmol), suspended in (10 min-sonicated under Ar atmosphere) DMF (0.1 mL), was added and the reaction mixture stirred at 60 °C for 2–3 h.

The conversion to product was monitored by TLC in eluent A. After full conversion, the reaction mixture was slowly added to stirring acetone (75 mL) resulting in white-yellow precipitate. The solid was recovered by filtration, washed with acetone (3 × 20 mL) and dried to constant weight in a vacuum drying box in the presence of P_2O_5 and KOH. The crude product was purified by column chromatography (50 g of silica gel) with ACN/H_2O/conc. aq. NH_3 12:5:1 as mobile phase. When the conversion to the product was incomplete, the starting material could be eventually recovered (eluted as the second compound). Eye-visible blue precipitation at the top of the column was observed as copper cations interact with ammonia. The purified product was dried at 50 °C in high vacuum.

6^{I}-Deoxy-6^{I}-(4-(((*S*)-quinolin-4-yl(5-vinylquinuclidin-2-yl)methoxy) methyl)-1*H*-1,2,3-triazol-1-yl)-α-CD (4a)

The compound **4a** was prepared according to the general procedure (**GP1**) from 6^{I}-azido-6^{I}-deoxy-α-CD (**1**, 164 mg, 0.16 mmol), 9-*O*-propargyl-cinchonine (**3a**, 71 mg, 0.2 mmol) and copper iodide (6 mg, 0.03 mmol). The product was obtained as white-yellow solid (168 mg, 77% yield). TLC: R_F = 0.45 (eluent A).

^{1}H NMR (DMSO-d$_6$): δ (ppm): 8.96 – 8.94 (m, 1H, H-2´´), 8.39 – 8.37 (m, 1H, H-8´´), 8.14 – 8.13 (s, 1H, H-14´), 8.10 – 8.08 (m, 1H, H-5´´), 7.82 – 7.80 (m, 1H, H-6´´), 7.70 – 7.68 (m, 1H, H-7´´), 7.64 – 7.63 (m, 1H, H-3´´), 5.89 – 5.87 (m, 1H, H-10´), 5.65 – 5.35 (m, 12H, OH-2, OH-3), 5.06 – 5.04 (m, 1H, H-1^{I}), 5.05 – 5.03 (m, 2H, H-11´), 4.86 – 4.72 (m, 5H, H-1), 4.84 – 4.83 (m, 2H, H-6^{I}), 4.58 – 4.50 (m, 5H, OH-6), 4.52 – 4.50 (m, 2H, H-12´), 4.02 – 4.00

(m, 1H, H-5[I]), 3.84 – 3.18 (m, 33H, H-2, H-3, H-4, H-5, H-6), 3.40 – 3.38 (m, 1H, H-8´), 3.14 – 3.12 (m, 2H, H-2´), 3.08 – 3.06 (m, 1H, H-6a´), 2.95 – 2.94 (m, 1H, H-6b´), 2.48 – 2.45 (m, 1H, H-3´), 1.81 – 1.79 (m, 1H, H-4´), 1.65 – 1.63 (m, 2H, H-5´), 1.29 – 1.27 (m, 2H, H-7´).

H-9´and C-9´signals are not seen.

^{13}C NMR (DMSO-d$_6$): δ (ppm): 150.24 (C-2´´), 147.97 (C-10´´*), 143.71 (C-4´´*), 142.67 (C-13´), 138.56 (C-10´), 129.81 (C-5´´), 129.34 (C-6´´), 126.98 (C-7´´), 126.14 (C-14´), 125.80 (C-9´´), 123.75 (C-8´´), 119.07 (C-3´´), 115.79 (C-11´), 101.59 (6 × C-1), 82.42 (6 × C-4), 72.62 (5 × C-5, 6 × C-3, 6 × C-2), 69.58 (C-5[I]), 61.93 (C-12´), 60.02 (5 × C-6), 59.36 (C-8´), 49.96 (C-6[I]), 48.62 (C-6´), 47.48 (C-2´), 37.25 (C-3´), 26.98 (C-4´), 23.80 (C-7´), 23.57 (C-5´).

Signals tagged with * can be mutually interchanged.

HRMS (ESI): found 1330.5064. For $C_{58}H_{83}N_5O_{30}$ $[M+H]^+$ calculated 1330.5196.

IR (KBr) 3291, 3105, 2938, 1640, 1413, 1240, 1150, 1076, 1039 cm^{-1}.

$[\alpha]_D^{25}$ = +61.7° (*c* = 0.30, DMSO).

6[I]-Deoxy-6[I]-(4-(((*R*)-quinolin-4-yl(5-vinylquinuclidin-2-yl)methoxy) methyl)-1*H*-1,2,3-triazol-1-yl)-α-CD (4b)

The compound **4b** was prepared according to the general procedure (**GP1**) from 6[I]-azido-6[I]-deoxy-α-CD (**1**, 100 mg, 0.10 mmol), 9-*O*-propargyl-cinchonidine (**3b**, 43 mg, 0.13 mmol) and copper iodide (4 mg, 0.02 mmol). The product was obtained as white-yellow solid (115 mg, 86% yield). TLC: R_F = 0.45 (eluent A).

^{1}H NMR (DMSO-d$_6$): δ (ppm): 8.96 – 8.94 (m, 1H, H-2´´), 8.43 – 8.40 (m, 1H, H-8´´), 8.27 – 8.24 (s, 2H, H-14´), 8.12 – 8.09 (m, 1H, H-5´´), 7.85 – 7.81 (m, 1H, H-6´´), 7.73 – 7.69 (m, 1H, H-7´´), 7.69 – 7.64 (m, 1H, H-3´´), 5.80 – 5.76 (m, 1H, H-10´), 5.67 – 5.34 (m, 12H, OH-2, OH-3), 5.04 – 5.00 (m, 1H, H-11a´), 5.01 – 4.99 (m, 1H, H-1[I]), 4.95 – 4.93 (m, 1H, H-1b´), 4.84 – 4.73 (m, 5H, H-1), 4.81 – 4.79 (m, 1H, H-6a[I]), 4.61 – 4.59 (m, 1H, H-6b[I]), 4.58 – 4.50 (m, 5H, OH-6), 4.55 – 4.52 (m, 1H, H-12a´), 4.50 – 4.47 (m, 1H, H-12b´), 4.10 – 4.08 (m, 1H, H-5[I]), 3.88 – 3.16 (m, 33H, H-2, H-3, H-4, H-5, H-6), 3.59 – 3.56 (m, 2H, H-6´), 3.51 – 3.48 (m, 1H, H-8´), 3.32 – 3.29 (m, 1H, H-2a´), 2.92 – 2.89 (m, 1H, H-2b´), 2.51 – 2.49 (m, 1H, H-3´), 1.89 – 1.85 (m, 1H, H-4´), 1.27 – 1.23 (m, 2H, H-5´), 1.24 – 1.21 (m, 2H, H-7´).

H-9´and C-9´signals are not seen.

^{13}C NMR (DMSO-d$_6$): δ (ppm): 150.19 (C-2´´), 147.99 (C-10´´*), 143.56 (C-4´´*), 142.85 (C-13´), 139.69 (C-10´), 129.77 (C-5´´), 129.39 (C-6´´), 127.14 (C-7´´), 125.94 (C-14´), 125.75 (C-9´´), 123.87 (C-8´´), 119.11 (C-3´´), 115.70 (C-11´), 101.30 (6 × C-1), 82.57 (6 × C-4),

72.35 (6 × C-2, 6 × C-3, 5 × C-5), 69.41 (C-5^{I}), 62.03 (C-12´), 60.14 (5 × C-6), 59.32 (C-8´), 53.92 (C-2´), 50.25 (C-6^{I}), 42.69 (C-6´), 37.45 (C-3´), 30.64 (C-7´), 26.62 (C-4´), 22.59 (C-5´).

Signals tagged with * can be mutually interchanged.

HRMS (ESI): found 1330.5151. For $C_{58}H_{83}N_5O_{30}$ $[M+H]^+$ calculated 1330.5196.

IR (KBr) 3288, 3166, 3105, 2926, 2361, 1156, 1120, 1075, 1036 cm^{-1}.

$[\alpha]_D^{25}$ = +25.8° (*c* = 0.33, DMSO).

6^{I}-Deoxy-6^{I}-((4-(((*S*)-(6-methoxyquinolin-4-yl)(5-vinylquinuclidin-2-yl)methoxy)methyl)-1*H*-1,2,3-triazol-1-yl)-α-CD (4c)

The compound **4c** was prepared according to the general procedure (**GP1**) from 6^{I}-azido-6^{I}-deoxy-α-CD (**1**, 316 mg, 0.3 mmol), 9-*O*-propargyl-quinine (**3c**, 149 mg, 0.4 mmol) and copper iodide (12 mg, 0.06 mmol). The product was obtained as white-yellow solid (306 mg, 72% yield). TLC: R_F = 0.45 (eluent A).

^{1}H NMR (DMSO-d$_6$): δ (ppm): 8.76 – 8.75 (m, 1H, H-2´´), 8.21 (s, 1H, H-14´), 7.98 – 7.96 (m, 1H, H-8´´), 7.87 – 7.86 (m, 1H, H-3´´), 7.56 – 7.55 (m, 1H, H-5´´), 7.44 – 7.42 (m, 1H, H-7´´), 5.82 – 5.76 (m, 1H, H-10´), 5.65 – 5.36 (m, 12H, OH-2, OH-3), 5.01 – 4.99 (m, 1H, H-11a´), 4.94 – 4.92 (m, 1H, H-11b´), 4.82 – 4.73 (m, 5H, H-1), 4.80 – 4.79 (m, 2H, H-6^{I}), 4.58 – 4.44 (m, 5H, OH-6), 4.52 – 4.50 (m, 1H, H-12a´), 4.47 – 4.45 (m, 1H, H-12b´), 4.05 – 4.04 (m, 1H, H-5^{I}), 3.95 (s, 3H, OC$\underline{H}_3$-6´´), 3.84 – 3.13 (m, 33H, H-2, H-3, H-4, H-5, H-6), 3.33 – 3.31 (m, 1H, 8´´), 3.24 – 3.22 (m, 1H, H-5^{I}), 3.13 – 3.12 (m, 2H, H-2´), 2.75 – 2.73 (m, 2H, H-6´), 2.38 – 2.36 (m, 1H, H-3´), 1.80 – 1.78 (m, 1H, H-4´), 1.69 – 1.65 (m, 1H, H-5a´), 1.55 – 1.52 (m, 1H, H-5b´), 1.53 – 1.51 (m, 2H, H-7´).

H-9´and C-9´signals are not seen.

^{13}C NMR (DMSO-d$_6$): δ (ppm): 157.41 (C-6´´), 147.50 (C-2´´), 147.20 (C-4´´*), 144.01 (C-10´´*), 143.12 (C-13´), 140.79 (C-10´), 131.26 (C-8´´), 127.09 (C-9´´), 126.07 (C-14´), 121.53 (C-7´´), 119.07 (C-3´´), 114.96 (C-11´), 102.33 (C-5´´), 101.90 (6 × C-1), 83.16 (C-4^{I}), 82.66 (5 × C-4), 72.22 (6 × C-2, 6 × C-3, 5 × C-5), 69.52 (C-5^{I}), 61.93 (C-12´), 60.18 (5 × C-6), 59.38 (C-8´), 55.97 (O$\underline{C}H_3$-6´´), 54.59 (C-2´), 50.12 (C-6^{I}), 42.39 (C-6´), 38.18 (C-3´), 26.79 (C-4´), 25.74 (C-5´), 23.18 (C-7´).

Signals tagged with * can be mutually interchanged.

HRMS (ESI): found 1360.5155. For $C_{59}H_{85}N_5O_{31}$ calculated $[M+H]^+$ 1360.5301.

IR (KBr) 3282, 2920, 1625, 1512, 1431, 1242, 1153, 1078, 1039 cm^{-1}.

$[\alpha]_D^{25}$ = +57.4° (*c* = 0.31, DMSO).

6^{I}-Deoxy-6^{I}-((4-(((*R*)-(6-methoxyquinolin-4-yl)(5-vinylquinuclidin-2-yl)methoxy)methyl)-1*H*-1,2,3-triazol-1-yl)-α-CD (4d)

The compound **4d** was prepared according to the general procedure (**GP1**) from 6^{I}-azido-6^{I}-deoxy-α-CD (**1**, 174 mg, 0.17 mmol), 9-*O*-propargyl-quinidine (**3d**, 82 mg, 0.23 mmol) and copper iodide (6 mg, 0.03 mmol). The product was obtained as white-yellow solid (176 mg, 74% yield). TLC: R_F = 0.45 (eluent A).

^{1}H NMR (DMSO-d$_6$): δ (ppm): 8.80 – 8.79 (m, 1H, H-2´´), 8.16 – 8.14 (m, 1H, H-14´), 8.00 – 7.99 (m, 1H, H-8´´), 7.60 – 7.56 (m, 1H, H-3´´), 7.57 – 7.55 (m, 1H, H-5´´), 7.48 – 7.45 (m, 1H, H-7´´), 5.88 – 5.81 (m, 1H, H-10´), 5.67 – 5.42 (m, 12H, OH-2, OH-3), 5.07 – 5.01 (m, 1H, H-1^{I}), 5.05 – 5.03 (m, 1H, H-11a´), 5.02 – 5.00 (m, 1H, H-11b´), 4.97 – 4.74 (m, 5H, H-1), 4.82– 4.80 (m, 1H, H-6a^{I}), 4.76 – 4.74 (m, 1H, H-6b^{I}), 4.64 – 4.50 (m, 5H, OH-6), 4.55 – 4.53 (m, 2H, H-12´), 3.99 (s, 3H, OC*H*$_3$-6´´), 3.98 (m, 1H, H-5^{I}), 3.86 – 3.18 (m, 33H, H-2, H-3, H-4, H-5, H-6), 3.69 – 3.67 (m, 1H, H-2a´), 3.49 – 3.47 (m, 1H, H-8´), 3.44 – 3.42 (m, 1H, H-9´), 3.28 – 3.26 (m, 1H, H-2b´), 3.23 – 3.21 (m, 1H, H-6a´), 3.07 – 3.05 (m, 1H, H-6b´), 2.53 – 2.51 (m, 1H, H-3´), 1.85 – 1.82 (m, 1H, H-4´), 1.71 – 1.69 (m, 2H, H-7´), 1.23 – 1.21 (m, 2H, H-5´).

^{13}C NMR (DMSO-d$_6$): δ (ppm): 157.73 (C-6´´), 147.57 (C-2´´), 144.05 (C-10´´*), 142.69 (C-13´), 141.43 (C-4´´*), 138.06 (C-10´), 131.34 (C-8´´), 126.64 (C-9´´), 126.13 (C-14´), 121.88 (C-7´´), 118.80 (C-3´´), 116.02 (C-11´), 101.89 (6 × C-1), 101.20 (C-5´´), 82.21 (6 × C-4), 73.10 (C-9´), 72.06 (6 × C-2, 6 × C-3, 5 × C-5), 69.66 (C-5^{I}), 62.02 (C-12´), 60.20 (5 × C-6), 58.81 (C-8´), 56.28 (O*C*H$_3$-6´´), 49.98 (C-6^{I}), 48.43 (C-6´), 47.25 (C-2´), 36.75 (C-3´), 28.98 (C-5´), 26.87 (C-4´), 23.12 (C-7´).

Signals tagged with * can be mutually interchanged.

HRMS (ESI): found 1360.5182. For $C_{59}H_{85}N_5O_{31}$ calculated $[M+H]^+$ 1360.5301.

IR (KBr) 3381, 3330, 2995, 1625, 1515, 1365, 1245, 1156, 1054 cm^{-1}.

$[\alpha]_D^{25}$ = +60.0° (*c* = 0.30, DMSO).

6^{I}-Deoxy-6^{I}-((4-(((*S*)-quinolin-4-yl(5-vinylquinuclidin-2-yl)methoxy)methyl)-1*H*-1,2,3-triazol-1-yl)-β-CD (5a)

The compound **5a** was prepared according to the general procedure (**GP1**) from 6^{I}-azido-6^{I}-deoxy-β-CD (**2**, 100 mg, 0.09 mmol) and 9-*O*-propargyl-cinchonine (**3a**, 37 mg, 0.11 mmol) and copper iodide (3 mg, 0.02 mmol). The product was obtained as white-yellow solid (114 mg, 89% yield). TLC: R_F = 0.39 (eluent A).

^{1}H NMR (DMSO-d$_6$): δ (ppm): 8.93 – 8.90 (m, 1H, H-2´´), 8.34 – 8.31 (m, 1H, H-8´´), 8.08 – 8.07 (m, 1H, H-5´´), 8.03 – 8.01 (m, 1H, H-14´), 7.82 – 7.76 (m, 1H, H-6´´), 7. 67 – 7.65 (m, 1H, H-7´´), 7.60 – 7.58 (m, 1H, H-3´´), 5.96 – 5.93 (m, 1H, H-10´), 5.85 – 5.64 (m, 14H, OH-2, OH-3), 5.05 – 4.91 (m, 7H, H-1), 5.03 – 5.01 (m, 2H, H-11´), 4.85 – 4.83 (m, 1H, H-6a^{I}), 4.64 – 4.62 (m, 1H, H-6b^{I}), 4.63 – 4.51 (m, 6H, OH-6), 4.46 – 4.44 (m, 1H, H-12a´), 4.38 – 4.36 (m, 1H, H-12b´), 3.98 – 3.96 (m, 1H, H-5^{I}), 3.79 – 3.24 (m, 39H, H-2, H-3, H-4, H-5, H-6), 3.17 – 3.15 (m, 1H, H-8´), 3.10 – 3.08 (m, 1H, H-2a´), 2.74 – 2.72 (m, 1H, H-2b´), 2.60 – 2.58 (m, 1H, H-6a´), 2.24 – 2.22 (m, 1H, H-3´), 1.83 – 1.81 (m, 1H, H-7a´), 1.69 – 1.67 (m, 1H, H-4´), 1.49 – 1.47 (m, 2H, H-5´), 1.48 – 1.46 (m, 1H, H-6b´), 1.43 – 1.41 (m, 1H, H-7b´).

H-9´signal is not seen.

^{13}C NMR (DMSO-d$_6$): δ (ppm): 150.13 (C-2´´), 147.93 (C-10´´*), 146.21 (C-4´´*), 143.20 (C-13´), 140.29 (C-10´), 129.75 (C-5´´), 129.50 (C-9´´), 129.06 (C-6´´), 126.54 (C-7´´), 125.40 (C-14´), 123.94 (C-8´´), 119.70 (C-3´´), 114.84 (C-11´), 101.94 (7 × C-1), 81.60 (7 × C-4), 80.60 (C-9´), 72.22 (7 × C-2, 7 × C-3, 6 × C-5), 69.83 (C-5^{I}), 61.71 (C-12´), 60.10 (6 × C-6), 60.05 (C-8´), 50.11 (C-6^{I}), 47.85 (C-2´), 39.50 (C-3´), 27.39 (C-4´), 25.41 (C-5´), 48.89 (C-6´), 25.41 (C-7´).

Signals tagged with * can be mutually interchanged.

HRMS (ESI): found 1492.5610. For $C_{64}H_{93}N_5O_{35}$ calculated $[M+H]^+$ 1492.5724.

IR (KBr) 3348, 3105, 2929, 1646, 1422, 1156, 1105, 1081, 1033 cm^{-1}.

$[\alpha]_D^{25}$ = + 75.0° (*c* = 0.30, DMSO).

6^{I}-Deoxy-6^{I}-((4-(((*R*)-quinolin-4-yl(5-vinylquinuclidin-2-yl)methoxy)methyl)-1*H*-1,2,3-triazol-1-yl)-β-CD (5b)

The compound **5b** was prepared according to the general procedure (**GP1**) from 6^{I}-azido-6^{I}-deoxy-β-CD (**2**, 100 mg, 0.09 mmol), 9-*O*-propargyl-cinchonidine (**3b**, 37 mg, 0.11 mmol) and copper iodide (3 mg, 0.02 mmol). The product was obtained as white-yellow solid (90 mg, 70% yield). TLC: R_F = 0.39 (eluent A).

^{1}H NMR (DMSO-d$_6$): δ (ppm): 8.93 – 8.91 (m, 1H, H-2´´), 8.39 – 8.37 (m, 1H, H-8´´), 8.09 – 8.07 (m, 1H, H-5´´), 8.08 – 8.07 (m, 1H, H-14´), 7.80 – 7.78 (m, 1H, H-6´´), 7.66 (m, 1H, H-7´´), 7.61 (m, 1H, H-3´´), 5.81 – 5.79 (m, 1H, H-10´), 5.79 – 5.64 (m, 14H, OH-2, OH-3), 5.08 – 5.07 (m, 1H, H-1^{I}), 5.00 – 4.98 (m, 1H, H-11a´), 4.93 – 4.91 (m, 1H, H-11b´), 4.87 – 4.85 (m, 1H, H-6a^{I}), 4.85 – 4.81 (m, 6H, H-1), 4.63 – 4.49 (m, 6H, OH-6), 4.61 – 4.58 (m, 1H, H-6b^{I}), 4.45 – 4.43 (m, 2H, H-12´), 3.99 – 3.97 (m, 1H, H-5^{I}), 3.78 – 3.26 (m, 39H, H-2, H-3, H-4, H-5, H-6), 3.31 – 3.29 (m, 1H, H-6a´), 3.27 – 3.25 (m, 1H, H-8´), 3.00 – 2.98 (m, 1H, H-

2a´), 2.61 – 2.59 (m, 1H, H-6b´), 2.60 – 2.58 (m, 1H, H-2b´), 2.32 – 2.30 (m, 1H, H-3´), 1.77 – 1.75 (m, 1H, H-4´), 1.69 – 1.67 (m, 1H, H-5a´), 1.51 – 1.49 (m, 1H, H-5b´), 1.64 – 1.61 (m, 2H, H-7´).

H-9´and C-9´ signals are not seen.

^{13}C NMR (DMSO-d$_6$): δ (ppm): 150.13 (C-2´´), 147.97 (C-10´´), 145.50 (C-4´´), 143.14 (C-13´), 141.24 (C-10´), 129.75 (C-5´´), 129.17 (C-6´), 126.73 (C-7´´), 126.24 (C-9´´), 125.40 (C-14´), 123.94 (C-8´´), 119.46 (C-3´´), 114.76 (C-11´), 101.83 (7 × C-1), 81.75 (7 × C-4), 72.46 (7 × C-2, 7 × C-3, 6 × C-5), 69.81 (C-5^{I}), 61.86 (C-12´), 60.10 (6 × C-6), 59.05 (C-8´), 55.14 (C-2´), 50.20 (C-6^{I}), 42.13 (C-6´), 38.61 (C-3´), 26.20 (C-5´), 26.97 (C-4´), 22.96 (C-7´).

Signals tagged with * can be mutually interchanged.HRMS (ESI): found 1492.5604. For $C_{64}H_{93}N_5O_{35}$ calculated $[M+H]^+$ 1492.5724.

IR (KBr) 3297, 2932, 1661, 1637, 1509, 1461, 1362, 1335, 1030 cm^{-1}.

$[\alpha]_D^{25}$ = +48.3° (*c* = 0.30, DMSO).

6^{I}-Deoxy-6^{I}-((4-(((*S*)-(6-methoxyquinolin-4-yl)(5-vinylquinuclidin-2-yl)methoxy)methyl)-1*H*-1,2,3-triazol-1-yl)-β-CD (5c)

The compound **5c** was prepared according to the general procedure (**GP1**) from 6^{I}-azido-6^{I}-deoxy-β-CD (**2**, 100 mg, 0.09 mmol), 9-*O*-propargyl-quinine (**3c**, 41 mg, 0.11 mmol) and copper iodide (3 mg, 0.02 mmol). The product was obtained as white-yellow pure product (105 mg, 80% yield). TLC: R_F = 0.43 (eluent A).

^{1}H NMR (DMSO-d$_6$): δ (ppm): 8.78 – 8.76 (m, 1H, H-2´´), 8.21 – 8.19 (m, 1H, H-14´), 7.99 – 7.97 (m, 1H, H-8´´), 7.66 – 7.64 (m, 1H, H-5´´), 7.59 – 7.57 (m, 1H, H-3´´), 7.46 – 7.44 (m, 1H, H-7´´), 5.84 – 5.71 (m, 14H, OH-2, OH-3), 5.82 – 5.80 (m, 1H, H-10´), 5.07 – 5.05 (m, 1H, H-11a´), 5.04 – 4.95 (m, 7H, H-1), 4.94 – 4.92 (m, 1H, H-11b´), 4.95 – 4.93 (m, 1H, H-6a^{I}), 4.64 – 4.50 (m, 6H, OH-6), 4.59 – 4.57 (m, 1H, H-6b^{I}), 4.50 – 4.48 (m, 2H, H-12´), 4.10 – 4.08 (m, 1H, H-5^{I}), 3.97 (s, 3H, OC$\underline{H}_3$-6´´), 3.78 – 3.16 (m, 39H, H-2, H-3, H-4, H-5, H-6), 3.07 – 3.05 (m, 1H, H-8´), 3.75 – 3.73 (m, 2H, H-2´), 2.54 – 2.52 (m, 2H, H-6´), 2.42 – 2.40 (m, 1H, H-3´), 1.85 – 1.83 (m, 1H, H-4´), 1.77 – 1.75 (m, 1H, H-5a´), 1.59 – 1.57 (m, 1H, H-5b´), 1.30 – 1.28 (m, 2H, H-7´).

H-9´and C-9´ signals are not seen.

^{13}C NMR (DMSO-d$_6$): δ (ppm): 157.58 (C-6´´), 147.44 (C-2´´), 144.01 (C-10´´*), 144.01 (C-4´´*), 143.11 (C-13´), 140.31 (C-10´), 131.17 (C-8´´), 127.09 (C-9´´), 125.34 (C-14´), 121.67 (C-7´´), 118.93 (C-3´´), 115.17 (C-11´), 102.16 (C-5´´), 101.50 (7 × C-1), 83.56 (C-4^{I}), 81.70

(6 × C-4), 73.12 (7 × C-2, 7 × C-3, 6 × C-5), 61.95 (C-12´), 69.76 (C-5[I]), 60.05 (6 × C-6), 59.30 (C-8´), 56.07 (O<u>C</u>H$_3$-6´´), 54.39 (C-2´), 50.38 (C-6[I]), 42.53 (C-6´), 38.15 (C-3´), 26.85 (C-4´), 25.52 (C-5´), 25.15 (C-7´).

Signals tagged with * can be mutually interchanged.

HRMS (ESI): found 1522.5741. For $C_{65}H_{95}N_5O_{36}$ calculated $[M+H]^+$ 1522.5831.

IR (KBr) 3396, 3330, 3073, 2935, 1655, 1622, 1419, 1237, 1030 cm^{-1}.

$[\alpha]_D^{25}$ = + 46.7° (*c* = 0.30, DMSO).

6[I]-Deoxy-6[I]-((4-(((*R*)-(6-methoxyquinolin-4-yl)(5-vinylquinuclidin-2-yl)methoxy)methyl)-1*H*-1,2,3-triazol-1-yl)-β-CD (5d)

The compound **5d** was prepared according to the general procedure (**GP1**) from 6[I]-azido-6[I]-deoxy-β-CD (**2**, 100 mg, 0.09 mmol), 9-*O*-propargyl-quinidine (**3d**, 41 mg, 0.11 mmol) and copper iodide (3 mg, 0.02 mmol). The product was obtained as white-yellow solid (124 mg, 95% yield). TLC: R_F = 0.43 (eluent A).

^{1}H NMR (DMSO-d$_6$): δ (ppm): 8.78 – 8.76 (m, 1H, H-2´´), 8.09 – 8.05 (m, 1H, H-14´), 8.00 – 7.98 (m, 1H, H-8´´), 7.58 – 7.56 (m, 1H, H-5´´), 7.59 – 7.57 (m, 1H, H-3´´), 7.46 – 7.44 (m, 1H, H-7´´), 5.93 – 5.88 (m, 1H, H-10´), 5.86 – 5.70 (m, 14H, OH-2, OH-3), 5.05 – 4.75 (m, 7H, H-1), 5.02 – 5.00 (m, 2H, H-11´), 4.85 – 4.83 (m, 1H, H-6a[I]), 4.64 – 4.50 (m, 6H, OH-6), 4.63 – 4.60 (m, 1H, H-6b[I]), 4.49 – 4.46 (m, 1H, H-12a´), 4.40 – 4.38 (m, 1H, H-12b´), 3.98 – 3.95 (m, 1H, H-5[I]), 3.96 (s, 3H, OC<u>*H*</u>$_3$-6´´), 3.78 – 3.16 (m, 39H, H-2, H-3, H-4, H-5, H-6), 3.07 – 3.05 (m, 1H, H-8´), 3.75 – 3.73 (m, 2H, H-2´), 2.54 – 2.52 (m, 2H, H-6´), 2.30 – 2.28 (m, 1H, H-3´), 1.73 – 1.71 (m, 1H, H-4´), 1.65 – 1.62 (m, 2H, H-5´), 1.30 – 1.28 (m, 2H, H-7´).

H-9´and C-9´signals are not seen.

^{13}C NMR (DMSO-d$_6$): δ (ppm): 157.20 (C-6´´), 147.58 (C-2´´), 144.0 (C-10´´*), 144.00 (C-4´´*), 143.17 (C-13´), 140.29 (C-10´), 131.24 (C-8´´), 127.31 (C-9´´), 125.41 (C-14´), 121.46 (C-7´´), 119.31 (C-3´´), 115.10 (C-11´), 101.50 (7 × C-1), 100.16 (C-5´´), 83.26 (C-4[I]), 81.70 (6 × C-4), 73.12 (7 × C-2, 7 × C-3, 6 × C-5), 69.77 (C-5[I]), 61.66 (C-12´), 60.05 (6 × C-6), 59.30 (C-8´), 55.75 (O<u>C</u>H$_3$-6´´), 50.17 (C-6[I]), 48.76 (C-6´), 47.74 (C-2´), 38.15 (C-3´), 27.25 (C-4´), 25.52 (C-5´), 25.15 (C-7´).

Signals tagged with * can be mutually interchanged.

HRMS (ESI): found 1522.5646. For $C_{65}H_{95}N_5O_{36}$ calculated $[M+H]^+$ 1522.5831.

IR (KBr) 3321, 2923, 2881, 1658, 1622, 1350, 1299, 1156, 1030 cm^{-1}.

$[\alpha]_D^{25}$ = +76.7° (*c* = 0.30, DMSO).

General procedure for the preparation of monosubstituted permethylated CD derivatives (GP2)

Starting material, 9-*O*-propargyl cinchona alkaloids **3a–d** (0.10 mmol) was dissolved in distilled (10 min-sonicated under Ar atmosphere) DMF (0.5 mL). The second starting material, 6^I-azido-6^I-deoxy-permethyl-α-CD [3] (**6**, 0.08 mmol) or 6^I-azido-6^I-deoxy-permethyl-β-CD [4] (**7**, 0.06 mmol), was dissolved in distilled (10 min-sonicated under Ar atmosphere) DMF (0.4 mL) and added to the solution of the cinchona alkaloid. Copper iodide (0.02 mmol), suspended in 10 min-sonicated under Ar atmosphere DMF (0.1 mL), was added to the reaction mixture and the mixture stirred at 50 °C for 16 h. The conversion to product was monitored on TLC in eluent B. After the starting material **6** or **7** disappeared on TLC, DMF was evaporated on rotary evaporator at 50 °C and the crude product was purified by column chromatography (50 g of silica gel) with $CHCl_3$/MeOH/conc. aq. NH_3 25:1:0.1 to 20:1:0.1 as mobile phase. When the conversion to the product was incomplete, the starting material could be eventually recovered (eluted as the first compound). Eye-visible blue precipitation at the top of the column was observed as copper cations interact with ammonia. The purified product was dried at 50 °C in high vacuum.

6^I-Deoxy-6^I-(4-(((*S*)-quinolin-4-yl(5-vinylquinuclidin-2-yl)methoxy) methyl)-1*H*-1,2,3-triazol-1-yl)-permethyl-α-CD (8a)

The compound **8a** was prepared according to the general procedure (**GP2**) from 6^I-azido-6^I-deoxy-permethyl-α-CD (**6**, 100 mg, 0.08 mmol), 9-*O*-propargyl-cinchonine (**3a**, 35 mg, 0.10 mmol) and copper iodide (3 mg, 0.02 mmol). The product was obtained white-yellow solid (75 mg, 59% yield). TLC: R_F = 0.40 (eluent B).

^{1}H NMR (MeOH-d*₄*): δ (ppm): 8.88 – 8.87 (m, 1H, H-2´´), 8.29 – 8.27 (m, 1H, H-8´´), 8.11 – 8.09 (m, 1H, H-5´´), 7.94 (s, 1H, H-14´), 7.83 – 7.80 (m, 1H, H-6´´), 7.72 – 7.69 (m, 1H, H-3´´), 7.71 – 7.68 (m, 1H, H-7´´), 6.03 – 5.97 (m, 1H, H-10´), 5.33 – 5.32 (m, 1H, H-1^I), 5.07 – 5.01 (m, 5H, H-1), 5.04 – 5.00 (m, 2H, H-11´), 4.93 – 4.90 (m, 2H, H-6^I), 4.58 (*b*s, 2H, H-12´), 4.21 – 4.18 (m, 1H, H-5^I), 4.12 – 3.89 (m, 5H, H-5), 3.91 – 3.79 (m, 10H, H-6), 3.66 – 3.62 (m, 18H, OC$\underline{H}_3$-3), 3.57 – 3.55 (m, 1H, H-9´), 3.55 – 3.49 (m, 18H, OC$\underline{H}_3$-2), 3.61 – 3.49 (m, 12H, H-3, H-4), 3.37 – 3.35 (m, 1H, H-2a´), 3.32 – 3.25 (m, 15H, OC$\underline{H}_3$-6), 3.21 – 3.23 (m, 1H, H-8´), 3.19 – 3.10 (m, 6H, H-2), 2.89 – 2.86 (m, 1H, H-6a´), 2.88 – 2.82 (m, 1H, H-2b´), 2.77 – 2.73 (m, 1H, H-6b´), 2.32 – 2.29 (m, 1H, H-3´), 2.09 – 2.06 (m, 1H, H-7a´), 1.73 – 1.72 (m, 1H, H-4´), 1.60 – 1.51 (m, 2H, H-5´), 1.32 – 1.28 (m, 1H, H-7b´).

^{13}C NMR (MeOH-d$_4$): δ (ppm): 150.98 (C-2´´), 149.19 (C-10´´*), 147.89 (C-4´´*), 144.95 (C-13´), 141.46 (C-10´), 130.98 (C-6´´), 130.31 (C-5´´), 128.43 (C-7´´), 127.89 (C-9´´), 127.48 (C-14´), 124.59 (C-8´´), 120.73 (C-3´´), 115.52 (C-11´), 100.19 (6 × C-1), 83.07 (6 × C-2, 6 × C-3, 6 × C-4), 81.03 (C-9´), 72.83 (5 × C-5, 5 × C-6), 71.66 (C-5^{I}), 63.50 (C-12´), 62.05 (6 × $O\underline{C}H_3$-3), 61.35 (C-8´), 59.39 (5 × $O\underline{C}H_3$-6), 58.64 (6 × $O\underline{C}H_3$-2), 52.41 (C-6^{I}), 50.81 (C-6´), 50.09 (C-2´), 40.94 (C-3´), 29.43 (C-4´), 26.95 (C-5´), 23.11 (C-7´).

Signals tagged with * can be mutually interchanged.

HRMS (ESI): found 1568.7713. For $C_{75}H_{117}N_5O_{30}$ $[M+H]^+$ calculated 1568.7856.

IR (KBr) 2926, 2830, 1715, 1512, 1461, 1365, 1196, 1168, 1036 cm^{-1}.

$[\alpha]_D^{25}$ = +139.0° (*c* = 0.30, MeOH).

6^{I}-Deoxy-6^{I}-(4-(((*R*)-quinolin-4-yl(5-vinylquinuclidin-2-yl)methoxy) methyl)-1*H*-1,2,3-triazol-1-yl)-permethyl-α-CD (8b)

The compound **8b** was prepared according to the general procedure (**GP2**) from 6^{I}-azido-6^{I}-deoxy-permethyl-α-CD (**6**, 100 mg, 0.08 mmol), 9-*O*-propargyl-cinchonidine (**3b**, 35 mg, 0.10 mmol) and copper iodide (3 mg, 0.02 mmol). The product was obtained as white-yellow solid (61 mg, 48% yield). TLC: R_F = 0.40 (eluent B).

^{1}H NMR (MeOH-d$_4$): δ (ppm): 8.87 – 8.85 (m, 1H, H-2´´), 8.33 – 8.29 (m, 1H, H-8´´), 8.11 – 8.08 (m, 1H, H-5´´), 7.97 (s, 1H, H-14´), 7.82 – 7.80 (m, 1H, H-6´´), 7.73 – 7.70 (m, 1H, H-3´´), 7.70 – 7.68 (m, 1H, H-7´´), 5.80 – 5.75 (m, 1H, H-10´), 5.32 – 5.31 (m, 1H, H-1^{I}), 5.07 – 4.97 (m, 5H, H-1), 4.97 – 4.95 (m, 1H, H-6a^{I}), 4.93 – 4.90 (m, 2H, H-11´), 4.89 – 4.86 (m, 1H, H-6b^{I}), 4.58 (*b*s, 2H, H-12´), 4.18 – 4.16 (m, 1H, H-5^{I}), 4.02 – 3.96 (m, 5H, H-5), 3.98 – 3.85 (m, 10H, H-6), 3.65 – 3.59 (m, 18H, $OC\underline{H}_3$-3), 3.60 – 3.56 (m, 12H, H-3, H-4), 3.58 – 3.47 (m, 18H, $OC\underline{H}_3$-2), 3.41 – 3.40 (m, 1H, H-6a´), 3.36 – 3.27 (m, 15H, $OC\underline{H}_3$-6), 3.22 – 3.19 (m, 1H, H-8´), 3.07 – 3.05 (m, 1H, H-2a´), 3.10 – 2.99 (m, 6H, H-2), 2.66 – 2.64 (m, 1H, H-6b´), 2.59 – 2.56 (m, 1H, H-2b´), 2.35 – 2.33 (m, 1H, H-3´), 1.79 – 1.76 (m, 1H, H-4´), 1.67 – 1.62 (m, 1H, H-5a´), 1.60 – 1.54 (m, 1H, H-5b´), 1.37 – 1.33 (m, 1H, H-7a´), 1.28 – 1.24 (m, 1H, H-7b´).

H-9´signal is not seen.

^{13}C NMR (MeOH-d$_4$): δ (ppm): 151.00 (C-2´´), 149.21 (C-10´´*), 148.07 (C-4´´*), 145.07 (C-13´), 142.53 (C-10´), 131.00 (C-6´´), 130.33 (C-5´´), 128.46 (C-7´´), 127.93 (C-9´´), 127.43 (C-14´), 124.65 (C-8´´), 120.54 (C-3´´), 115.11 (C-11´), 100.27 (6 × C-1), 83.04 (6 × C-4, 6 × C-3, 6 × C-2), 81.30 (C-9´), 72.50 (5 × C-5, 5 × C-6), 71.69 (C-5^{I}), 63.50 (C-12´), 62.03 (6 × $O\underline{C}H_3$-3), 59.57 (5 × $O\underline{C}H_3$-6), 58.90(6 × $O\underline{C}H_3$-2), 61.73 (C-8´), 57.46 (C-2´), 52.45 (C-6^{I}), 43.88 (C-6´), 40.81 (C-3´), 29.02 (C-4´), 28.25 (C-5´), 23.48 (C-7´).

Signals tagged with * can be mutually interchanged.

HRMS (ESI): found 1568.7708. For $C_{75}H_{117}N_5O_{30}$ calculated $[M+H]^+$ 1568.7856.

IR (KBr) 2920, 2839, 1595, 1455, 1359, 1165, 1111, 1069, 1039 cm^{-1}.

$[\alpha]_D^{25}$ = +75.0° (*c* = 0.30, MeOH).

6^I-Deoxy-6^I-(4-(((*R*)-1-(6-methoxyquinolin-4-yl(5-vinylquinuclidin-2-yl)methoxy) methyl)-1*H*-1,2,3-triazol-1-yl)-permethyl-α-CD (8c)

The compound **8c** was prepared according to the general procedure (**GP2**) from 6^I-azido-6^I-deoxy-permethyl-α-CD (**6**, 100 mg, 0.08 mmol), 9-*O*-propargyl-quinine (**3c**, 38 mg, 0.10 mmol) and copper iodide (4 mg, 0.02 mmol). The product was obtained as white-yellow solid (63 mg, 49% yield). TLC: R_F = 0.40 (eluent B).

^{1}H NMR (MeOH-d$_4$): δ (ppm): 8.71 – 8.70 (m, 1H, H-2´´), 7.99 – 7.98 (m, 1H, H-8´´), 7.98 (s, 1H, H-14´), 7.69 – 7.67 (m, 1H, H-3´´), 7.53 – 7.50 (m, 1H, H-5´´), 7.48 – 7.45 (m, 1H, H-7´´), 5.81 – 5.74 (m, 1H, H-10´), 5.34 – 5.28 (m, 1H, H-1^I), 5.00 – 4.88 (m, 5H, H-1), 4.97 – 4.92 (m, 2H, H-11´), 4.93 – 4.91 (m, 2H, H-6^I), 4.59 (s, 2H, H-12´), 4.19 – 4.16 (m, 1H, H-5^I), 3.99 (s, 3H, OC*H*$_3$-6´´), 3.92 – 3.81 (m, 5H, H-5) 3.82 – 3.25 (m, 10H, H-6), 3.67 – 3.60 (m, 18H, OC*H*$_3$-3), 3.63 – 3.61 (m, 1H, H-8´), 3.55 – 3.53 (m, 1H, H-9´), 3.52 – 3.45 (m, 18H, OC*H*$_3$-2), 3.61 – 3.41 (m, 12H, H-3, H-4), 3.37 – 3.25 (m, 15H, OC*H*$_3$-6), 3.21 – 3.12 (m, 6H, H-2), 3.08 – 3.05 (m, 1H, H-2a´), 2.69 – 2.66 (m, 2H, H-6´), 2.61 – 2.59 (m, 1H, H-2b´), 2.38 – 2.33 (m, 1H, H-3´), 1.80 – 1.79 (m, 1H, H-4´), 1.77 – 1.75 (m, 1H, H-5a´), 1.58 – 1.56 (m, 1H, H-5b´), 1.33 – 1.28 (m, 2H, H-7´).

^{13}C NMR (MeOH-d$_4$): δ (ppm): 159.90 (C-6´´), 148.22 (C-2´´), 146.39 (C-10´´*), 145.18 (C-4´´*), 145.12 (C-13´), 142.60 (C-10´), 131.65 (C-8´´), 128.98 (C-9´´), 127.42 (C-14´), 123.63 (C-7´´), 120.40 (C-3´´), 102.55 (C-5´´), 100.26 (6 × C-1), 83.10 (6 × C-2, 6 × C-3, 6 × C-4), 79.47 (C-9´), 72.42 (6 × C-5, 6 × C-6), 63.40 (C-12´), 62.07 (6 × O*C*H$_3$-3), 61.16 (C-8´), 59.29 (6 × O*C*H$_3$-2), 58.99 (5 × O*C*H$_3$-6), 57.50 (C-2´), 56.56 (O*C*H$_3$-6´´), 52.44 (C-6^I), 44.08 (C-6´), 40.84 (C-3´), 29.07 (C-4´), 28.32 (C-5´), 22.51 (C-7´).

Signals tagged with * can be mutually interchanged.

HRMS (ESI): found 1568.7849. For $C_{76}H_{119}N_5O_{31}$ $[M+H]^+$ calculated 1598.7962.

IR (KBr) 2926, 2836, 1622, 1506, 1476, 1365, 1228, 1141, 1036 cm^{-1}.

$[\alpha]_D^{25}$ = +92.4° (*c* = 0.21, MeOH).

6^{I}-Deoxy-6^{I}-(4-(((*S*)-1-(6-methoxyquinolin-4-yl(5-vinylquinuclidin-2-yl)methoxy) methyl)-1*H*-1,2,3-triazol-1-yl)-permethyl-α-CD (8d)

The compound **8d** was prepared according to the general procedure (**GP2**) from 6^{I}-azido-6^{I}-deoxy-permethyl-α-CD (**6**, 100 mg, 0.08 mmol), 9-*O*-propargyl-quinidine (**3d**, 38 mg, 0.10 mmol) and copper iodide (4 mg, 0.02 mmol). The product was obtained as white-yellow solid (54 mg, 42% yield). TLC: R_F = 0.40 (eluent B).

^{1}H NMR (MeOH-d$_4$): δ (ppm): 8.71 – 8.70 (m, 1H, H-2´´), 7.99 – 7.98 (m, 1H, H-8´´), 7.96 (s, 1H, H-14´), 7.66 – 7.65 (m, 1H, H-3´´), 7.51 – 7.50 (m, 1H, H-5´´), 7.46 – 7.44 (m, 1H, H-7´´), 6.00 – 5.97 (m, 1H, H-10´), 5.33 – 5.32 (m, 1H, H-1^{I}), 5.08 –5.00 (m, 5H, H-1), 5.05 – 5.03 (m, 2H, H-11´), 4.92 – 4.91 (m, 2H, H-6^{I}), 4.60 – 4.58 (m, 1H, H-9´), 4.55 (s, 2H, H-12´), 4.20 – 4.16 (m, 1H, H-5^{I}), 3.99 (s, 3H, OC$\underline{H}_3$-6´´), 3.91 – 3.81 (m, 4H, H-5), 3.91 – 3.61 (m, 10H, H-6), 3.69 – 3.63 (m, 18H, OC$\underline{H}_3$-3), 3.65 – 3.21 (m, 12H, H-3, H-4), 3.49 – 3.41 (m, 18H, OC$\underline{H}_3$-2), 3.34 – 3.32 (m, 1H, H-2a´), 3.25 – 3.31 (m, 15H, OC$\underline{H}_3$-6), 3.12 – 3.10 (m, 1H, H-8´), 3.21 – 3.05 (m, 6H, H-2), 2.88 – 2.85 (m, 1H, H-2b´), 2.87 – 2.85 (m, 1H, H-6a´), 2.79 – 2.74 (m, 1H, H-6b´), 2.31 – 2.28 (m, 1H, H-3´), 2.11 – 2.08 (m, 1H, H-7a´), 1.72 – 1.70 (m, 1H, H-4´), 1.60 – 1.53 (m, 2H, H-5´), 1.32 – 1.24 (m, 1H, H-7b´).

^{13}C NMR (MeOH-d$_4$): δ (ppm): 159.82 (C-6´´), 148.22 (C-2´´), 146.37 (C-10´´*), 145.15 (C-4´´*), 145.05 (C-13´), 141.64 (C-10´), 131.60 (C-8´´), 128.92 (C-9´´), 127.46 (C-14´), 123.59 (C-7´´), 120.54 (C-3´´), 115.42 (C-11´), 102.59 (C-5´´), 100.27 (6 × C-1), 83.08 (6 × C-2, 6 × C-3, 6 × C-4), 81.33 (C-9´), 72.33 (5 × C-5, 5 × C-6), 71.67 (C-5^{I}), 63.44 (C-12´), 61.11 (6 × O$\underline{C}H_3$-3), 60.88 (C-8´), 59.80 (6 × O$\underline{C}H_3$-2), 58.52 (5 × O$\underline{C}H_3$-6), 56.45 (O$\underline{C}H_3$-6´´), 52.41 (C-6^{I}), 50.85 (C-6´), 50.24 (C-2´), 41.06 (C-3´), 29.50 (C-4´), 27.07 (C-5´), 22.66 (C-7´).

Signals tagged with * can be mutually interchanged.

HRMS (ESI): found 1598.7962. For $C_{76}H_{119}N_5O_{31}$ $[M+H]^+$ calculated 1598.7859.

IR (KBr) 2929, 2839, 1622, 1512, 1456, 1368, 1257, 1144, 1039 cm^{-1}.

$[\alpha]_D^{25}$ = +154.8° (*c* = 0.20, MeOH).

6^{I}-Deoxy-6^{I}-(4-(((*S*)-quinolin-4-yl(5-vinylquinuclidin-2-yl)methoxy) methyl)-1*H*-1,2,3-triazol-1-yl)-permethyl-β-CD (9a)

The compound **9a** was prepared according to the general procedure (**GP2**) from 6^{I}-azido-6^{I}-deoxy-permethyl-β-CD (**7**, 100 mg, 0.07 mmol), 9-*O*-propargylcinchonine (**3a**, 29 mg, 0.09 mmol) and copper iodide (3 mg, 0.01 mmol). The product was obtained as white-yellow solid (38 mg, 64% yield). TLC: R_F = 0.38 (eluent B).

^{1}H NMR (MeOH-d$_4$): δ (ppm): 8.90 – 8.89 (m, 1H, H-2´´), 8.32 – 8.29 (m, 1H, H-8´´), 8.12 – 8.10 (m, 1H, H-5´´), 7.94 (s, 1H, H-14´), 7.84 – 7.81 (m, 1H, H-6´´), 7.72 – 7.69 (m, 1H, H-3´´), 7.71 – 7.68 (m, 1H, H-7´´), 6.05 – 5.99 (m, 1H, H-10´), 5.55 – 5.21 (m, 7H, H-1), 5.04 – 5.01 (m, 1H, H-6a^{I}), 5.04 – 5.03 (m, 2H, H-11´), 4.80 – 4.79 (m, 1H, H-6b^{I}), 4.57 (s, 2H, H-12´), 4.09 – 4.07 (m, 1H, H-5^{I}), 3.88 – 3.50 (m, 18H, H-5, H-6), 3.65 – 3.41 (m, 14H, H-3, H-4), 3.65 – 3.59 (m, 21H, OC$\underline{H}_3$-3), 3.52 – 3.43 (m, 21H, OC$\underline{H}_3$-2), 3.36 – 3.25 (m, 18H, OC$\underline{H}_3$-6), 3.28 – 3.26 (m, 1H, H-2a´), 3.18 – 3.16 (m, 1H, H-8´), 3.19 – 3.10 (m, 7H, H-2), 2.86 – 2.84 (m, 1H, H-2b´), 2.84 – 2.81 (m, 1H, H-6a´), 2.74 – 2.73 (m, 1H, H-6b´), 2.33 – 2.29 (m, 1H, H-3´), 2.10 – 2.07 (m, 1H, H-7a´), 1.73 – 1.72 (m, 1H, H-4´), 1.58 – 1.54 (m, 2H, H-5´), 1.31 – 1.28 (m, 1H, H-7b´).

H-9´signal is not seen.

^{13}C NMR (MeOH-d$_4$): δ (ppm): 150.99 (C-2´´), 149.18 (C-10´´*), 148.04 (C-4´´*), 144.98 (C-13´), 141.59 (C-10´), 130.96 (C-6´´), 130.29 (C-5´´), 128.41 (C-7´´), 127.91 (C-9´´), 127.46 (C-14´), 124.62 (C-8´´), 120.81 (C-3´´), 115.45 (C-11´), 99.67 (7 × C-1), 83.65 (7 × C-4, 7 × C-2), 81.67 (C-9´), 81.03 (7 × C-3), 72.87 (6 × C-5, 6 × C-6), 71.81 (C-5^{I}), 63.45 (C-12´), 62.14 (7 × O$\underline{C}H_3$-3), 61.38 (C-8´), 59.72 (6 × O$\underline{C}H_3$-6, 7 × O$\underline{C}H_3$-2), 52.33 (C-6^{I}), 50.81 (C-6´), 50.10 (C-2´), 41.07 (C-3´), 29.45 (C-4´), 27.07 (C-5´), 23.34 (C-7´).

Signals tagged with * can be mutually interchanged.

HRMS (ESI): found 1772.8706. For $C_{84}H_{133}N_5O_{35}$ $[M+H]^+$ calculated 1772.8716.

IR (KBr) 2923, 2833, 1679, 1458, 1368, 1195, 1162, 1108, 1033 cm^{-1}.

$[\alpha]_D^{25}$= +152.9° (*c* = 0.43, MeOH).

6^{I}-Deoxy-6^{I}-(4-(((*R*)-quinolin-4-yl(5-vinylquinuclidin-2-yl)methoxy) methyl)-1*H*-1,2,3-triazol-1-yl)-permethyl-β-CD (9b)

The compound **9b** was prepared according to the general procedure (**GP2**) from 6^{I}-azido-6^{I}-deoxy-permethyl-β-CD (**7**, 100 mg, 0.07 mmol), 9-*O*-propargylcinchonidine (**3b**, 29 mg, 0.09 mmol) and copper iodide (3 mg, 0.01 mmol). The product was obtained as white-yellow solid (85 mg, 69% yield). TLC: R_F = 0.38 (eluent B).

^{1}H NMR (MeOH-d$_4$): δ (ppm): 8.88 – 8.86 (m, 1H, H-2´´), 8.33 – 8.31 (m, 1H, H-8´´), 8.12 – 8.10 (m, 1H, H-5´´), 7.97 (s, 1H, H-14´), 7.83 – 7.80 (m, 1H, H-6´´), 7.73 – 7.71 (m, 1H, H-3´´), 7.71 – 7.69 (m, 1H, H-7´´), 5.80 – 5.75 (m, 1H, H-10´), 5.37 – 5.36 (m, 1H, H-1^{I}), 5.27 – 5.13 (m, 6H, H-1), 5.12 – 5.10 (m, 1H, H-6a^{I}), 4.98 – 4.96 (m, 1H, H-11a´), 4.93 – 4.91 (m, 1H, H-11b´), 4.78 – 4.74 (m, 1H, H-6b^{I}), 4.58 (s, 2H, H-12´), 4.09 – 4.06 (m, 1H, H-5^{I}), 3.90 – 3.72 (m, 18H, H-5, H-6), 3.68 – 3.60 (m, 21H, OC$\underline{H}_3$-3), 3.59 – 3.40 (m, 13H, H-3, H-4), 3.52

– 3.45 (m, 21H, OC$\underline{H}_3$-2), 3.44 – 3.42 (m, 1H, H-6b´), 3.39 – 3.36 (m, 1H, H-4^{I}), 3.39 – 3.24 (m, 18H, OC$\underline{H}_3$-6), 3.22 – 3.20 (m, 1H, H-8´), 3.20 – 3.07 (m, 7H, H-2), 3.07 – 3.05 (m, 1H, H-2a´), 2.71 – 2.67 (m, 1H, H-6a´), 2.61 – 2.58 (m, 1H, H-2b´), 2.36 – 2.33 (m, 1H, H-3´), 1.82 – 1.77 (m, 1H, H-4´), 1.80 – 1.77 (m, 1H, H-5a´), 1.60 – 1.59 (m, 1H, H-5b´), 1.32 – 1.29 (m, 2H, H-7´).

H-9´ and C-9´ signals are not seen.

^{13}C NMR (MeOH-d$_4$): δ (ppm): 151.00 (C-2´´), 149.22 (C-10´´*), 147.92 (C-4´´*), 145.08 (C-13´), 142.43 (C-10´), 131.00 (C-6´´), 130.35 (C-5´´), 128.48 (C-7´´), 127.90 (C-9´´), 127.38 (C-14´), 124.63 (C-8´´), 120.67 (C-3´´), 115.19 (C-11´), 99.38 (7 × C-1), 83.21 (7 × C-2, 7 × C-4), 80.37 (7 × C-3), 72.57 (6 × C-5, 6 × C-6), 71.85 (C-5^{I}), 63.52 (C-12´), 61.88 (7 × O$\underline{C}H_3$-3), 61.71 (C-8´), 59.25 (6 × O$\underline{C}H_3$-6, 7 × O$\underline{C}H_3$-2), 57.43 (C-2´), 52.38 (C-6^{I}), 43.91 (C-6´), 40.81 (C-3´), 29.02 (C-4´), 28.22 (C-5´), 23.46 (C-7´).

Signals tagged with * can be mutually interchanged.

HRMS (ESI): found 1772.8671. For $C_{84}H_{133}N_5O_{35}$ $[M+H]^+$ calculated 1772.8716.

IR (KBr) 2944, 2836, 1679, 1455, 1329, 1201, 1159, 1105, 1033 cm^{-1}.

$[\alpha]_D^{25}$= +96.9° (*c* = 0.18, MeOH).

6^{I}-Deoxy-6^{I}-(4-(((*R*)-1-(6-methoxyquinolin-4-yl(5-vinylquinuclidin-2-yl)methoxy) methyl)-1*H*-1,2,3-triazol-1-yl)-permethyl-β-CD (9c)

The compound **9c** was prepared according to the general procedure (**GP2**) from 6^{I}-azido-6^{I}-deoxy-permethyl-β-CD (**7**, 100 mg, 0.07 mmol), 9-*O*-propargyl-quinine (**3c**, 32 mg, 0.09 mmol) and copper iodide (3 mg, 0.01 mmol). The product was obtained as white-yellow solid (60 mg, 48% yield). TLC: R_F = 0.38 (eluent B).

^{1}H NMR (MeOH-d$_4$): δ (ppm): 8.72 – 8.71 (m, 1H, H-2´´), 8.01 (s, 1H, H-14´), 8.00 – 7.99 (m, 1H, H-8´´), 7.70 – 7.68 (m, 1H, H-3´´), 7.56 – 7.54 (m, 1H, H-5´´), 7.48 – 7.46 (m, 1H, H-7´´), 5.82 – 5.78 (m, 1H, H-10´), 5.38 (m, 1H, H-1^{I}), 5.28 – 5.10 (m, 6H, H-1), 5.12 – 5.10 (m, 1H, H-6a^{I}), 5.00 – 4.99 (m, 1H, H-11a´), 4.97 – 4.94 (m, 1H, H-11b´), 4.84 – 4.80 (m, 1H, H-6b^{I}), 4.65 – 4.60 (m, 2H, H-12´), 4.10 – 4.07 (m, 1H, H-5^{I}), 4.02 (s, 3H, OC$\underline{H}_3$-6´´), 4.01 – 3.21 (m, 18H, H-5, H-6), 3.65 – 3.58 (m, 21H, OC$\underline{H}_3$-3), 3.55 – 3.45 (m, 21H, OC$\underline{H}_3$-2), 3.35 – 3.25 (m, 18H, OC$\underline{H}_3$-6), 3.64 – 3.62 (m, 1H, H-8´), 3.18 – 3.16 (m, 1H, H-2a´), 3.65 – 3.25 (m, 14H, H-3, H-4), 3.25 – 3.07 (m, 7H, H-2), 2.82 – 2.80 (m, 2H, H-6´), 2.73 – 2.70 (m, 1H, H-2b´), 2.43 – 2.40 (m, 1H, H-3´), 1.86 – 1.85 (m, 1H, H-4´), 1.83 – 1.80 (m, 1H, H-5a´), 1.67 – 1.60 (m, 1H, H-5b´), 1.39 – 1.24 (m, 2H, H-7´).

H-9´signal is not seen.

^{13}C NMR (MeOH-d$_4$): δ (ppm): 160.00 (C-6´´), 148.23 (C-2´´), 145.86 (C-10´´*), 145.20 (C-4´´*), 144.98 (C-13´), 142.08 (C-10´), 131.66 (C-8´´), 128.89 (C-9´´), 127.45 (C-14´), 123.75 (C-7´´), 120.30 (C-3´´), 115.40 (C-11´), 102.52 (C-5´´), 99.38 (7 × C-1), 83.69 (7 × C-2, 7 × C-3, 7 × C-4), 80.05 (C-9´), 72.62 (6 × C-5, 6 × C-6), 71.89 (C-5^{I}), 63.37 (C-12´), 61.84 (7 × O<u>C</u>H$_3$-3), 61.14 (C-8´), 59.10 (7 × O<u>C</u>H$_3$-2, 7 × O<u>C</u>H$_3$-6), 57.20 (C-2´), 56.77 (O<u>C</u>H$_3$-6´´), 52.38 (C-6^{I}), 44.31 (C-6´), 40.49 (C-3´), 28.97 (C-4´), 27.90 (C-5´), 23.11 (C-7´).

Signals tagged with * can be mutually interchanged.

HRMS (ESI): found 1802.8819. For $C_{85}H_{135}N_5O_{36}$ calculated $[M+H]^+$ 1802.8960.

IR (KBr) 2932, 2830, 1715, 1626, 1512, 1467, 1365, 1144, 1036 cm^{-1}.

$[\alpha]_D$= +105.9° (*c* = 0.25, MeOH).

6^{I}-Deoxy-6^{I}-(4-(((*S*)-1-(6-methoxyquinolin-4-yl(5-vinylquinuclidin-2-yl)methoxy) methyl)-1*H*-1,2,3-triazol-1-yl)-permethyl-β-CD (9d)

The compound **9d** was prepared according to the general procedure (**GP2**) from 6^{I}-azido-6^{I}-deoxy-permethyl-β-CD (**7**, 100 mg, 0.07 mmol), 9-*O*-propargylquinidine (**3d**, 32 mg, 0.09 mmol) and copper iodide (3 mg, 0.01 mmol). The product was obtained as white-yellow solid (78 mg, 63% yield). TLC: R_F = 0.38 (eluent B).

^{1}H NMR (MeOH-d$_4$): δ (ppm): 8.74 – 8.72 (m, 1H, H-2´´), 8.00 – 7.99 (m, 1H, H-8´´), 7.97 (s, 1H, H-14´), 7.69 – 7.66 (m, 1H, H-3´´), 7.54 – 7.50 (m, 1H, H-5´´), 7.49 – 7.46 (m, 1H, H-7´´), 6.03 – 5.97 (m, 1H, H-10´), 5.39 – 5.37 (m, 1H, H-1^{I}), 5.25 – 5.10 (m, 6H, H-1), 5.06 – 5.04 (m, 1H, H-6a^{I}), 5.03 – 5.01 (m, 2H, H-11´), 4.85 – 4.83 (m, 1H, H-6b^{I}), 4.59 (s, 2H, H-12´), 4.15 – 4.11 (m, 1H, H-5^{I}), 3.99 (s, 3H, OC<u>*H*</u>$_3$-6´´), 3.90 – 3.35 (m, 18H, H-5, H-6), 3.67 – 3.53 (m, 21H, OC<u>*H*</u>$_3$-3), 3.58 – 3.48 (m, 21H, OC<u>*H*</u>$_3$-2), 3.62 – 3.45 (m, 14H, H-3, H-4), 3.45 – 3.27 (m, 18H, OC<u>*H*</u>$_3$-6), 3.23 – 3.05 (m, 7H, H-2), 3.13 – 3.11 (m, 1H, H-8´), 2.89 – 2.87 (m, 2H, H-6´), 2.86 – 2.84 (m, 1H, H-2a´), 2.77 – 2.75 (m, 1H, H-2b´), 2.34 – 2.31 (m, 1H, H-3´), 2.16 – 2.12 (m, 1H, H-7a´), 1.74 – 1.72 (m, 1H, H-4´), 1.62 – 1.54 (m, 2H, H-5´), 1.35 – 1.27 (m, 1H, H-7b´).

H-9´signal is not seen.

^{13}C NMR (MeOH-d$_4$): δ (ppm): 159.82 (C-6´´), 148.44 (C-2´´), 146.45 (C-10´´*), 145.14 (C-4´´*), 145.06 (C-13´), 141.69 (C-10´), 131.59 (C-8´´), 128.94 (C-9´´), 127.42 (C-14´), 123.61 (C-7´´), 120.66 (C-3´´), 115.39 (C-11´), 102.56 (C-5´´), 99.39 (7 × C-1), 81.52 (C-9´), 83. 47 (7 × C-2, 7 × C-3, 7 × C-4), 72.51 (6 × C-5, 6 × C-6), 71.80 (C-5^{I}), 63.41 (C-12´), 61.91 (7 × O<u>C</u>H$_3$-3), 60.89 (C-8´), 59.25 (7 × O<u>C</u>H$_3$-2, 6 × O<u>C</u>H$_3$-6), 56.46 (O<u>C</u>H$_3$-6´´), 52.33 (C-6^{I}), 50.85 (C-2´), 50.26 (C-6´), 41.12 (C-3´), 29.50 (C-4´), 27.12 (C-5´), 23.67 (C-7´).

Signals tagged with * can be mutually interchanged.

HRMS (ESI): found 1802.8839. For $C_{85}H_{135}N_5O_{36}$ $[M+H]^+$ calculated 1802.8960.

IR (KBr) 2929, 2836, 1619, 1461, 1365, 1228, 1135, 1072, 1042 cm^{-1}.

$[\alpha]_D^{25}$= +146.9° (*c* = 0.48, MeOH).

Preparation of disubstituted α-CD derivatives

$6^{I,IV}$-Dideoxy-$6^{I,IV}$-bis((4-(((*S*)-(6-methoxyquinolin-4-yl)(5-vinylquinuclidin-2-yl)methoxy)methyl)-1*H*-1,2,3-triazol-1-yl)-α-CD (11)

The starting material $6^{I,IV}$-diazido-$6^{I,IV}$-dideoxy-α-CD (**10**, 25 mg, 0.025 mmol), prepared according to a combination of the published procedures [5,6], was dissolved in (10 min-sonicated under Ar atmosphere) H_2O (0.2 mL). Then, 9-*O*-propargyl-quinine (**3c**, 12 mg, 0.03 mmol) was dissolved in (10 min-sonicated under Ar atmosphere) THF (0.2 mL) and added to the CD derivative. Copper iodide (0.013 mmol), suspended in (10 min-sonicated under Ar atmosphere) H_2O (0.1 mL), was added and the reaction mixture was stirred at 50 °C for 16 h. The conversion to product was monitored by TLC in eluent A. After full conversion to product, the reaction mixture was slowly added to stirring acetone (75 mL) resulting in white-yellow precipitate. The solid was recovered by filtration, washed with acetone (3 × 20 mL) and dried to constant weight in a vacuum drying box in the presence of P_2O_5 and KOH. The crude product was purified by column chromatography (50 g of silica gel) with ACN/H_2O/NH_3 12:5:1 as mobile phase. Eye-visible blue precipitation at the top of the column was observed as copper cations interact with ammonia. The purified product was dried at 50 °C in high vacuum and obtained as white-yellow product (33 mg, 76% yield). TLC: R_F = 0.60 (eluent A).

^{1}H NMR (DMSO-d$_6$): δ (ppm): 8.75 – 8.73 (m, 2H, H-2´´), 8.22 (s, 2H, H-14´), 7.94 – 7.95 (m, 2H, H-8´´), 7.83 – 7.81 (m, 2H, H-3´´), 7.65 – 7.63 (m, 2H, H-5´´), 7.43 – 7.41 (m, 2H, H-7´´), 5.78 – 5.76 (m, 2H, H-10´), 5.62 – 5.36 (m, 12H, OH-2, OH-3), 5.01 – 4.99 (m, 2H, H-11a´), 4.94 – 4.92 (m, 2H, H-11b´), 4.87 – 4.85 (m, 2H, H-6a^{I,IV}), 4.80 – 4.72 (m, 6H, H-1), 4.78 – 4.76 (m, 2H, H-6b^{I,IV}), 4.58 – 4.44 (m, 4H, OH-6), 4.52 – 4.50 (m, 2H, H-12a´), 4.40 – 4.38 (m, 2H, H-12b´), 4.00 – 3.98 (m, 2H, H-5^{I,IV}), 3.95 (s, 6H, OC$\underline{H}_3$-6´´), 3.88 – 3.21 (m, 33H, H-2, H-3, H-4, H-5, H-6), 3.38 – 3.39 (m, 2H, 8´´), 2.44 – 2.42 (m, 2H, H-3´), 1.80 – 1.78 (m, 2H, H-4´).

H-2´, H-6´, H-5´, H-7´, H-9´, C-5´, C-7´, C-9´signals are not fully seen.

^{13}C NMR (DMSO-d$_6$): δ (ppm): 157.56 (2 × C-6´´), 147.44 (2 × C-2´´), 144.20 (2 × C-4´´*), 144.02 (2 × C-10´´*), 142.94 (2 × C-13´), 140.35 (2 × C-10´), 131.21 (2 × C-8´´), 126.95 (2 × C-9´´), 125.50 (2 × C-14´), 121.76 (2 × C-7´´), 118.77 (2 × C-3´´), 115.22 (2 × C-11´), 102.18 (2 × C-5´´), 101.90 (6 × C-1), 83.06 (6 × C-4), 71.52 (6 × C-2, 6 × C-3, 4 × C-5), 69.07 (2 × C-5^{I,IV}), 61.93 (2 × C-12´), 61.58 (4 × C-6), 59.02 (2 × C-8´), 56.18 (2 × O<u>C</u>H$_3$-6´´), 54.10 (2 × C-2´), 50.46 (2 × C-6^{I,IV}), 42.65 (2 × C-6´), 37.79 (2 × C-3´), 26.73 (2 × C-4´).

Signals tagged with * can be mutually interchanged.

HRMS (ESI): found 874.3652. For $C_{82}H_{110}N_{10}O_{32}$ calculated $[M+2H]^{2+}$ 874.3717.

IR (KBr) 3183, 3120, 2935, 1710, 1650, 1401, 1258, 1111, 1048 cm^{-1}.

$[\alpha]_D^{25}$= +40.7° (c = 0.17, DMSO).

Catalytic activity of CD derivatives

Decarboxylative asymmetric allylic amination (AAA)

The starting material, MBH carbamate **12**, prepared according to the published procedure [7], was dissolved in ACN/H_2O (for CD catalysts **4a–d**, **5a–d** and **11**) or toluene (for CD catalysts **8a–d**, **9a–d**) to achieve a 0.4 M solution. Then, CD catalysts (**4a–d**, **5a–d**, **11**, **8a–d**, **9a–d**) were added and the mixture stirred at 40 °C for 168 h. Conversion to product was monitored by TLC in hexane/EtOAc 6:1. The reaction mixture was evaporated and the crude product purified by column chromatography (hexane/EtOAc 10:1). NMR and MS spectra of product **9** are in accordance with the literature [7]. Enantiomeric excess was measured on an IB column (heptane/propan-2-ol 99:1), flow 1 mL/min, λ = 205 nm, T = 25 °C). Retention times of products: t_R = 9.0–10.5 min (major enantiomer), t_R = 9.5–11.2 min (minor enantiomer).

Starting material: **Methyl 2-(phenyl((phenylcarbamoyl)oxy)methyl)acrylate (12)**

^{1}H NMR (CDCl$_3$): δ (ppm): 7.46 – 7.24 (m, 9H), 7.06 (t, J = 7.4 Hz, 1H), 6.80 (s, 1H), 6.71 (s, 1H), 6.44 (s, 1H), 5.91 (s, 1H), 3.72 (s, 3H).

Spectra are in accordance with the literature [7].

MS (ESI): found: 334.1. For $C_{18}H_{17}NO_4$ calculated $[M+Na]^+$ 334.1.

Product: **Methyl (*R*)-2-(phenyl(phenylamino)methyl)acrylate (13)**

^{1}H NMR ($CDCl_3$): δ (ppm): 7.41 – 7.27 (m, 5H), 7.22 – 7.10 (m, 2H), 6.77 (t, *J* = 7.3 Hz, H), 6.64 (d, *J* = 7.8 Hz, 2H), 6.41 (s, 1H), 6.03 (s, 1H), 5.43 (s, 1H), 4.17 (*b*s, 1H) 3.71 (s, 3H).

Spectra are in accordance with the literature [8].

MS (ESI): found 290.1. For $C_{17}H_{17}NO_2$ calculated $[M+Na]^+$ 290.1.

References

(1) Kacprzak, K. M. Chemistry and Biology of Cinchona Alkaloids. In *Natural Products*; Ramawat, K. G., Mérillon, J.-M., Eds.; Springer Berlin Heidelberg: Berlin, Heidelberg, 2013; pp 605–641.
(2) Tang, W.; Ng, S.-C. *Nat. Protoc.* **2008**, *3* (4), 691–697.
(3) Bauer, M.; Fajolles, C.; Charitat, T.; Wacklin, H.; Daillant, J. *J. Phys. Chem. B* **2011**, *115* (51), 15263–15270.
(4) Al Temimi, A. H. K.; Boltje, T. J.; Zollinger, D.; Rutjes, F. P. J. T.; Feiters, M. C. *Bioconjug. Chem.* **2017**, *28* (8), 2160–2166.
(5) Guieu, S.; Sollogoub, M. *J. Org. Chem.* **2008**, *73* (7), 2819–2828.
(6) Kumprecht, L.; Buděšínský, M.; Vondrášek, J.; Vymětal, J.; Černý, J.; Císařová, I.; Brynda, J.; Herzig, V.; Koutník, P.; Závada, J.; et al. *J. Org. Chem.* **2009**, *74* (3), 1082–1092.
(7) Dočekal, V.; Šimek, M.; Dračínský, M.; Veselý, J. *Chem. - Eur. J.* **2018**, *24* (51), 13441–13445.
(8) Liu, K.; Han, X.; Wang, Z.; Wang, K.; Ding, J. *J. Am. Chem. Soc.* **2015**, *137*, 15346–15349.

^{1}H and ^{13}C NMR spectra of prepared CD derivatives

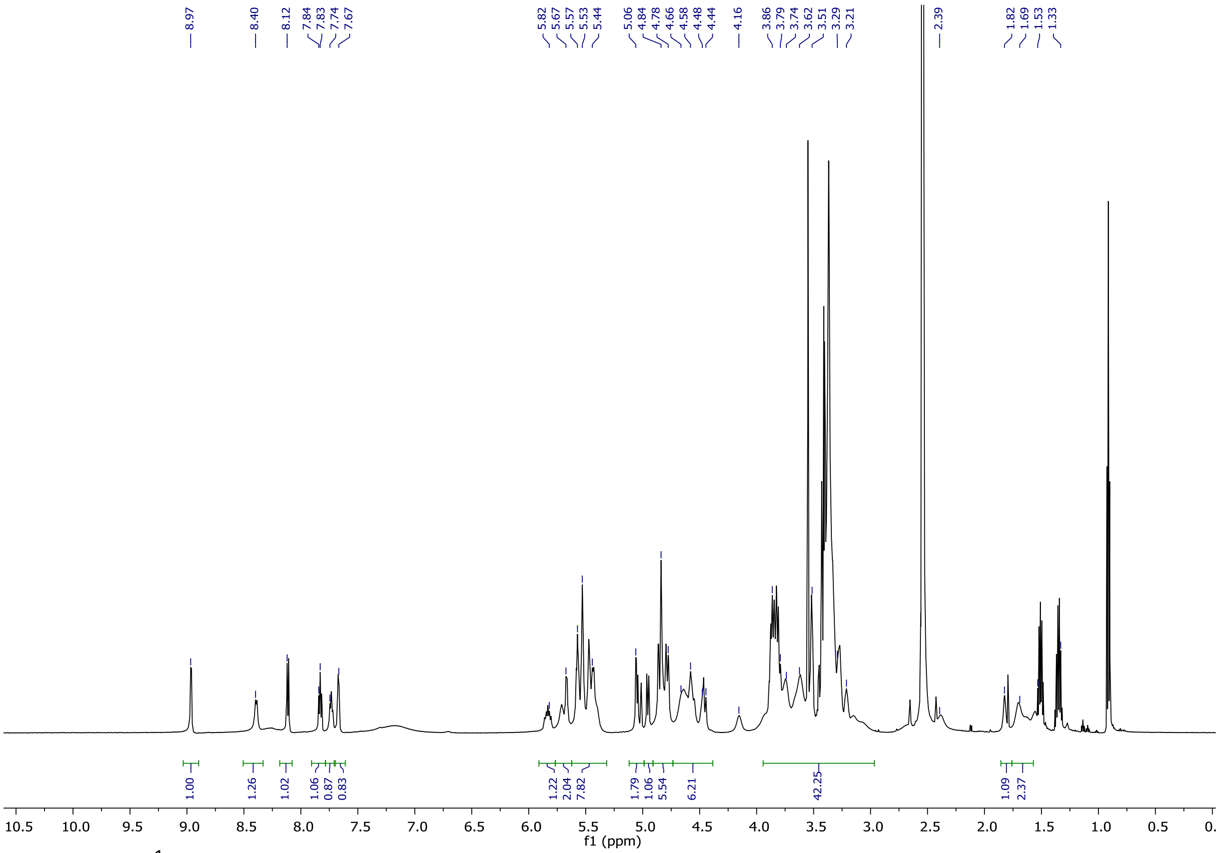

Figure S2: ^{1}H NMR spectrum of compound **4a**.

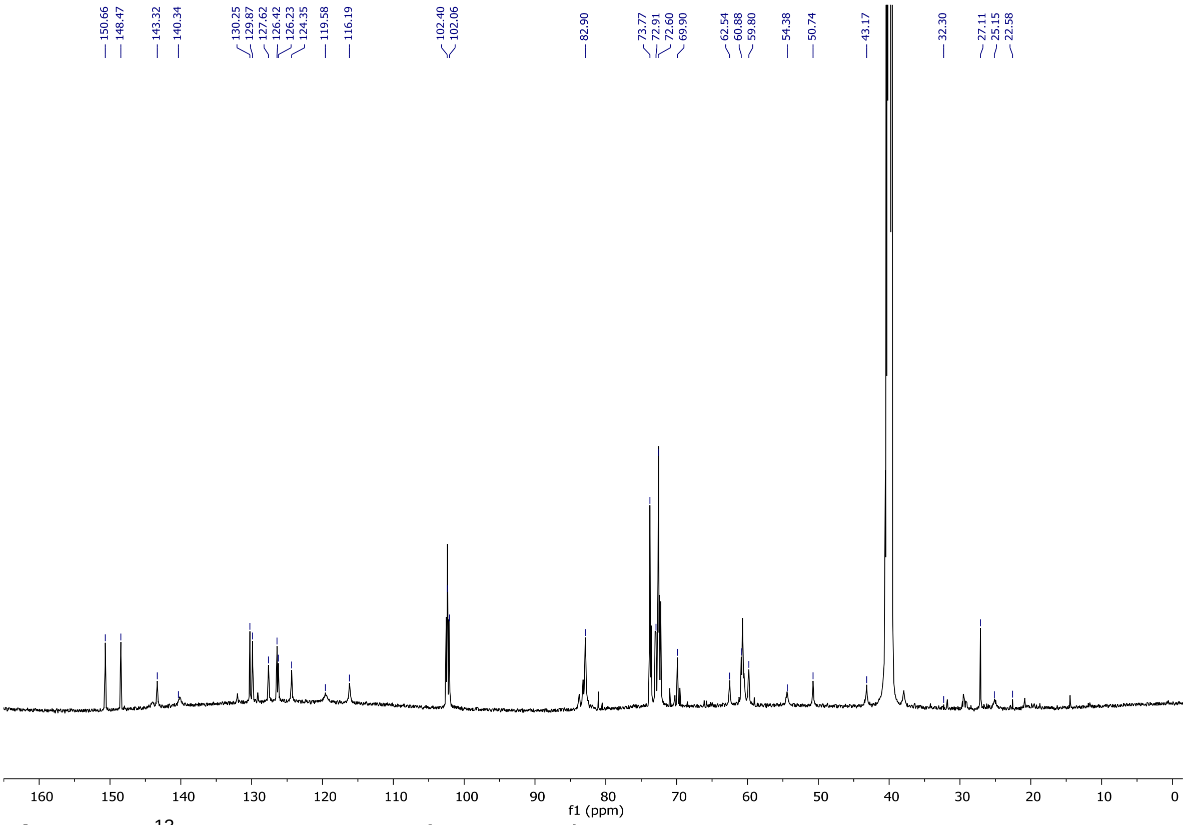

Figure S3: ^{13}C NMR spectrum of compound **4a**.

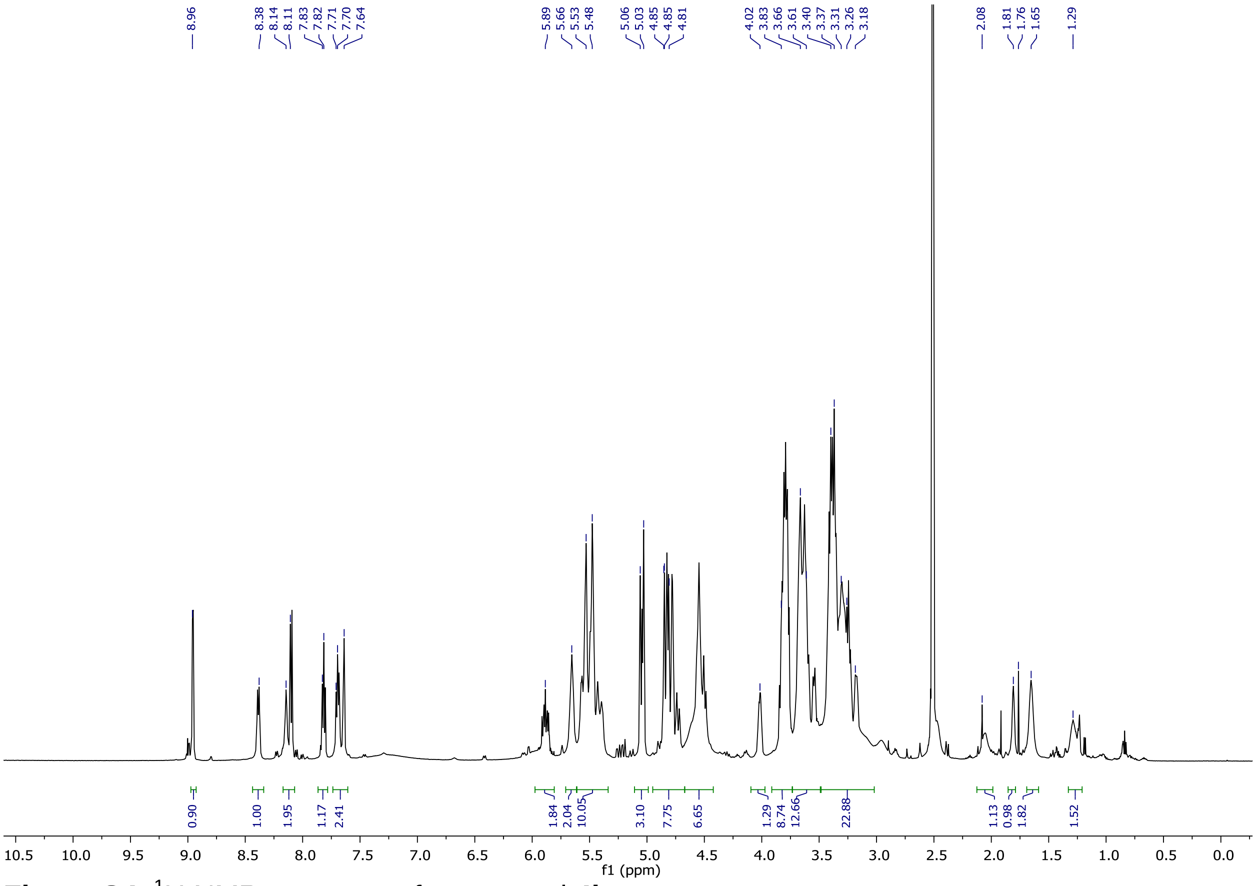

Figure S4: ^{1}H NMR spectrum of compound **4b**.

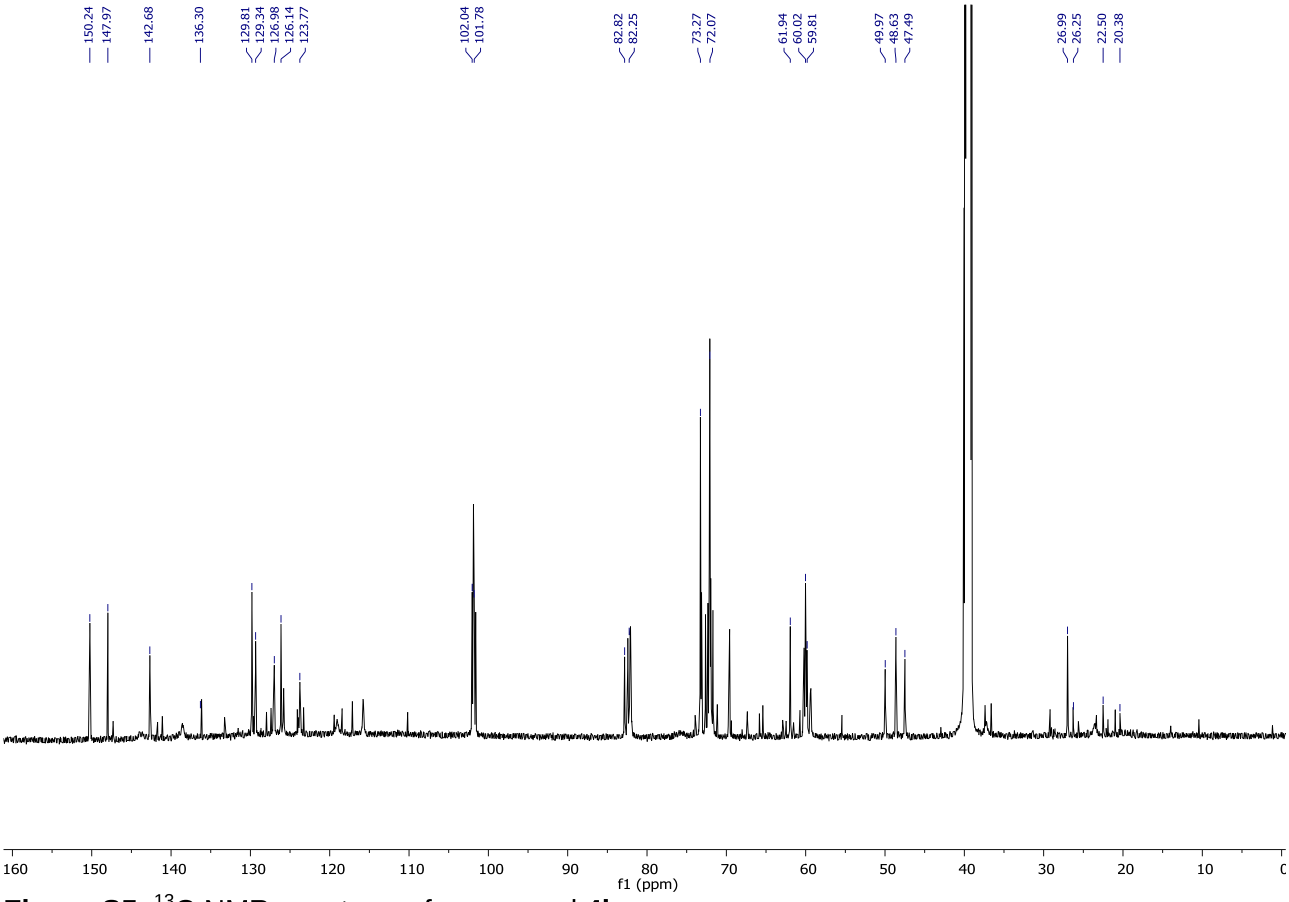

Figure S5: ^{13}C NMR spectrum of compound **4b.**

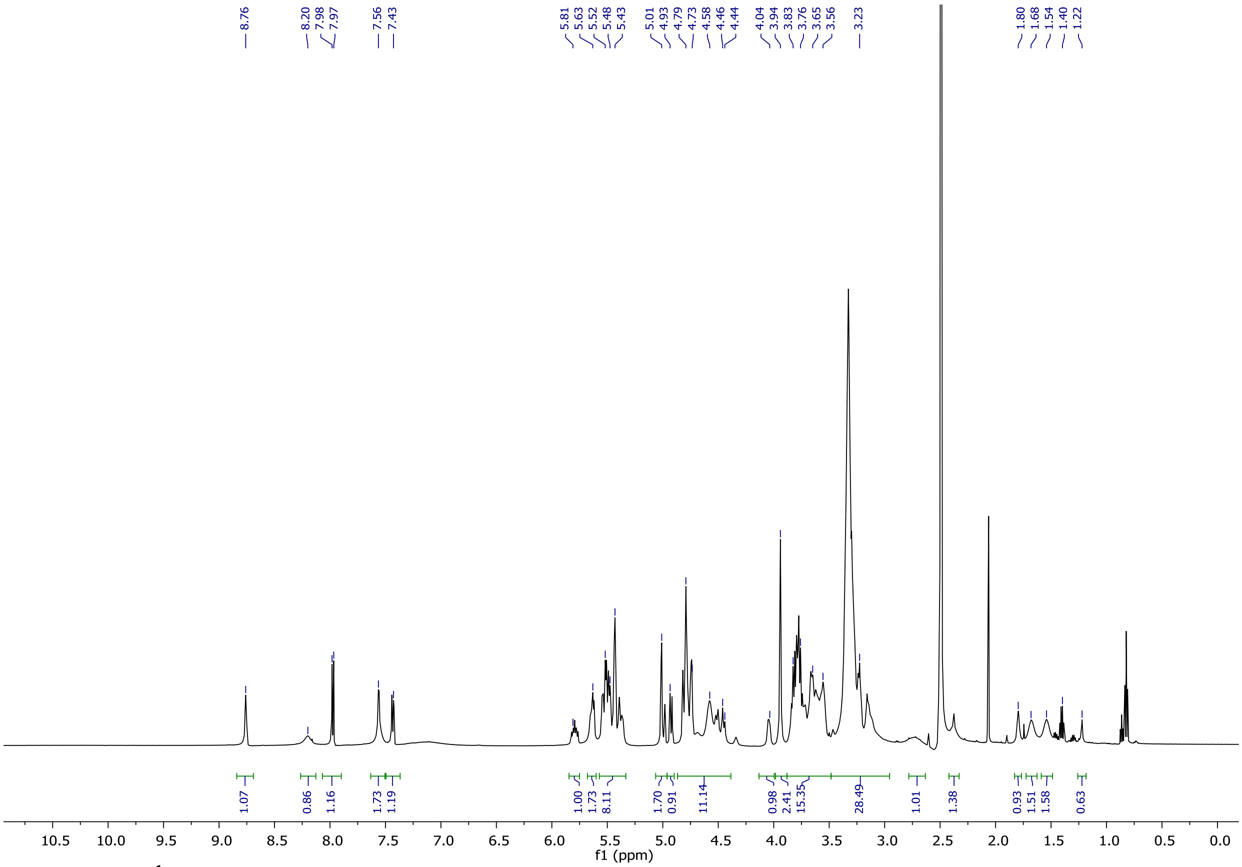

Figure S6: ^{1}H NMR spectrum of compound **4c**.

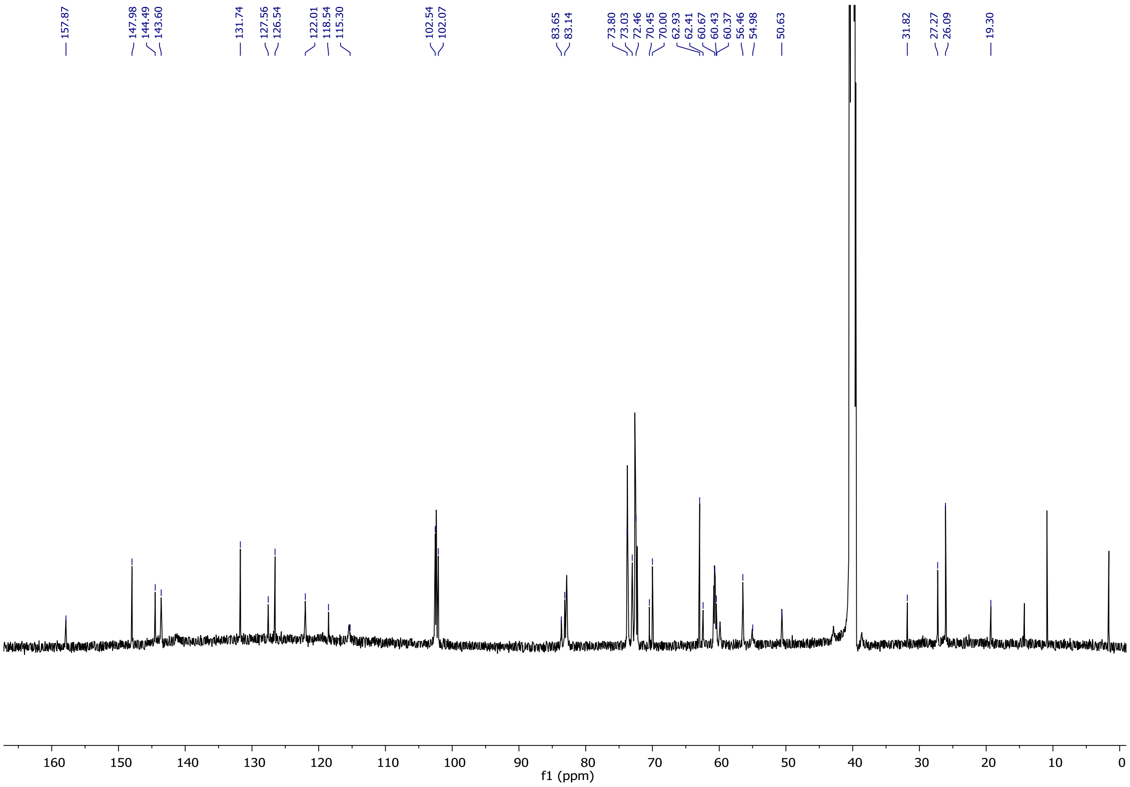

Figure S7: ^{13}C NMR spectrum of compound **4c.**

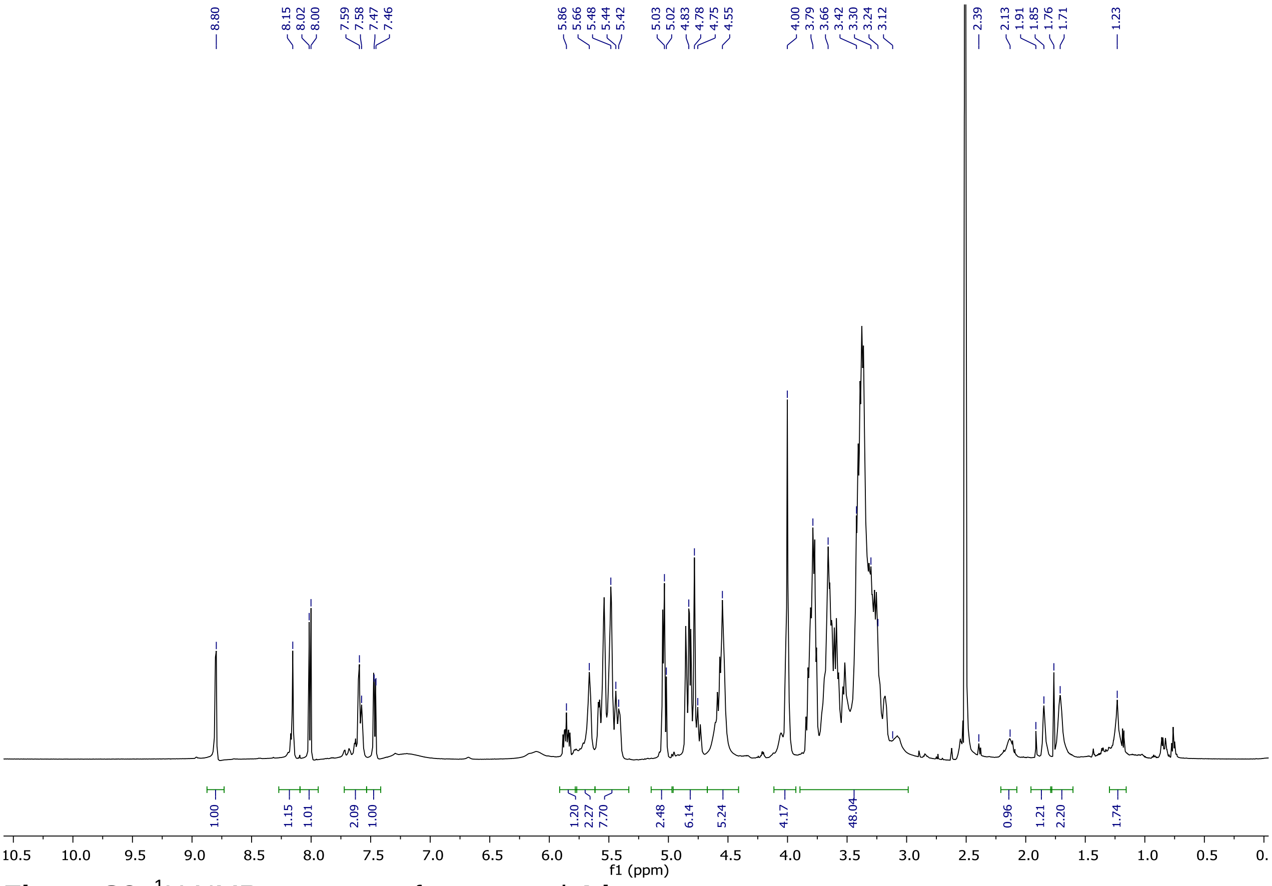

Figure S8: ^{1}H NMR spectrum of compound **4d**.

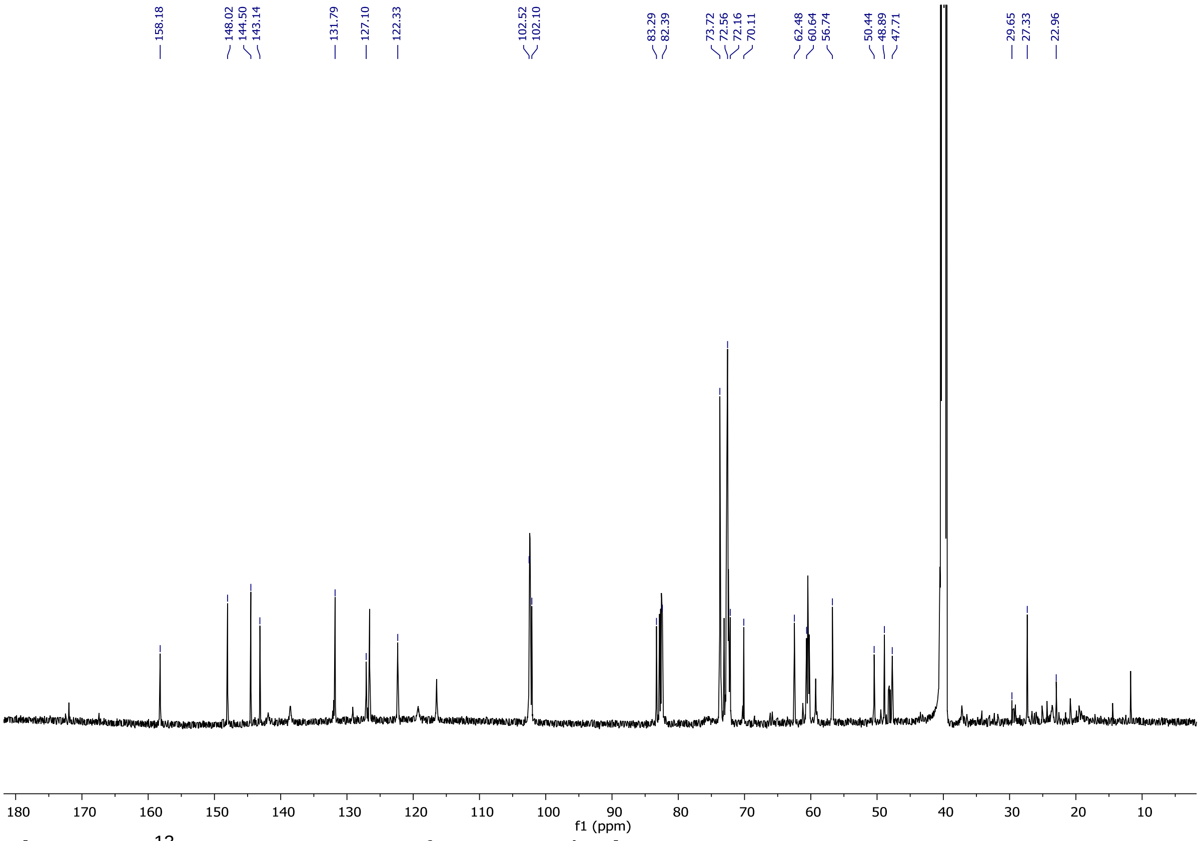

Figure S9: ^{13}C NMR spectrum of compound **4d**.

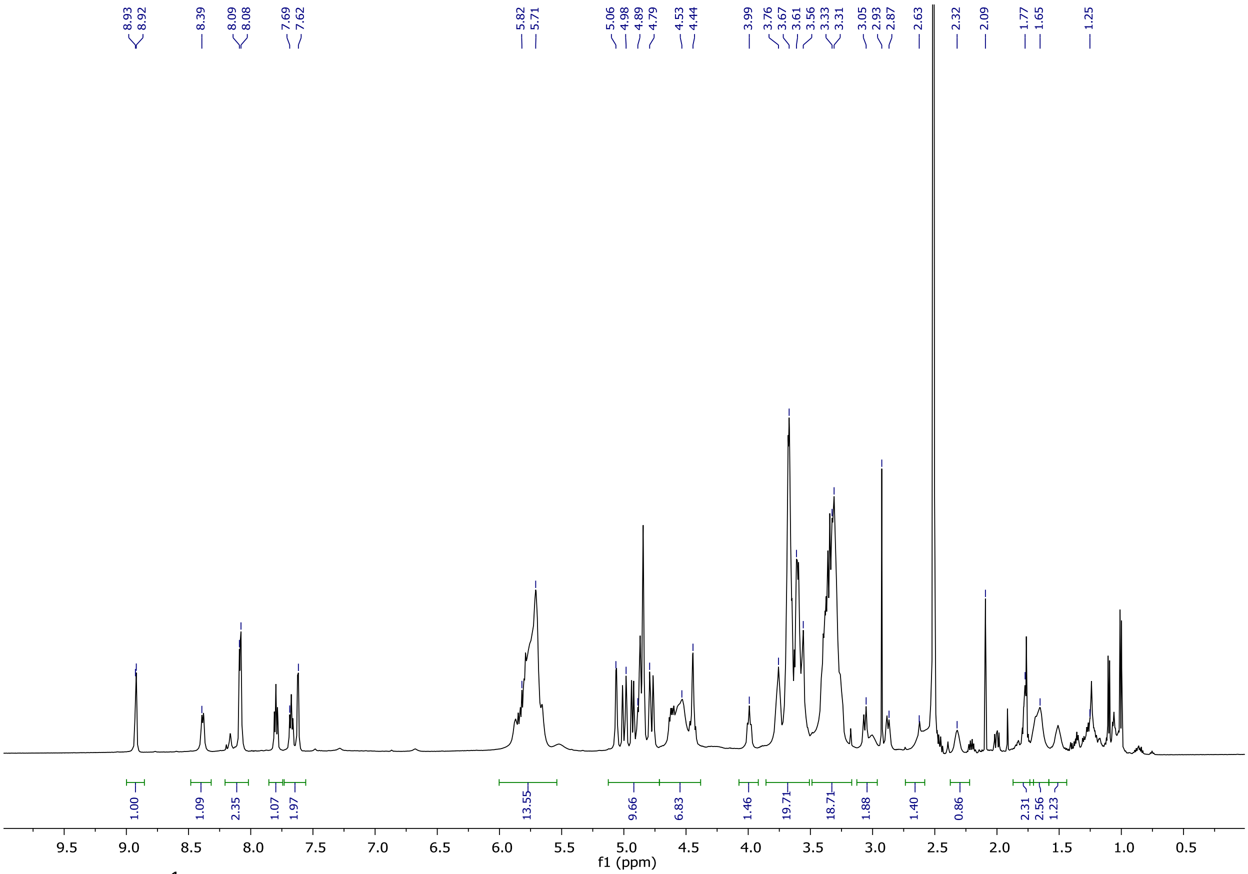

Figure S10: ^{1}H NMR spectrum of compound **5a**.

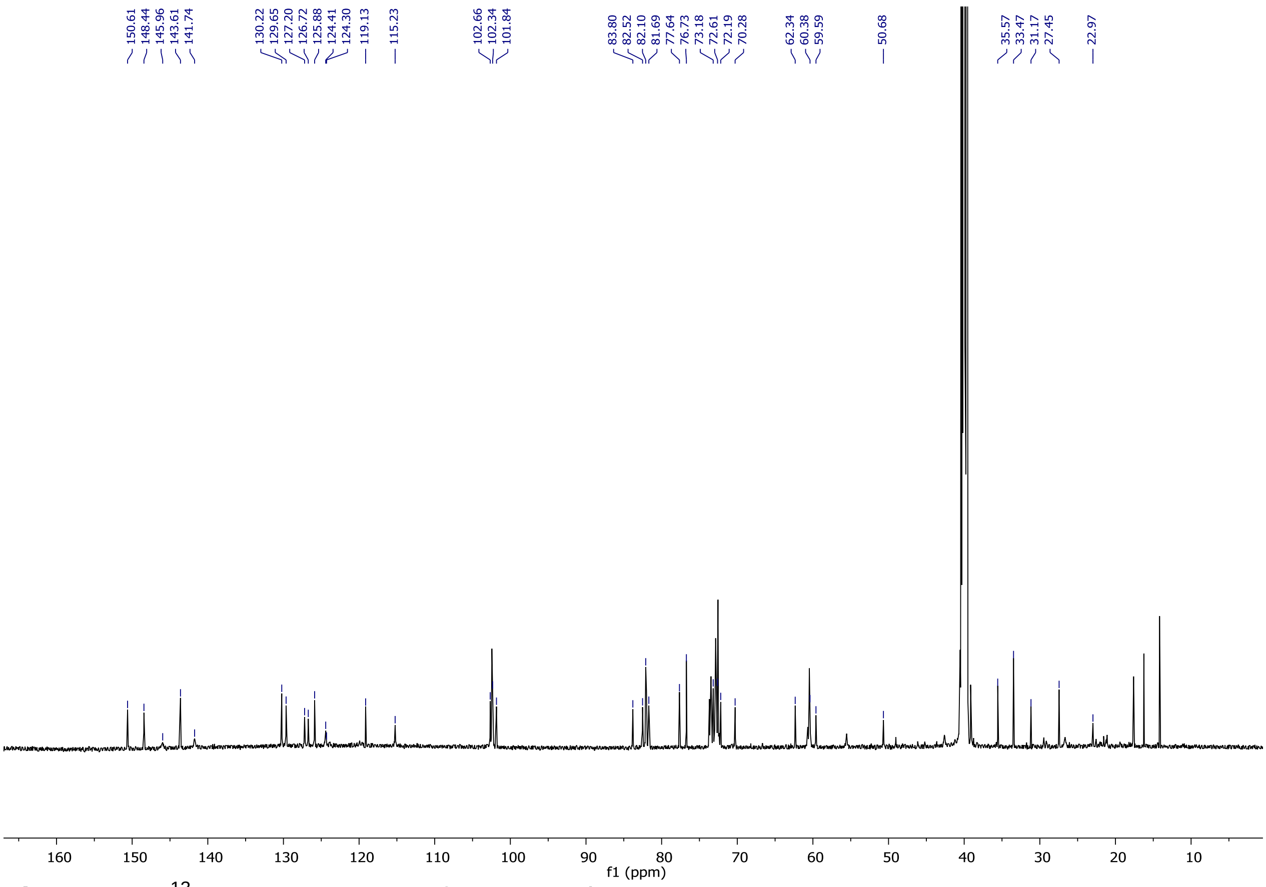

Figure S11: ^{13}C NMR spectrum of compound **5a**.

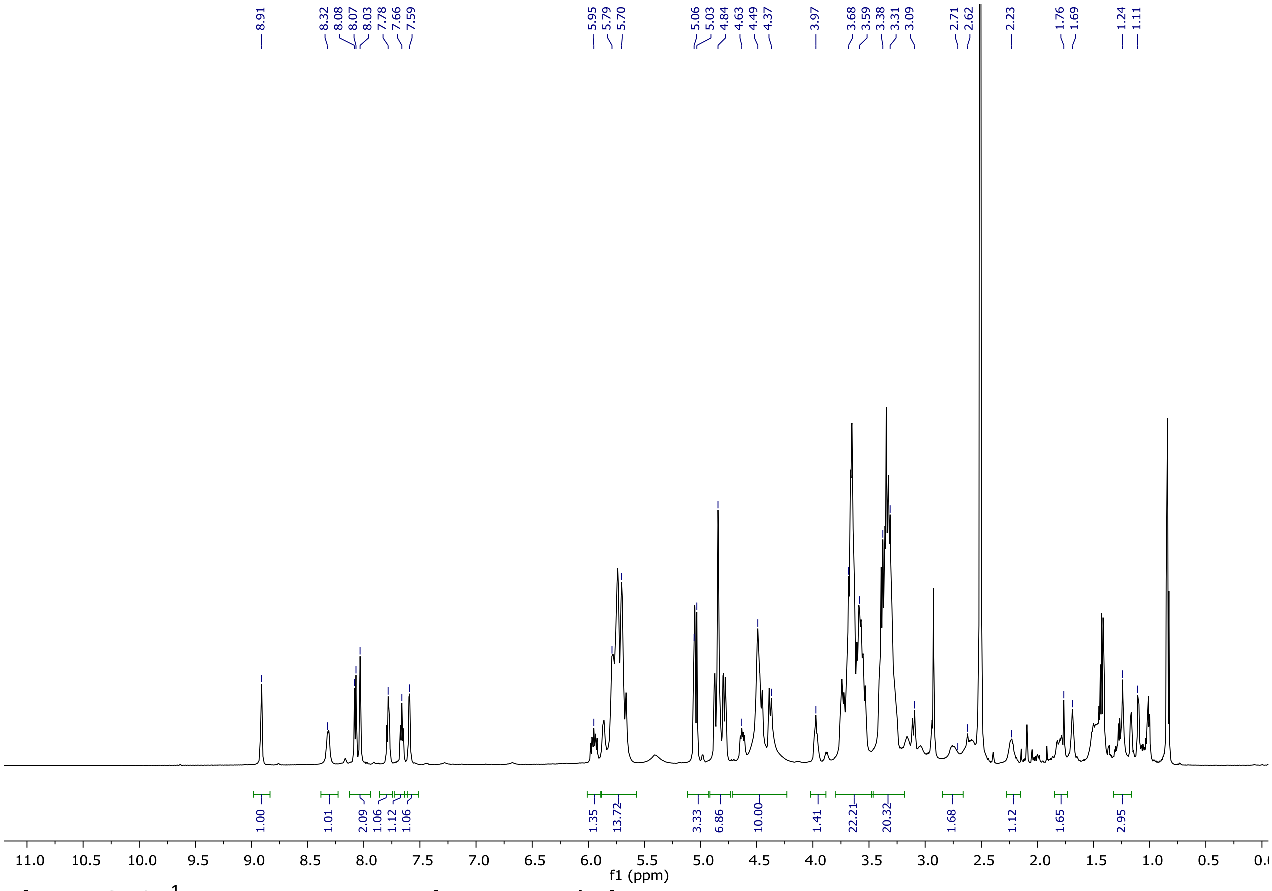

Figure S12: ^{1}H NMR spectrum of compound **5b**.

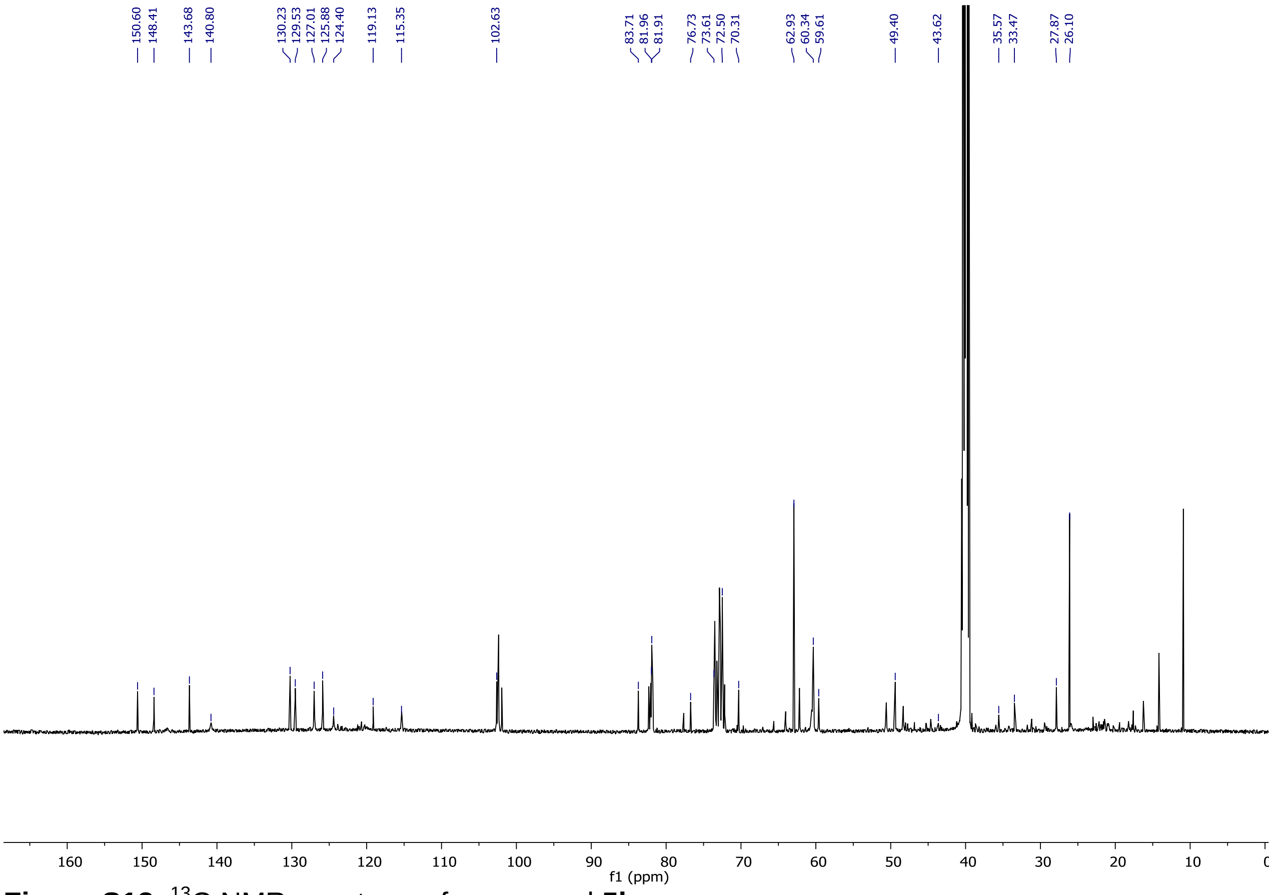

Figure S13: ^{13}C NMR spectrum of compound **5b**.

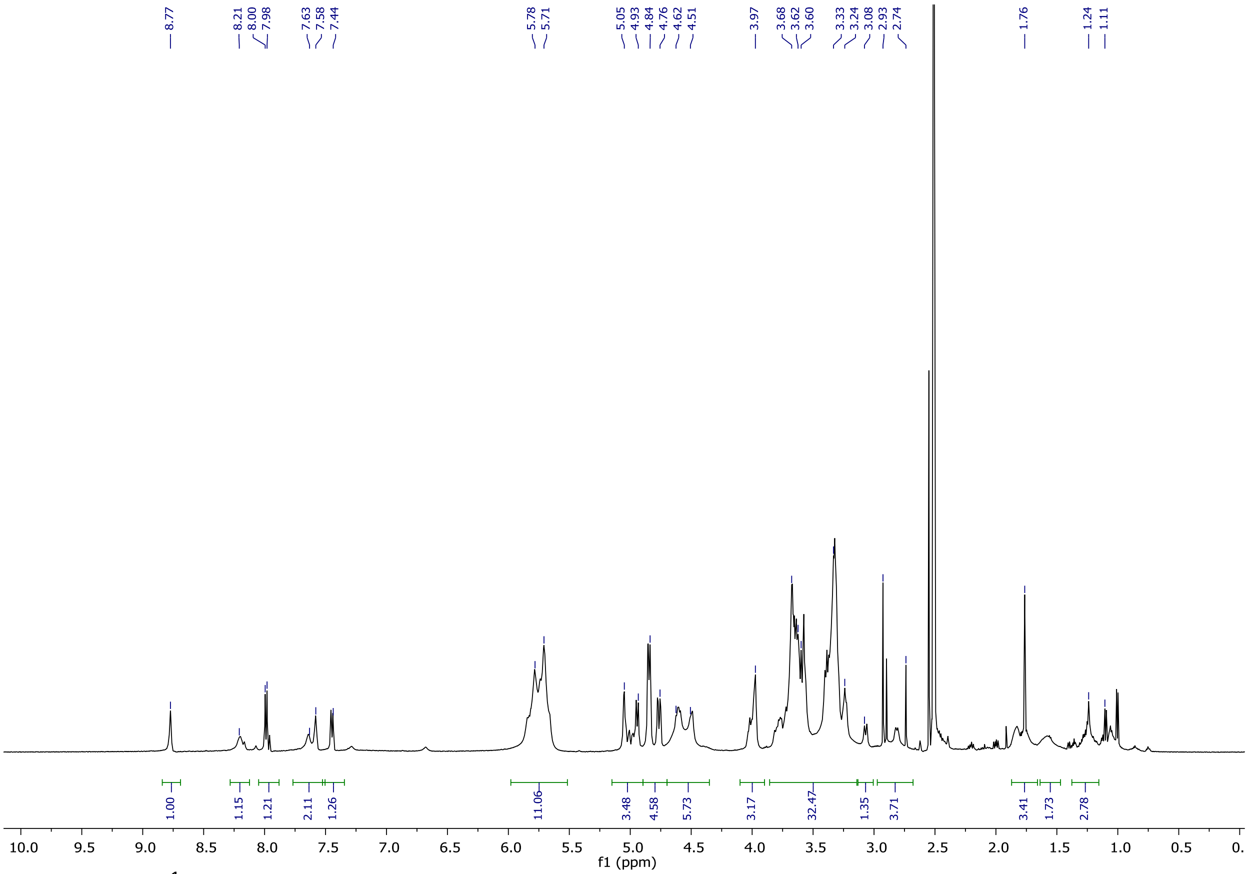

Figure S14: ^{1}H NMR spectrum of compound **5c**.

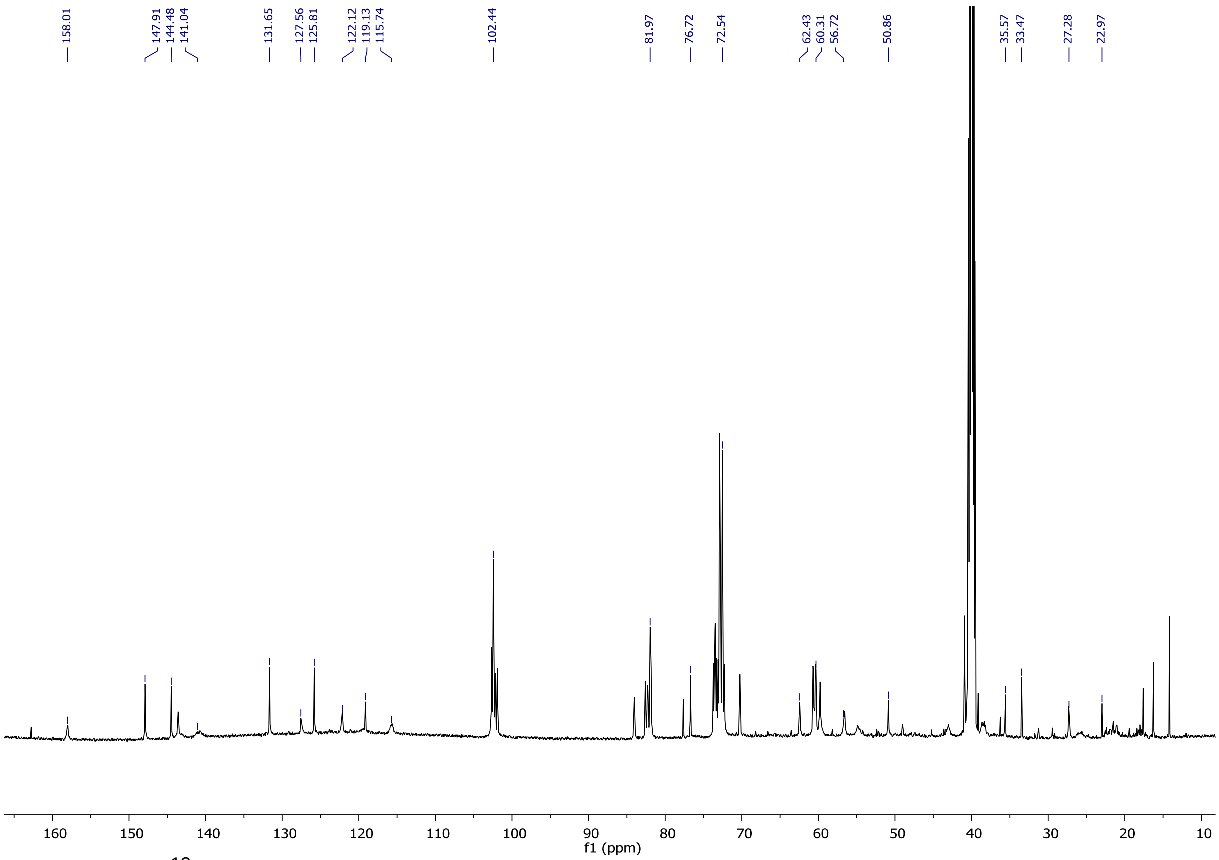

Figure S15: ^{13}C NMR spectrum of compound **5c**.

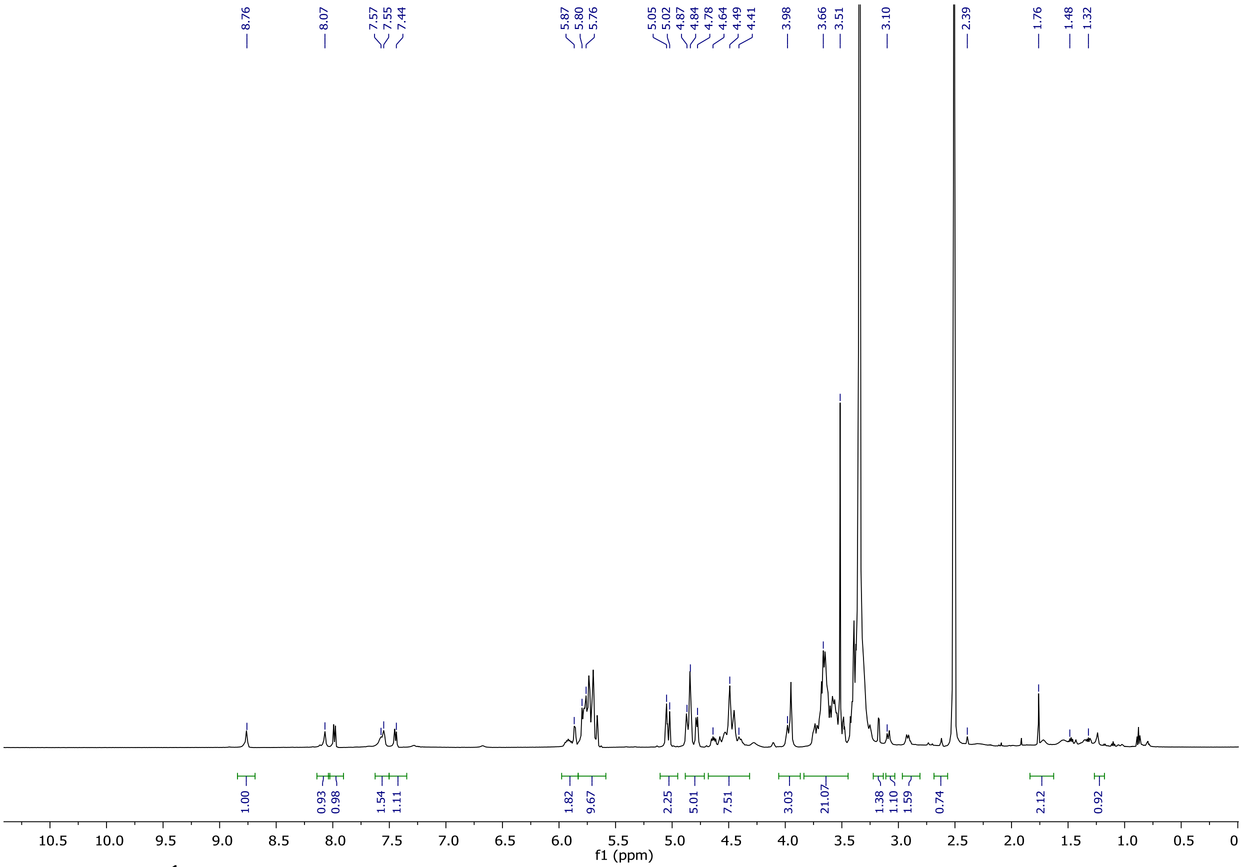

Figure S16: ^{1}H NMR spectrum of compound **5d**.

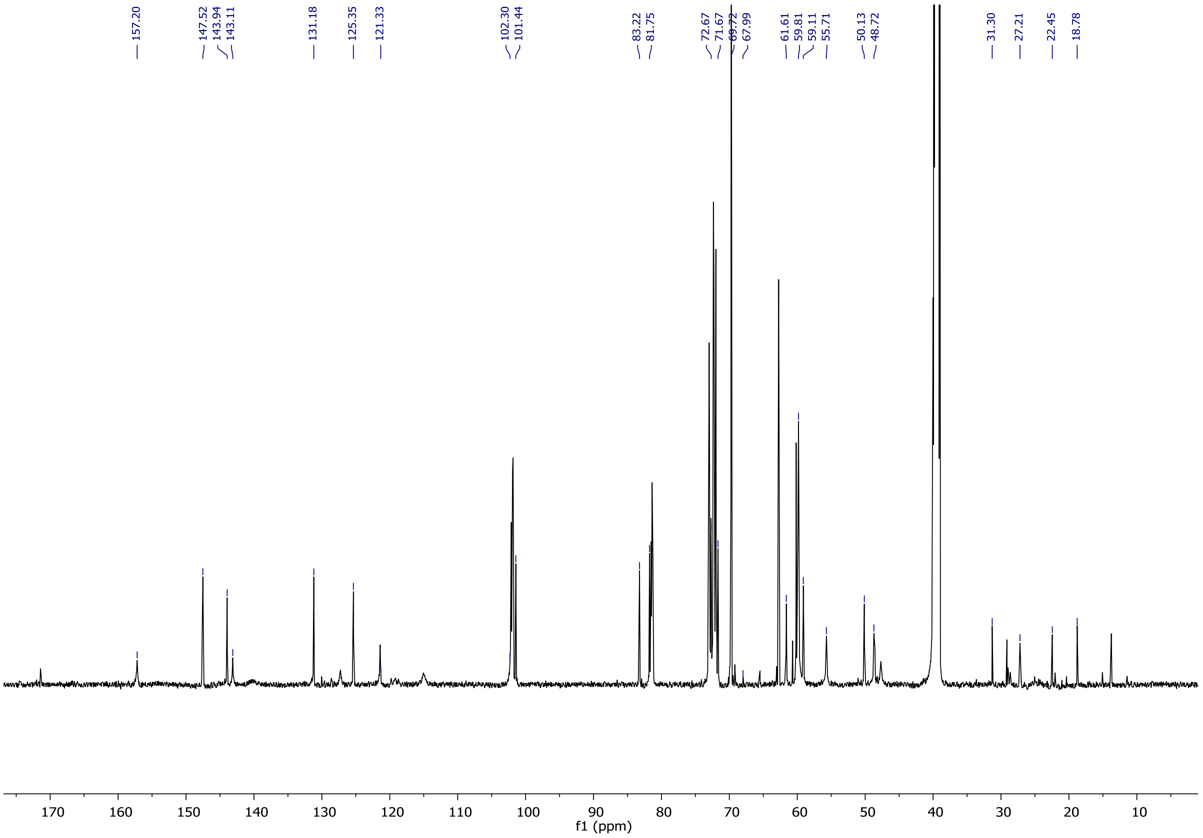

Figure S17: ^{13}C NMR spectrum of compound **5d**.

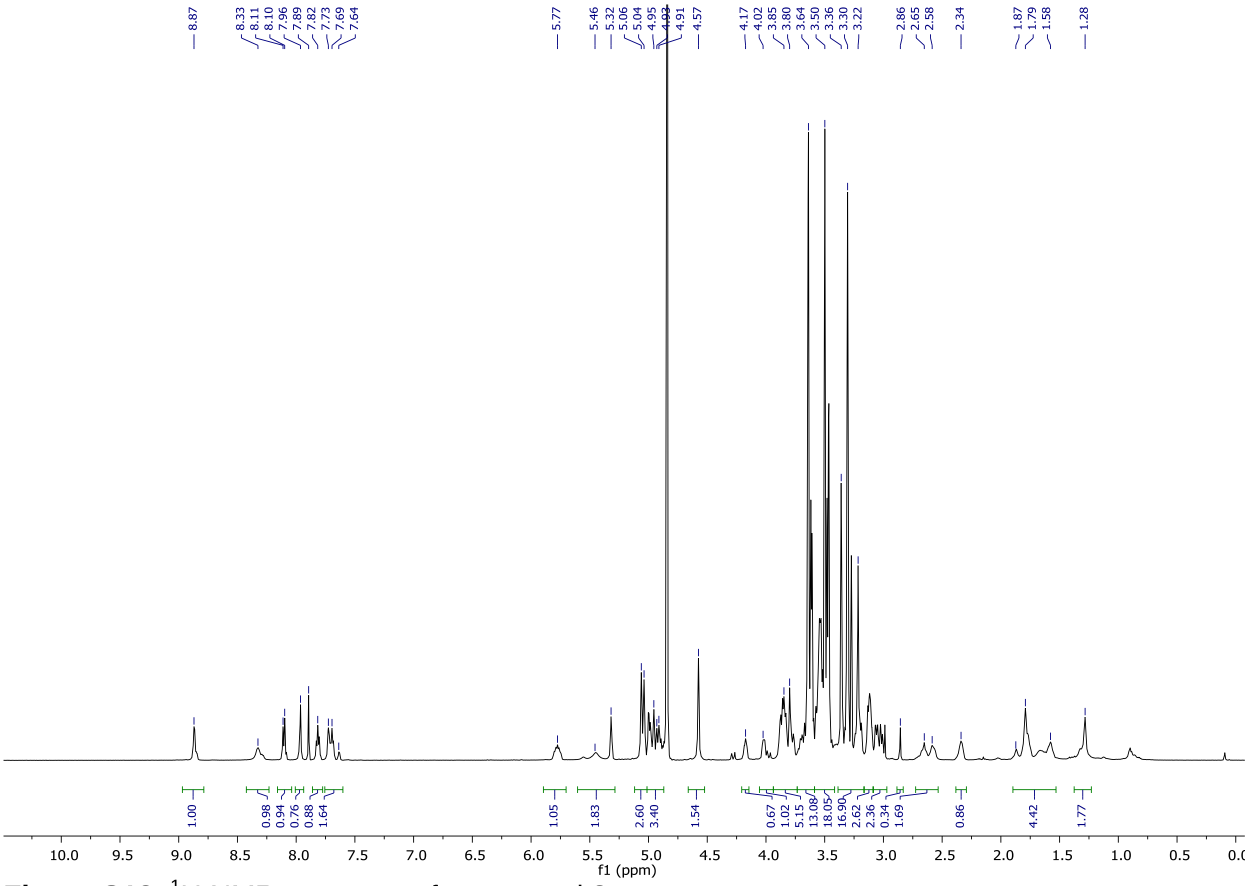

Figure S18: ^{1}H NMR spectrum of compound **8a**.

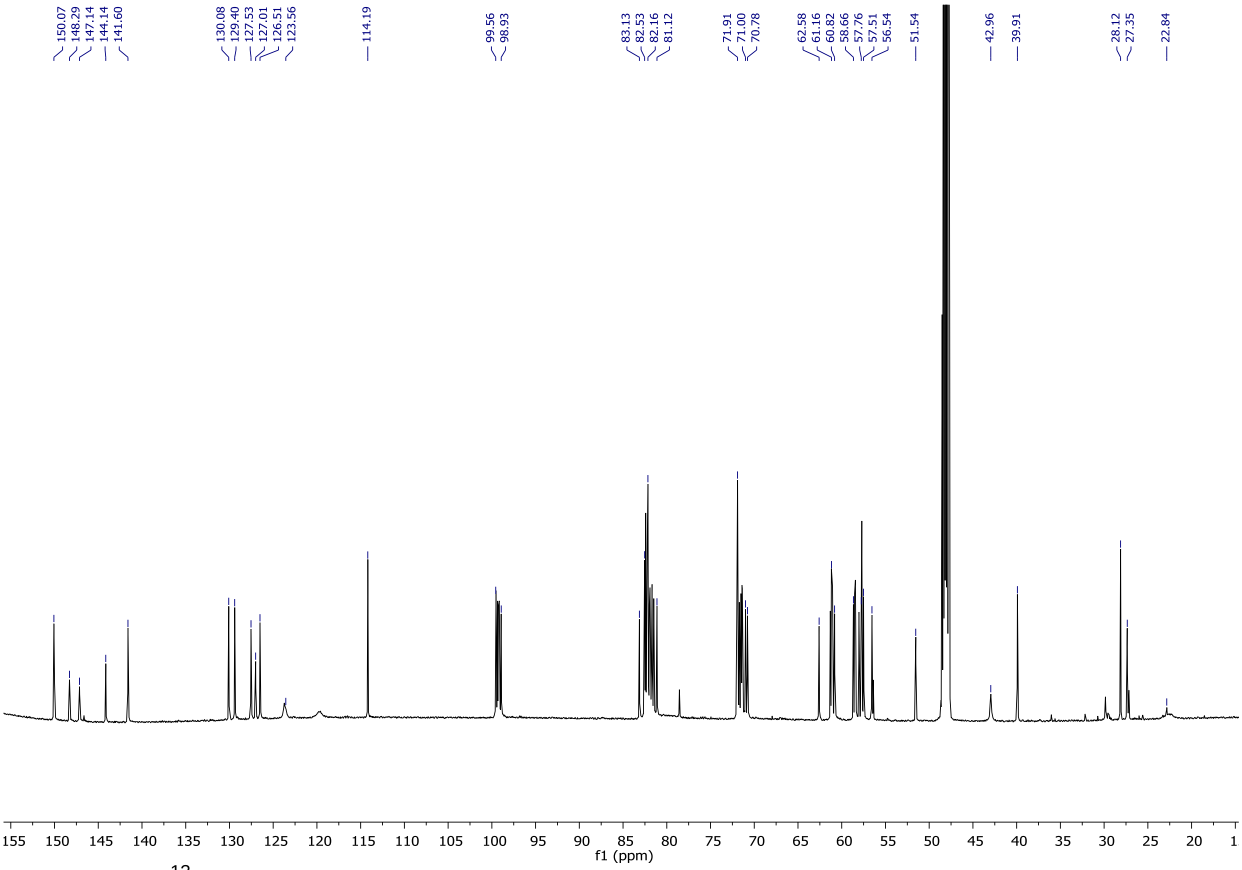

Figure S19: ^{13}C NMR spectrum of compound **8a**.

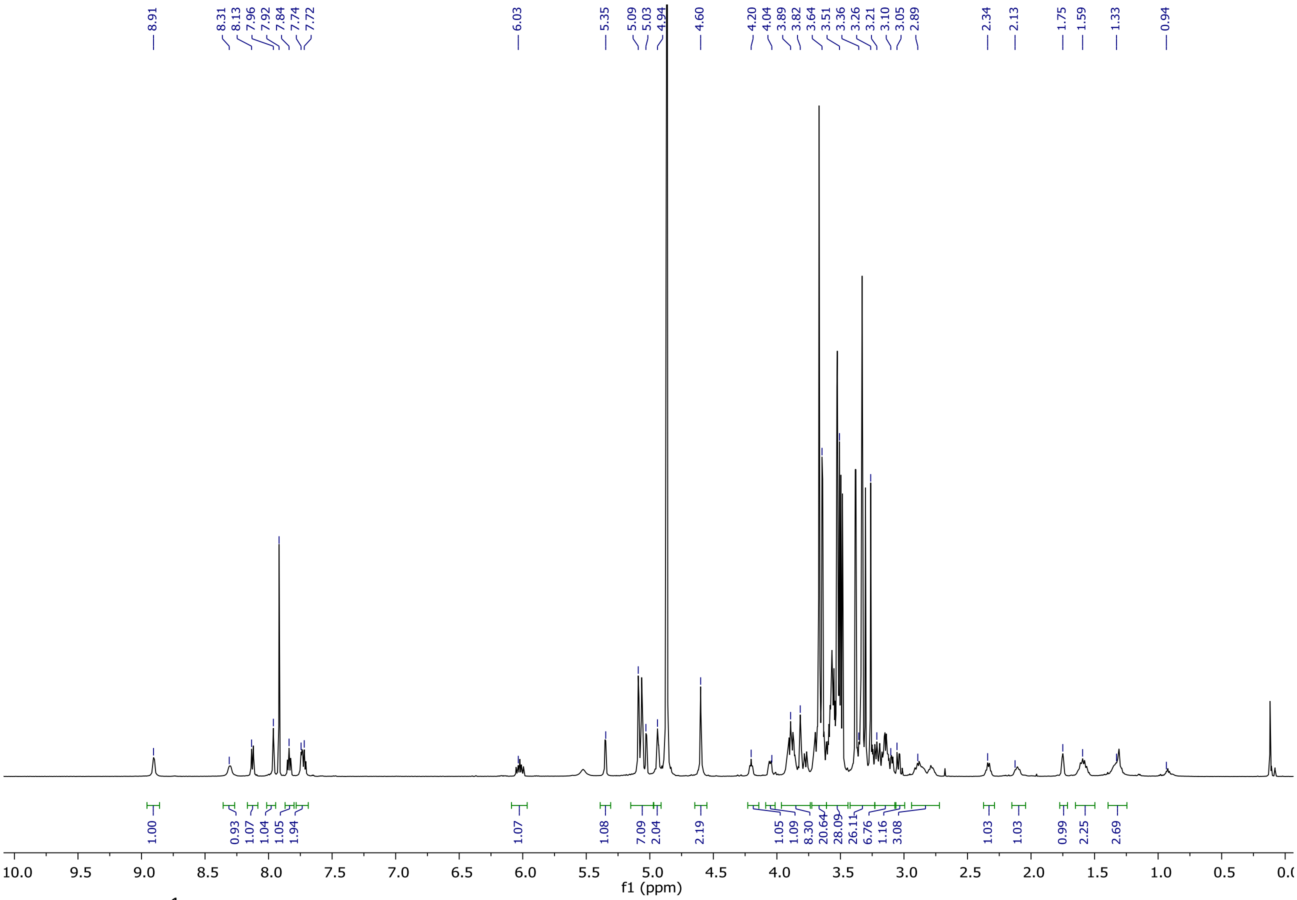

Figure S20: ^{1}H NMR spectrum of compound **8b**.

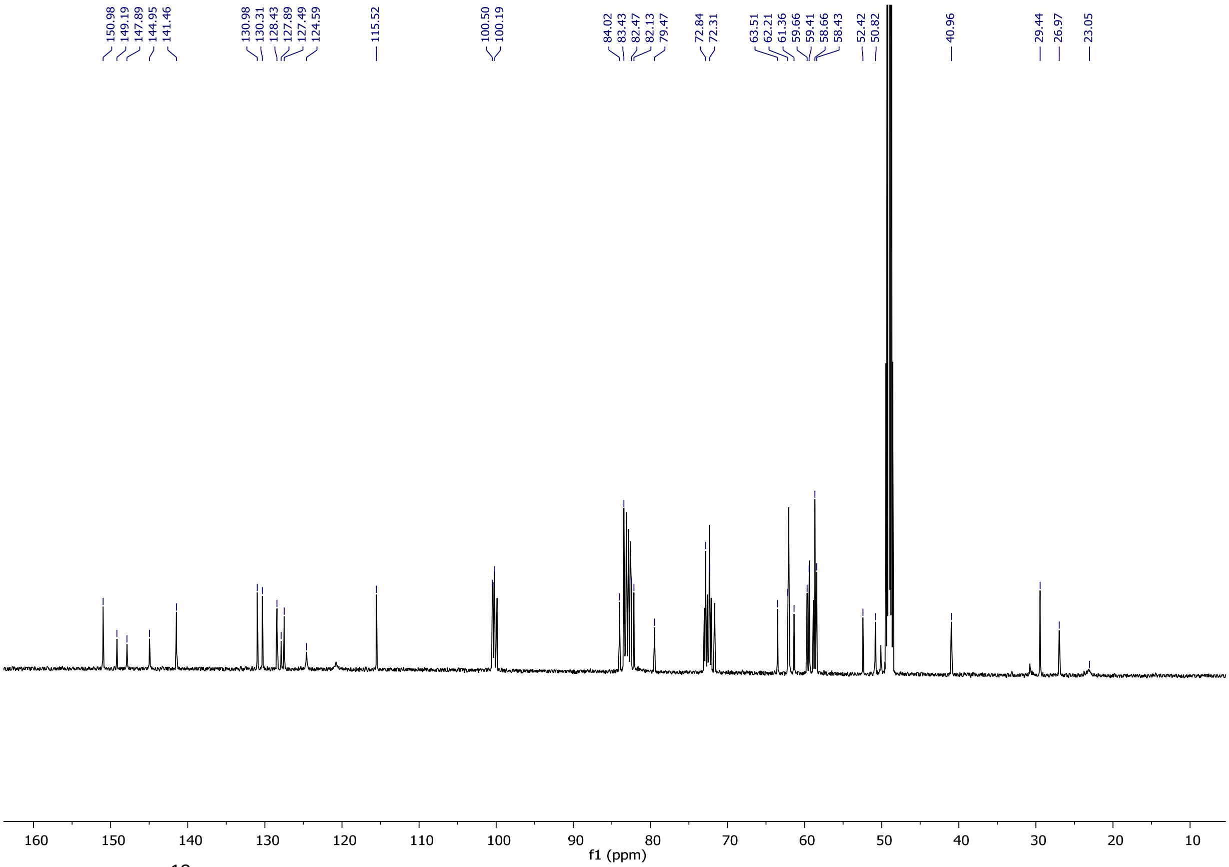

Figure S21: ^{13}C NMR spectrum of compound **8b**.

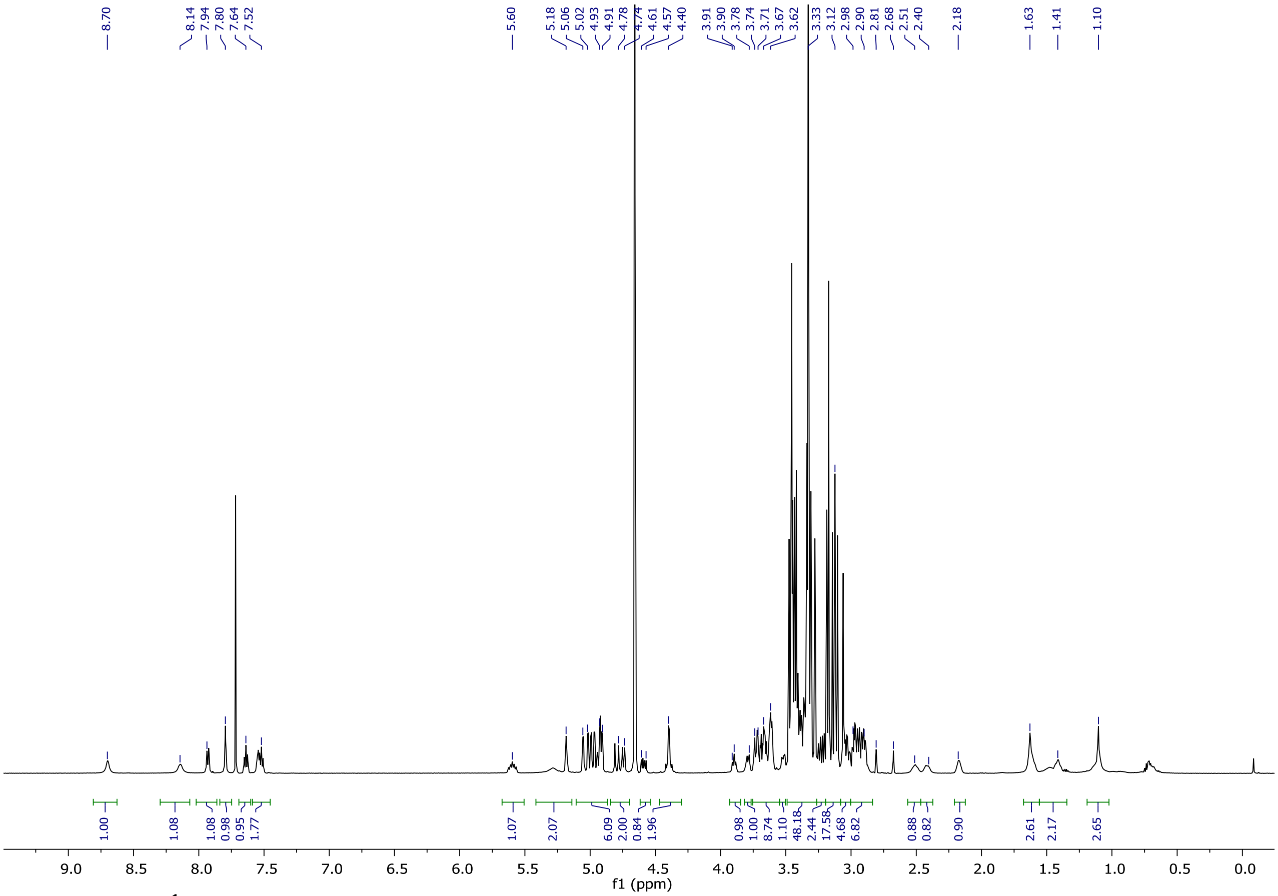

Figure S22: ^{1}H NMR spectrum of compound **8c**.

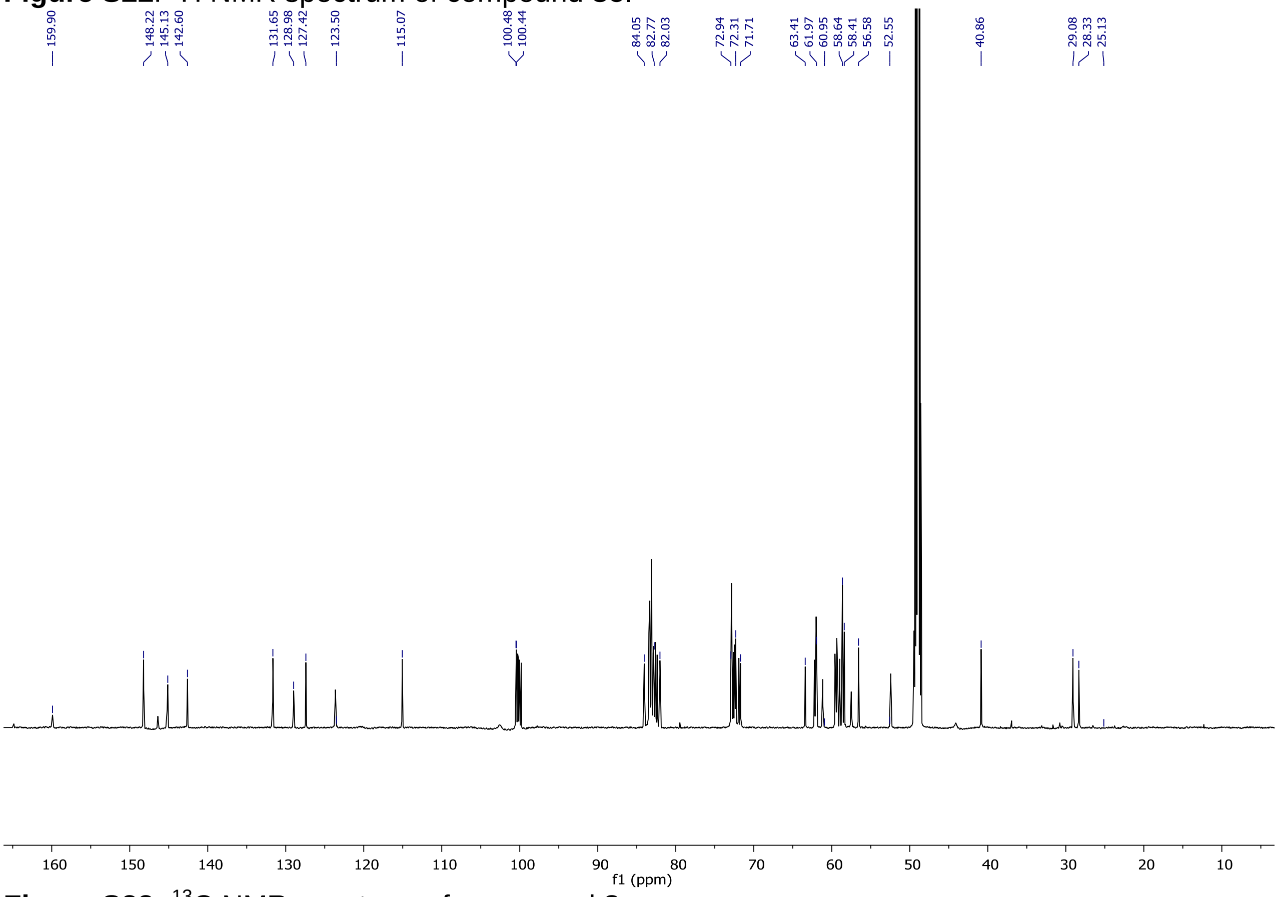

Figure S23: ^{13}C NMR spectrum of compound **8c**.

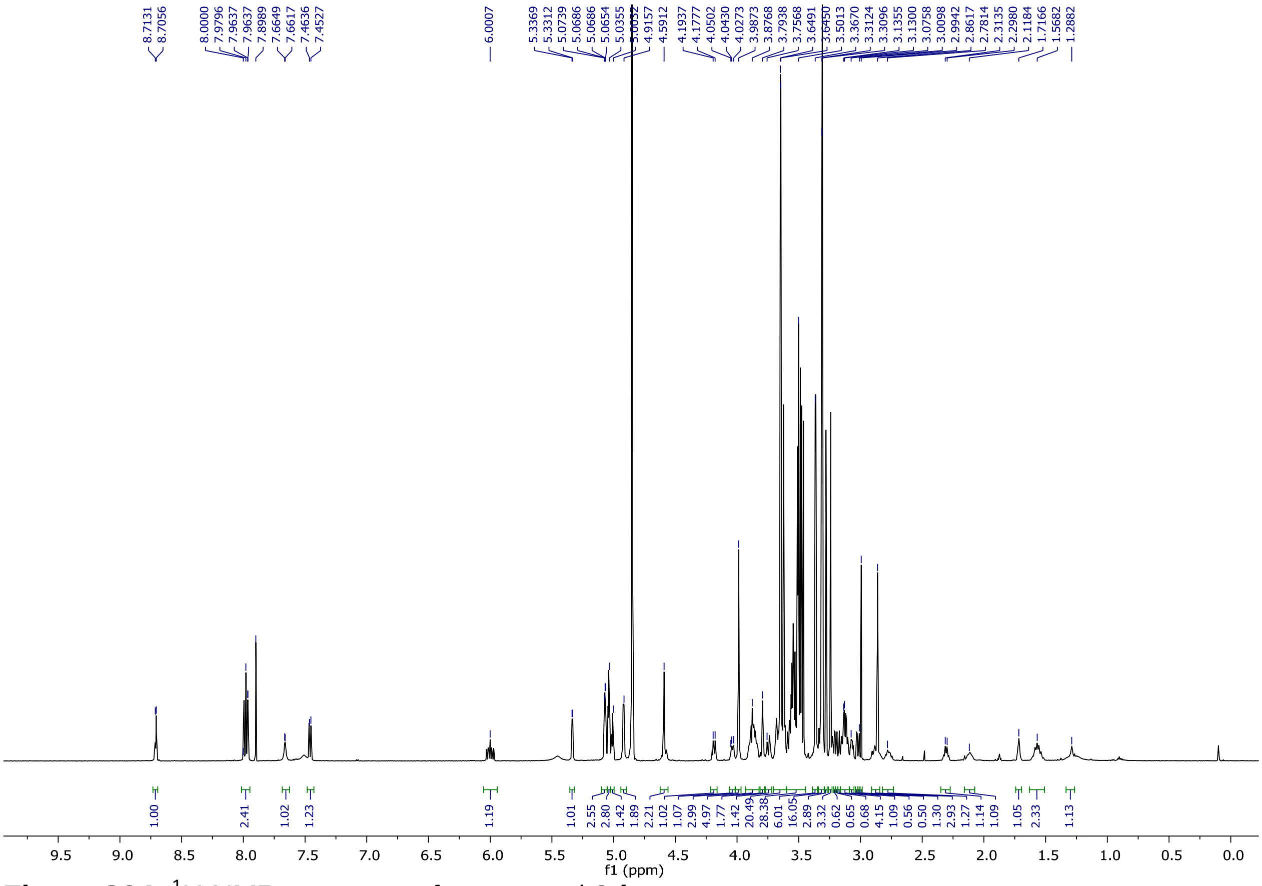

Figure S24: ^{1}H NMR spectrum of compound **8d**.

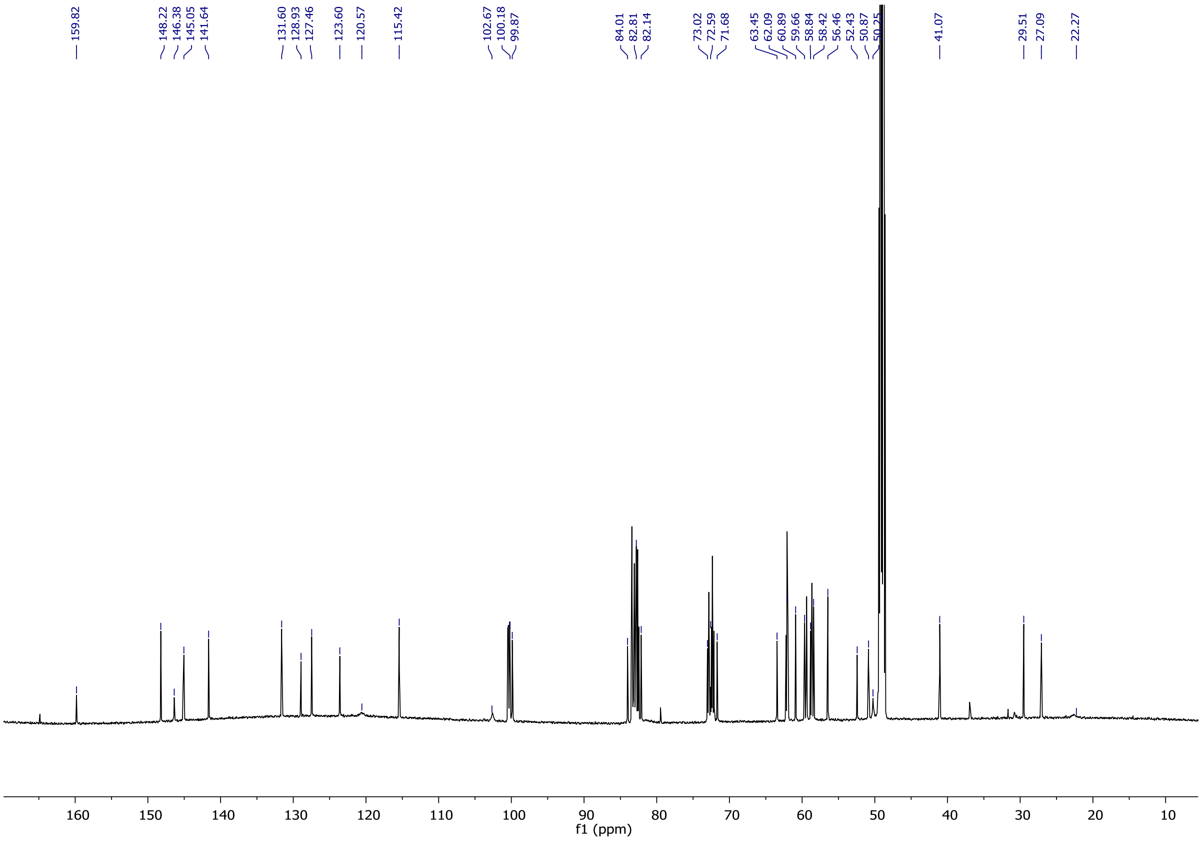

Figure S25: ^{13}C NMR spectrum of compound **8d**.

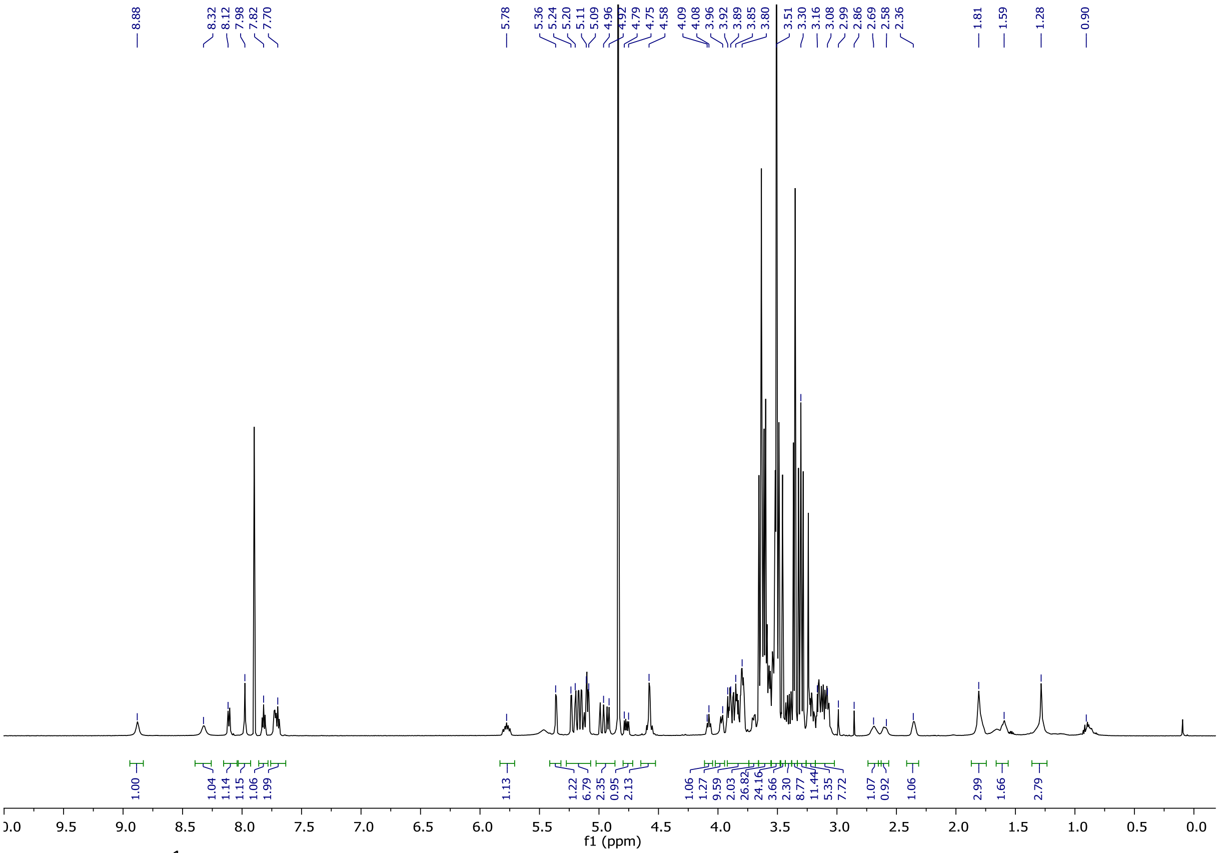

Figure S26: ^{1}H NMR spectrum of compound **9a**.

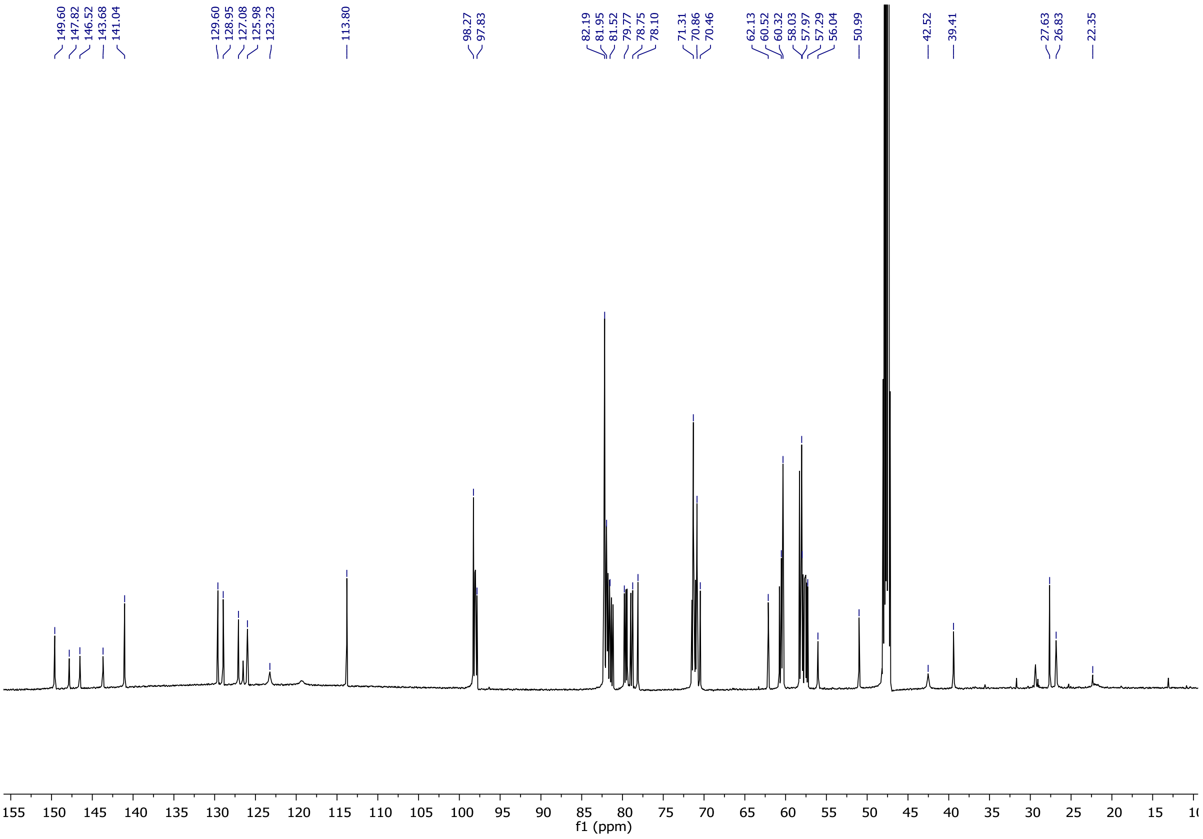

Figure S27: ^{13}C NMR spectrum of compound **9a**.

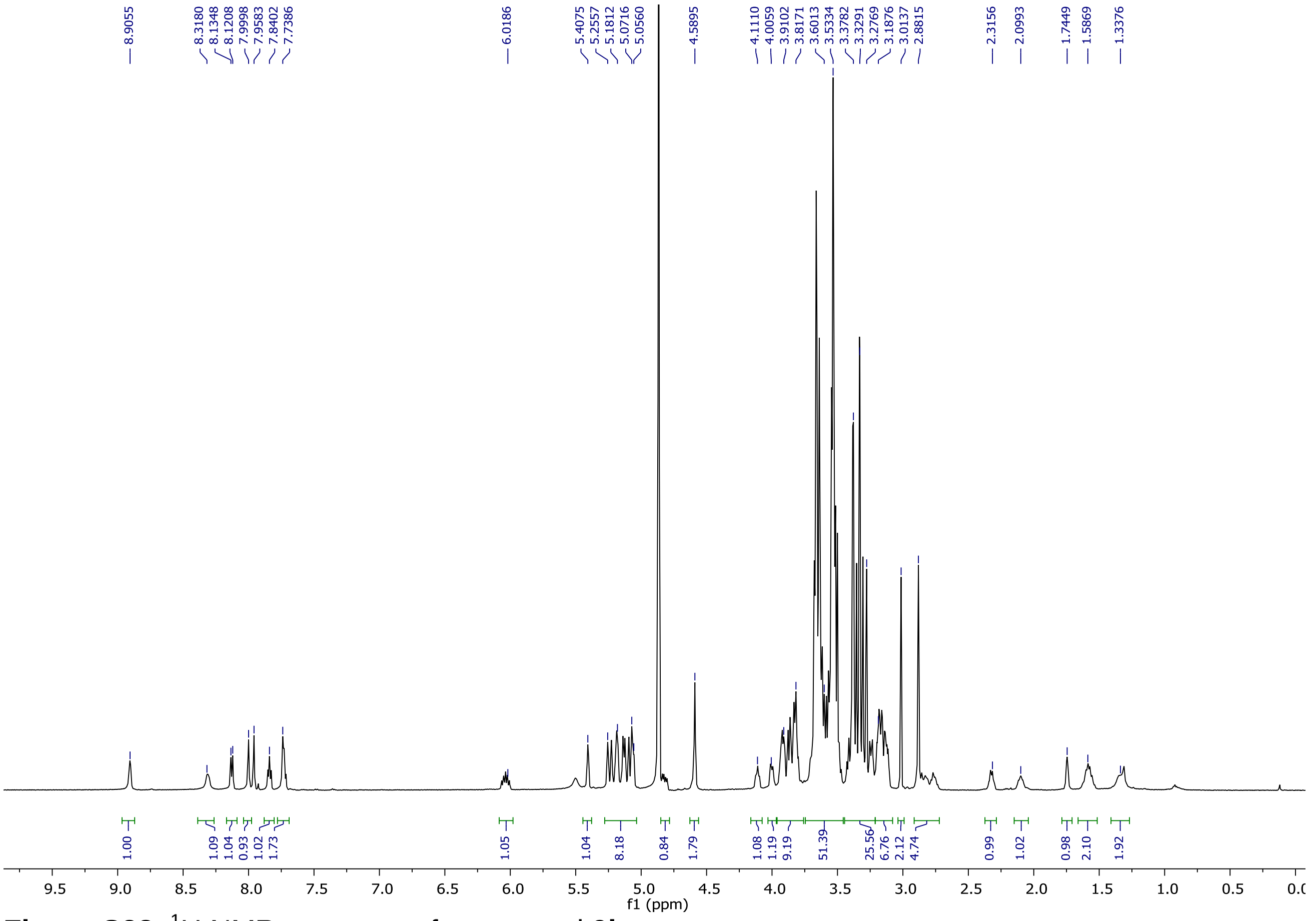

Figure S28: ^{1}H NMR spectrum of compound **9b**.

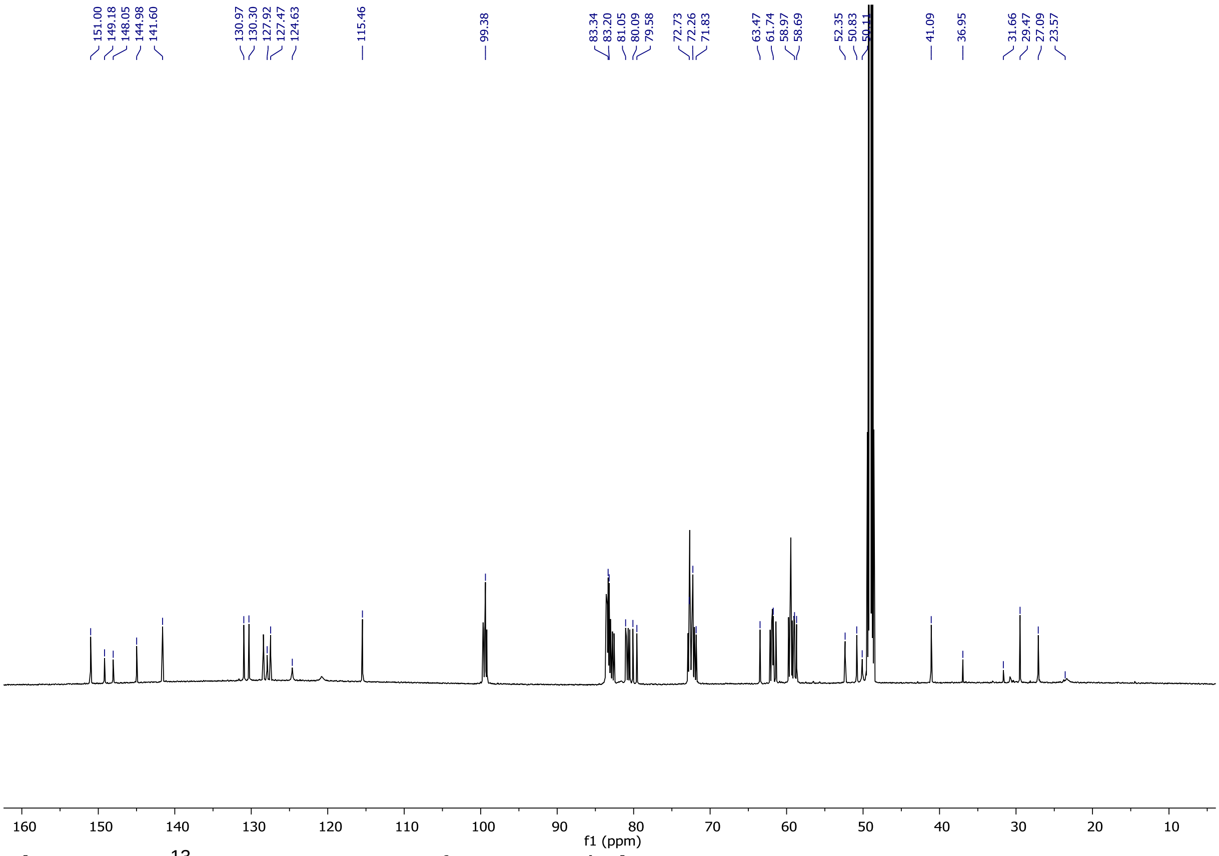

Figure S29: ^{13}C NMR spectrum of compound **9b**.

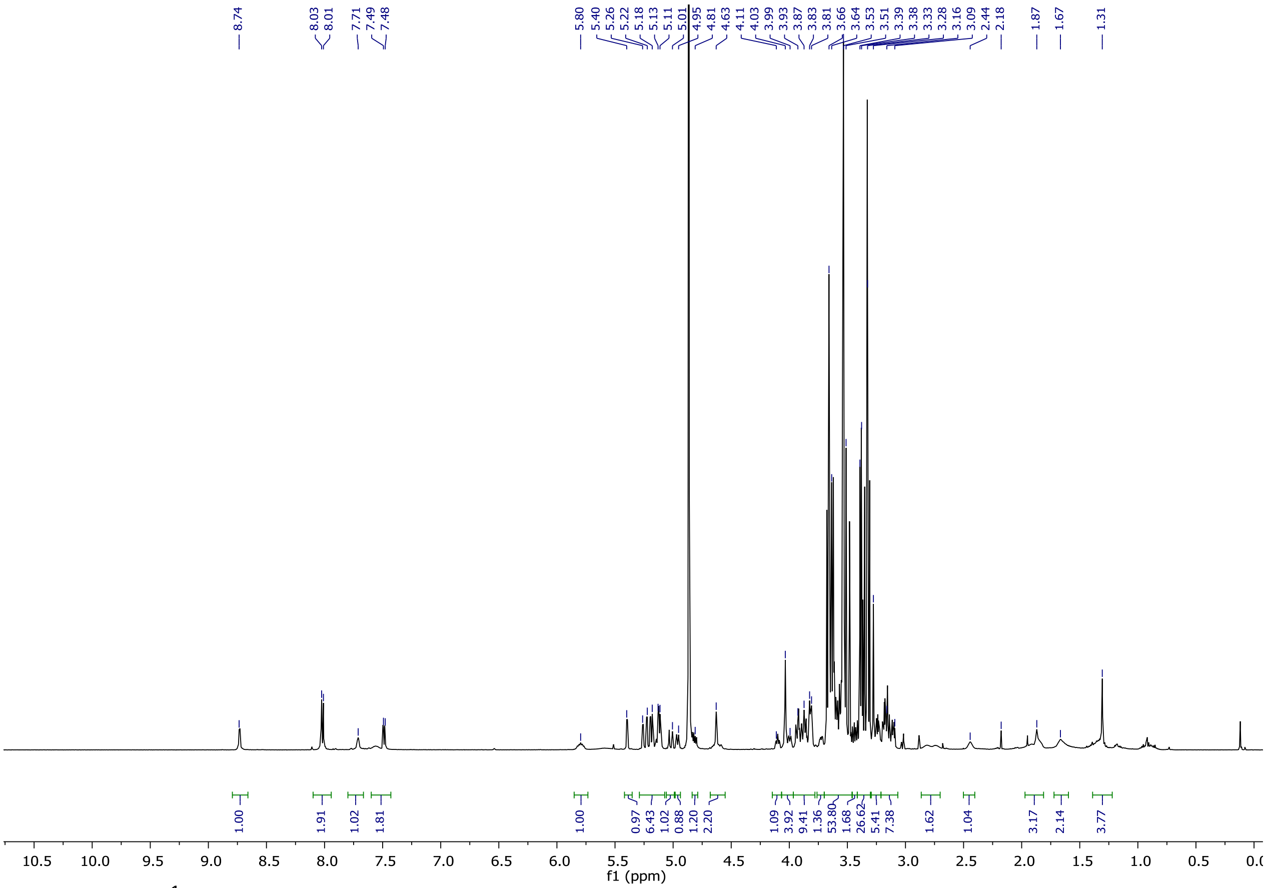

Figure S30: ^{1}H NMR spectrum of compound **9c**.

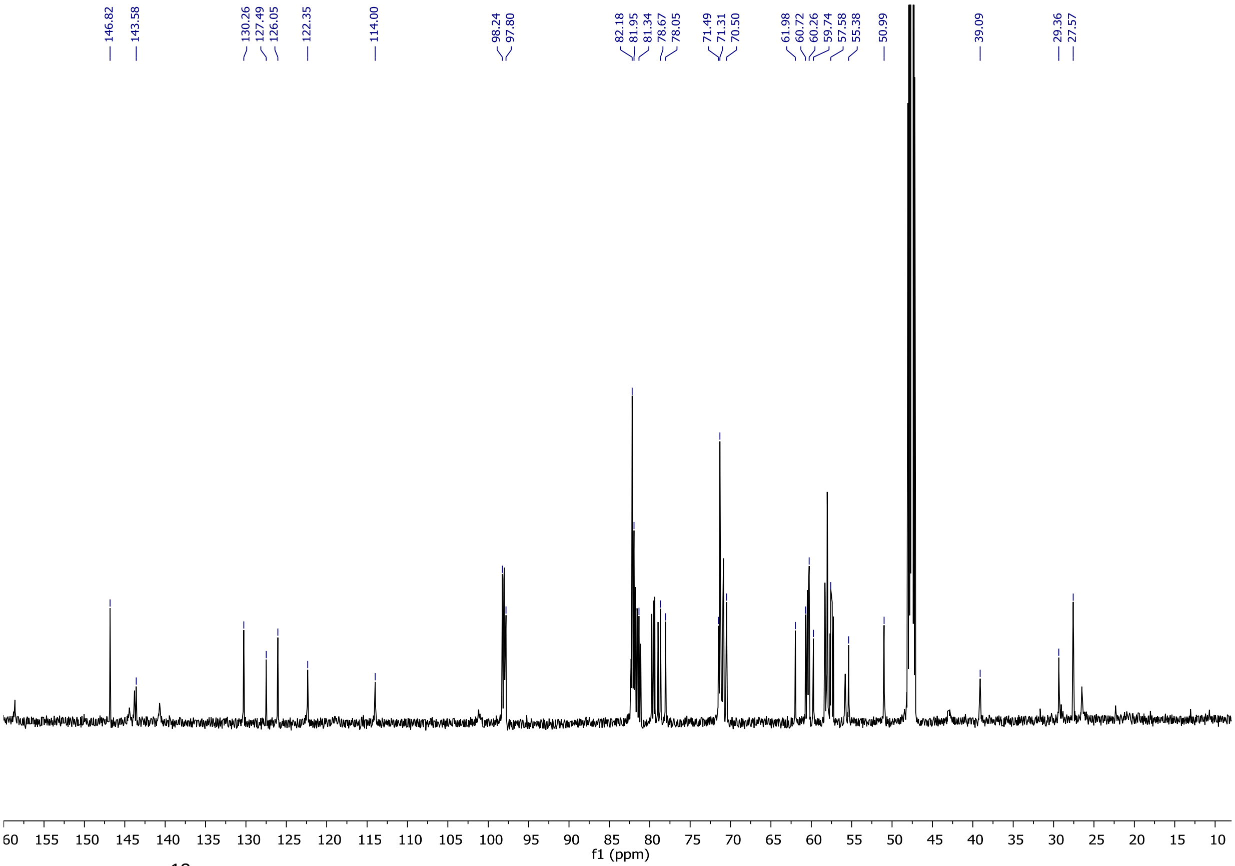

Figure S31: ^{13}C NMR spectrum of compound **9c**.

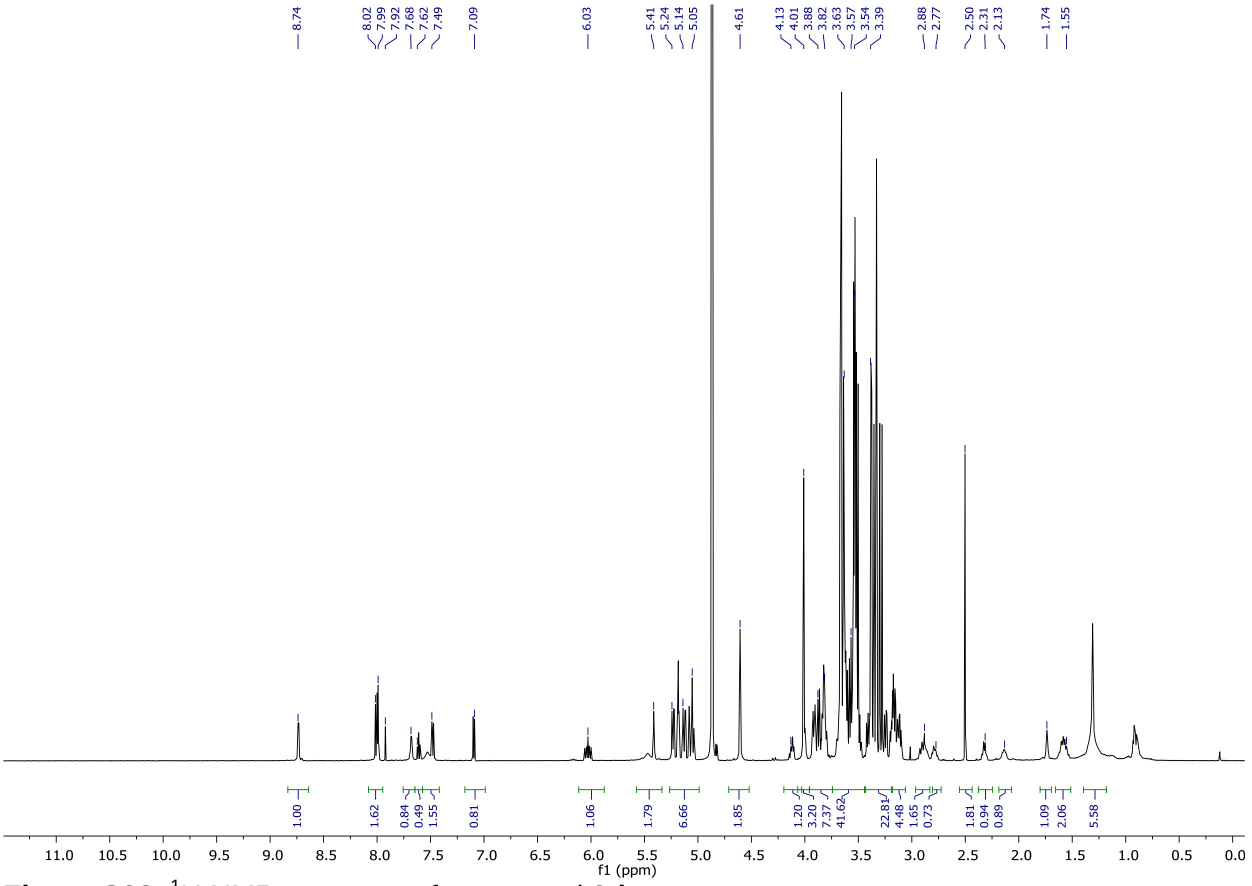

Figure S32: ^{1}H NMR spectrum of compound **9d**.

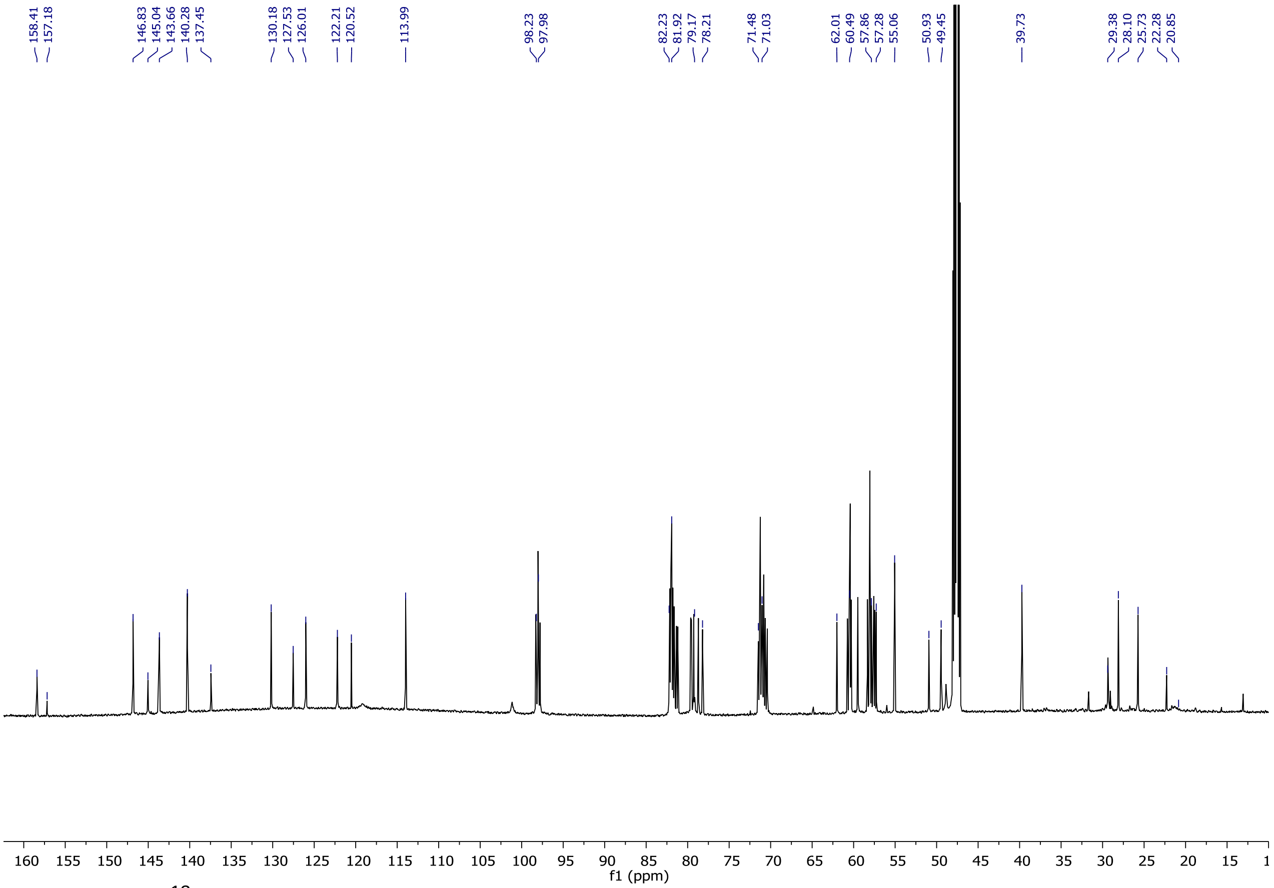

Figure S33: ^{13}C NMR spectrum of compound **9d**.

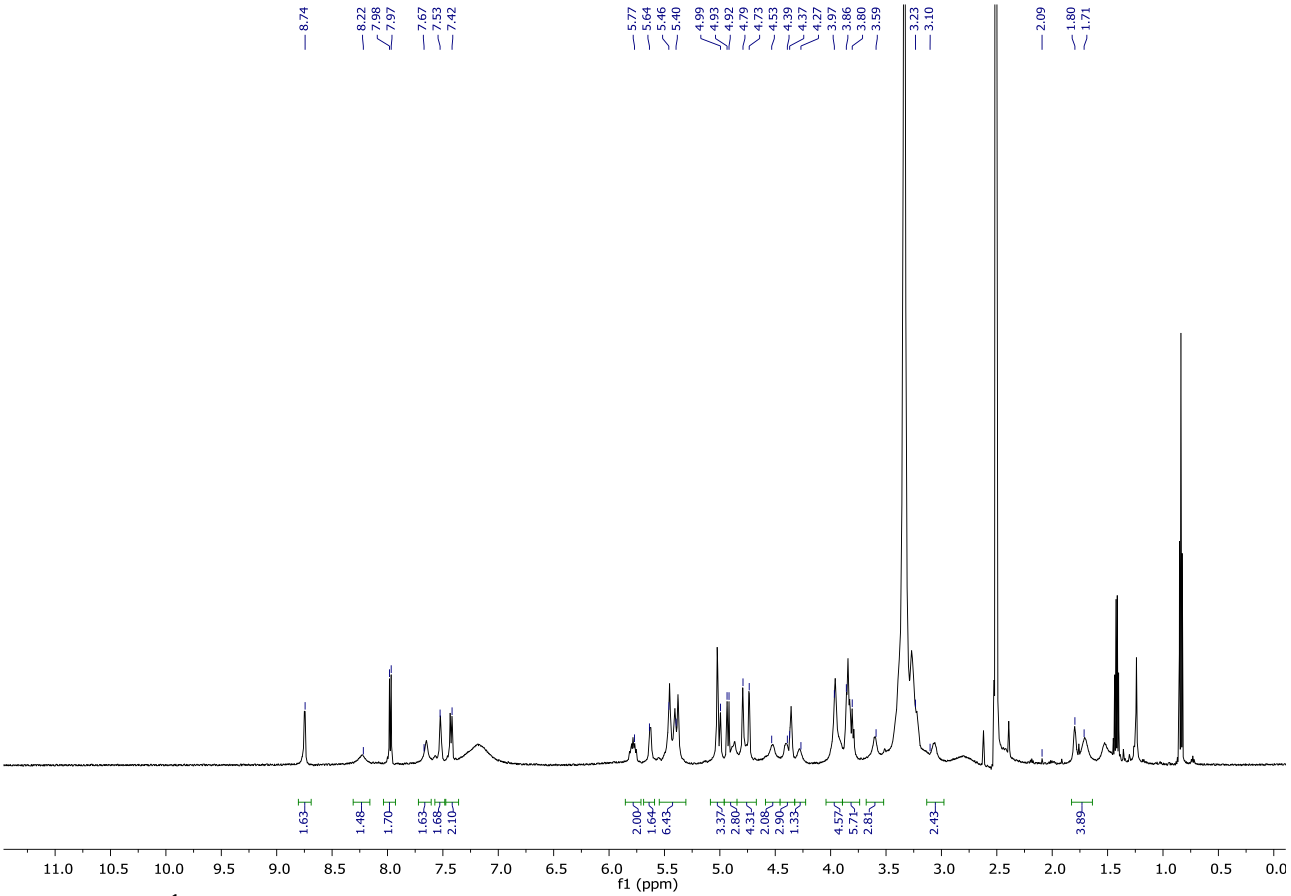

Figure S34: ^{1}H NMR spectrum of compound **11**.

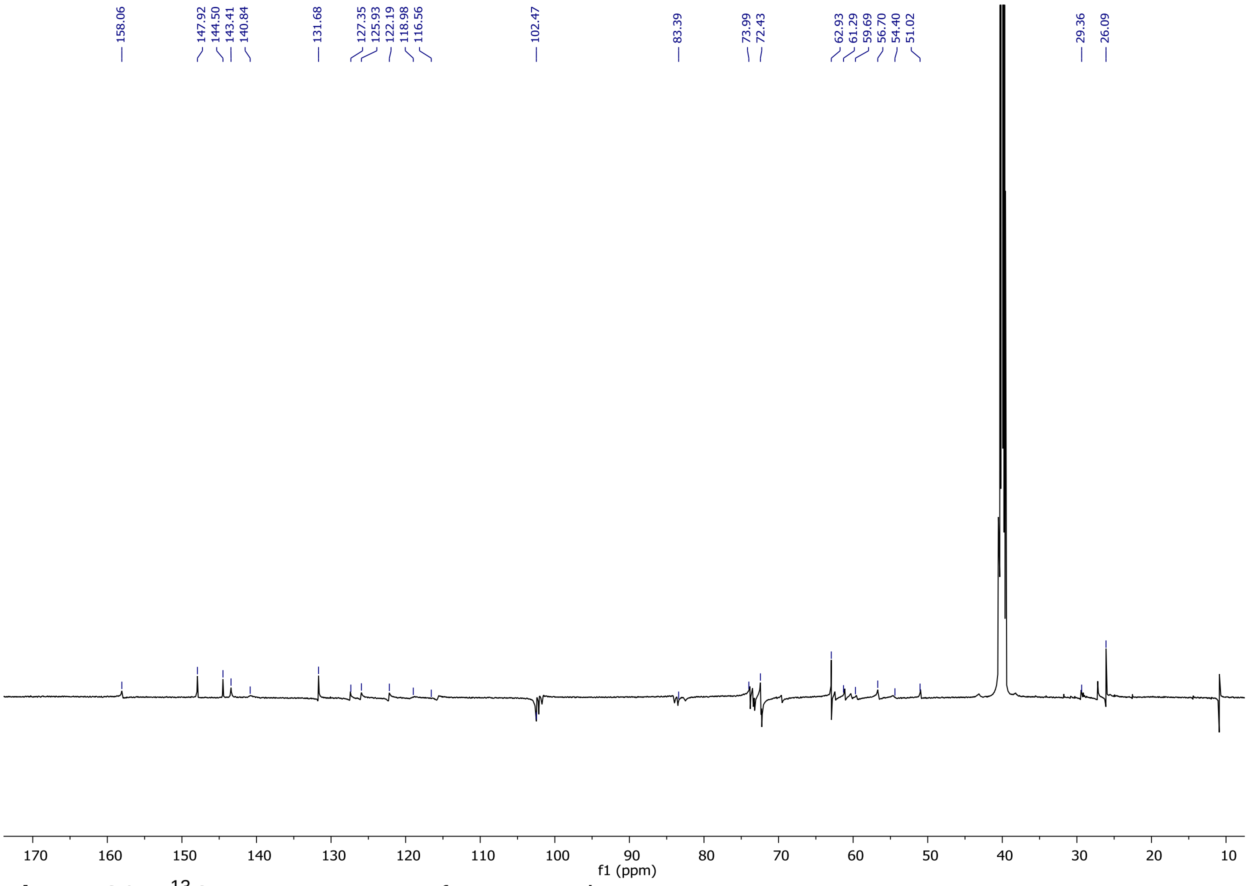

Figure S35: ^{13}C NMR spectrum of compound **11**.

HRMS spectra of prepared CD compounds

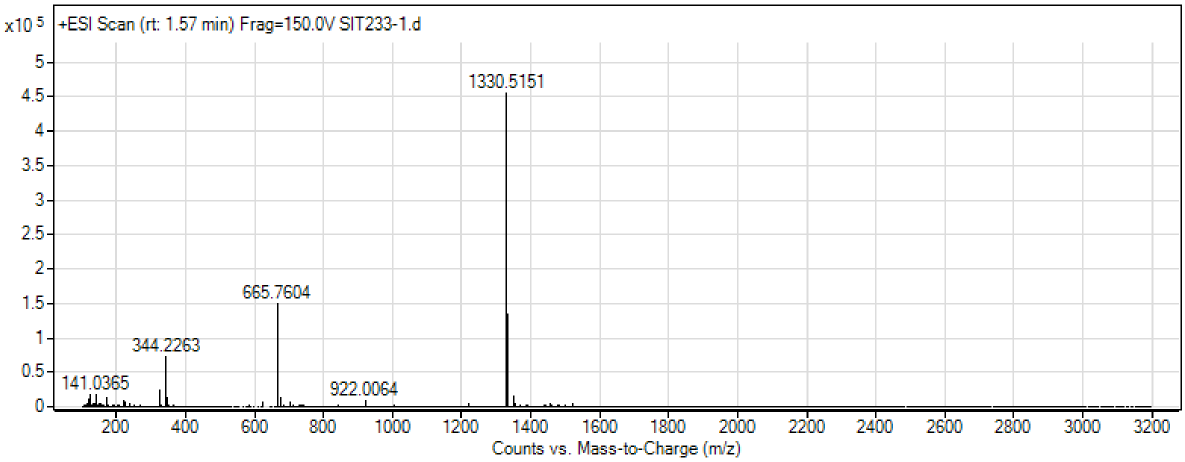

Figure S36: HRMS spectrum of compound **4a**.

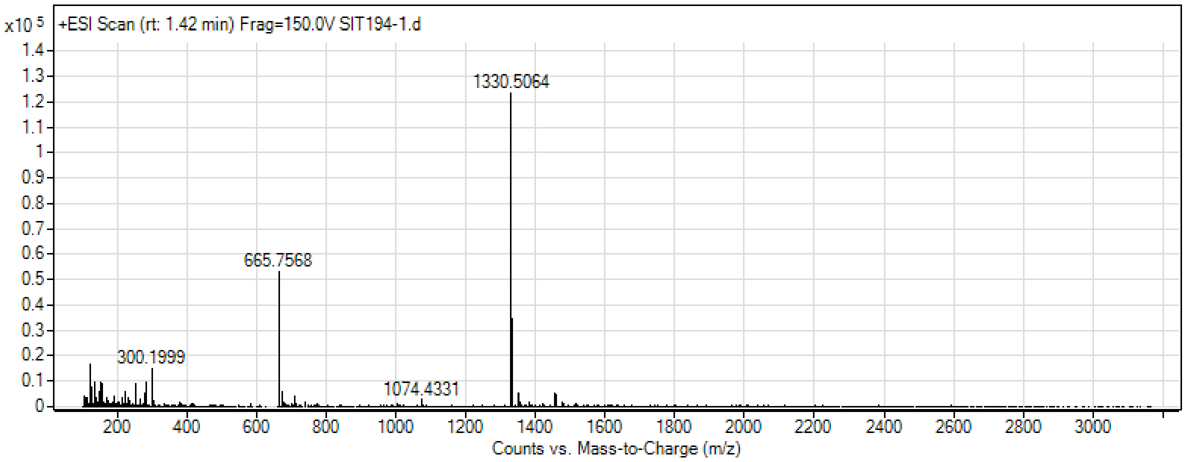

Figure S37: HRMS spectrum of compound **4b**.

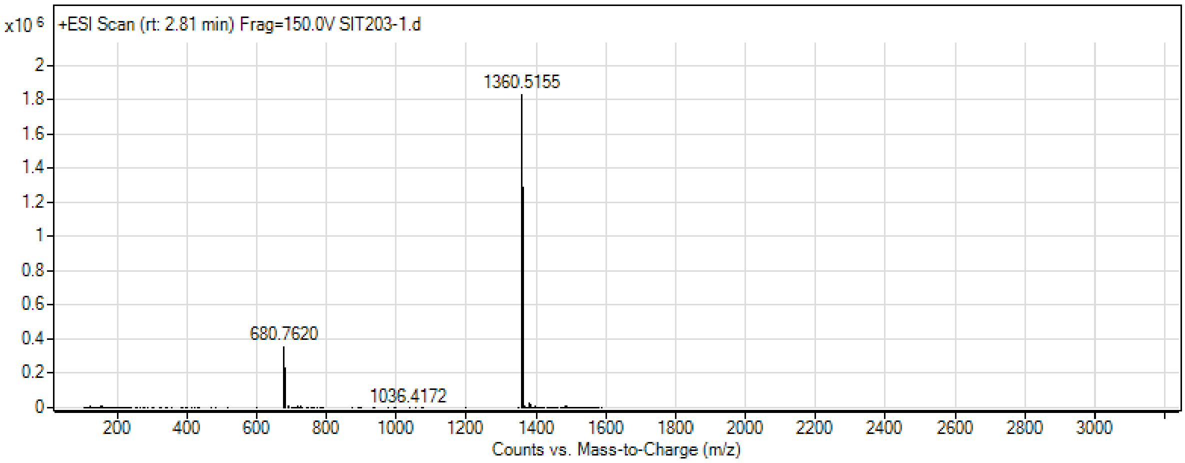

Figure S38: HRMS spectrum of compound **4c**.

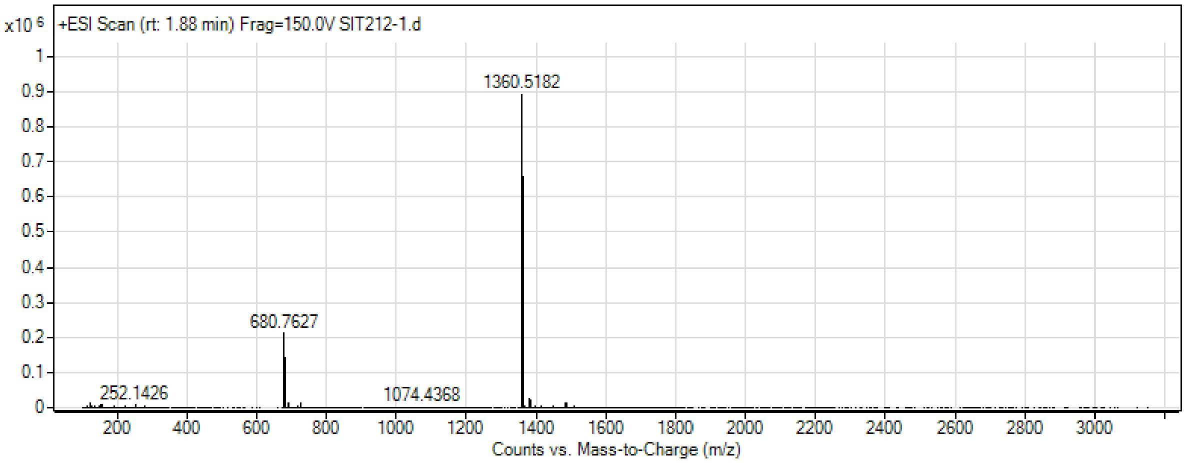

Figure S39: HRMS spectrum of compound **4d**.

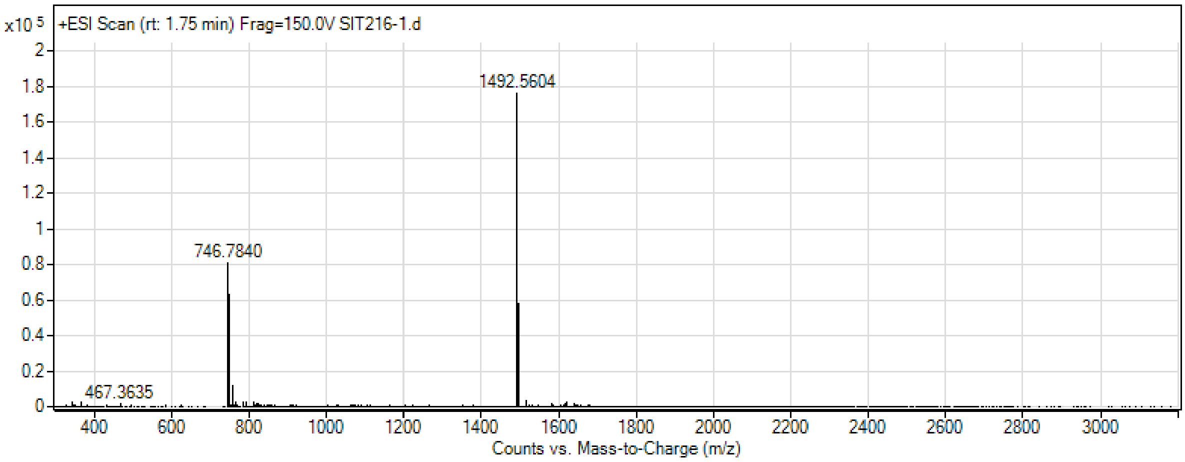

Figure S40: HRMS spectrum of compound **5a**.

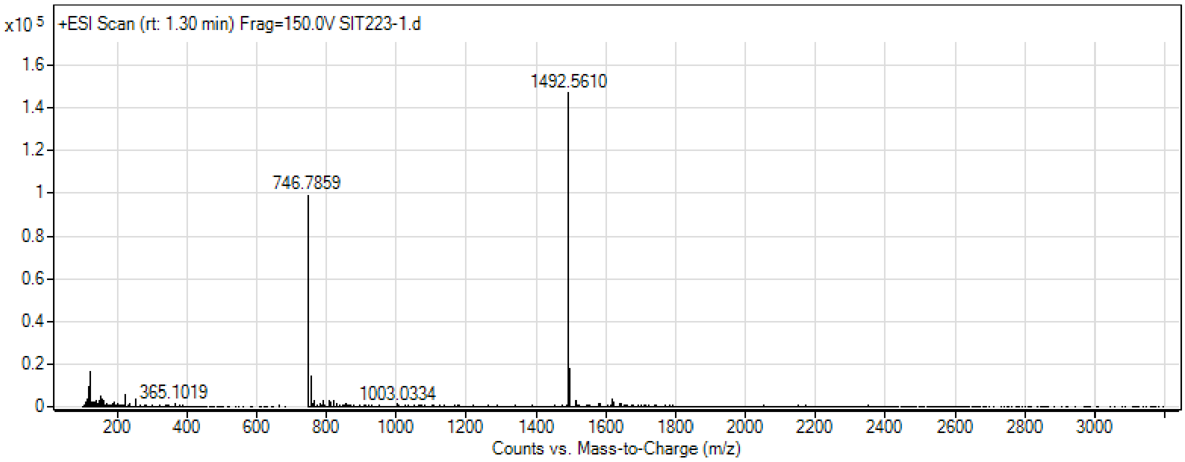

Figure S41: HRMS spectrum of compound **5b**.

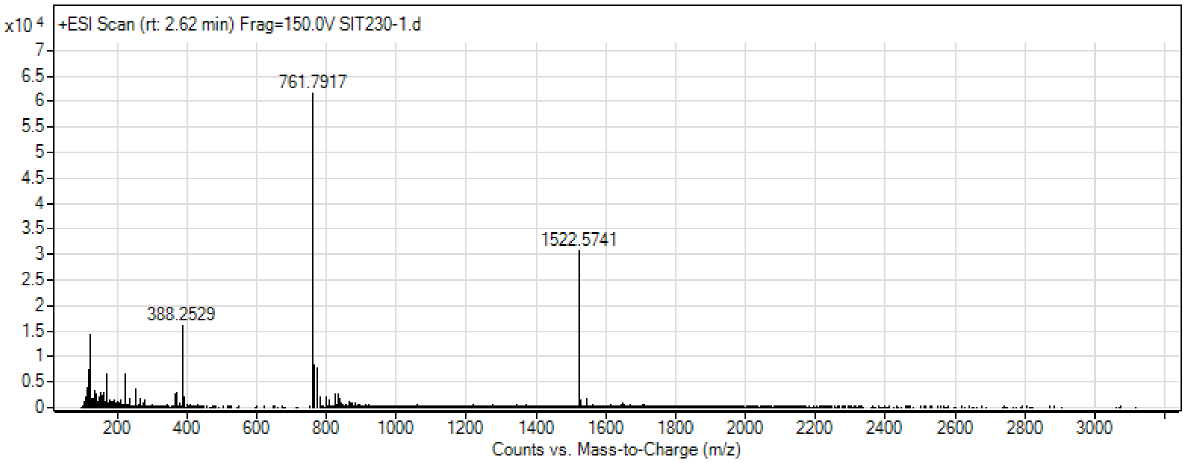

Figure S42: HRMS spectrum of compound **5c**.

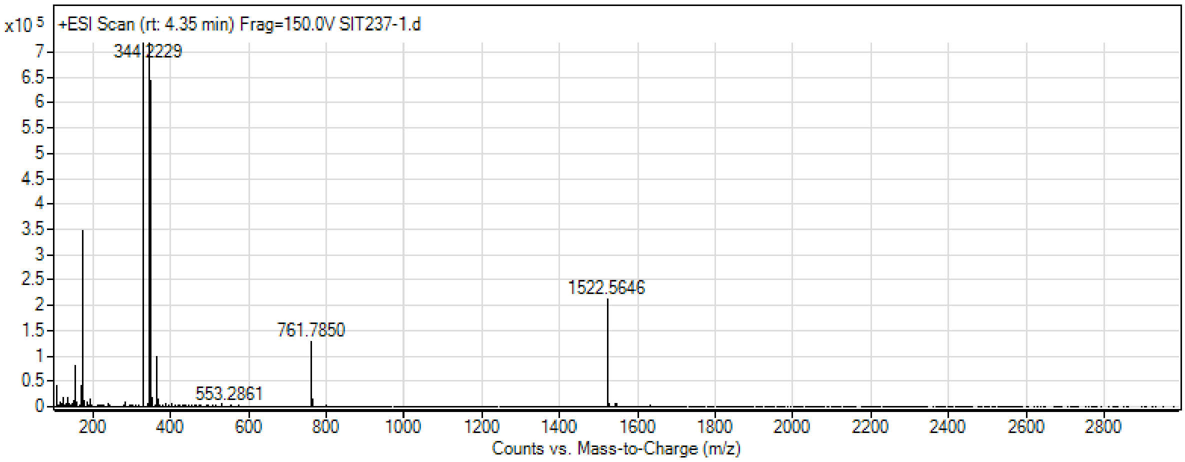

Figure S43: HRMS spectrum of compound **5d**.

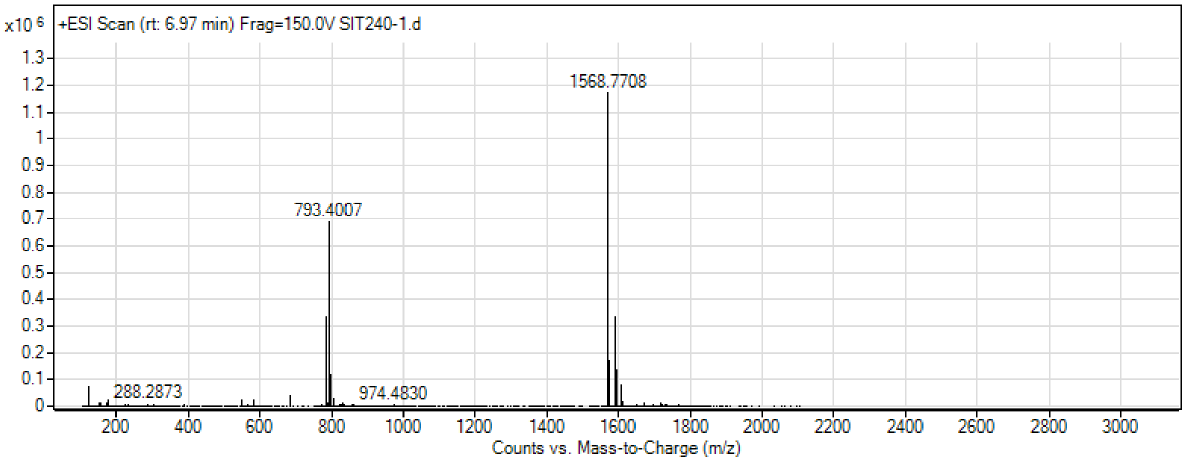

Figure S44: HRMS spectrum of compound **8a**.

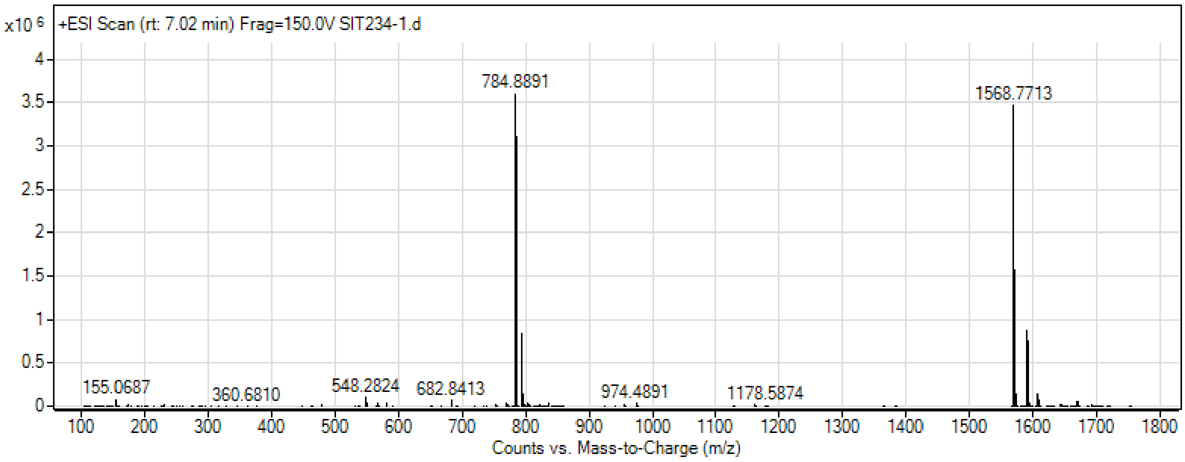

Figure S45: HRMS spectrum of compound **8b**.

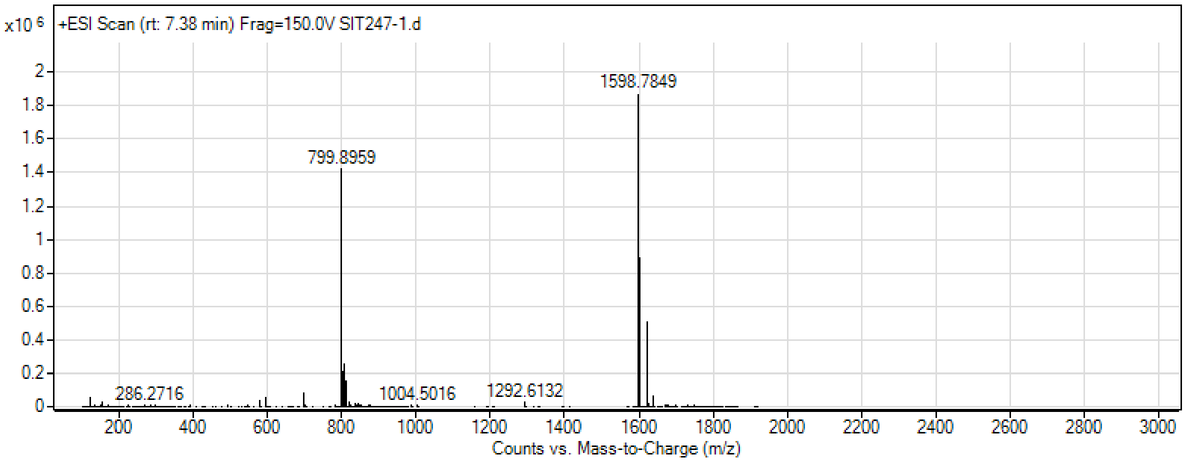

Figure S46: HRMS spectrum of compound **8c**.

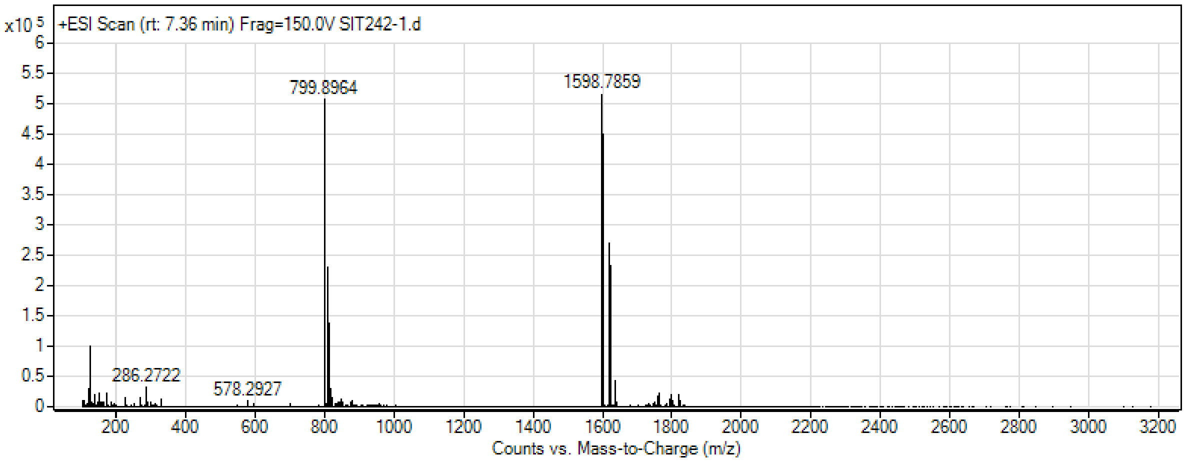

Figure S47: HRMS spectrum of compound **8d**.

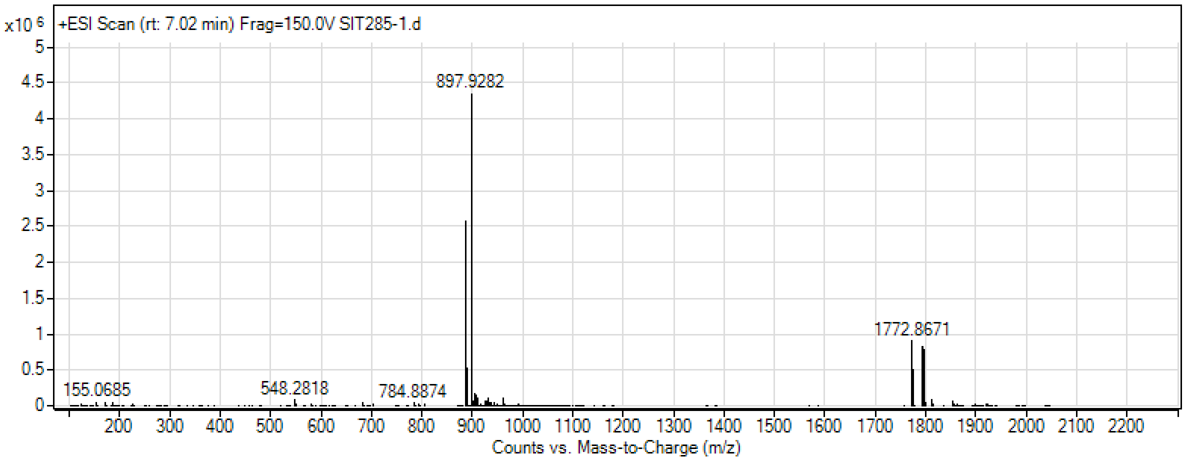

Figure S48: HRMS spectrum of compound **9a**.

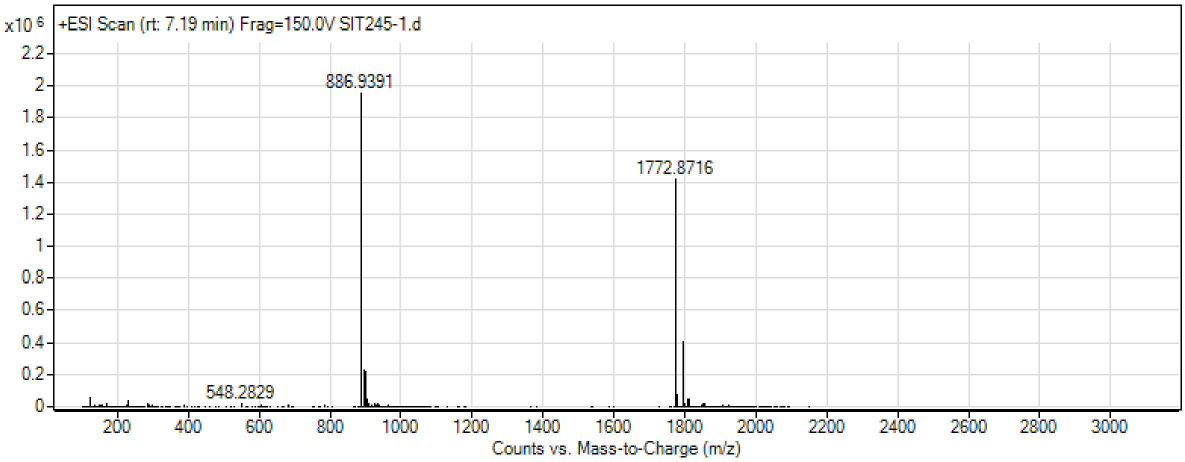

Figure S49: HRMS spectrum of compound **9b**.

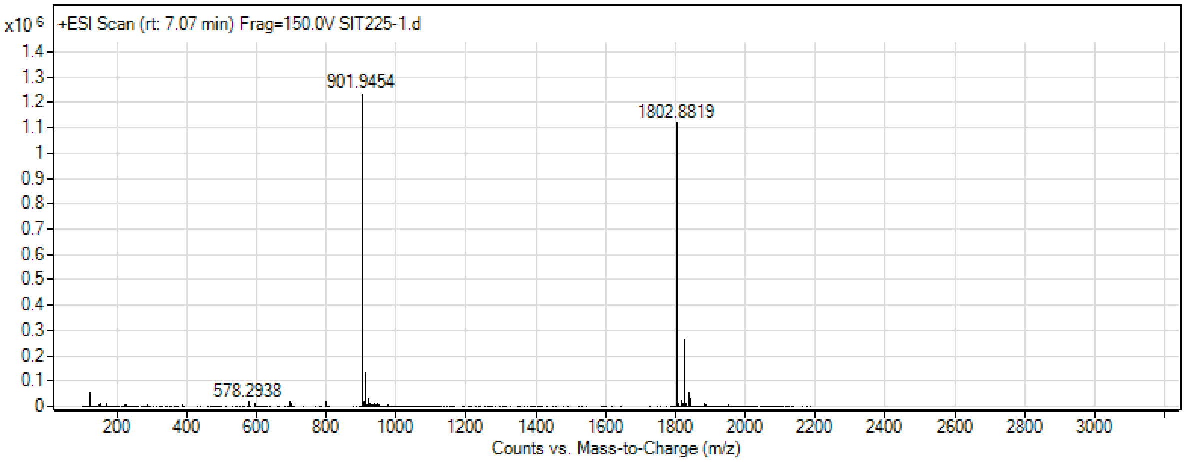

Figure S50: HRMS spectrum of compound **9c**.

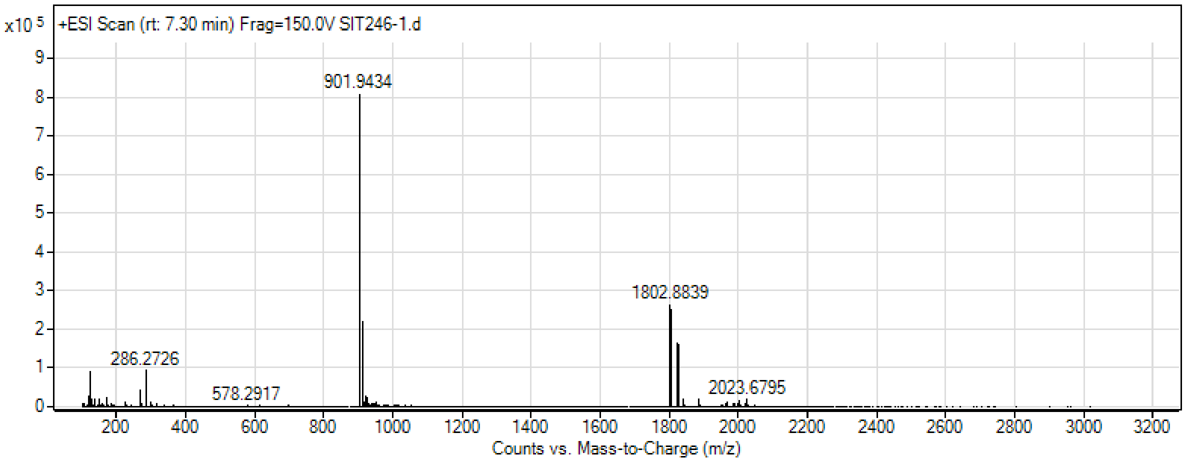

Figure S51: HRMS spectrum of compound **9d**.

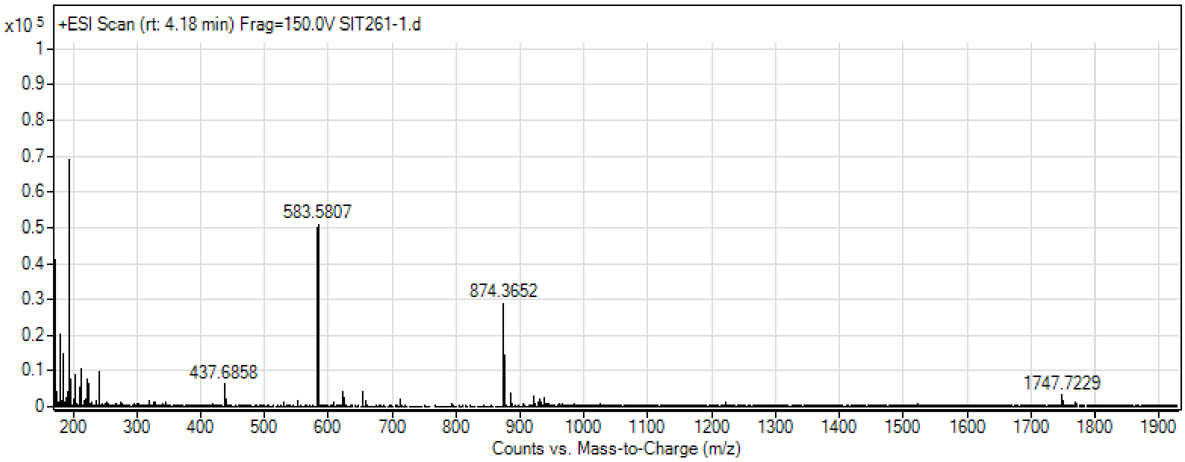

Figure S52: HRMS spectrum of compound **11**.

Chiral HPLC chromatograms

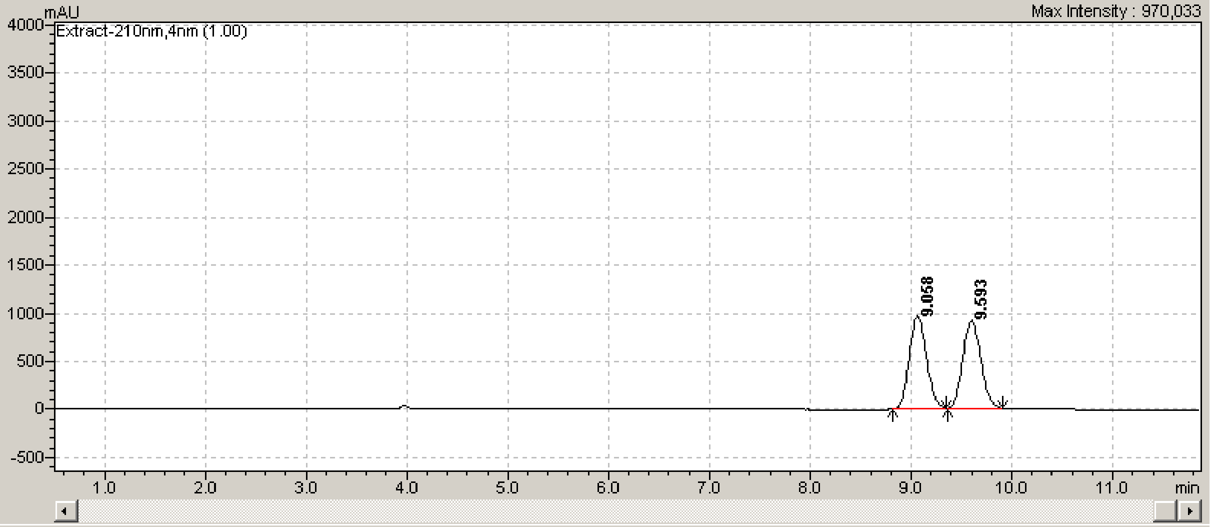

Peak Table

Peak#	ID#	Ret. Time	Conc.	Area	Height	Peak Start	Peak End	Area%
1		9.058	50.08058	11503801	963457	8.821	9.344	50.0806
2		9.593	49.91942	11466784	914222	9.365	9.909	49.9194

Figure S53: HPLC chromatogram of racemic product **13**.

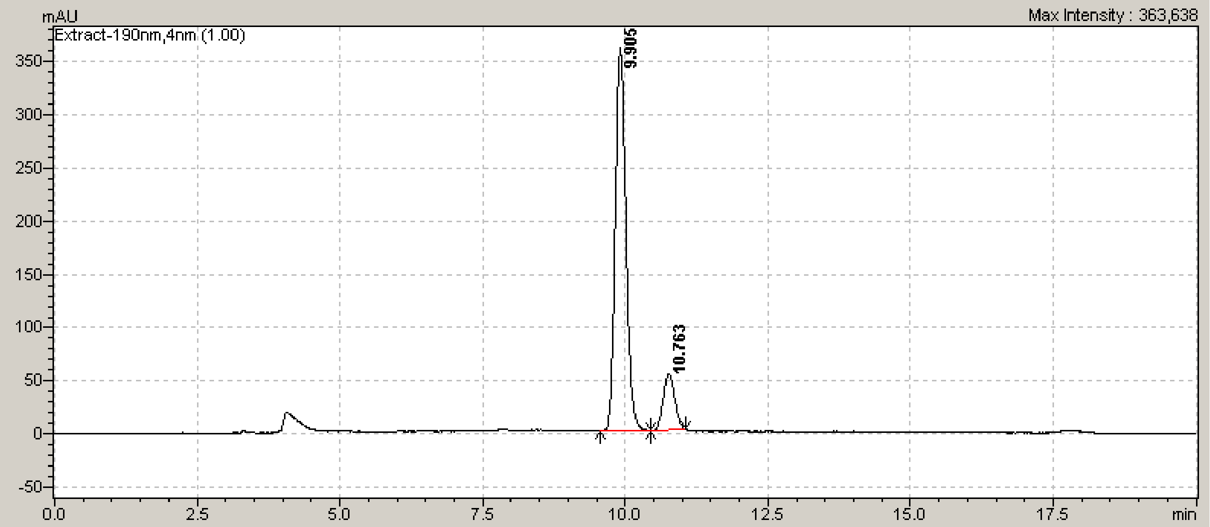

Peak Table

Peak#	ID#	Ret. Time	Conc.	Area	Height	Peak Start	Peak End	Area%
1		9.905	86.73262	4780624	360719	9.568	10.443	86.7326
2		10.763	13.26738	731286	52857	10.443	11.061	13.2674

Figure S54: HPLC chromatogram of chiral product **13** (catalyst **8a**).

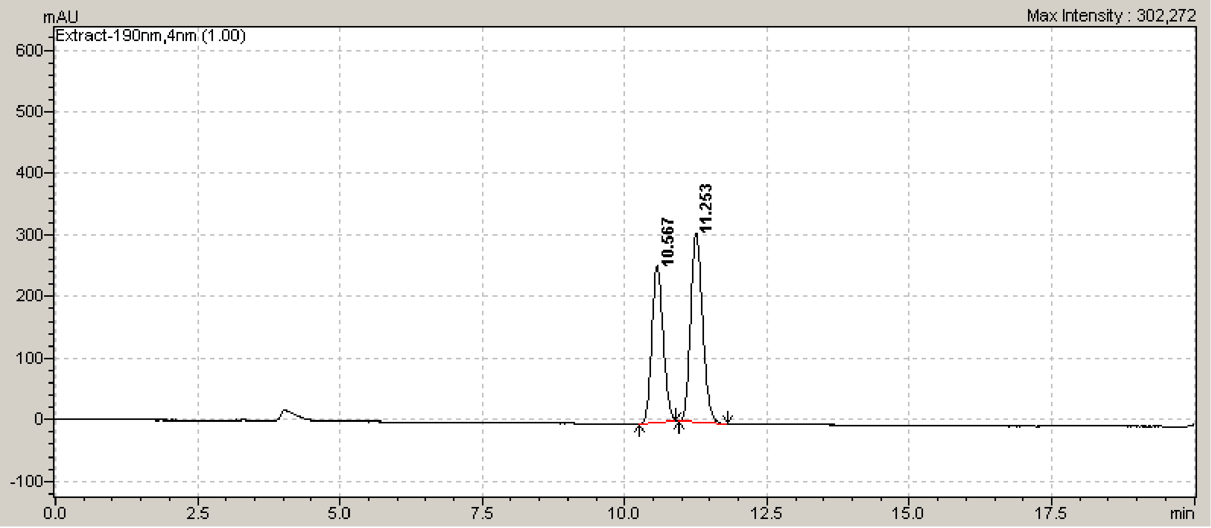

Peak Table

Peak#	ID#	Ret. Time	Conc.	Area	Height	Peak Start	Peak End	Area%
1		10.567	43.70220	3532174	253805	10.251	10.912	43.7022
2		11.253	56.29780	4550197	306394	10.944	11.797	56.2978

Figure S55: HPLC chromatogram of chiral product **13** (catalyst **8b**).

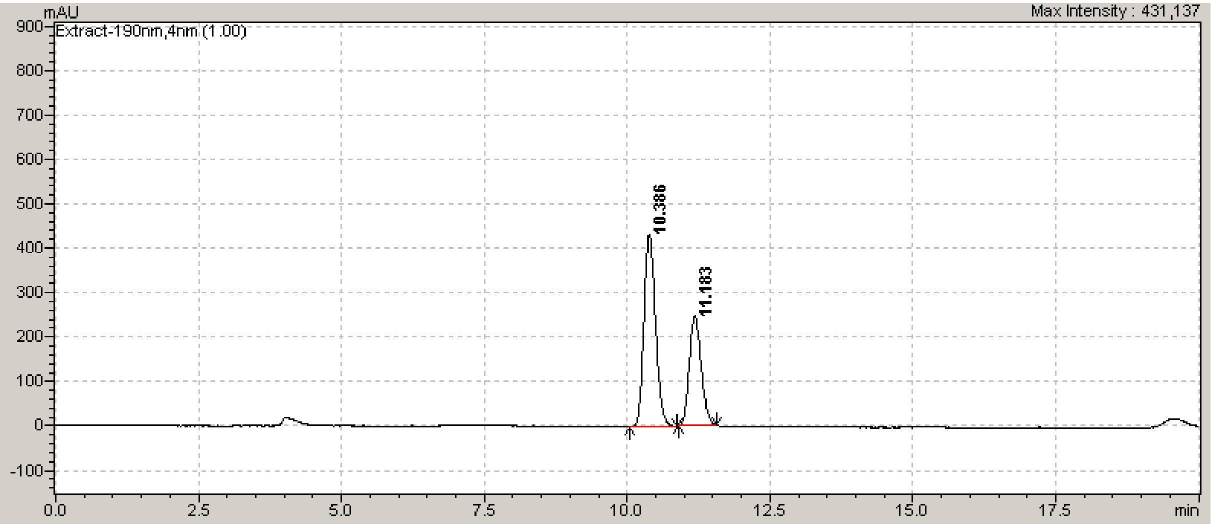

Peak Table

Peak#	ID#	Ret. Time	Conc.	Area	Height	Peak Start	Peak End	Area%
1		10.386	62.32941	6002694	432918	10.059	10.880	62.3294
2		11.183	37.67059	3627902	247336	10.912	11.573	37.6706

Figure S56: HPLC chromatogram of chiral product **13** (catalyst **8c**).

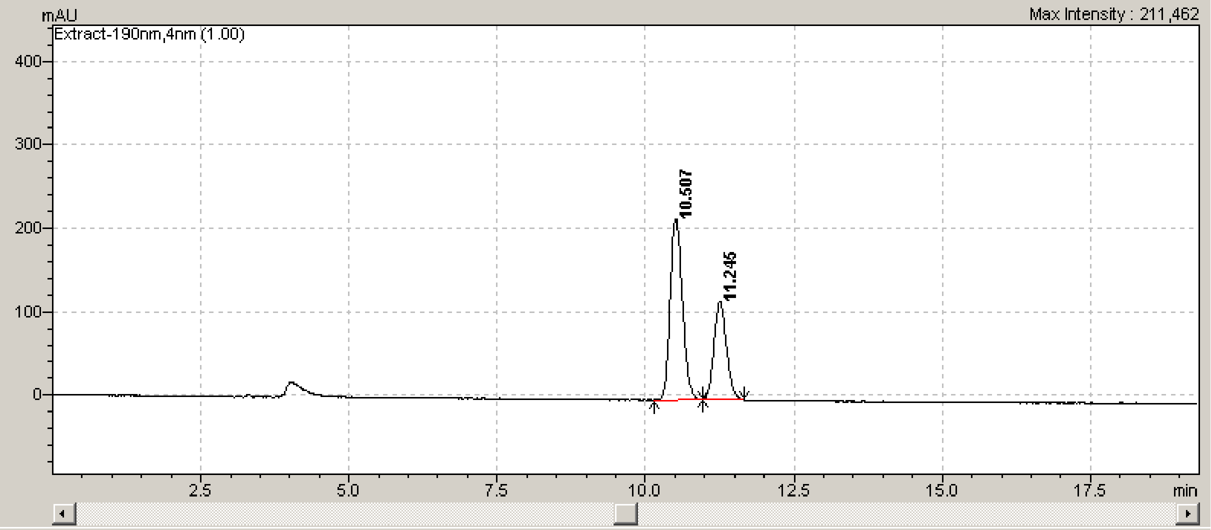

Peak Table

Peak#	ID#	Ret. Time	Conc.	Area	Height	Peak Start	Peak End	Area%
1		10.507	63.62087	3059348	216906	10.144	10.965	63.6209
2		11.245	36.37913	1749370	117583	10.965	11.659	36.3791

Figure S57: HPLC chromatogram of chiral product **13** (catalyst **8d**).

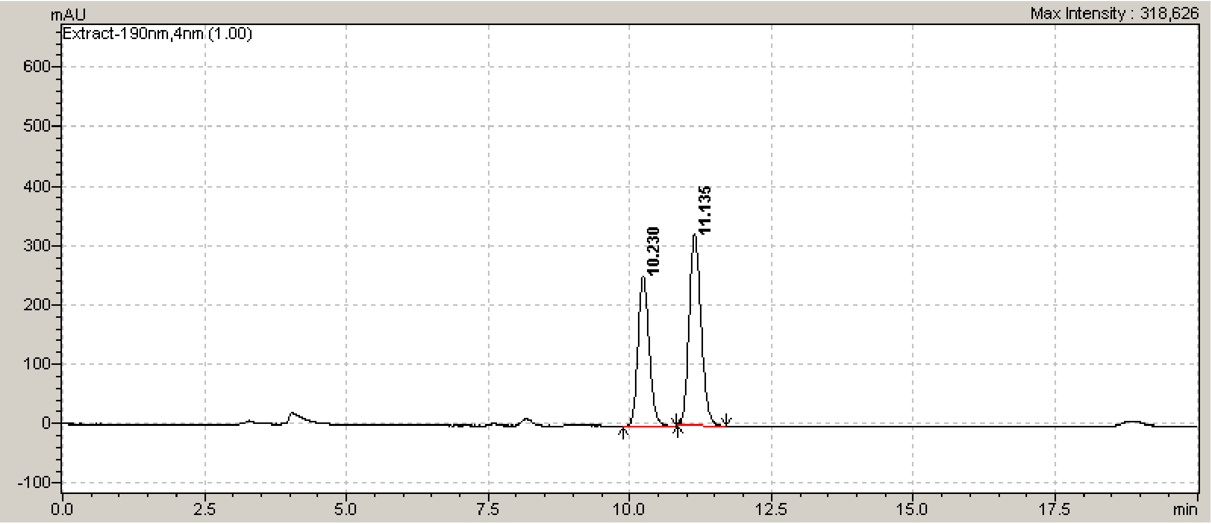

Peak Table

Peak#	ID#	Ret. Time	Conc.	Area	Height	Peak Start	Peak End	Area%
1		10.230	42.34221	3492986	253614	9.877	10.816	42.3422
2		11.135	57.65779	4756433	321884	10.837	11.712	57.6578

Figure S58: HPLC chromatogram of chiral product **13** (catalyst **9a**).

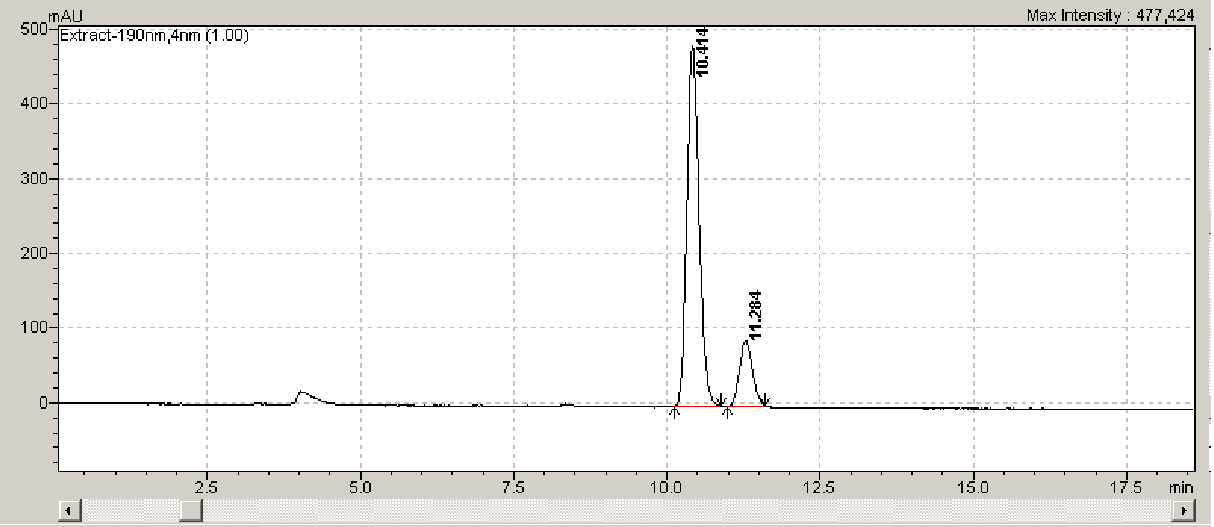

Peak Table

Peak#	ID#	Ret. Time	Conc.	Area	Height	Peak Start	Peak End	Area%
1		10.414	83.82985	6697262	481981	10.123	10.880	83.8298
2		11.284	16.17015	1291852	87472	10.976	11.616	16.1702

Figure S59: HPLC chromatogram of chiral product **13** (catalyst **9b**).

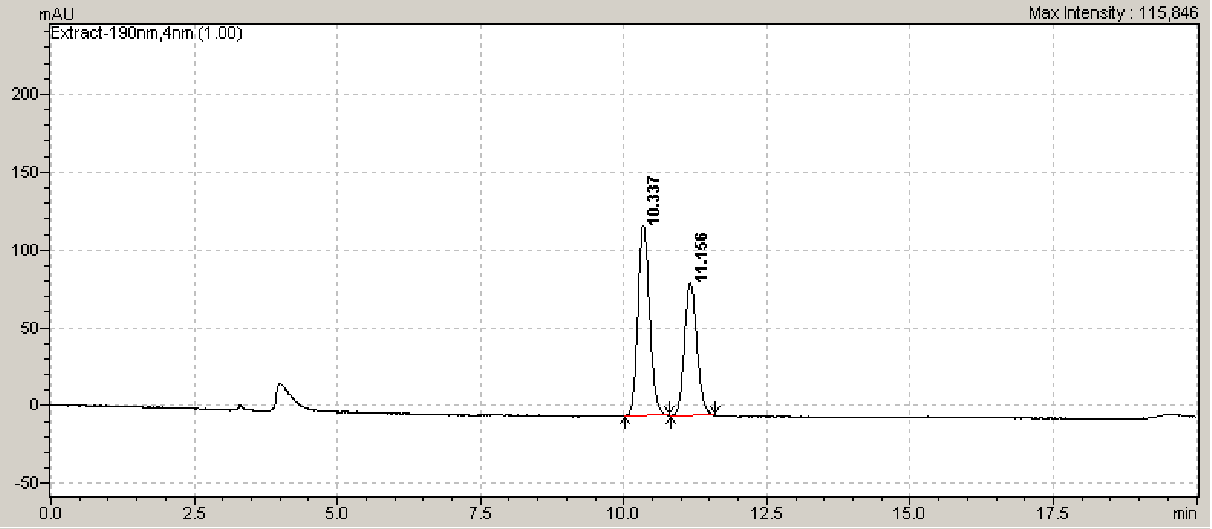

Peak Table

Peak#	ID#	Ret. Time	Conc.	Area	Height	Peak Start	Peak End	Area%
1		10.337	57.28755	1709529	121943	10.016	10.784	57.2876
2		11.156	42.71245	1274590	85100	10.816	11.584	42.7124

Figure S60: HPLC chromatogram of chiral product **13** (catalyst **9c**).

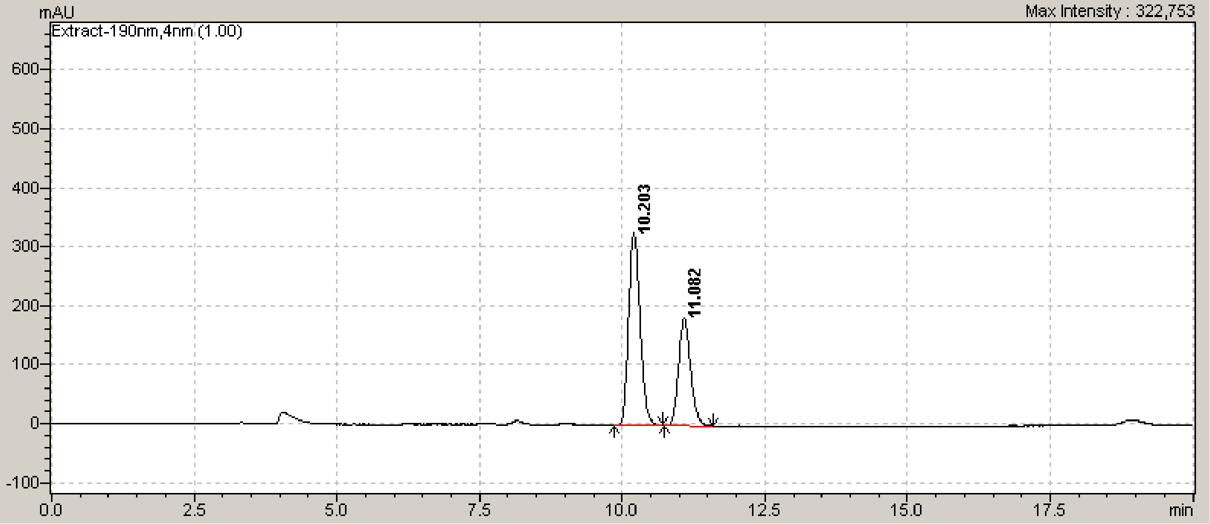

Peak Table

Peak#	ID#	Ret. Time	Conc.	Area	Height	Peak Start	Peak End	Area%
1		10.203	62.63667	4491112	325910	9.856	10.709	62.6367
2		11.082	37.36333	2678988	180910	10.731	11.584	37.3633

Figure S61: HPLC chromatogram of chiral product **13** (catalyst **9d**).

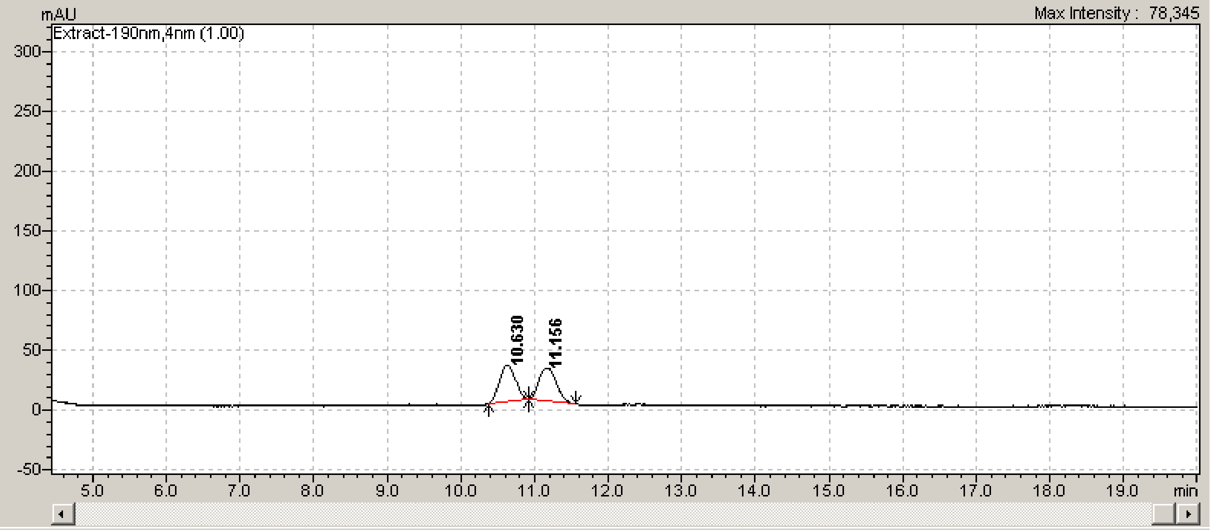

Peak Table

Peak#	ID#	Ret. Time	Conc.	Area	Height	Peak Start	Peak End	Area%
1		10.630	50.49993	468212	30765	10.368	10.912	50.4999
2		11.156	49.50007	458942	27997	10.912	11.563	49.5001

Figure S62: HPLC chromatogram of chiral product **13** (catalyst **4a**).

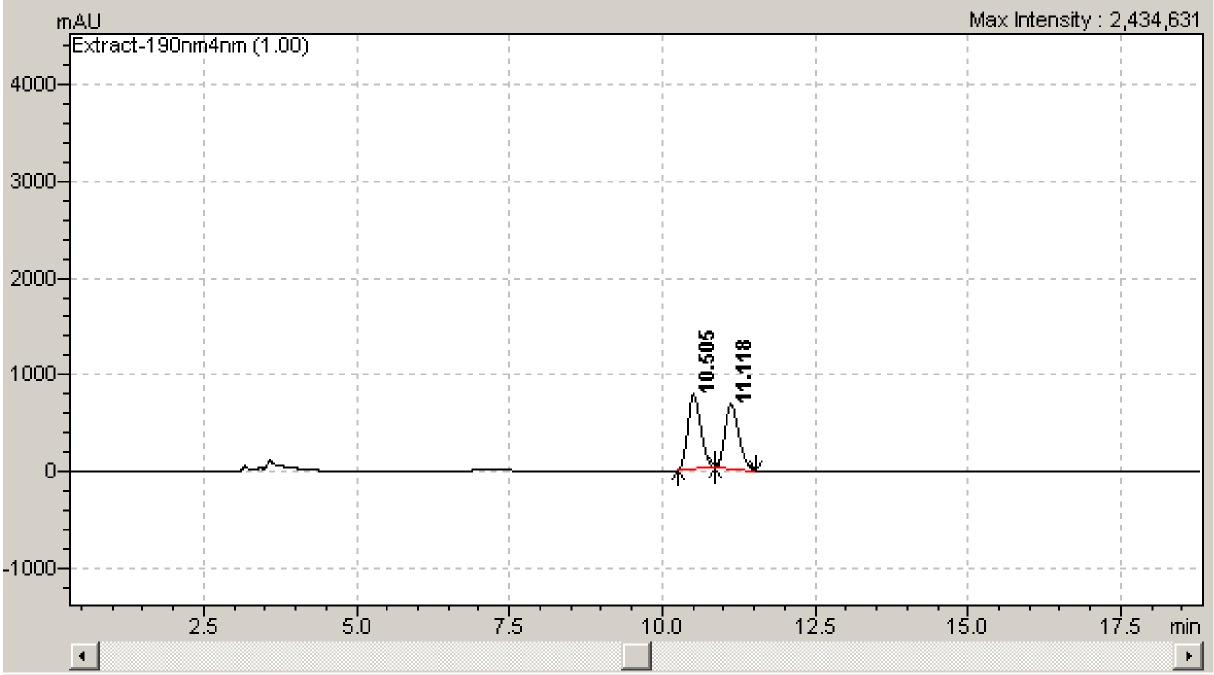

Peak Table

Peak#	ID#	Ret. Time	Conc.	Area	Height	Peak Start	Peak End	Area%
1		10.505	51.40051	11180505	777420	10.261	10.848	51.4005
2		11.118	48.59949	10571237	675552	10.848	11.541	48.5995

Figure S63: HPLC chromatogram of chiral product **13** (catalyst **4b**).

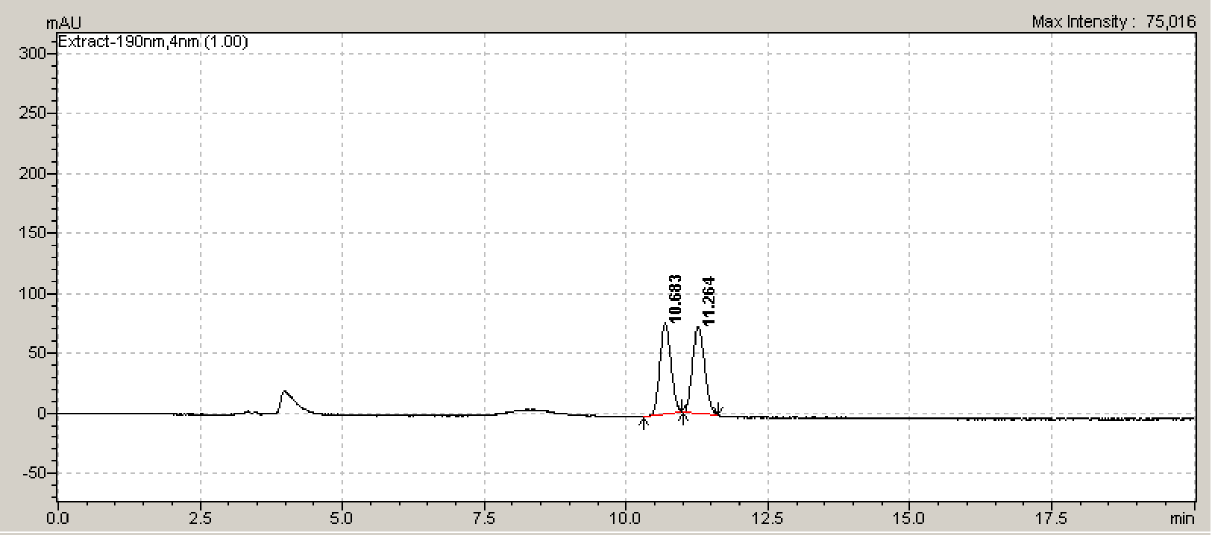

Peak Table

Peak#	ID#	Ret. Time	Conc.	Area	Height	Peak Start	Peak End	Area%
1		10.683	49.58074	1052343	76259	10.325	10.976	49.5807
2		11.264	50.41926	1070140	72988	10.997	11.627	50.4193

Figure S64: HPLC chromatogram of chiral product **13** (catalyst **4c**).

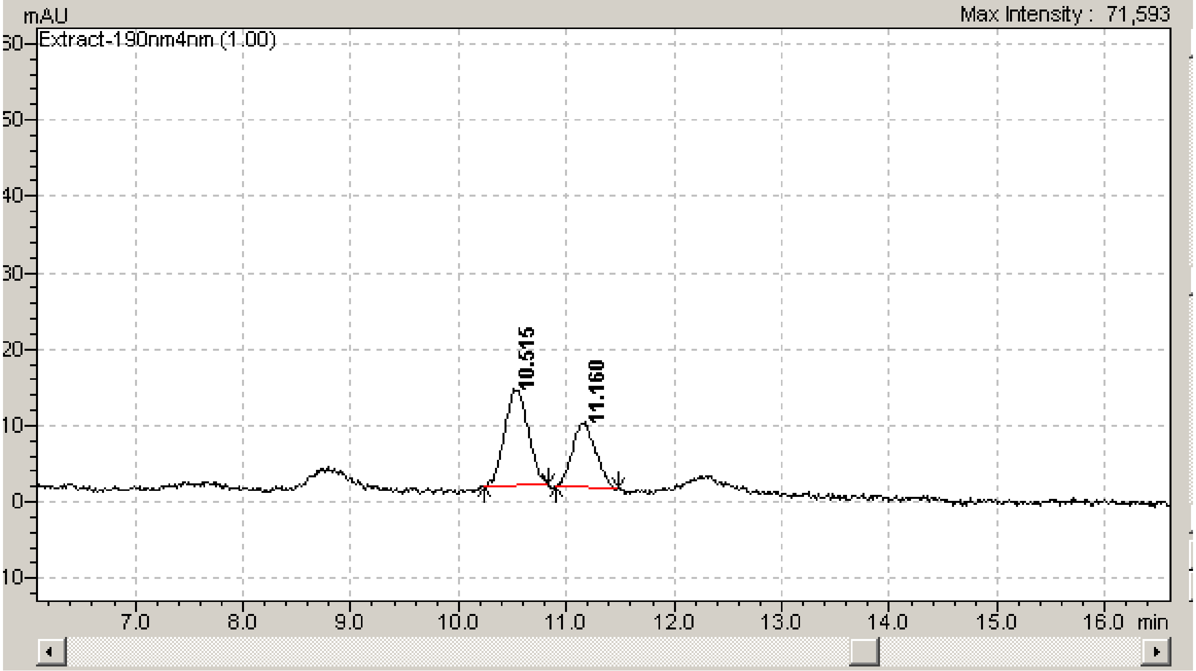

Peak Table

Peak#	ID#	Ret. Time	Conc.	Area	Height	Peak Start	Peak End	Area%
1		10.515	60.07654	193020	12619	10.240	10.837	60.0765
2		11.160	39.92346	128270	8496	10.901	11.488	39.9235

Figure S65: HPLC chromatogram of chiral product **13** (catalyst **4d**).

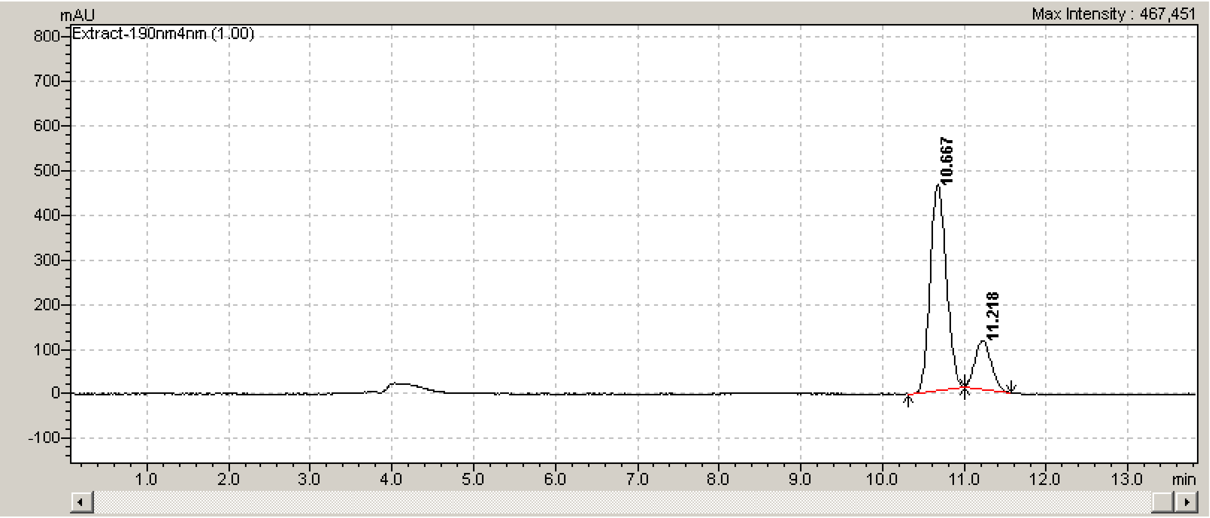

Peak Table

Peak#	ID#	Ret. Time	Conc.	Area	Height	Peak Start	Peak End	Area%
1		10.667	80.66815	6294632	460099	10.304	11.008	80.6681
2		11.218	19.33185	1508487	109689	11.008	11.573	19.3319

Figure S66: HPLC chromatogram of chiral product **13** (catalyst **5a**).

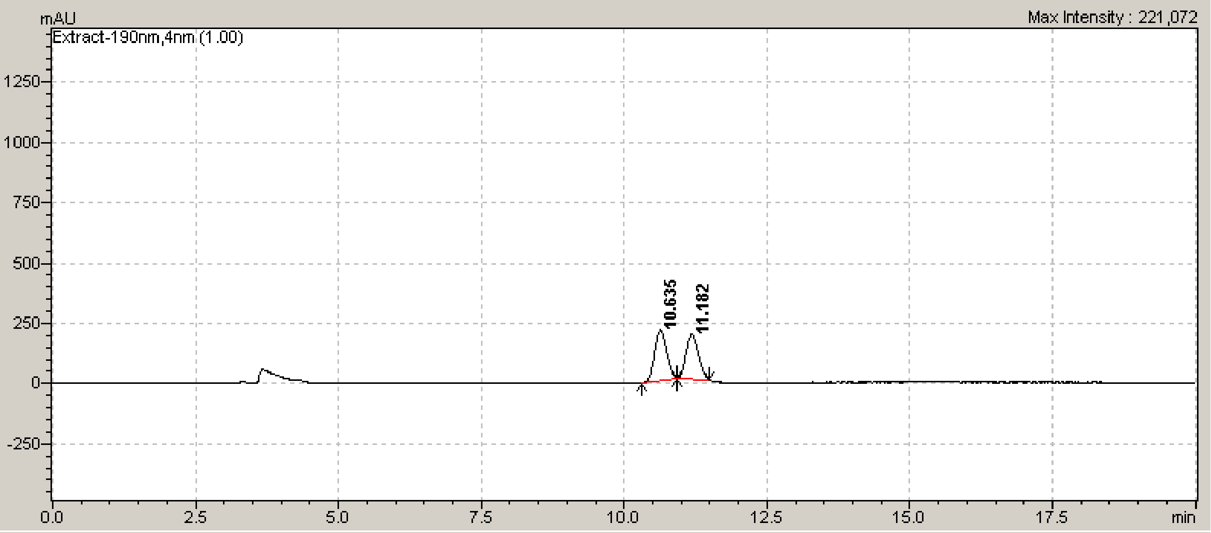

Peak Table

Peak#	ID#	Ret. Time	Conc.	Area	Height	Peak Start	Peak End	Area%
1		10.635	52.47964	3043538	207479	10.325	10.923	52.4796
2		11.182	47.52036	2755926	183013	10.923	11.488	47.5204

Figure S67: HPLC chromatogram of chiral product **13** (catalyst **5b**).

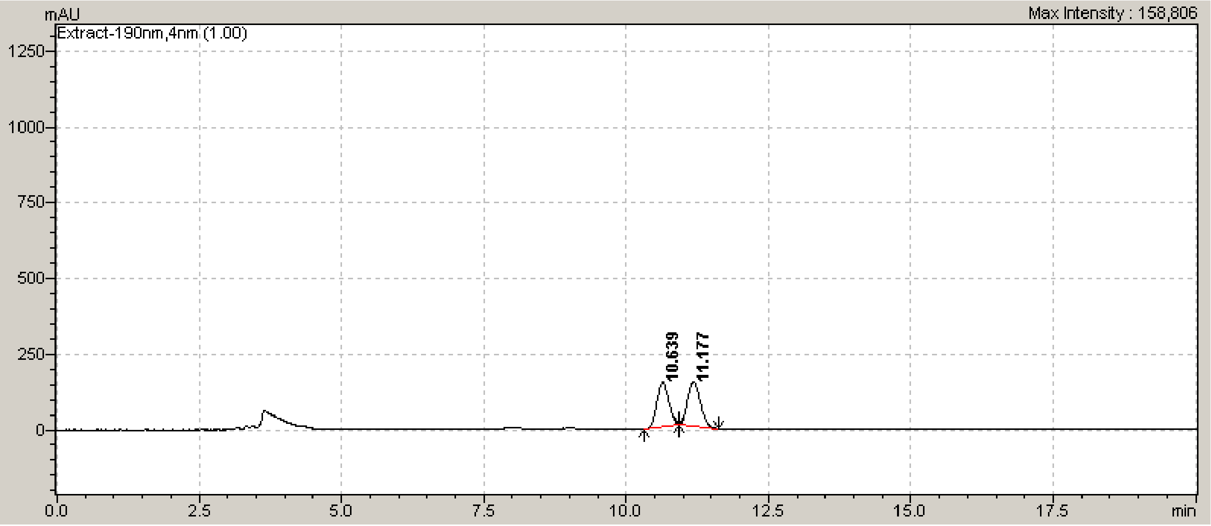

Peak Table

Peak#	ID#	Ret. Time	Conc.	Area	Height	Peak Start	Peak End	Area%
1		10.639	48.47170	2136562	146708	10.315	10.933	48.4717
2		11.177	51.52830	2271293	145882	10.933	11.616	51.5283

Figure S68: HPLC chromatogram of chiral product **13** (catalyst **5c**).

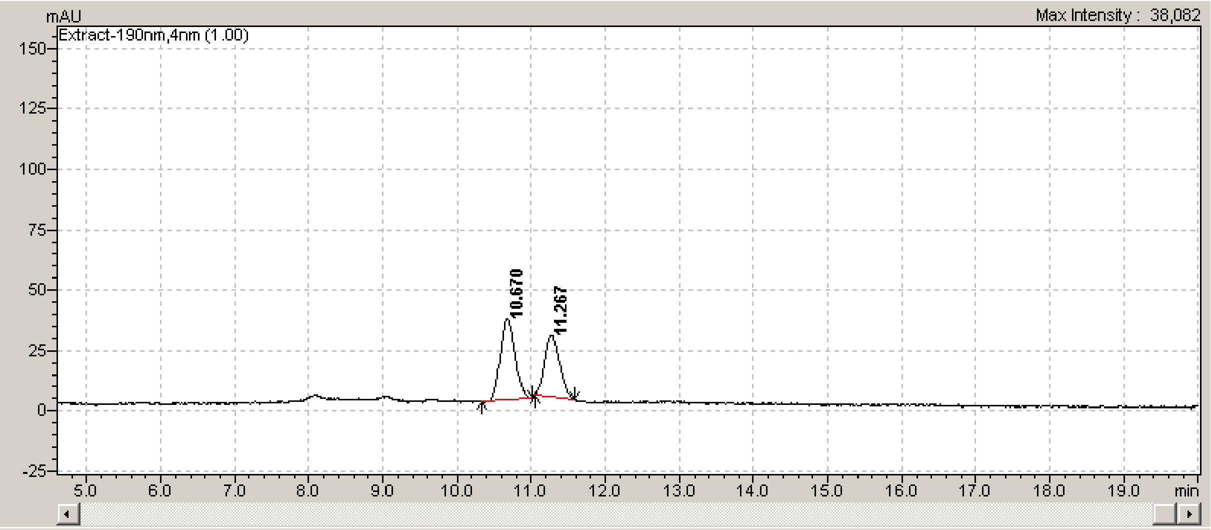

Peak Table

Peak#	ID#	Ret. Time	Conc.	Area	Height	Peak Start	Peak End	Area%
1		10.670	56.06584	463822	33477	10.325	11.019	56.0658
2		11.267	43.93416	363458	25786	11.051	11.595	43.9342

Figure S69: HPLC chromatogram of chiral product **13** (catalyst **5d**).
