# Supplementary material for: New α- and β-cyclodextrin derivatives with cinchona alkaloids used in asymmetric organocatalytic reactions
Source: Beilstein J Org Chem. 2019 Apr 1;15:830–9. doi: 10.3762/bjoc.15.80 (PMC6466772; doi:10.3762/bjoc.15.80)
Supplement: File 2 — 2D NMR spectra of compounds 4a–d and 5a–d. [file Beilstein_J_Org_Chem-15-830-s002.pdf]

BEILSTEIN JOURNAL OF ORGANIC CHEMISTRY

Supporting Information

for

New α- and β-cyclodextrin derivatives with cinchona alkaloids used in asymmetric organocatalytic reactions

Iveta Chena Tichá, Simona Hybelbauerová and Jindřich Jindřich

Beilstein J. Org. Chem. **2019,** *15,* 830–839. doi:10.3762/bjoc.15.80

2D NMR spectra of compounds 4a–d and 5a–d

License and Terms: This is a supporting information file under the terms of the Creative Commons Attribution License (http://creativecommons.org/licenses/by/4.0). Please note that the reuse, redistribution and reproduction in particular requires that the authors and source are credited.
The license is subject to the *Beilstein Journal of Organic Chemistry* terms and conditions: (https://www.beilstein-journals.org/bjoc)

Table of contents

2D NMR spectra of prepared CD derivatives **4a–d**, **5a–d** S2

2D NMR spectra of prepared CD derivatives 4a–d, 5a–d

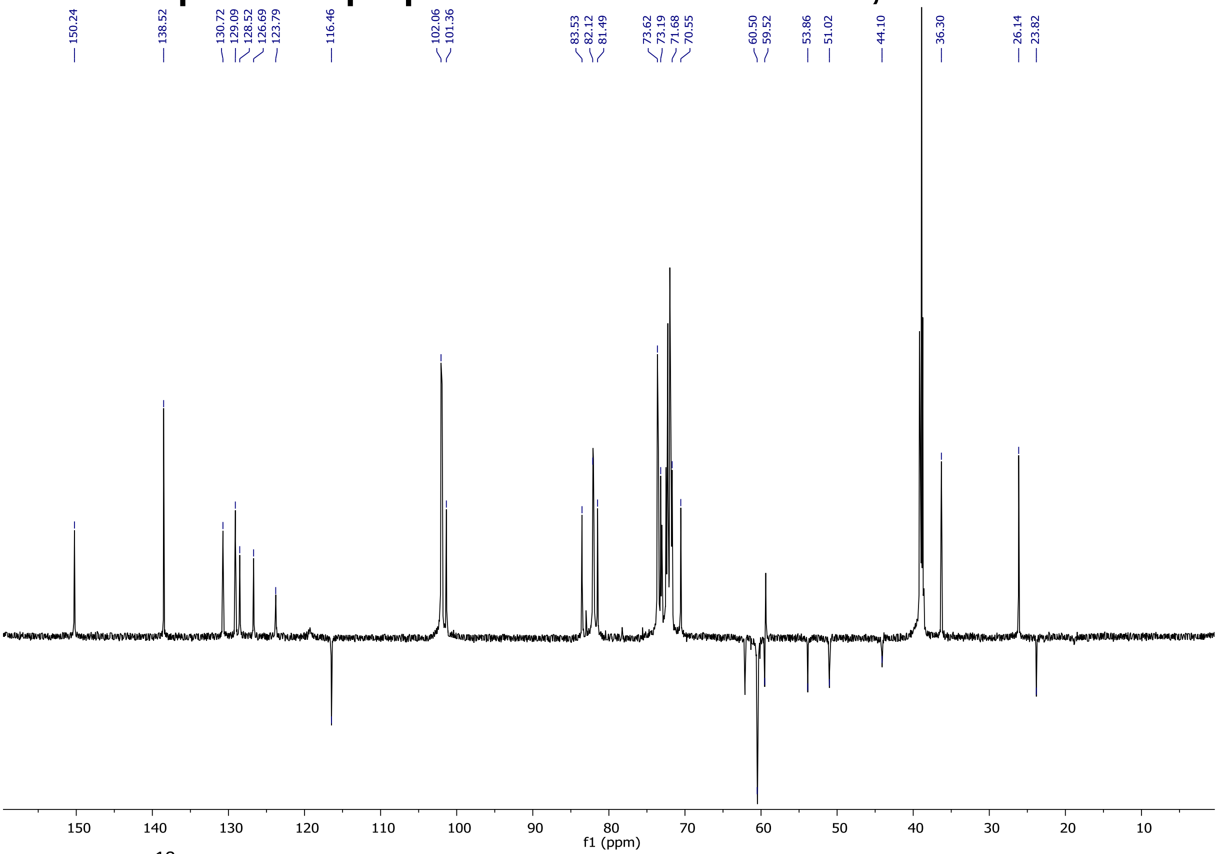

Figure S1: ^{13}C DEPT spectrum of compound **4a**.

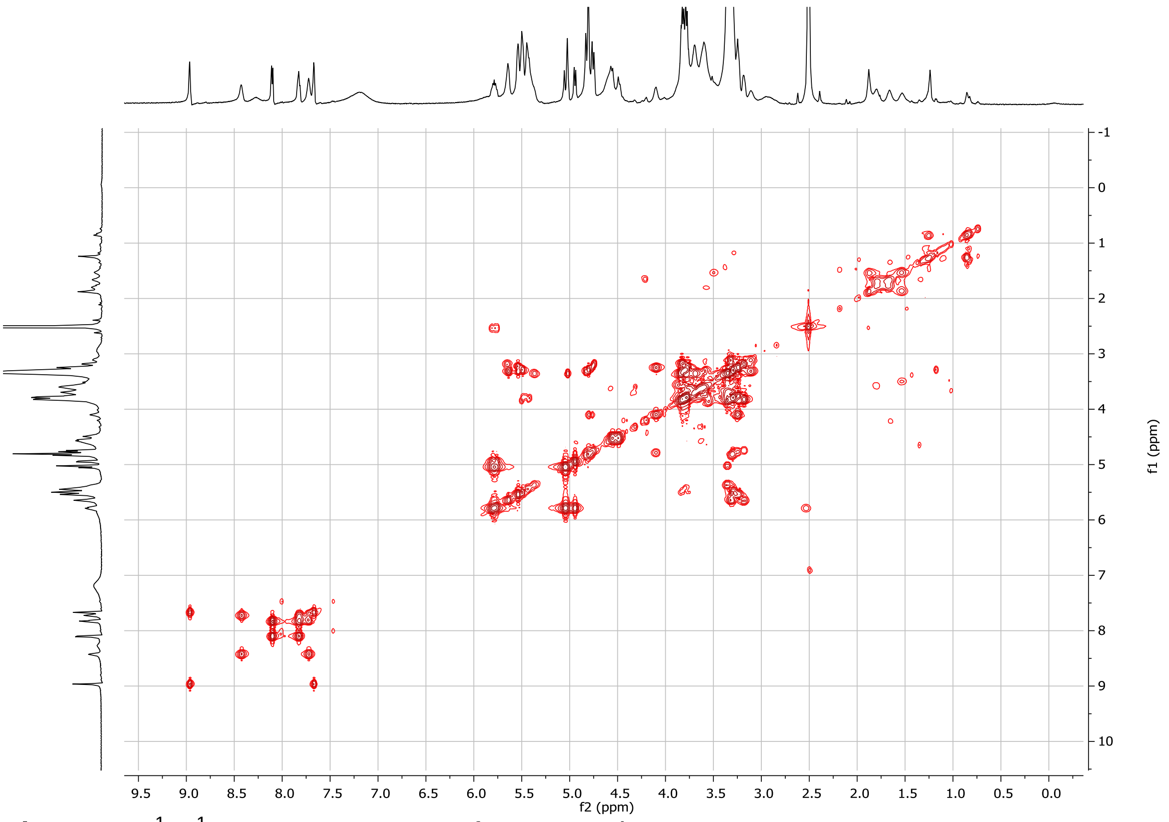

Figure S2: ^{1}H-^{1}H COSY spectrum of compound **4a**.

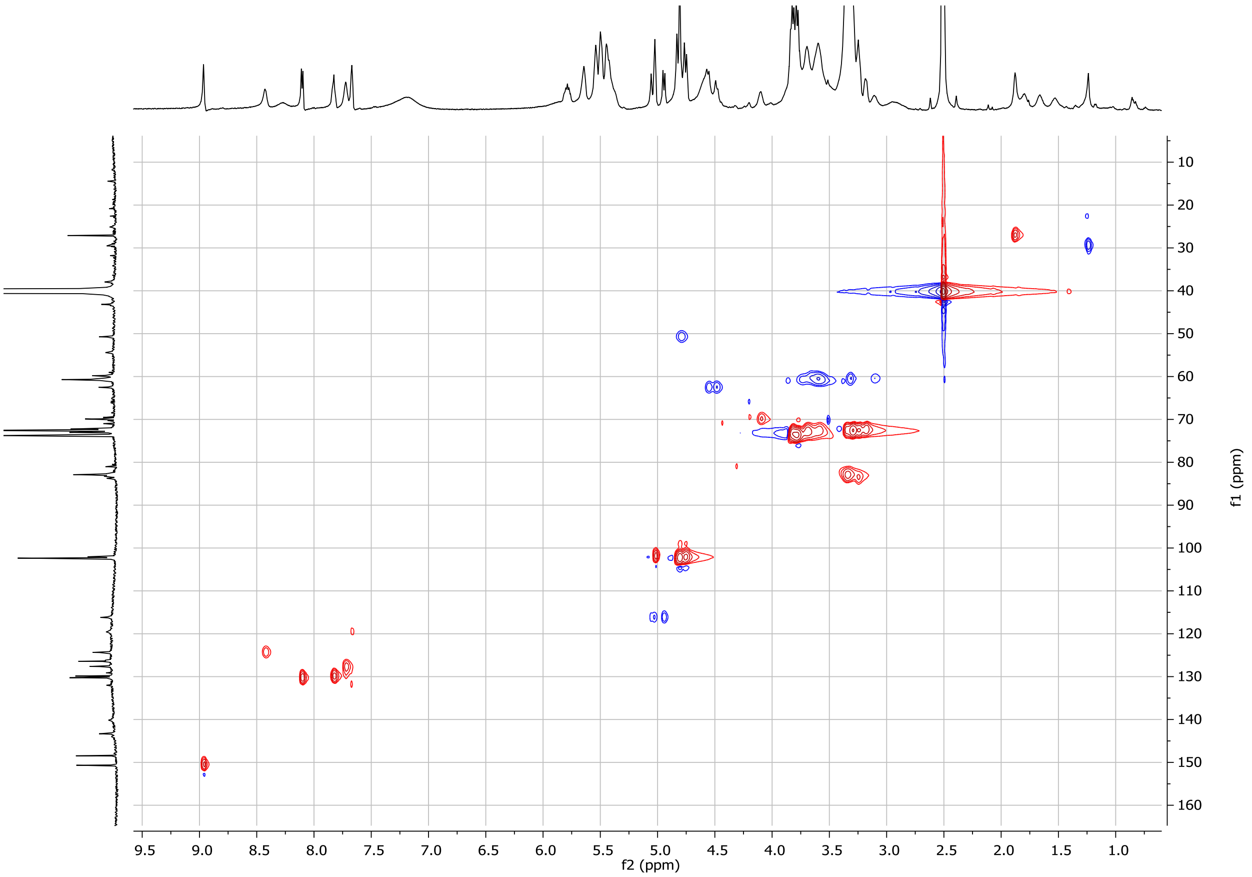

Figure S3: HSQC spectrum of compound **4a**.

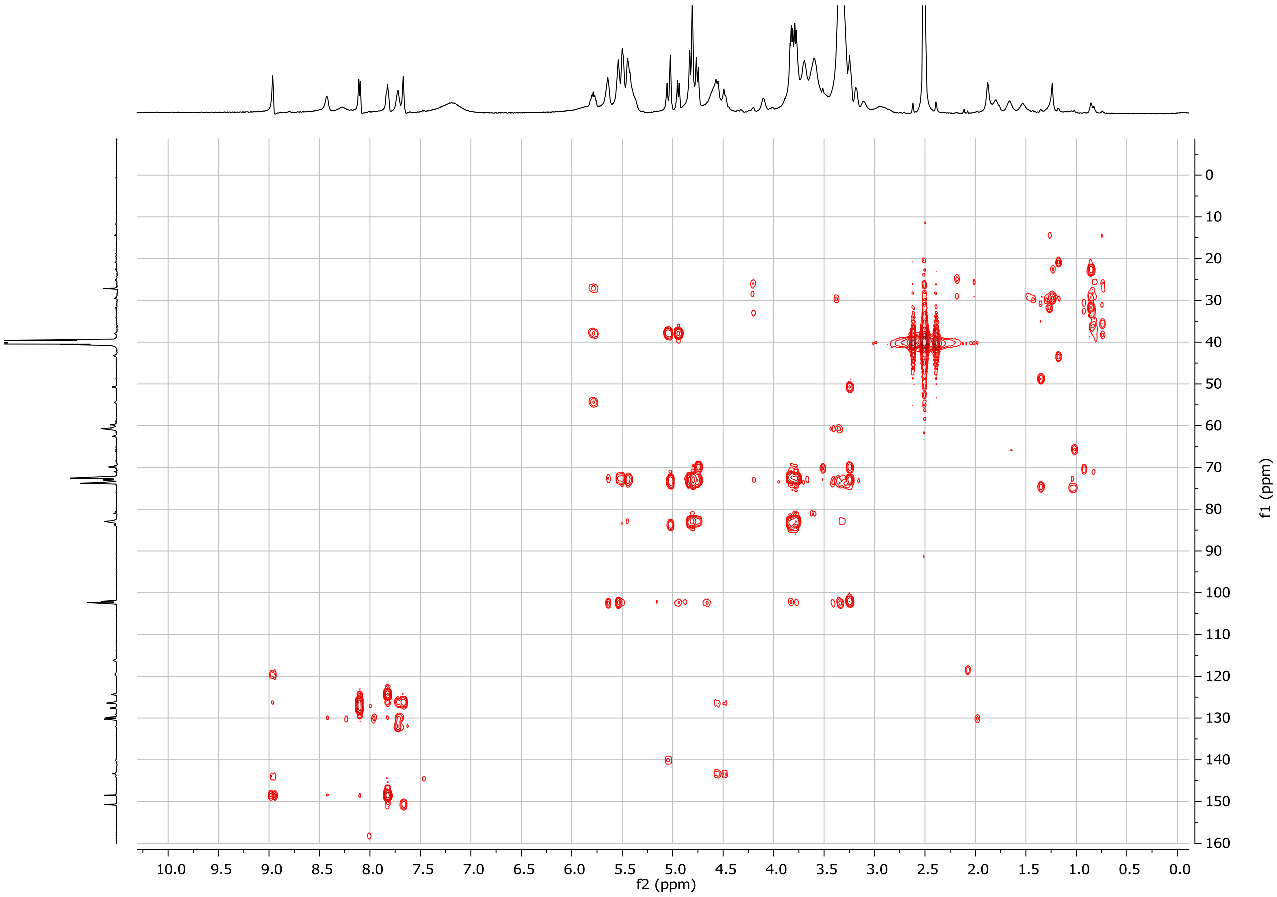

Figure S4: HMBC spectrum of compound **4a**.

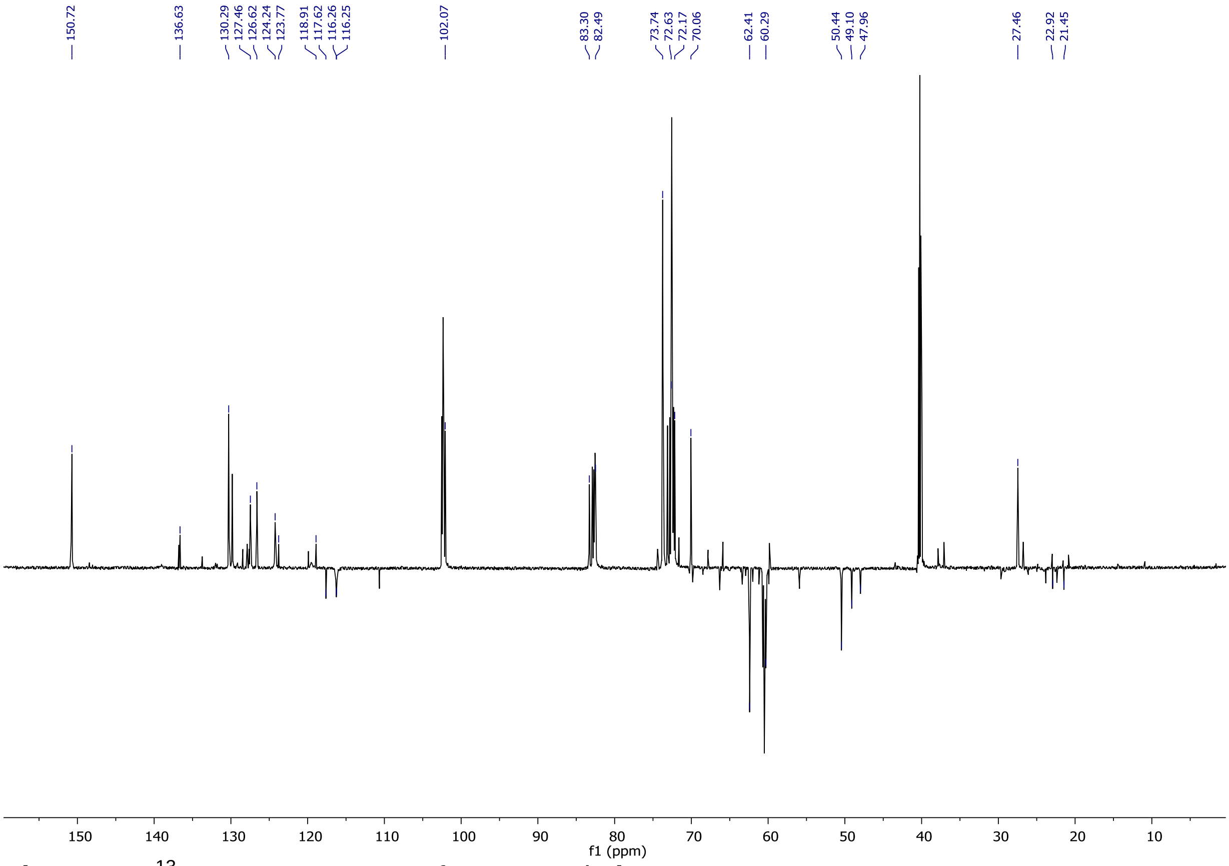

Figure S5: ^{13}C DEPT spectrum of compound **4b**.

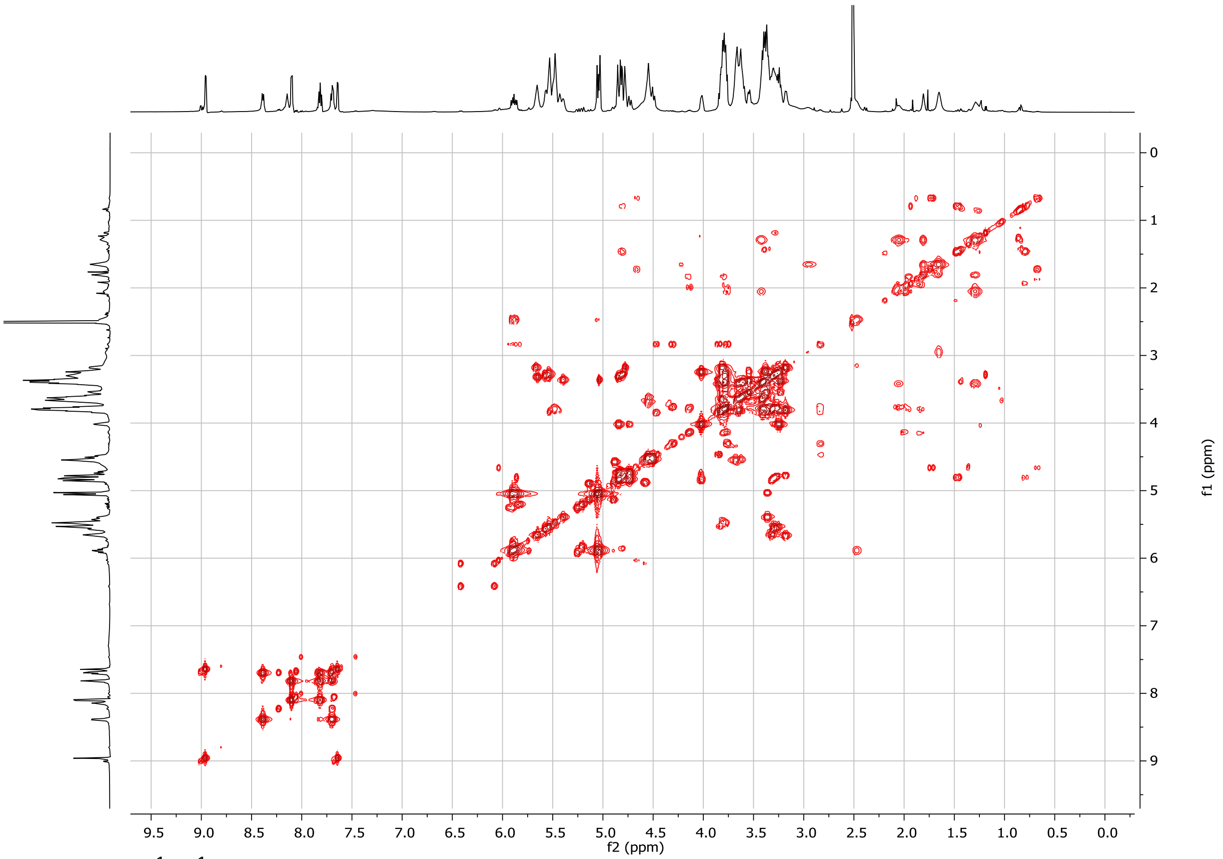

Figure S6: ^{1}H-^{1}H COSY spectrum of compound **4b**.

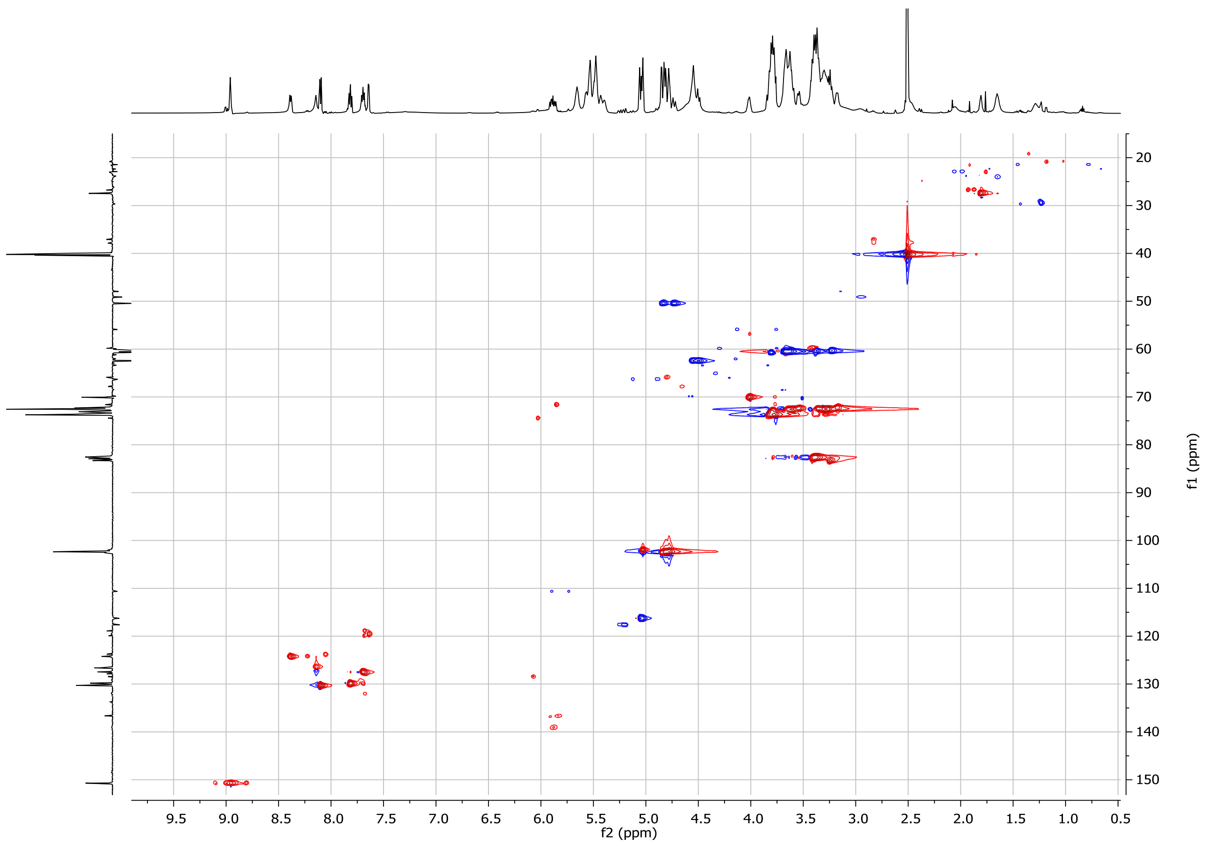

Figure S7: HSQC spectrum of compound **4b**.

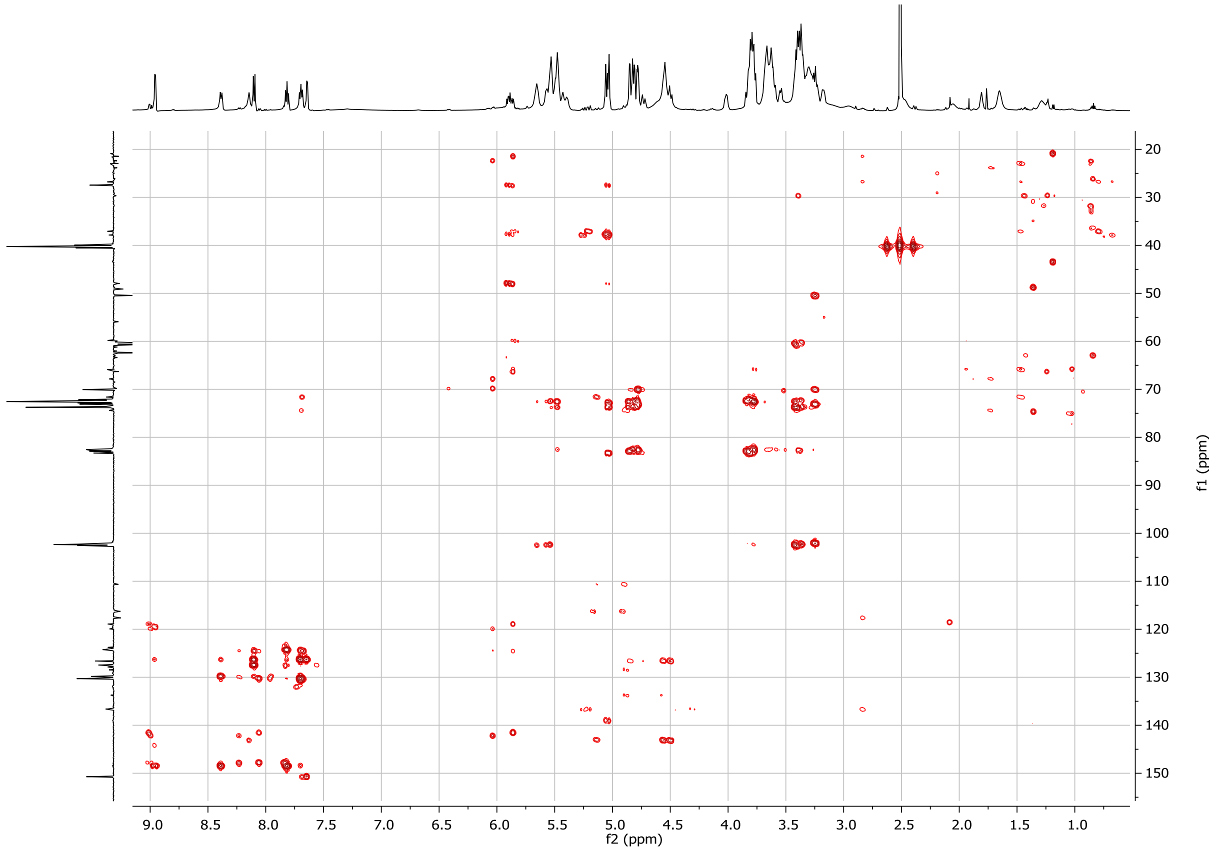

Figure S8: HMBC spectrum of compound **4b**.

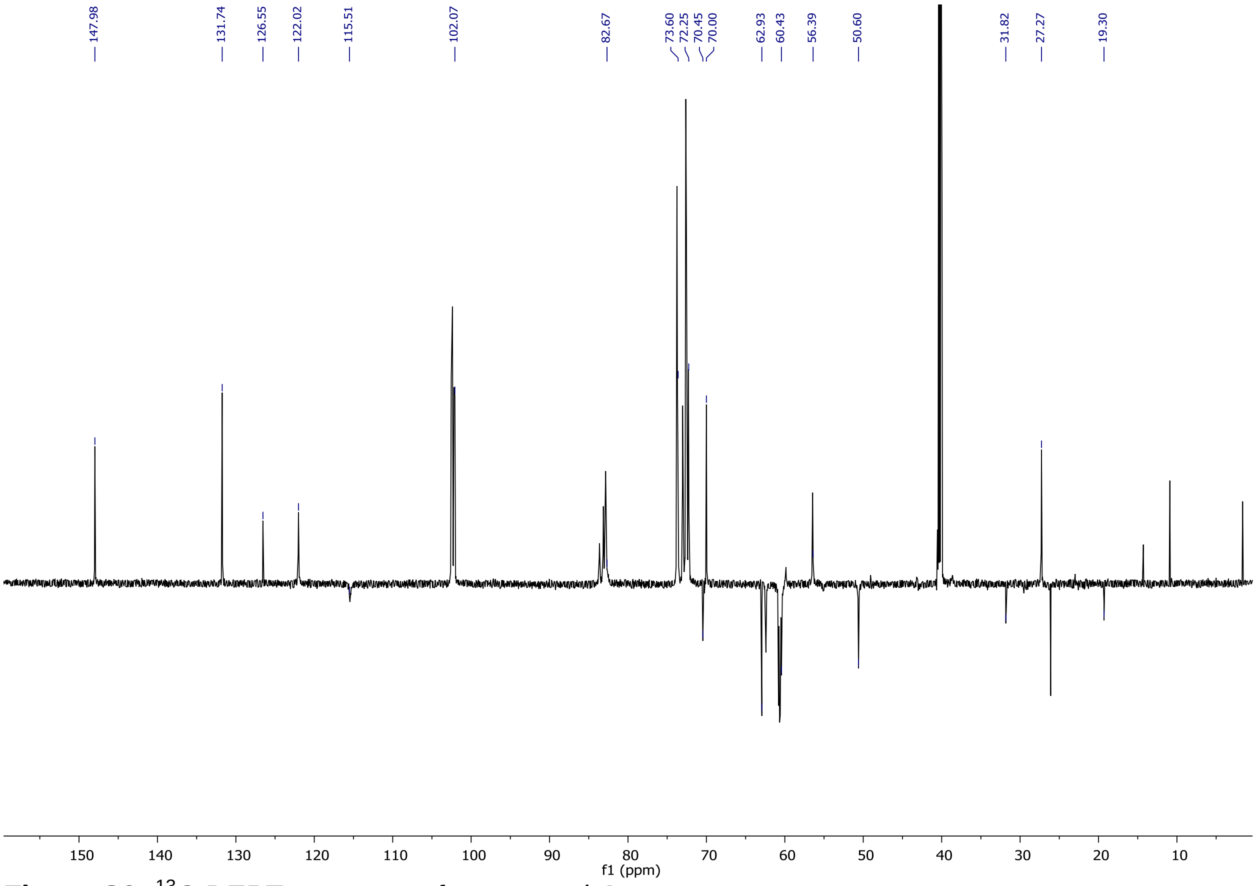

Figure S9: ^{13}C DEPT spectrum of compound **4c**.

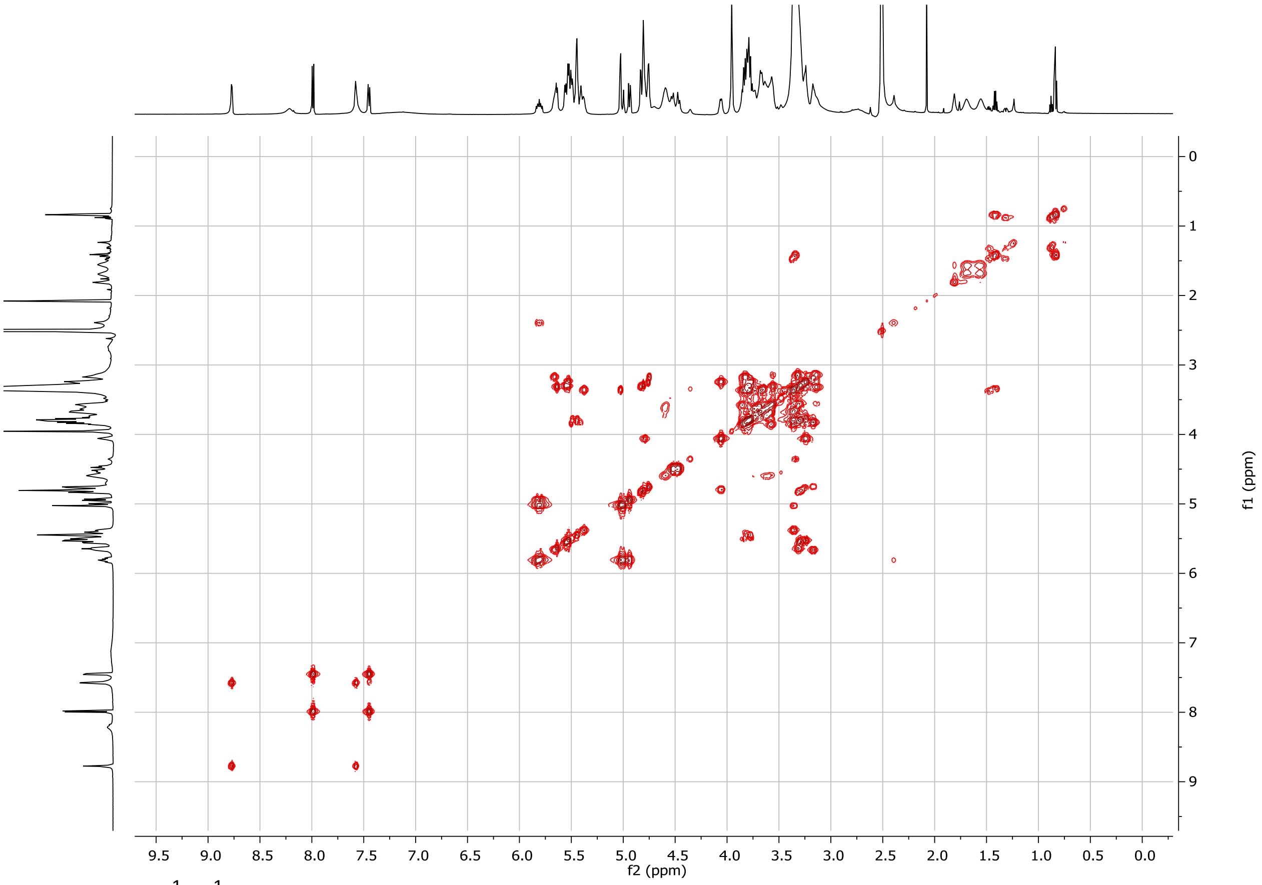

Figure S10: ^{1}H-^{1}H COSY spectrum of compound **4c**.

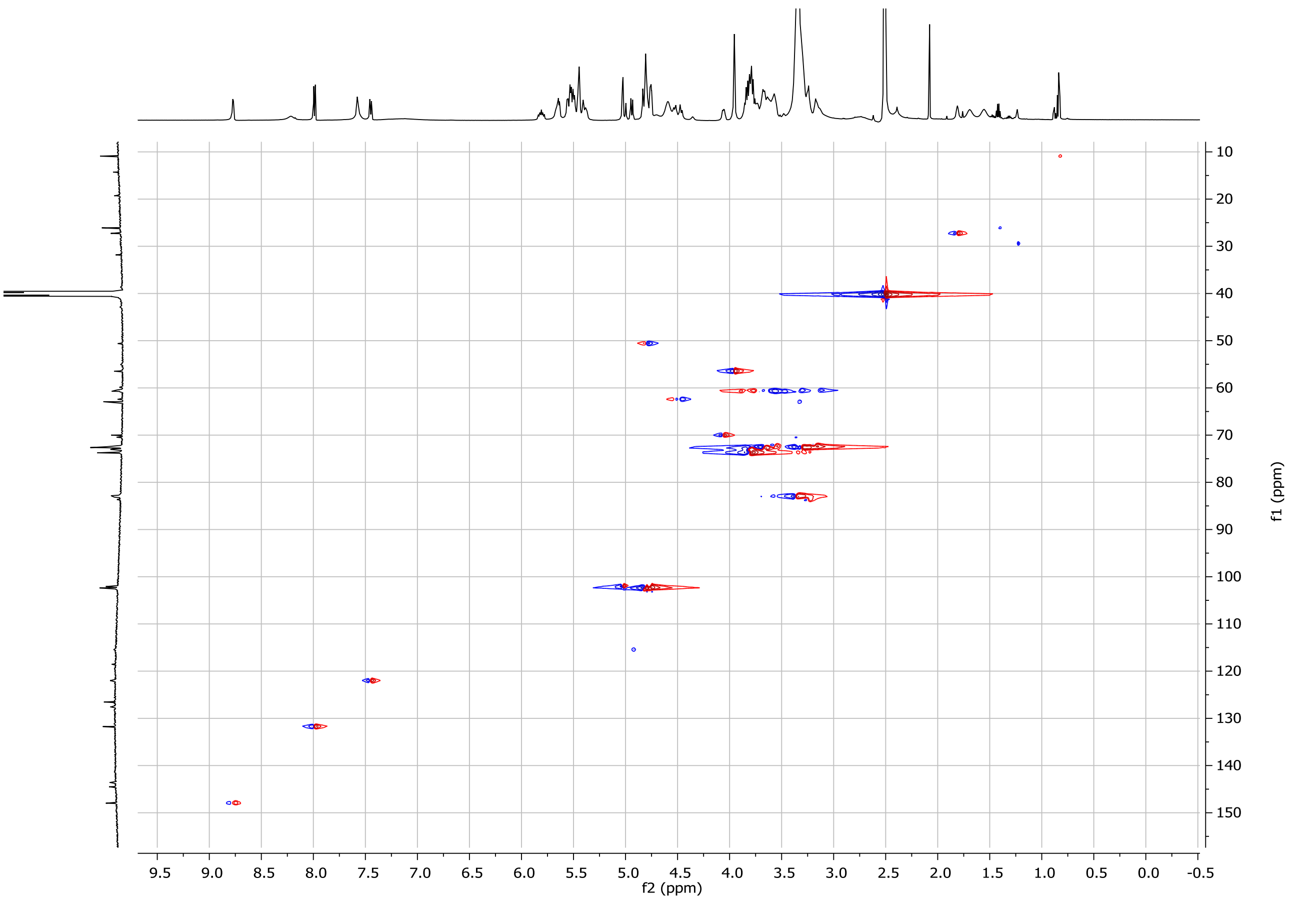

Figure S11: HSQC spectrum of compound **4c**.

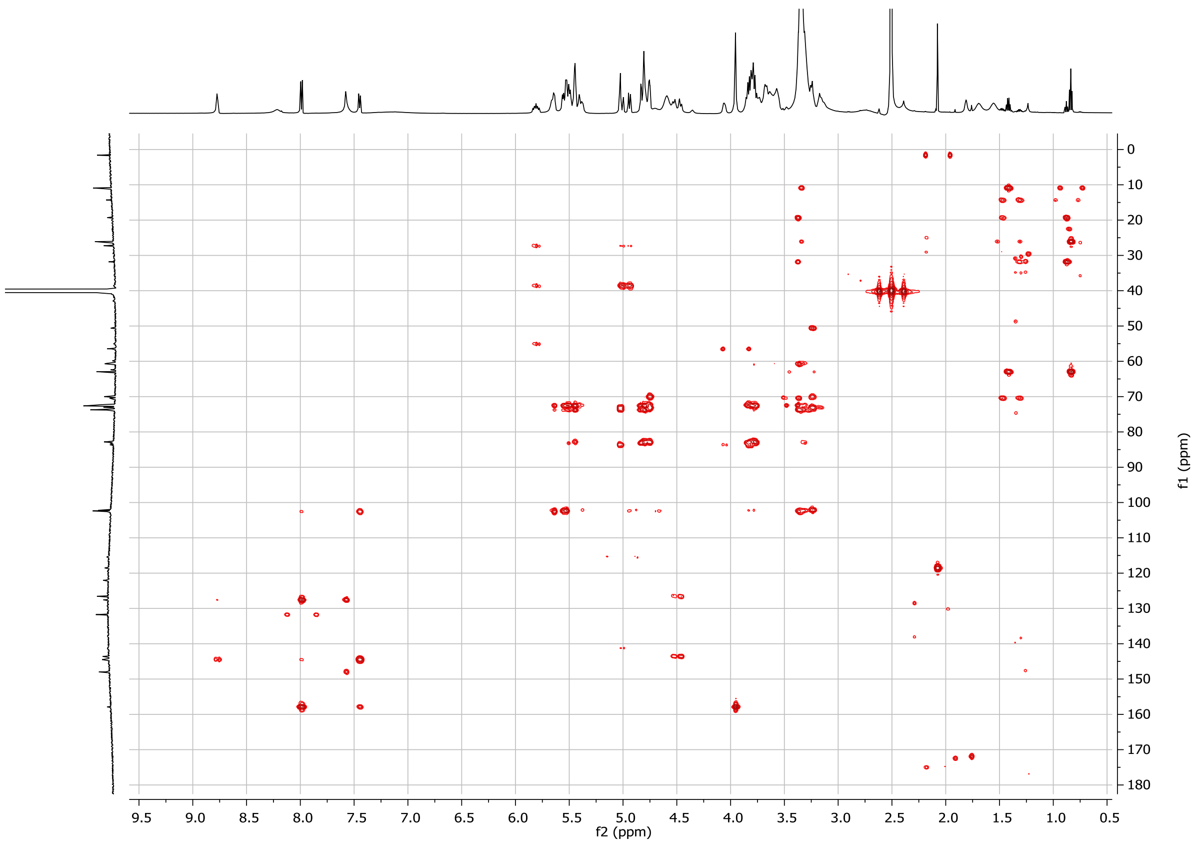

Figure S12: HMBC spectrum of compound **4c**.

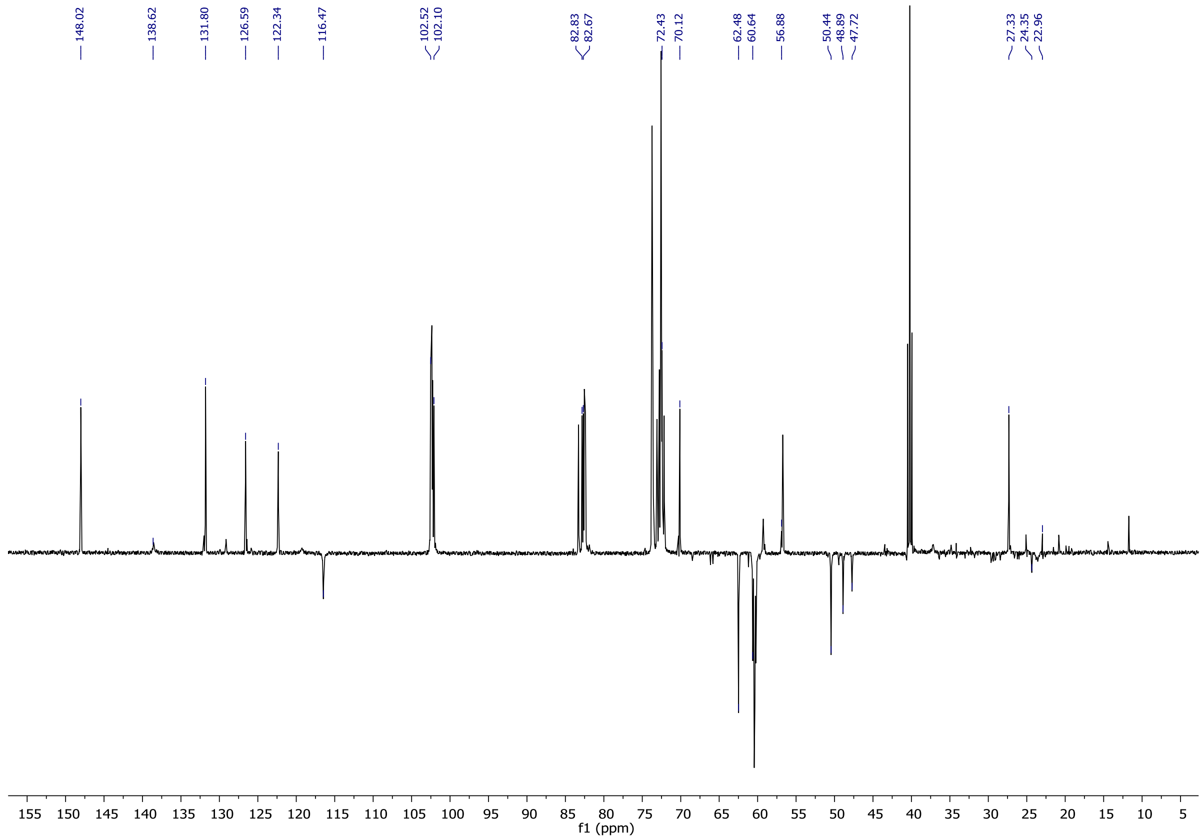

Figure S13: ^{13}C DEPT spectrum of compound **4d**.

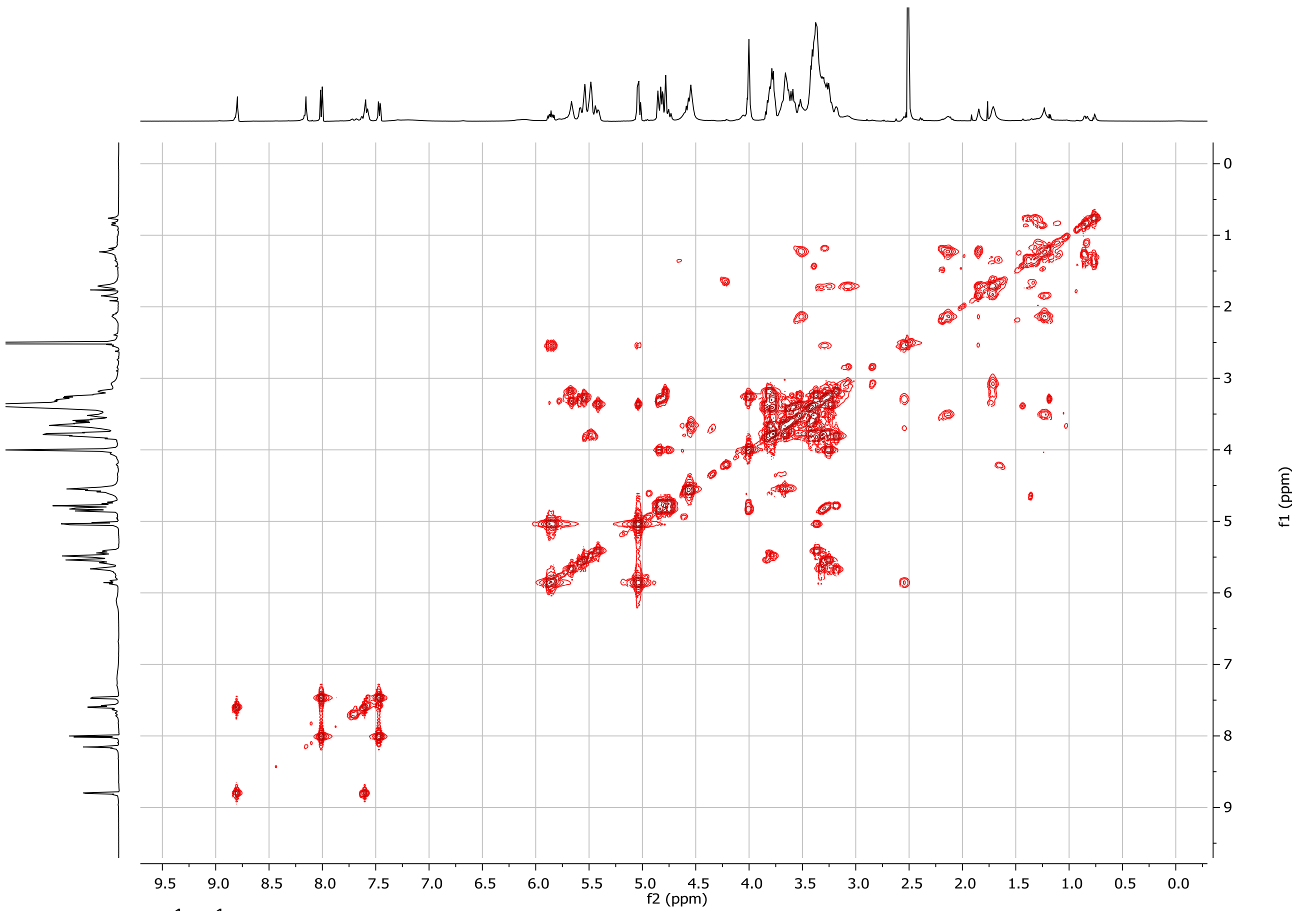

Figure S14: ^{1}H-^{1}H COSY spectrum of compound **4d**.

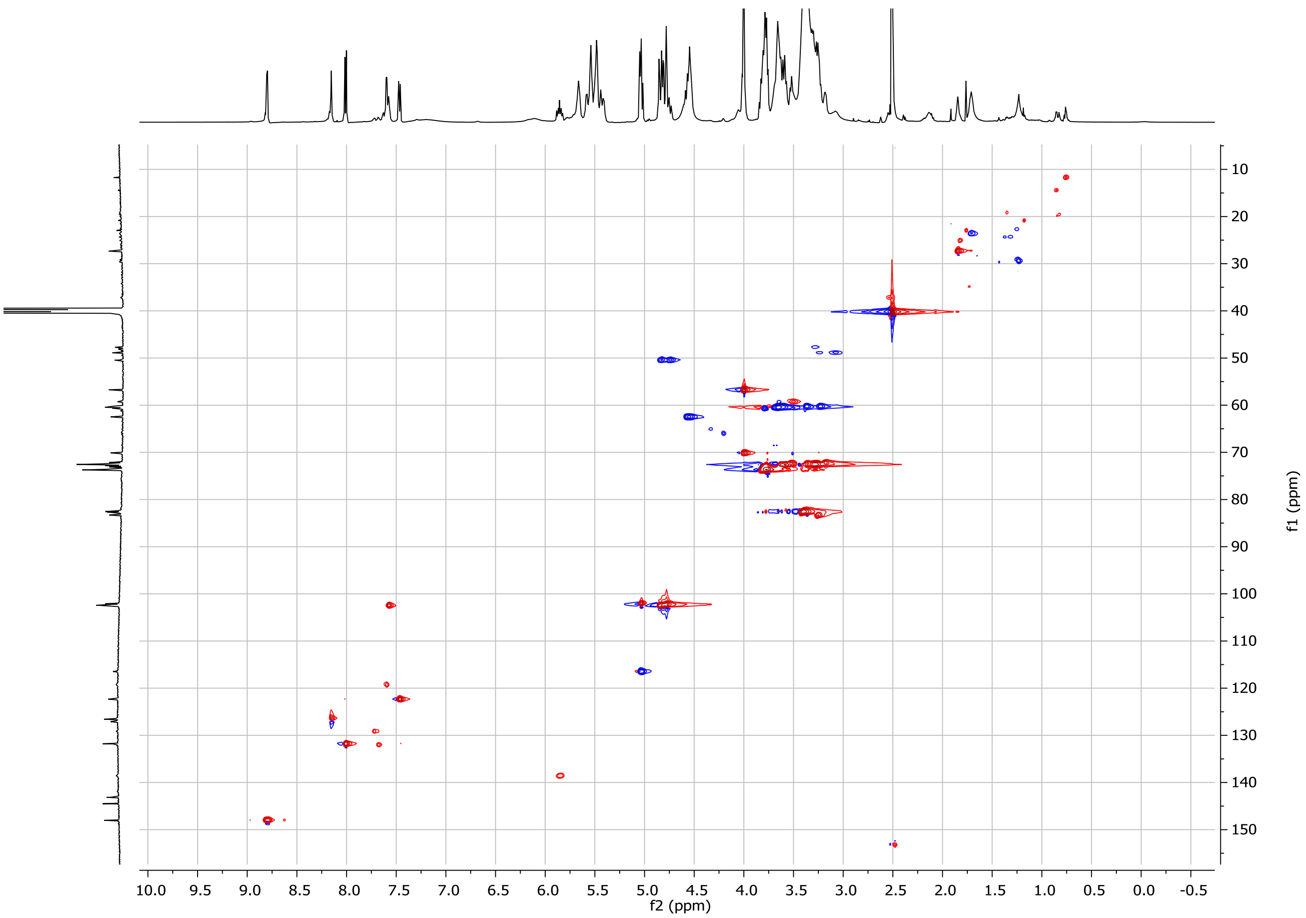

Figure S15: HSQC spectrum of compound **4d**.

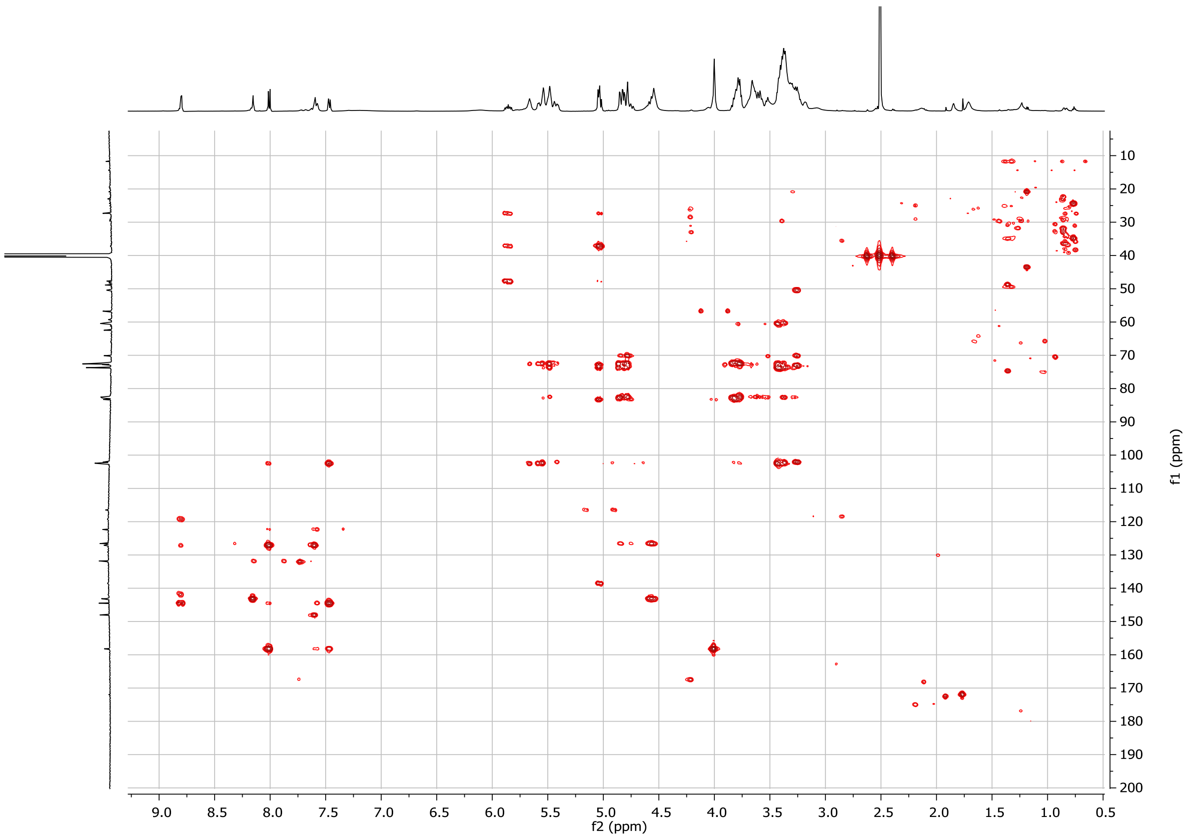

Figure S16: HMBC spectrum of compound **4d**.

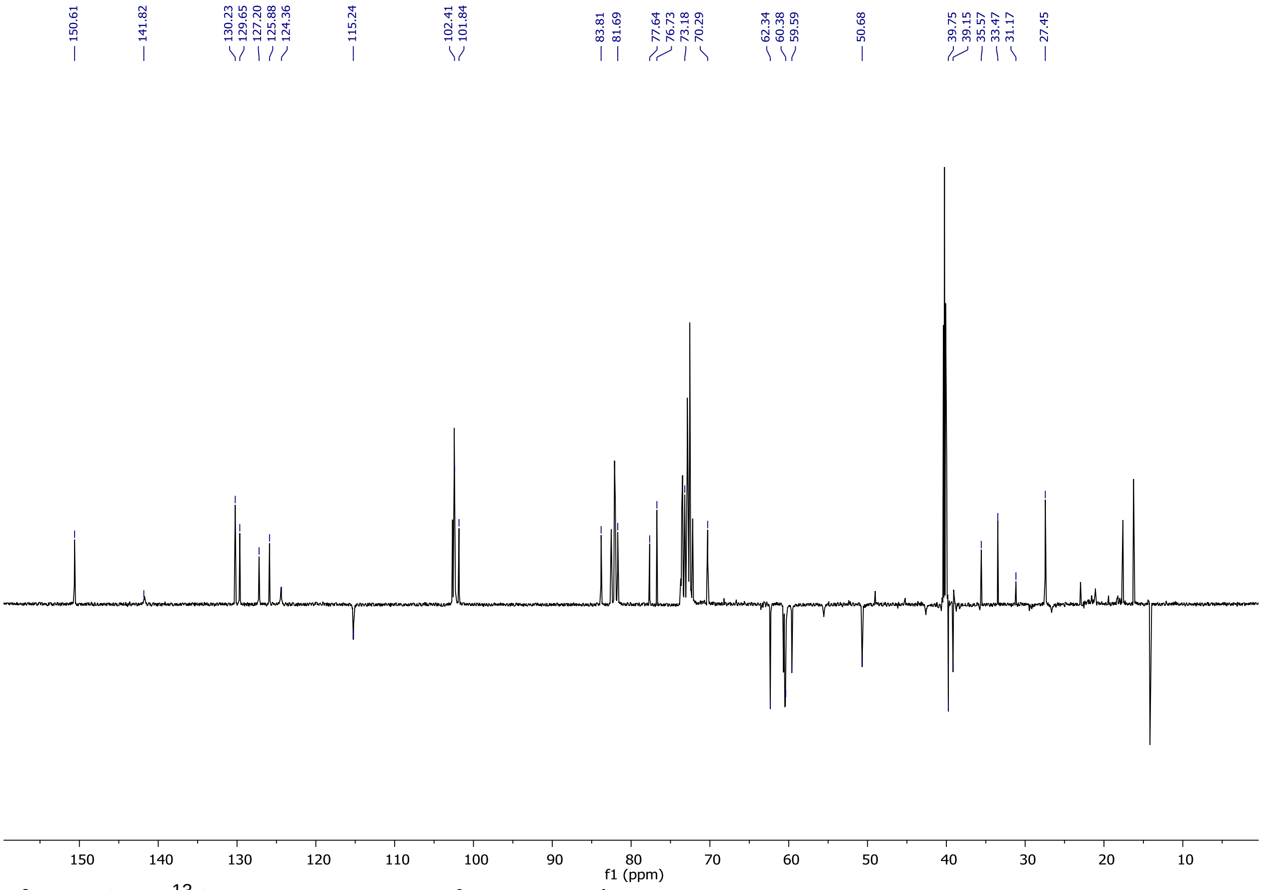

Figure S17: ^{13}C DEPT spectrum of compound **5a**.

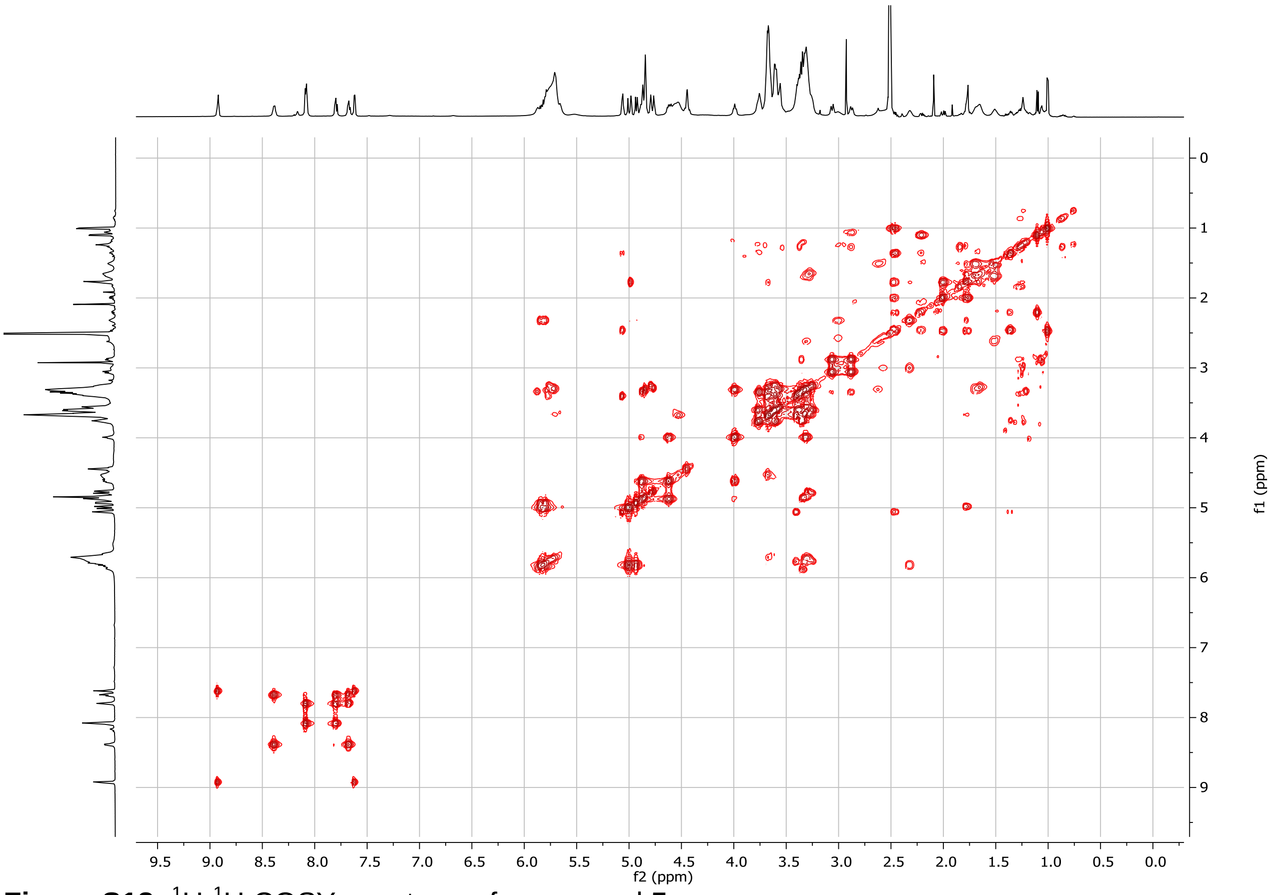

Figure S18: ^{1}H-^{1}H COSY spectrum of compound **5a**.

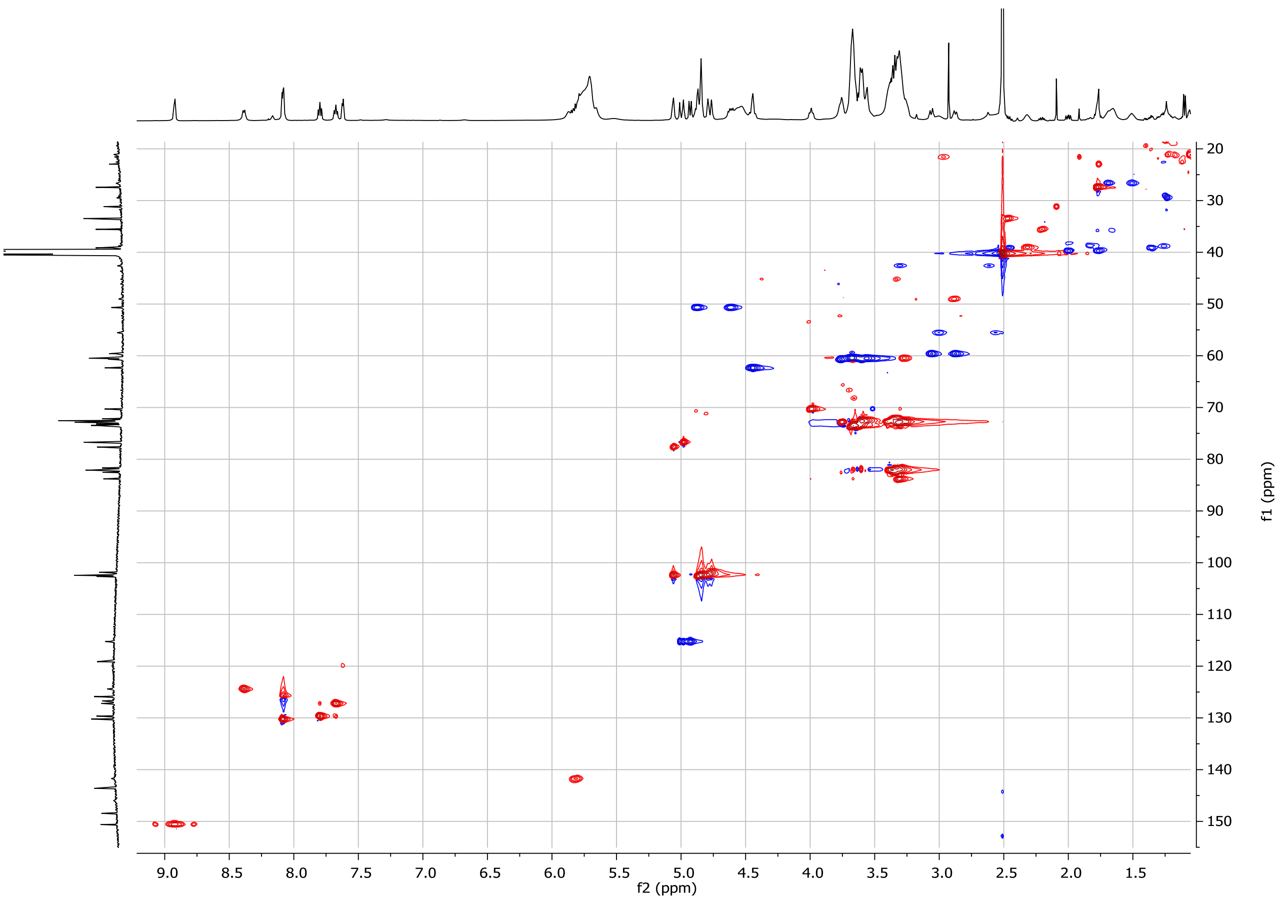

Figure S19: HSQC spectrum of compound **5a**.

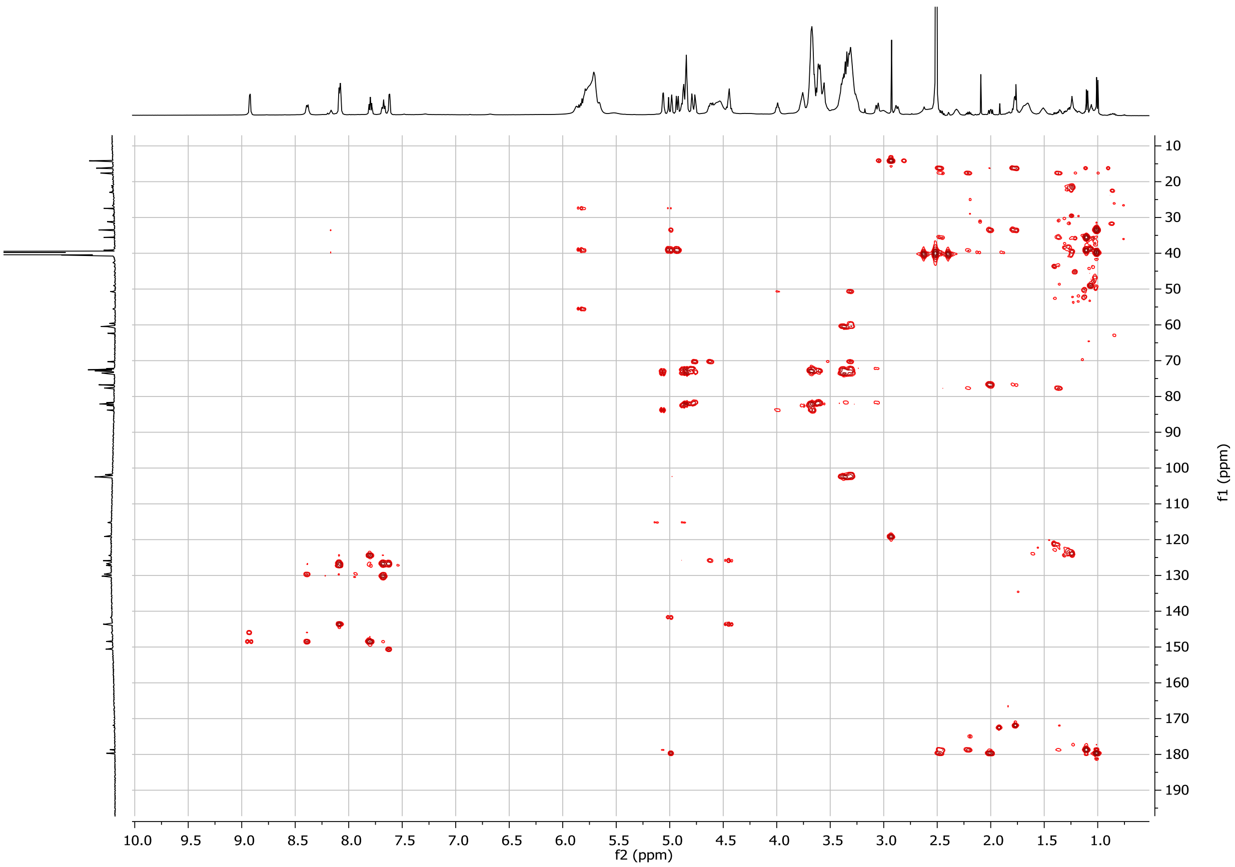

Figure S20: HMBC spectrum of compound **5a**.

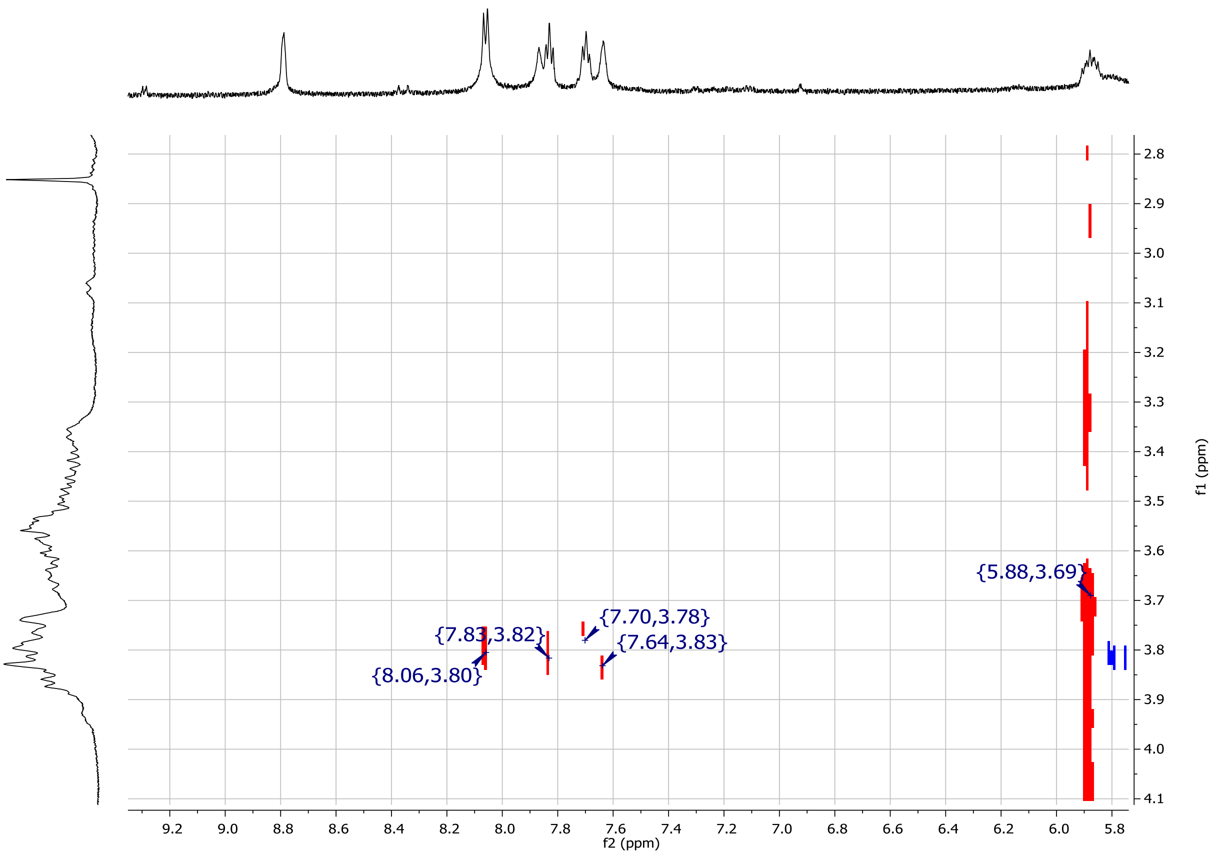

Figure **S21**: The part of 2D ROESY spectrum of compound **5a**.

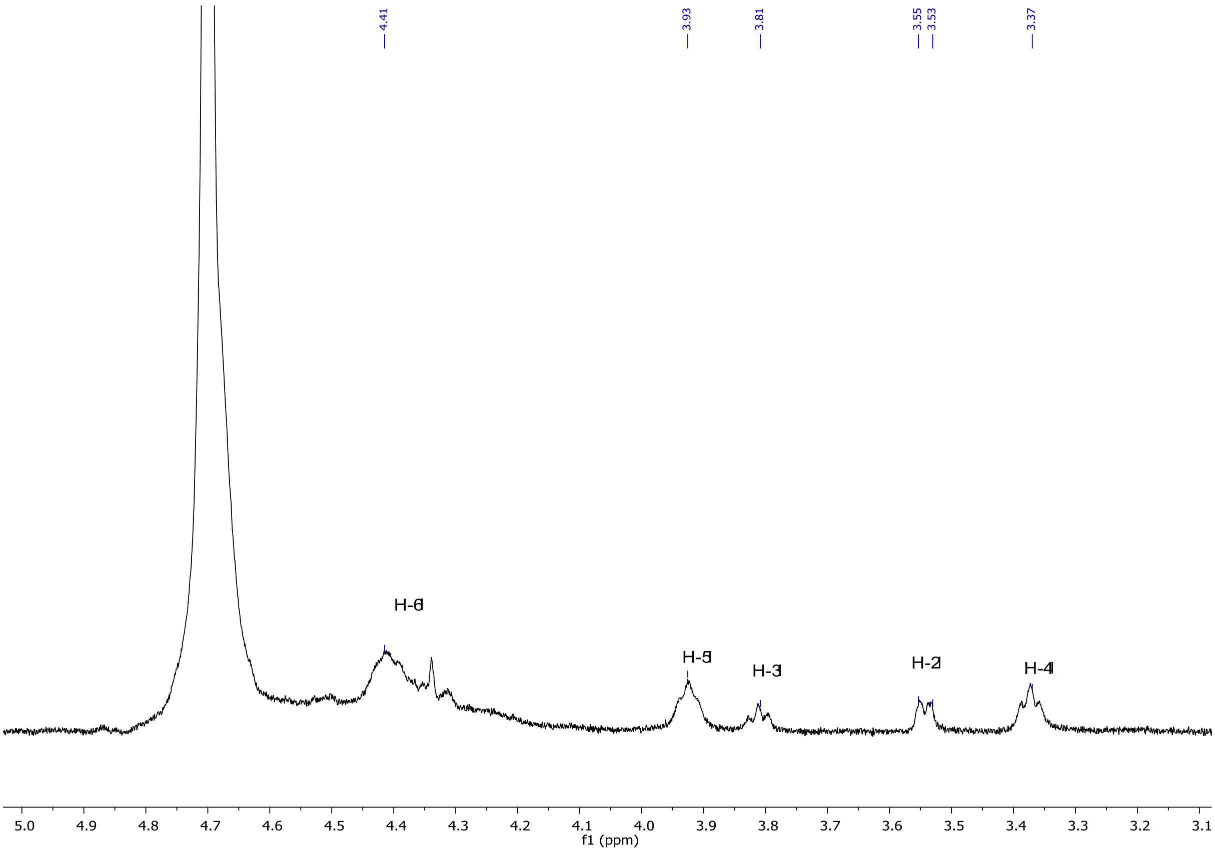

Figure **S22**: 1D TOCSY spectrum of compound **5a**.

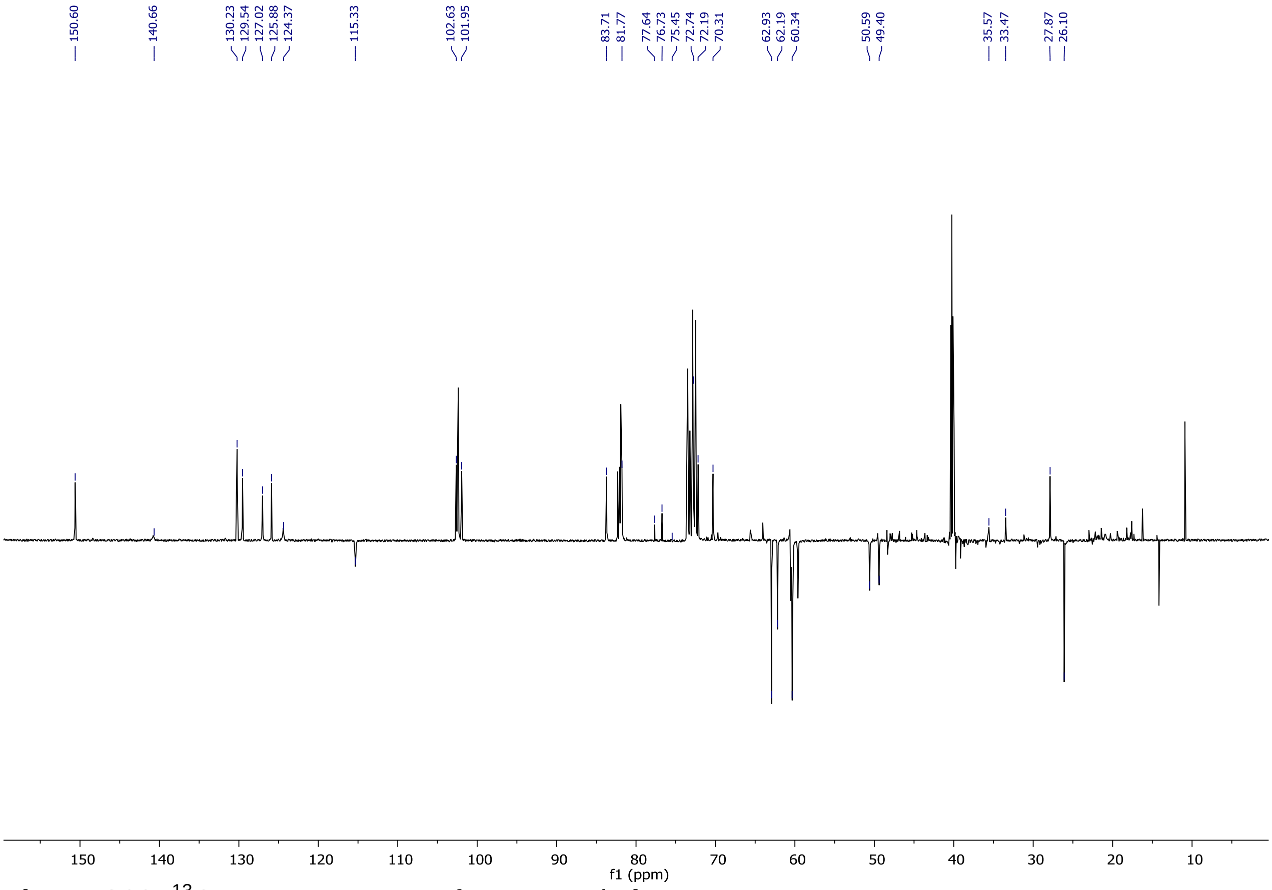

Figure S23: ^{13}C DEPT spectrum of compound **5b**.

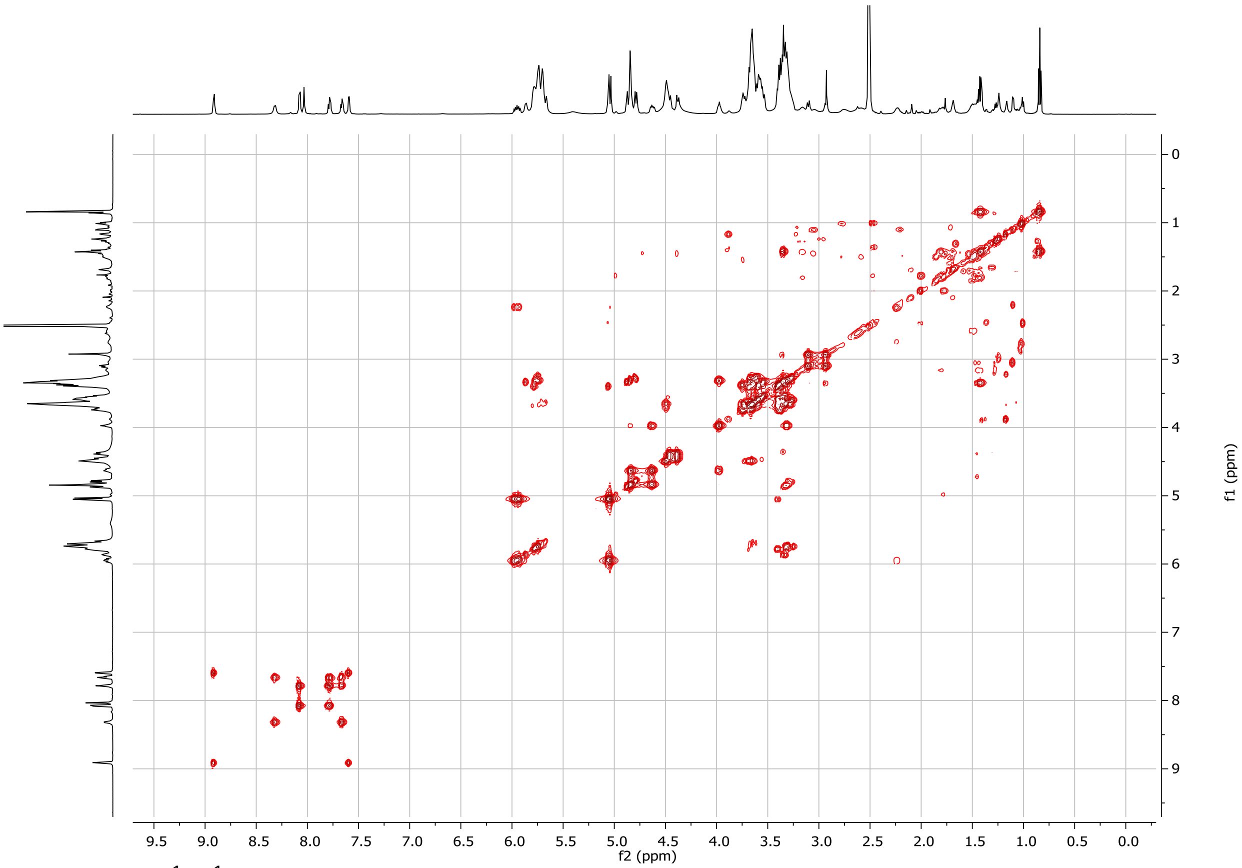

Figure S24: ^{1}H-^{1}H COSY spectrum of compound **5b**.

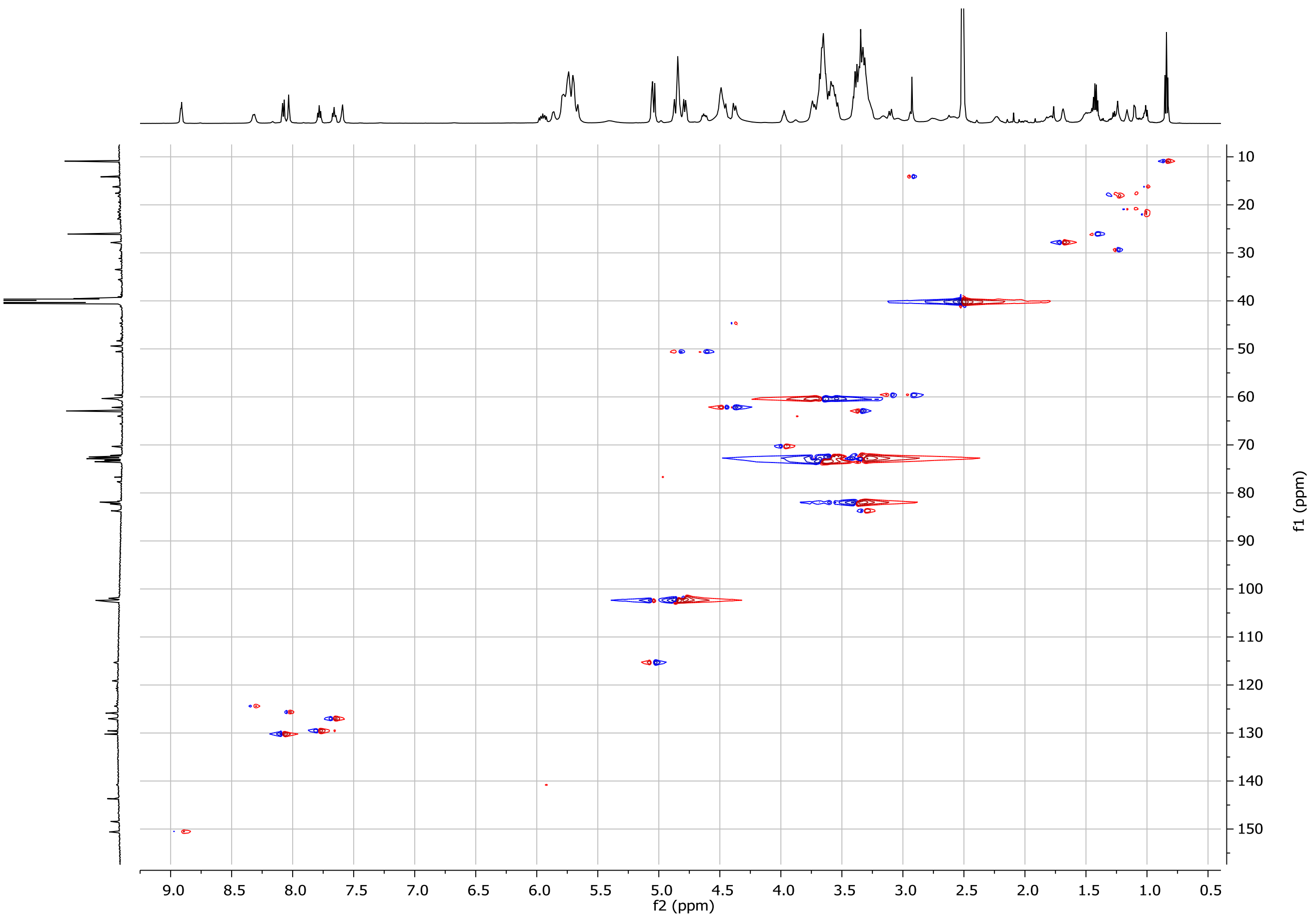

Figure S25: HSQC spectrum of compound **5b**.

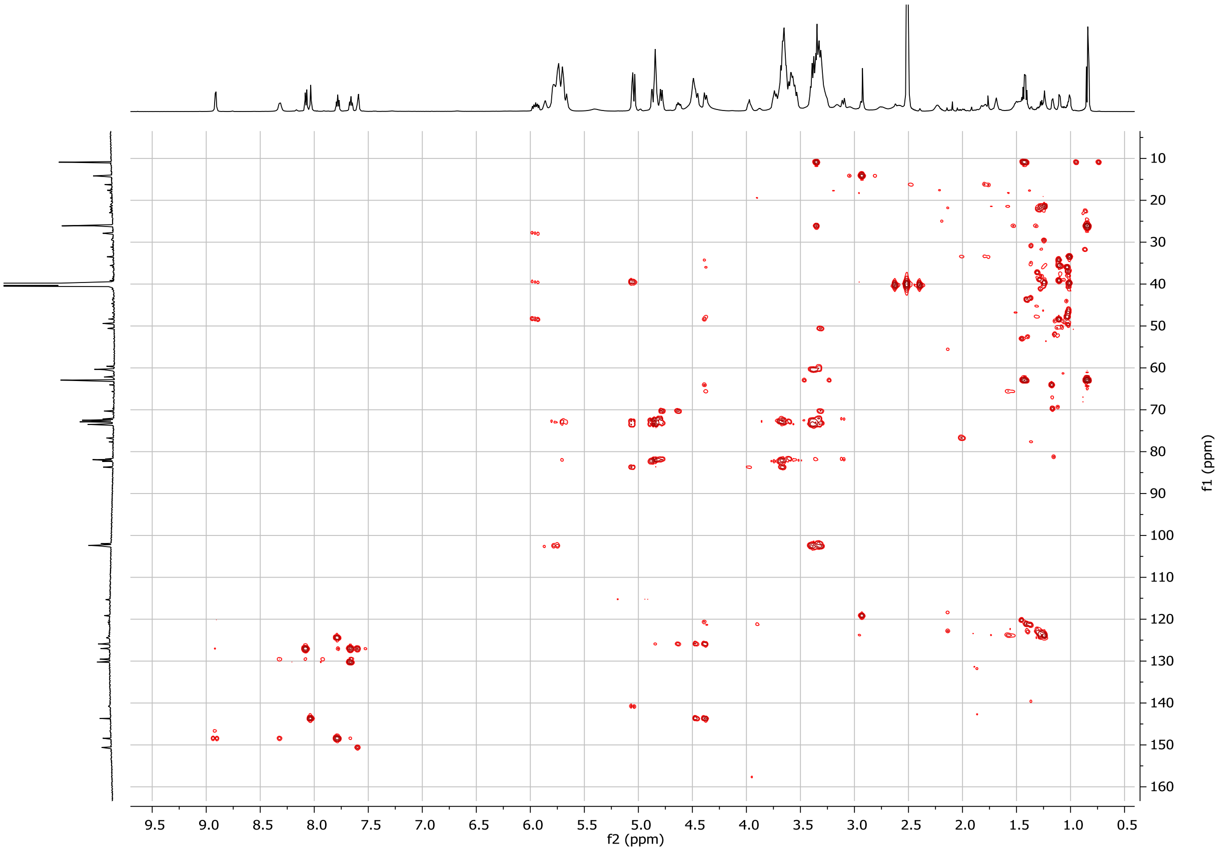

Figure S26: HMBC spectrum of compound **5b**.

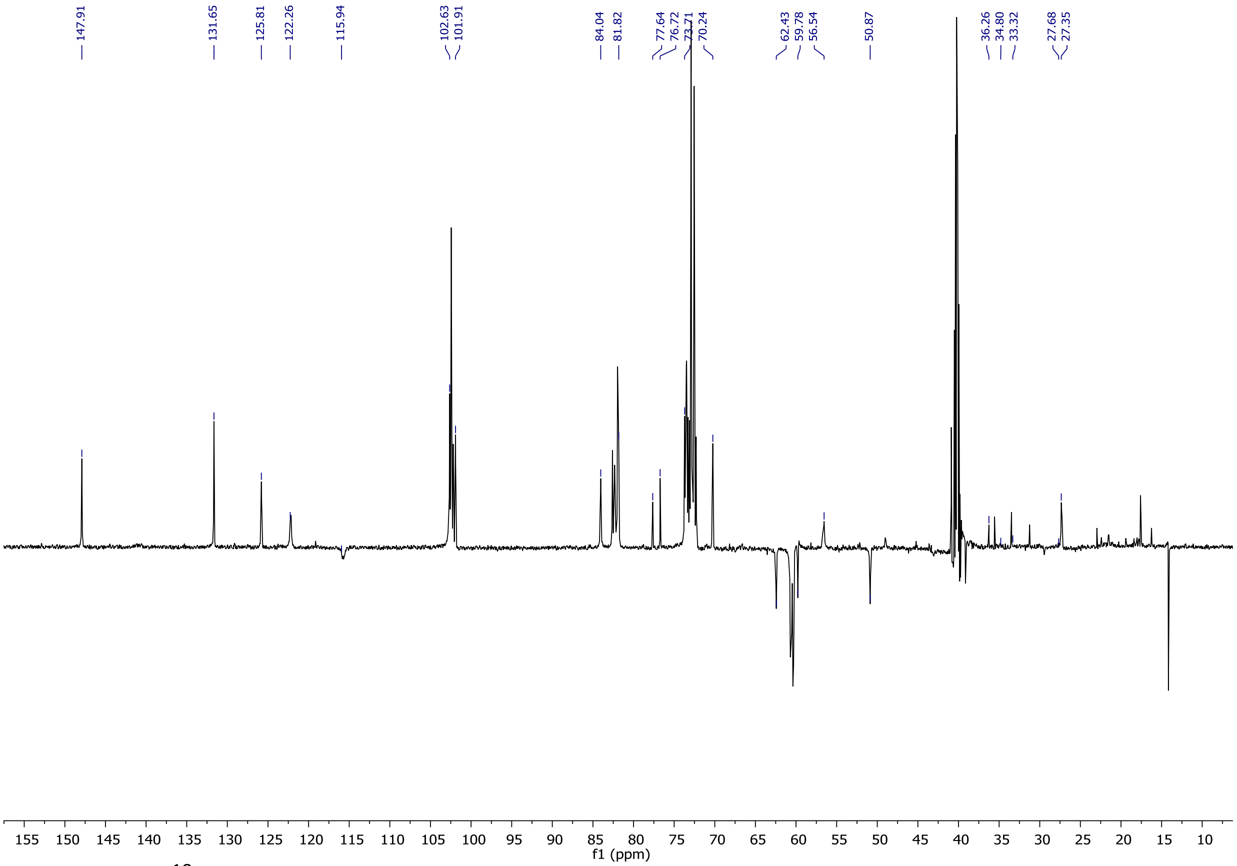

Figure S27: ^{13}C DEPT spectrum of compound **5c**.

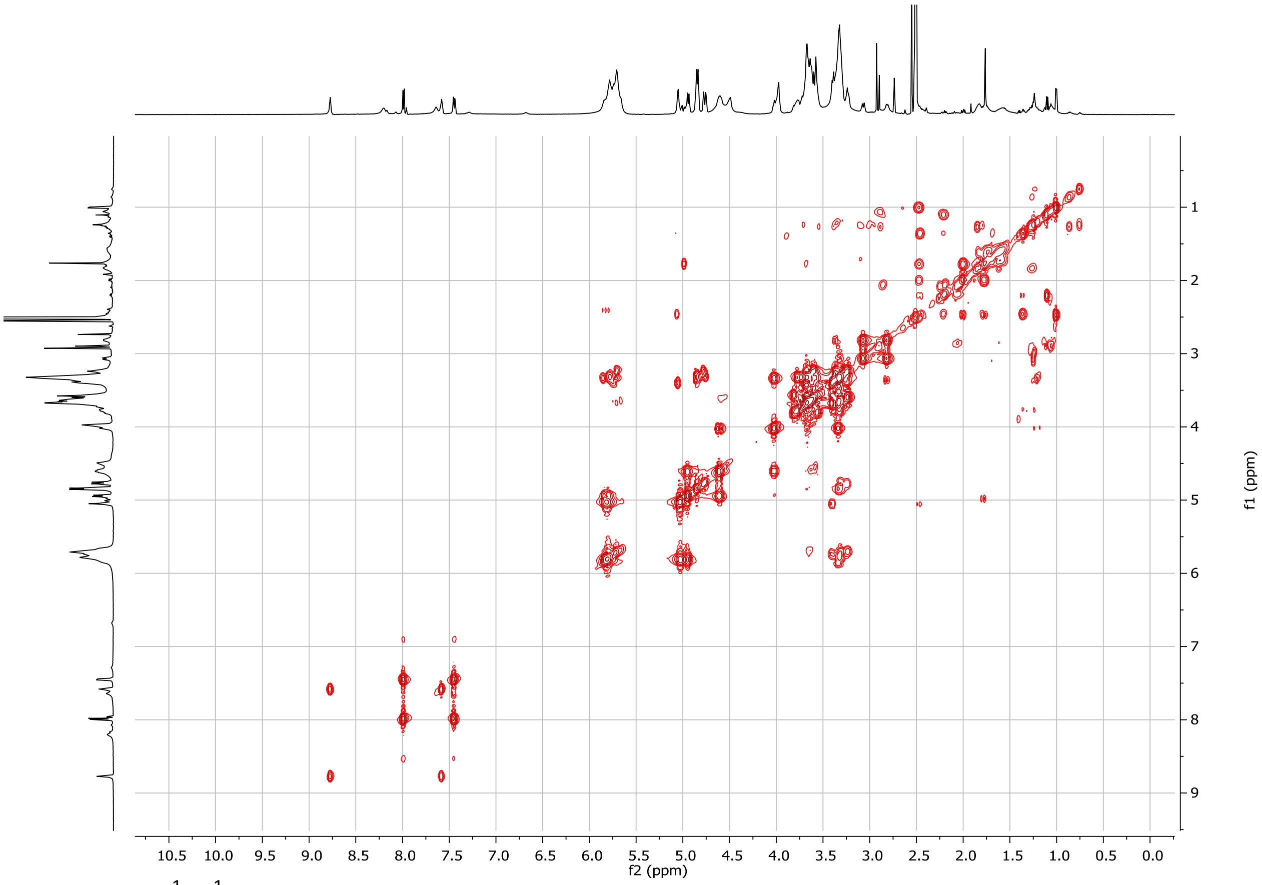

Figure S28: ^{1}H-^{1}H COSY spectrum of compound **5c**.

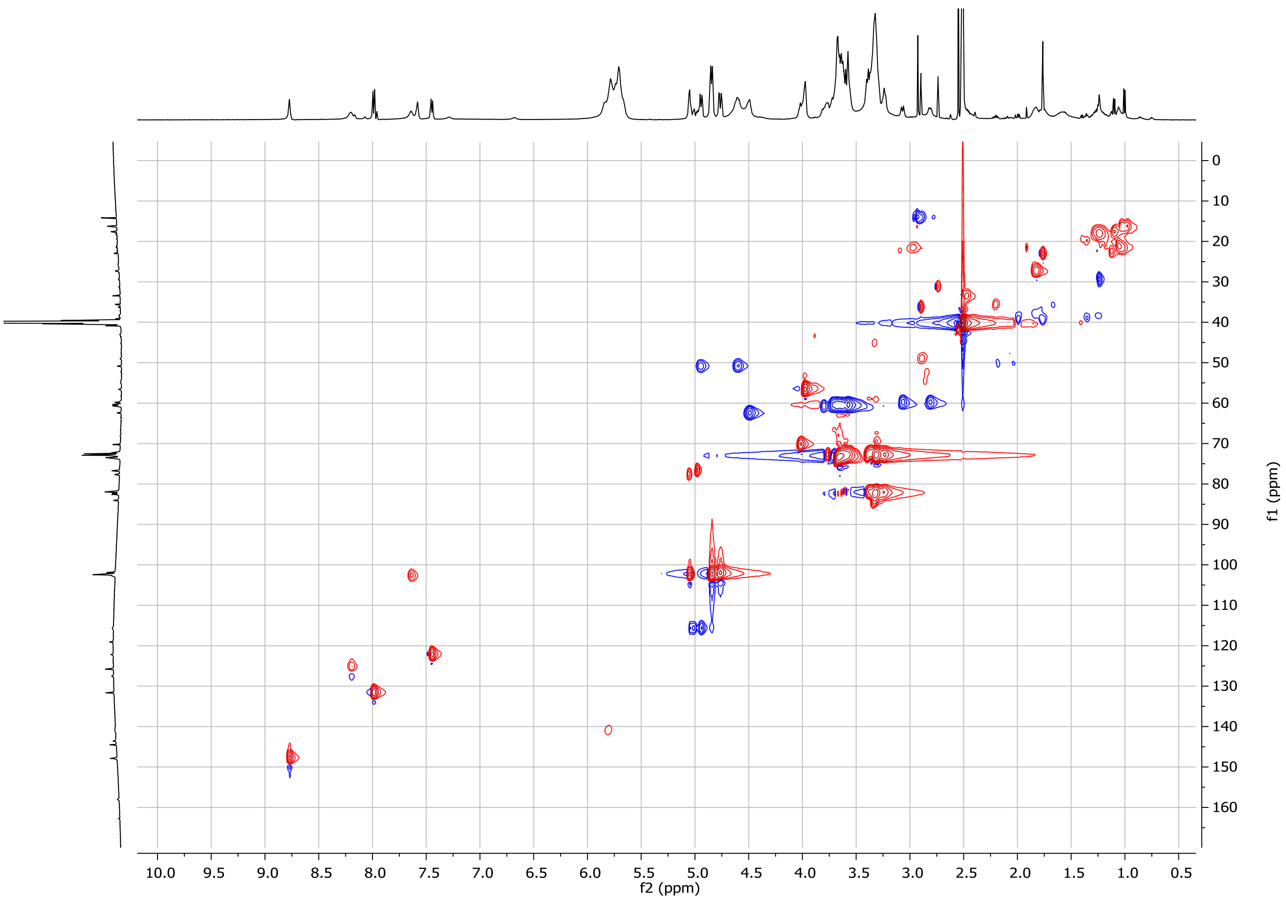

Figure S29: HSQC spectrum of compound **5c**.

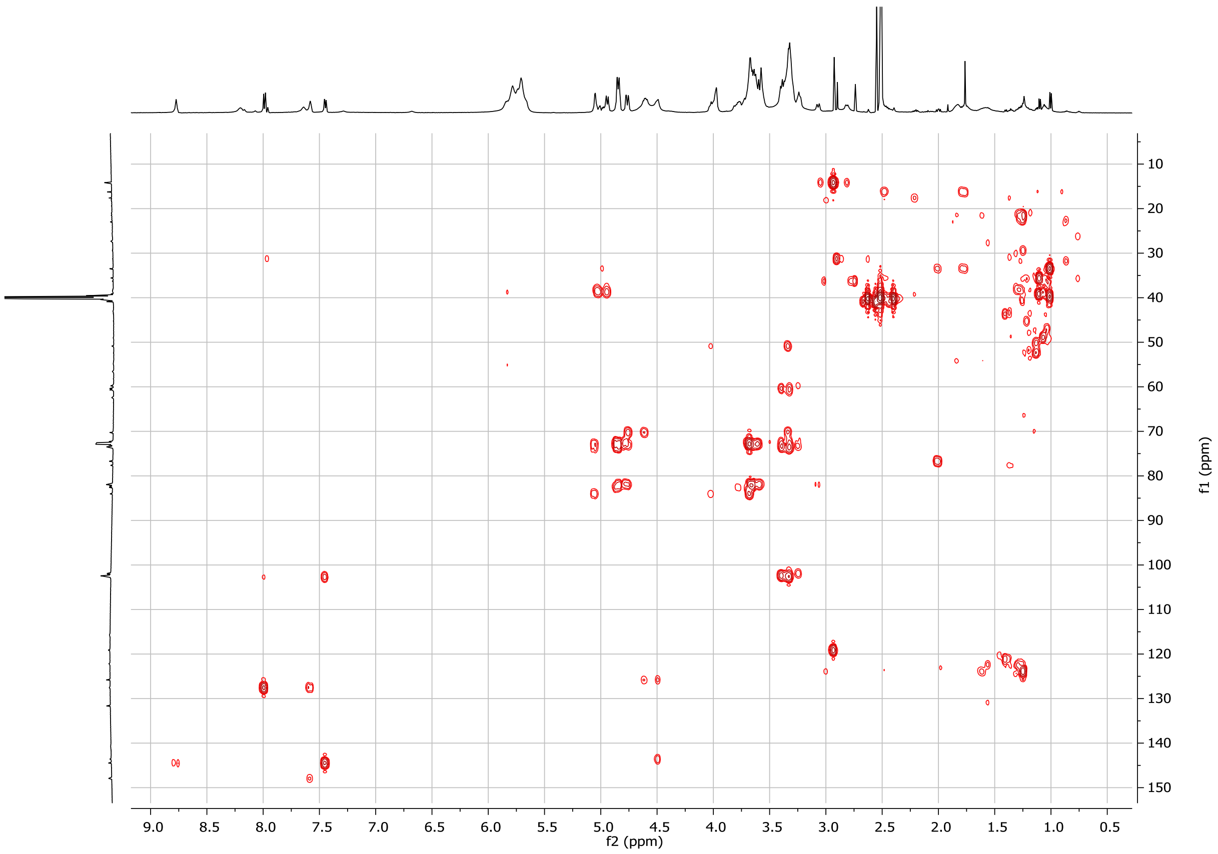

Figure S30: HMBC spectrum of compound **5c**.

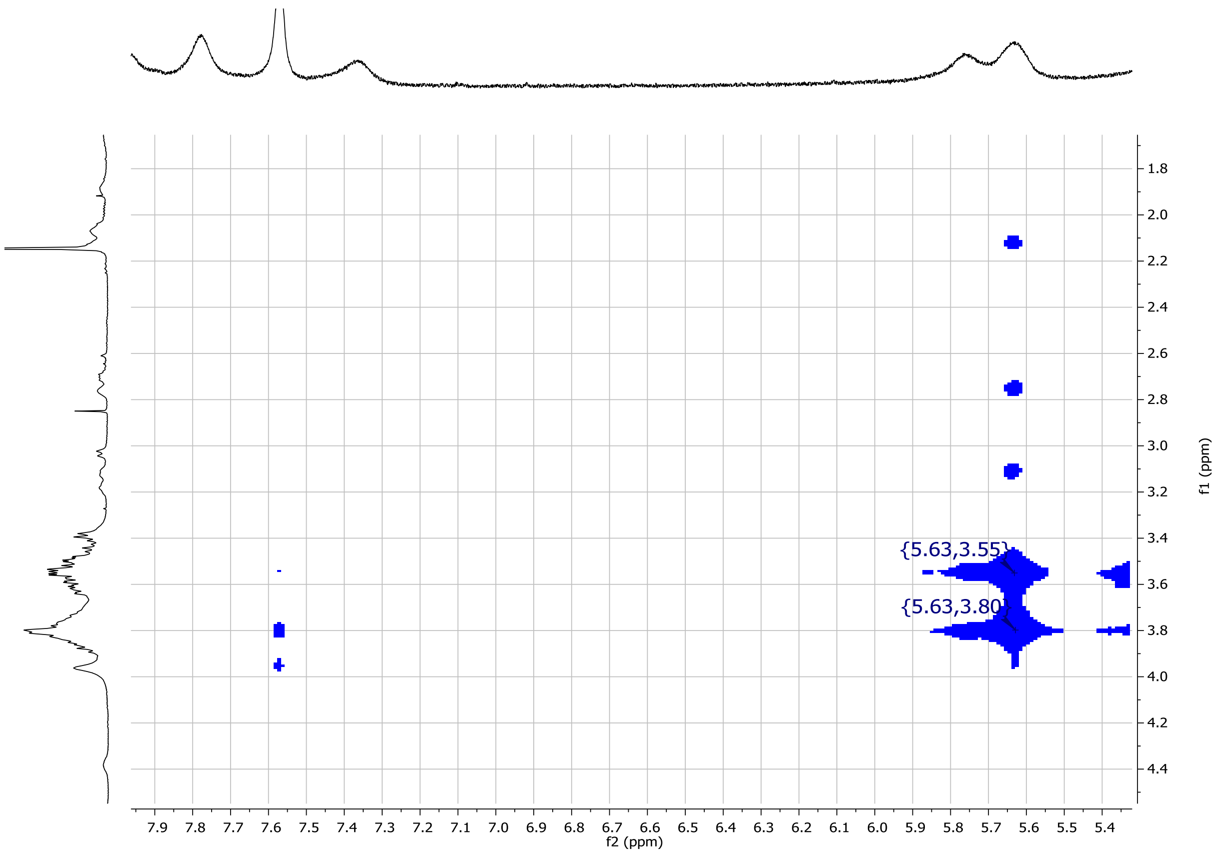

Figure S31: The part of 2D ROESY spectrum of compound **5c**.

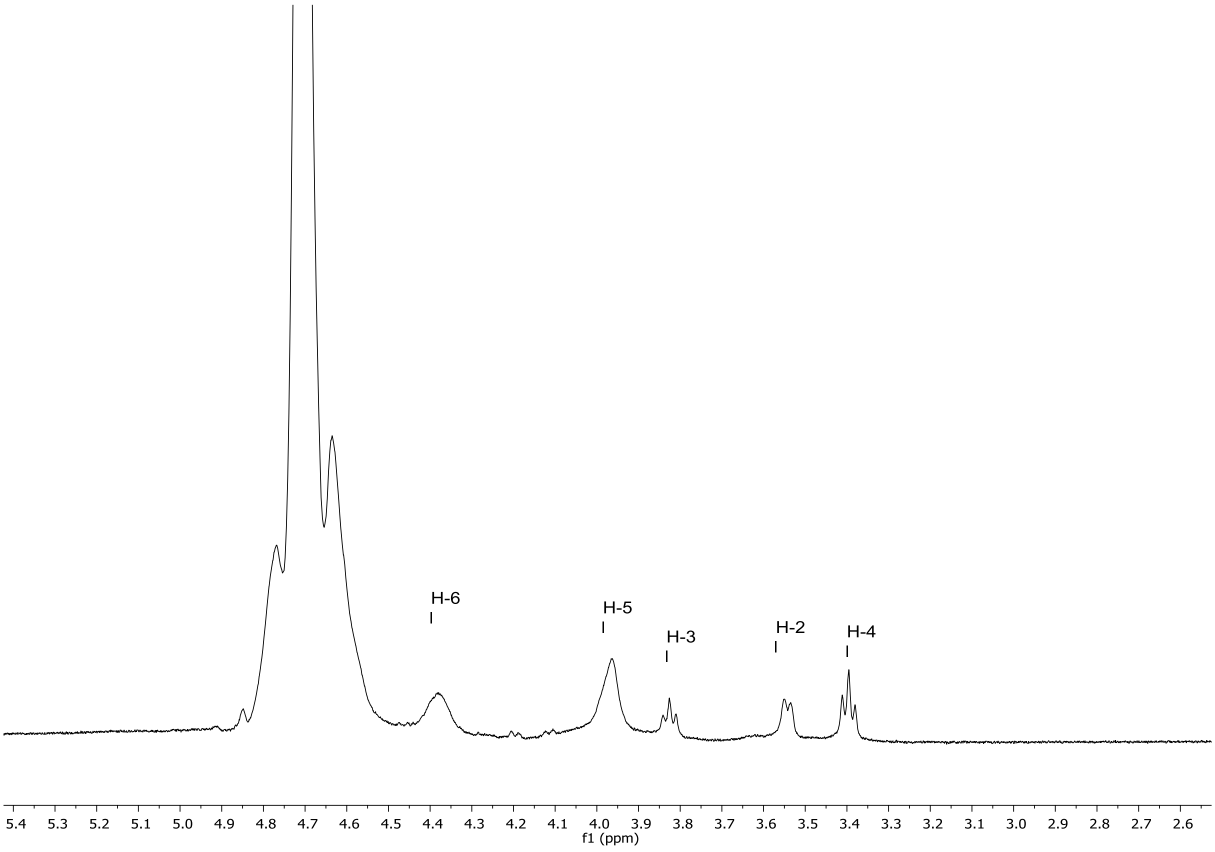

Figure S32: 1D TOCSY spectrum of compound **5c**.

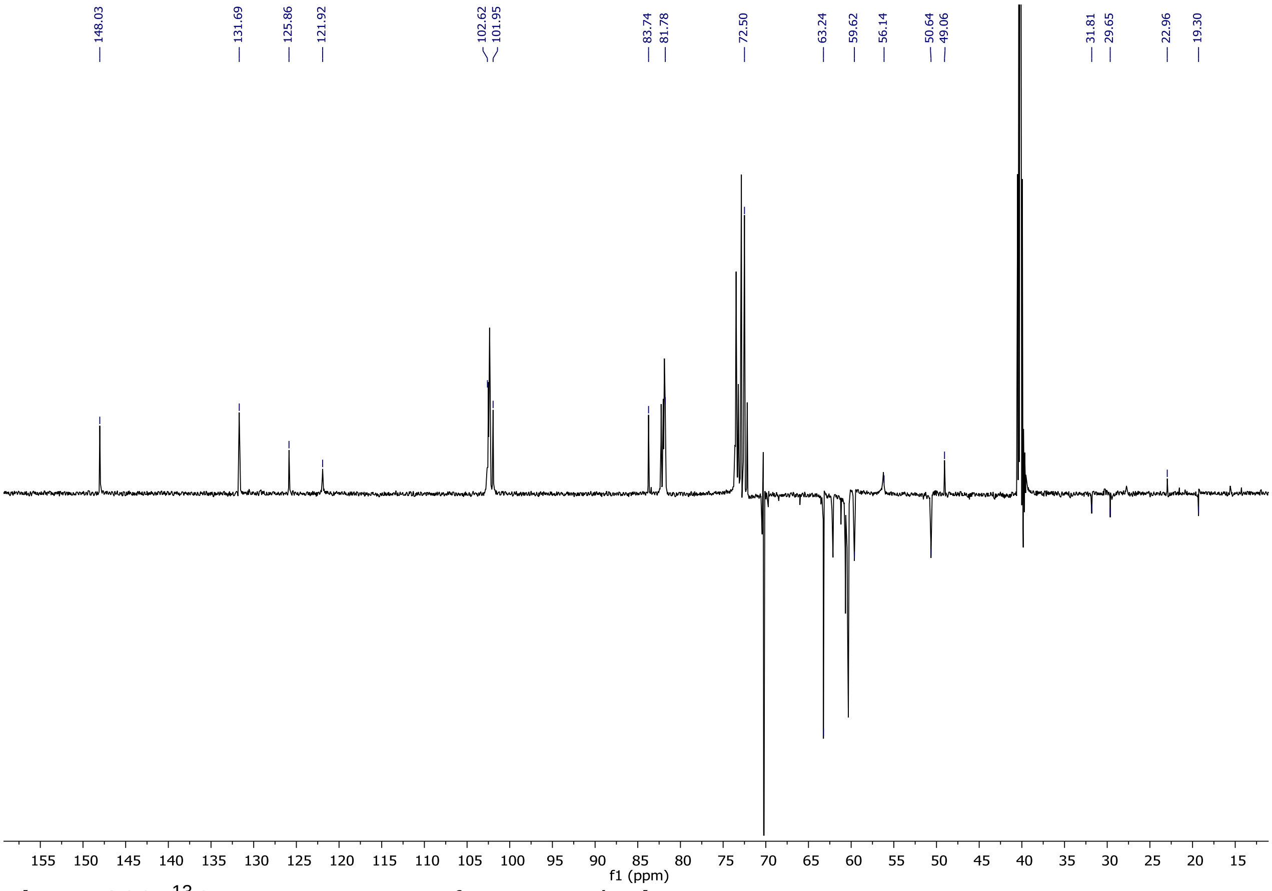

Figure S33: ^{13}C DEPT spectrum of compound **5d**.

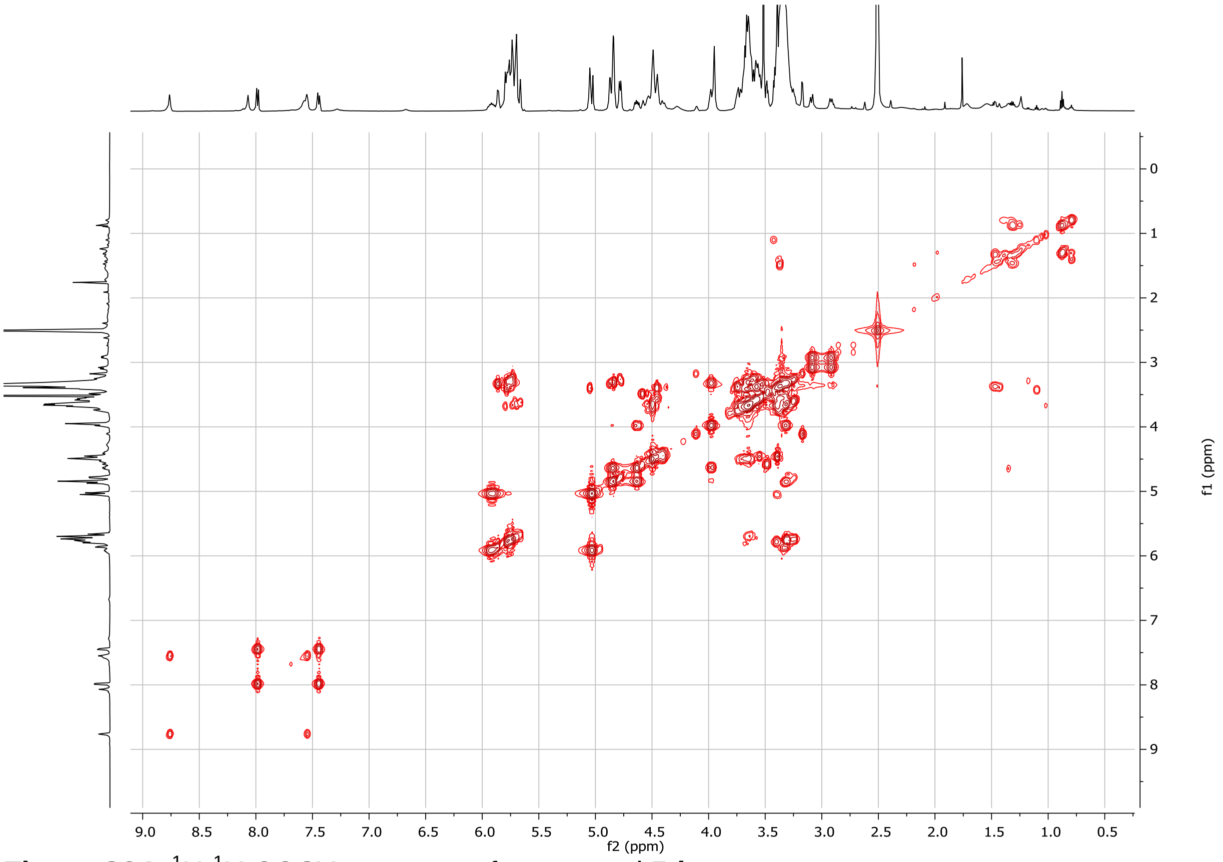

Figure S34: ^{1}H-^{1}H COSY spectrum of compound **5d**.

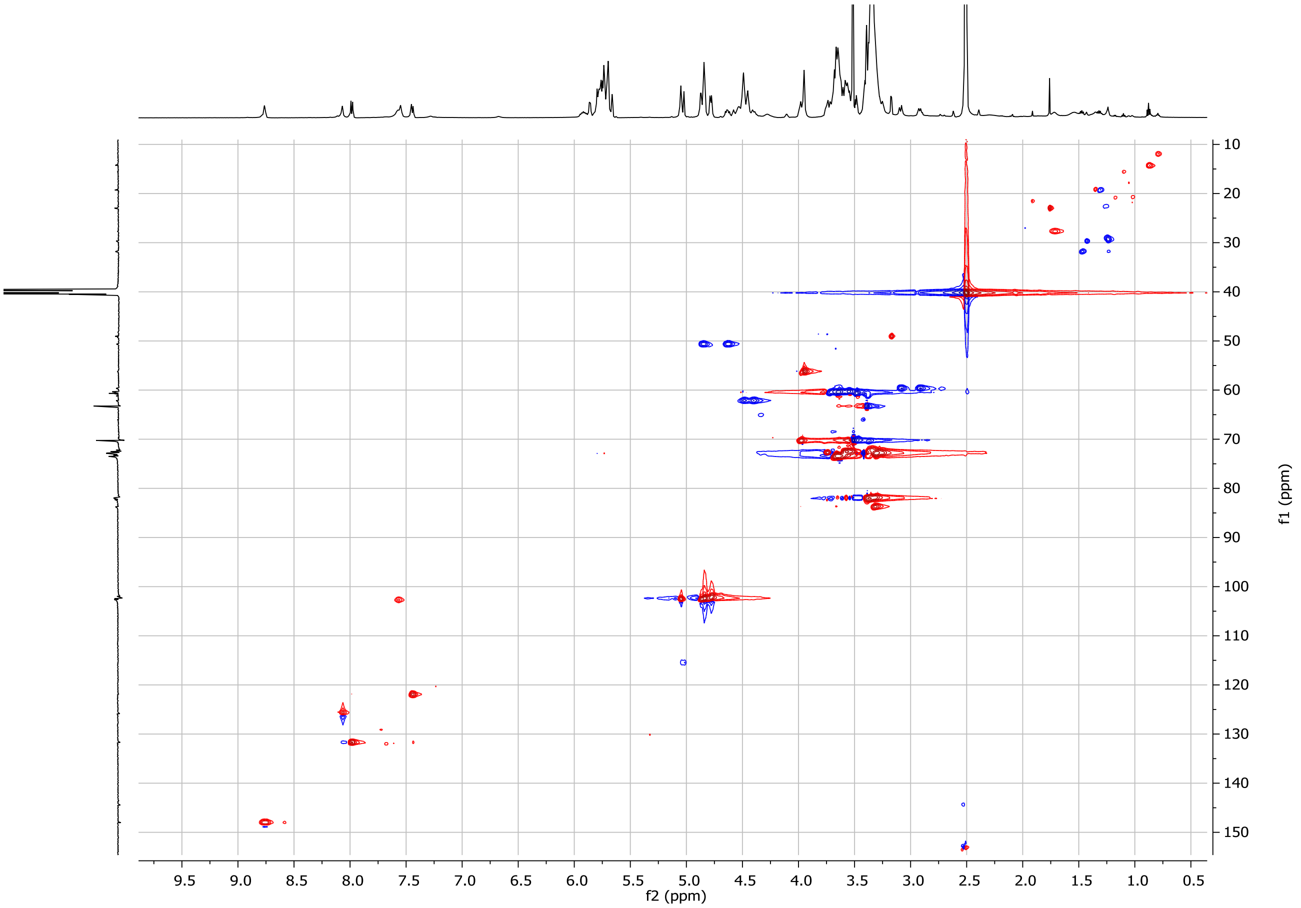

Figure S35: HSQC spectrum of compound **5d**.

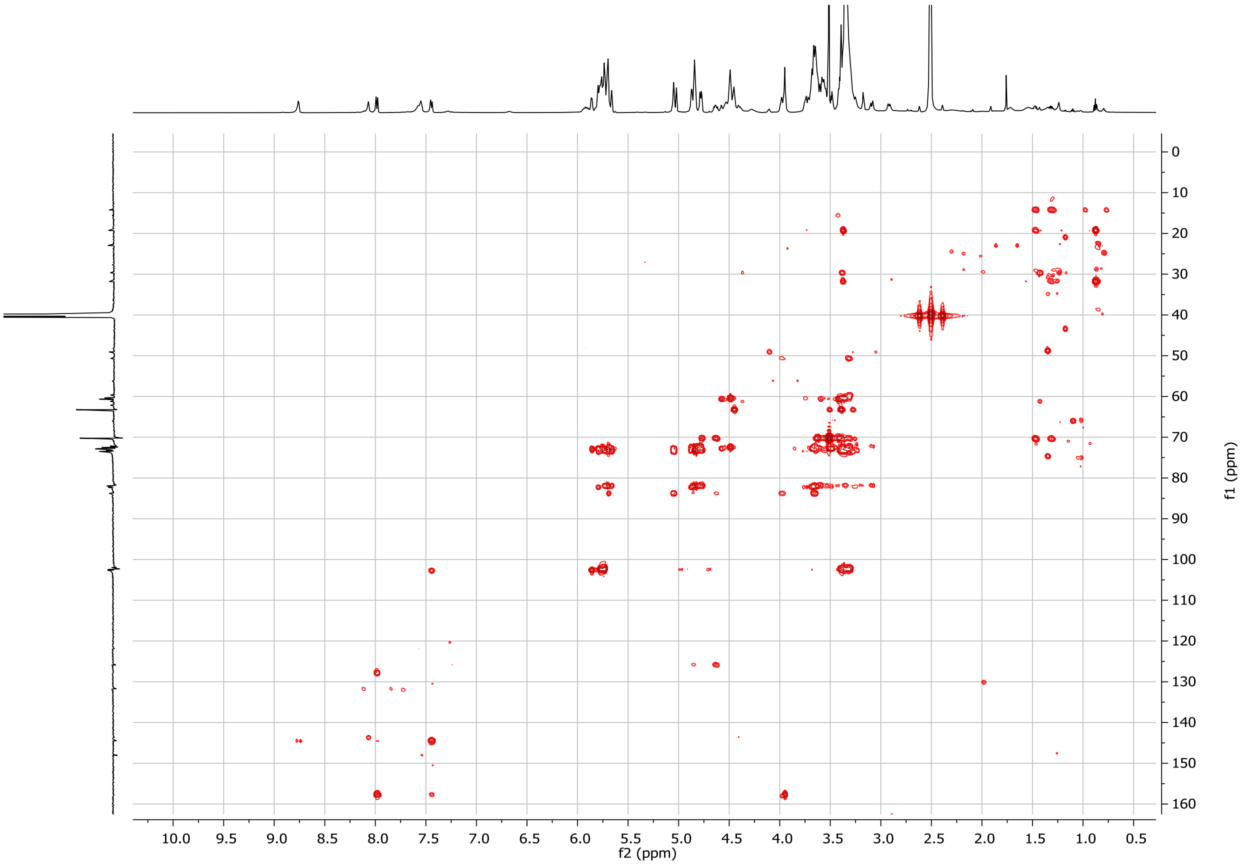

Figure S36: HMBC spectrum of compound **5d**.
