# Supplementary material for: New α- and β-cyclodextrin derivatives with cinchona alkaloids used in asymmetric organocatalytic reactions
Source: Beilstein J Org Chem. 2019 Apr 1;15:830–9. doi: 10.3762/bjoc.15.80 (PMC6466772; doi:10.3762/bjoc.15.80)
Supplement: File 3 — 2D NMR spectra of compounds 8a–d, 9a–d and 11. [file Beilstein_J_Org_Chem-15-830-s003.pdf]

BEILSTEIN JOURNAL OF ORGANIC CHEMISTRY

Supporting Information

for

New α- and β-cyclodextrin derivatives with cinchona alkaloids used in asymmetric organocatalytic reactions

Iveta Chena Tichá, Simona Hybelbauerová and Jindřich Jindřich

Beilstein J. Org. Chem. **2019,** *15,* 830–839. doi:10.3762/bjoc.15.80

2D NMR spectra of compounds 8a–d, 9a–d and 11

License and Terms: This is a supporting information file under the terms of the Creative Commons Attribution License (http://creativecommons.org/licenses/by/4.0). Please note that the reuse, redistribution and reproduction in particular requires that the authors and source are credited.
The license is subject to the *Beilstein Journal of Organic Chemistry* terms and conditions: (https://www.beilstein-journals.org/bjoc)

Table of contents

2D NMR spectra of prepared CD derivatives **8a–d**, **9a–d**, and **11** 2

2D NMR spectra of prepared CD derivatives 8a–d, 9a–d, and 11

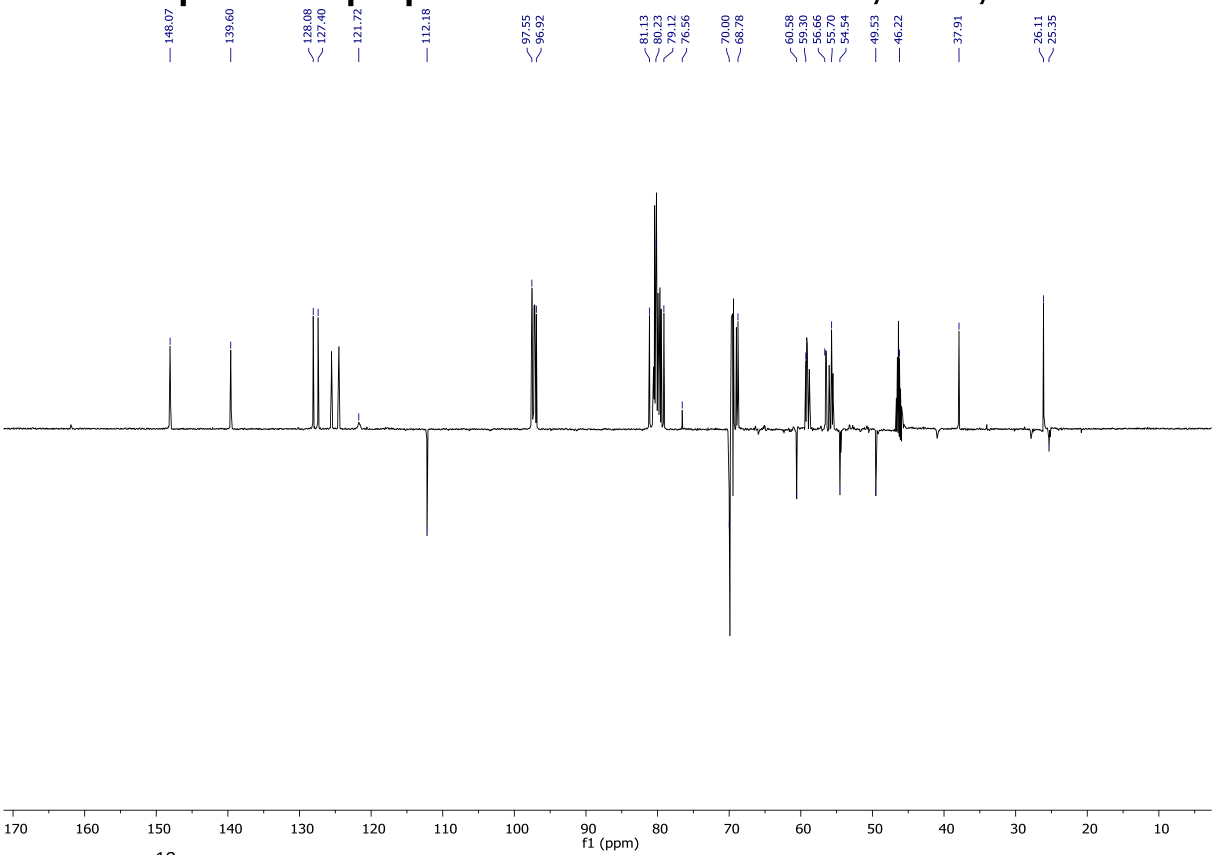

Figure S1: ^{13}C DEPT spectrum of compound **8a**.

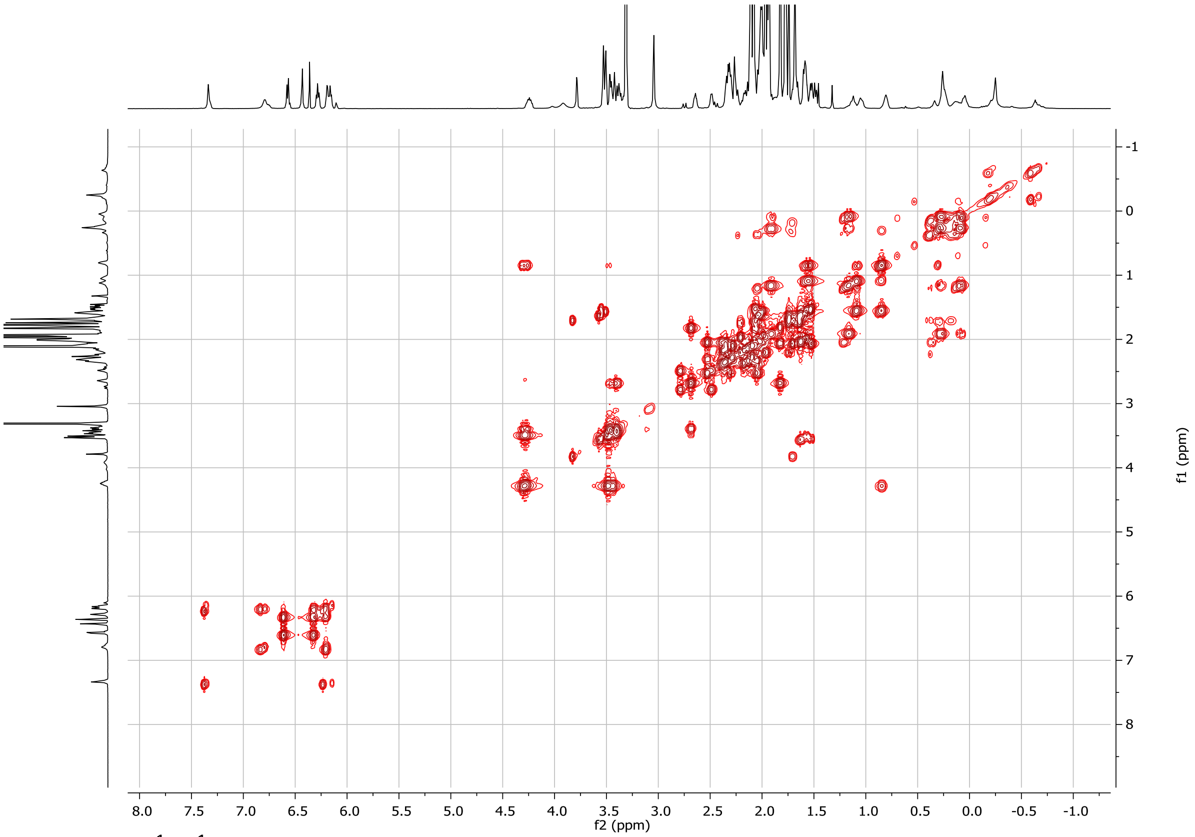

Figure S2: ^{1}H-^{1}H COSY spectrum of compound **8a**.

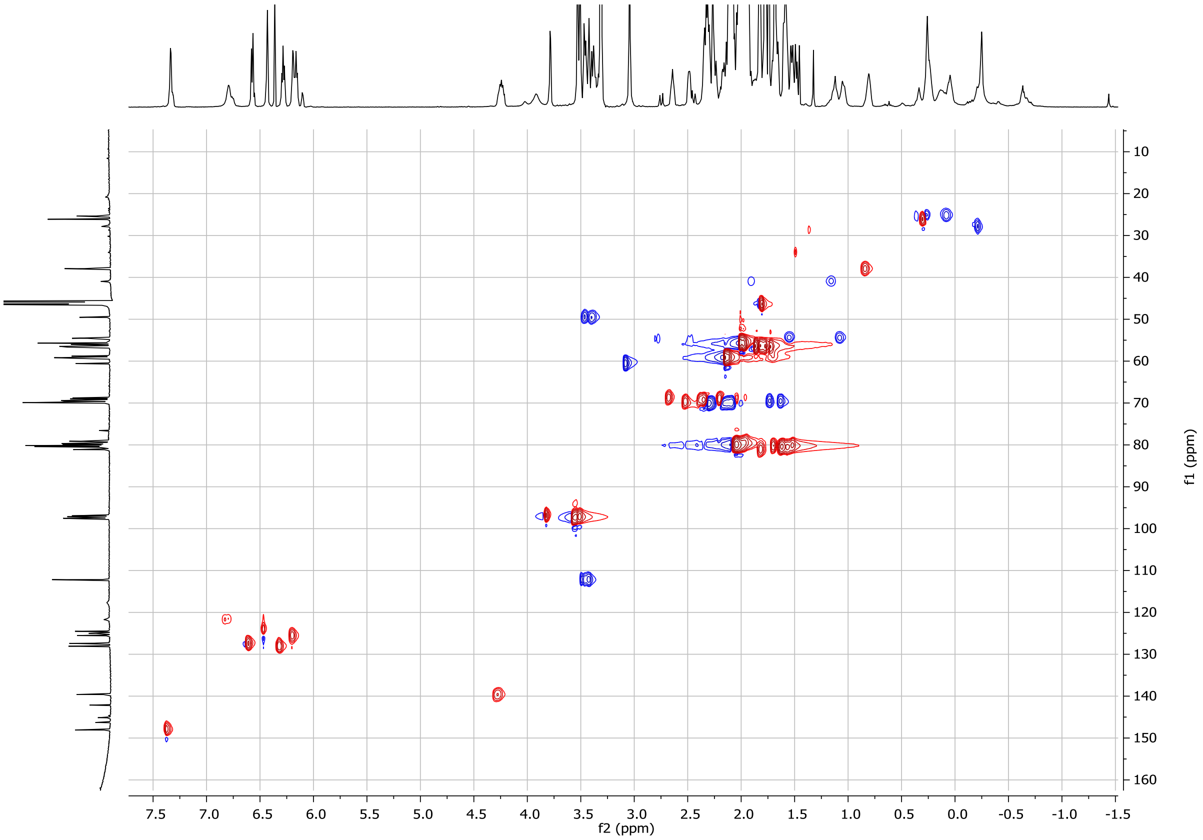

Figure S3: HSQC spectrum of compound **8a**.

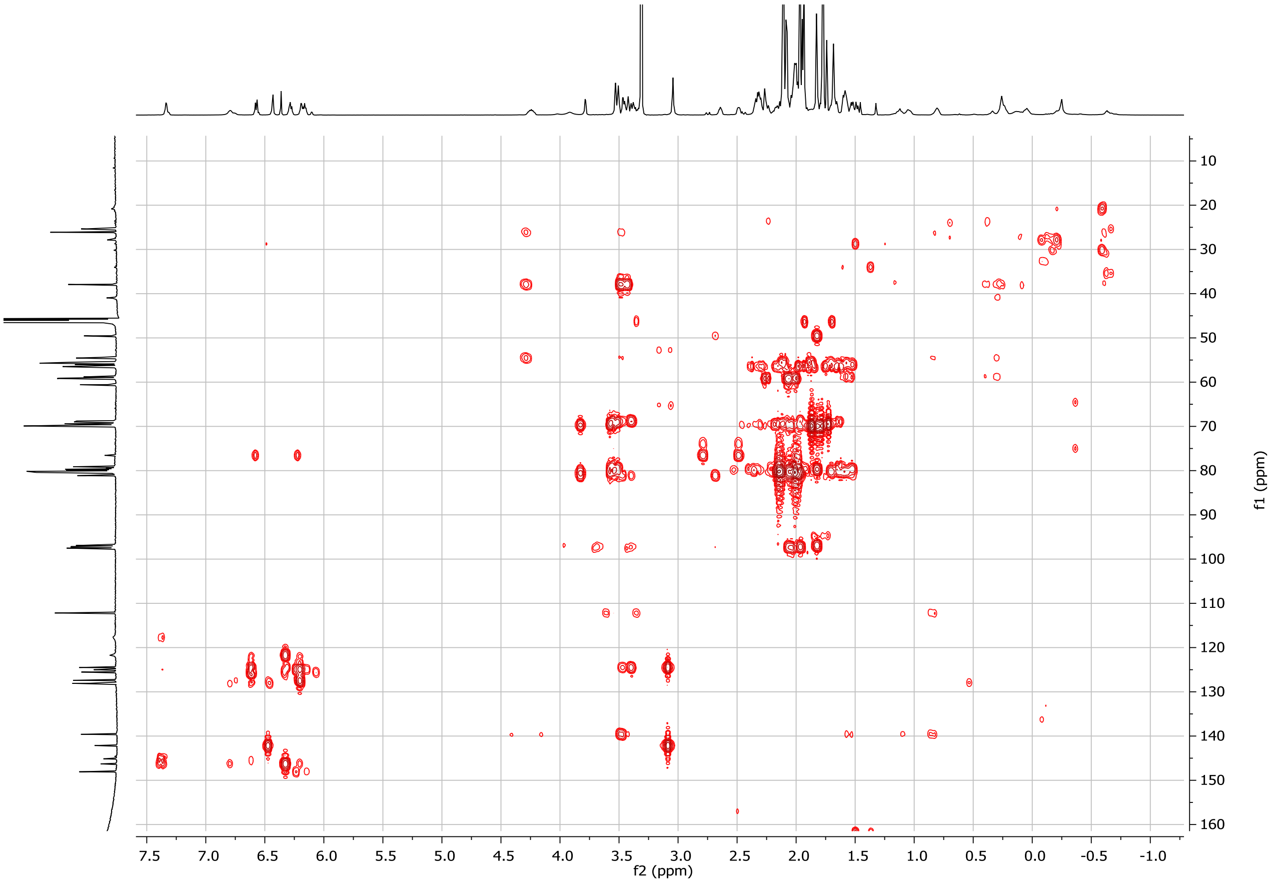

Figure S4: HMBC spectrum of compound **8a**.

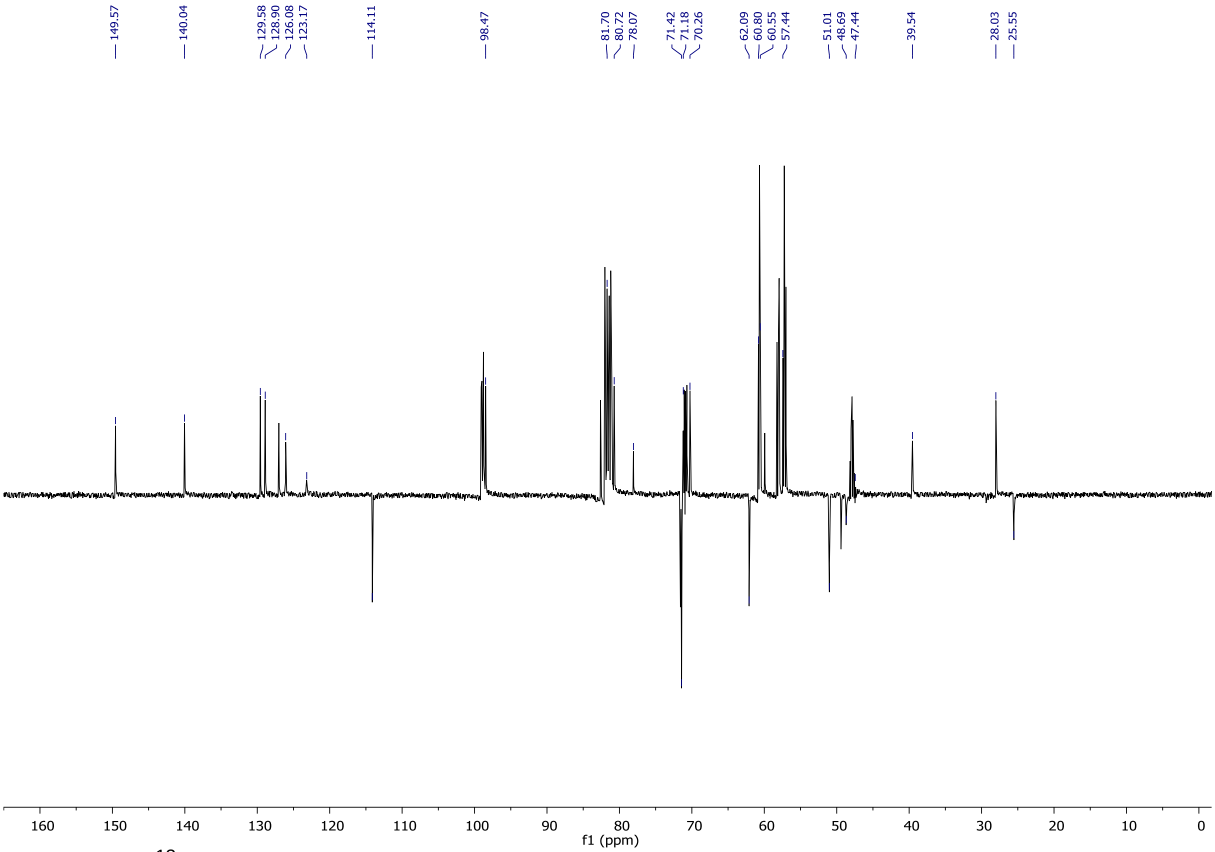

Figure S5: ^{13}C DEPT spectrum of compound **8b**.

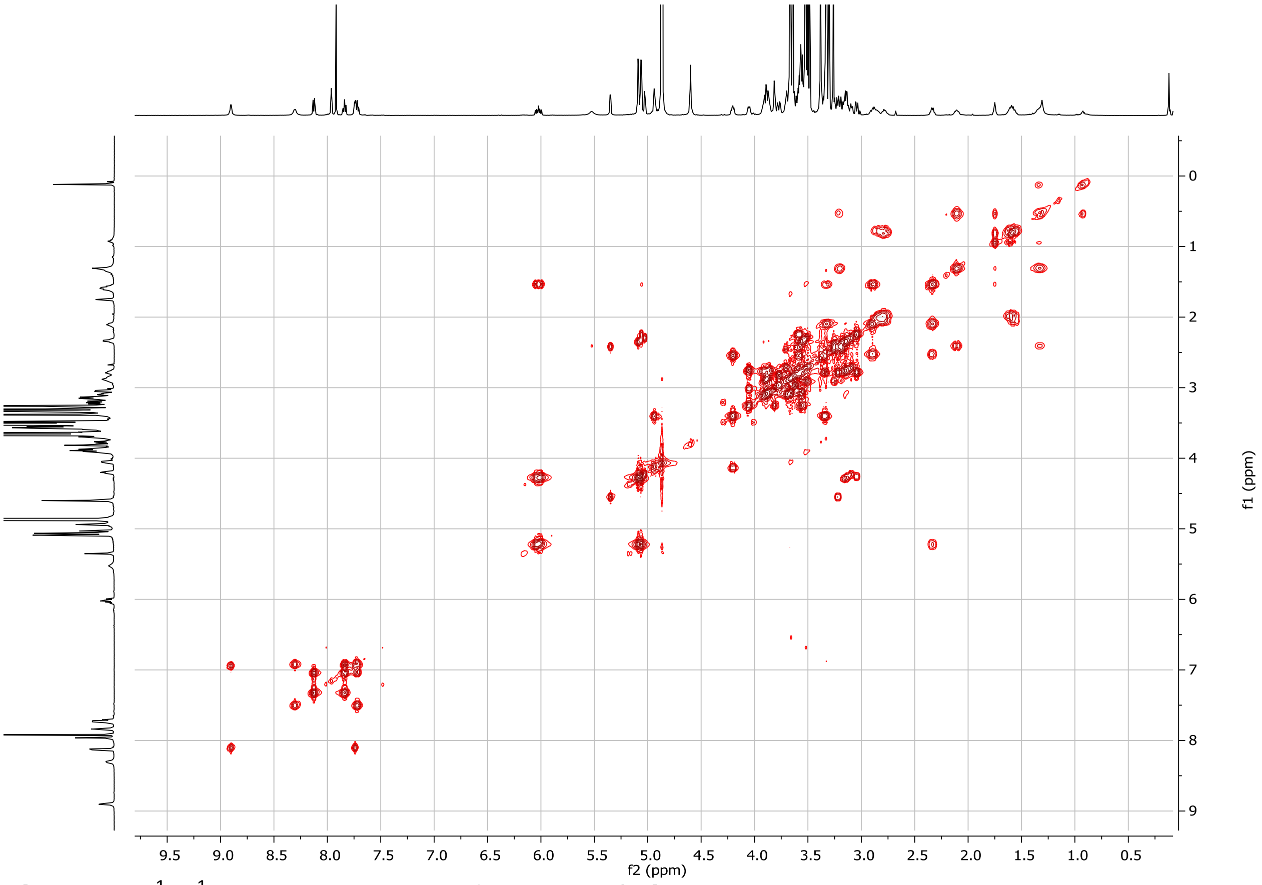

Figure S6: ^{1}H-^{1}H COSY spectrum of compound **8b**.

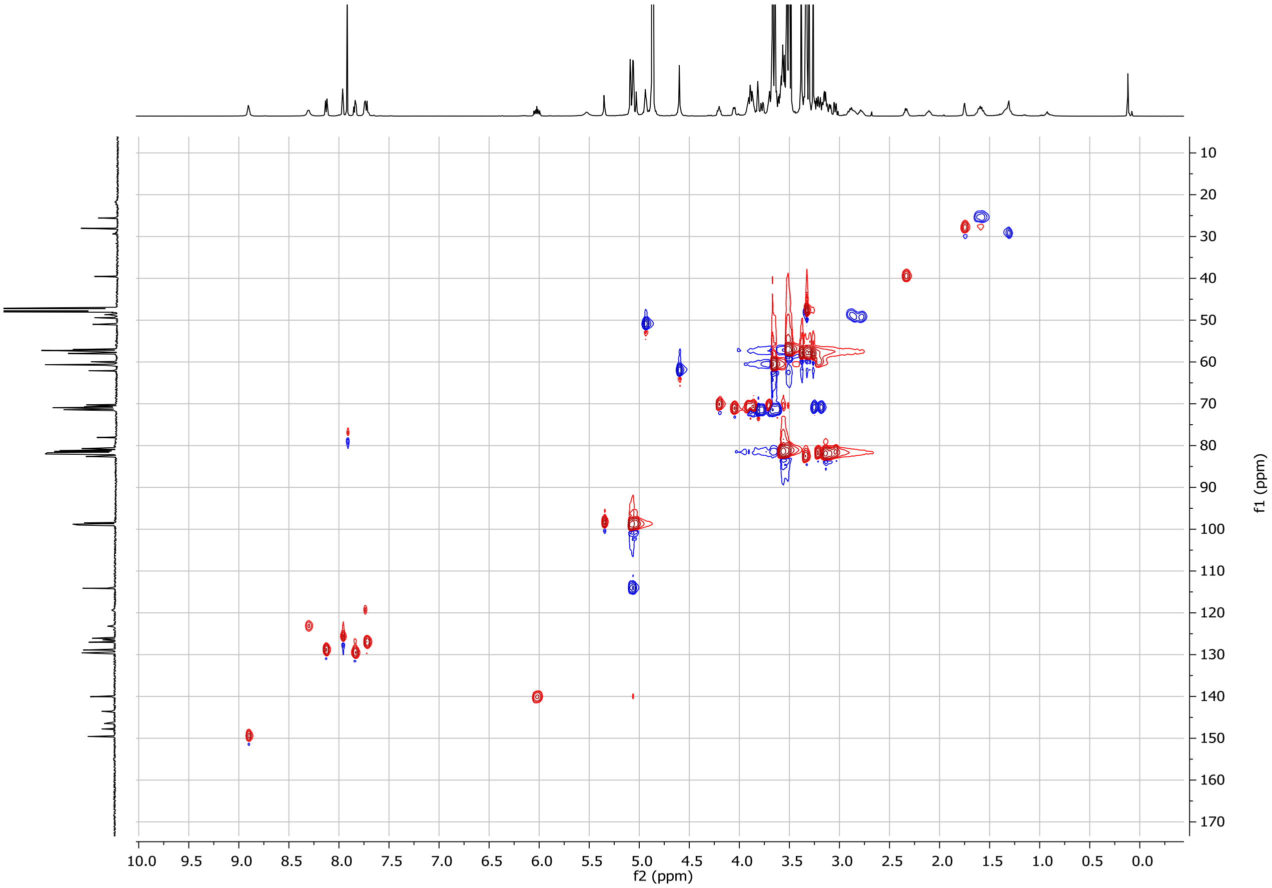

Figure S7: HSQC spectrum of compound **8b**.

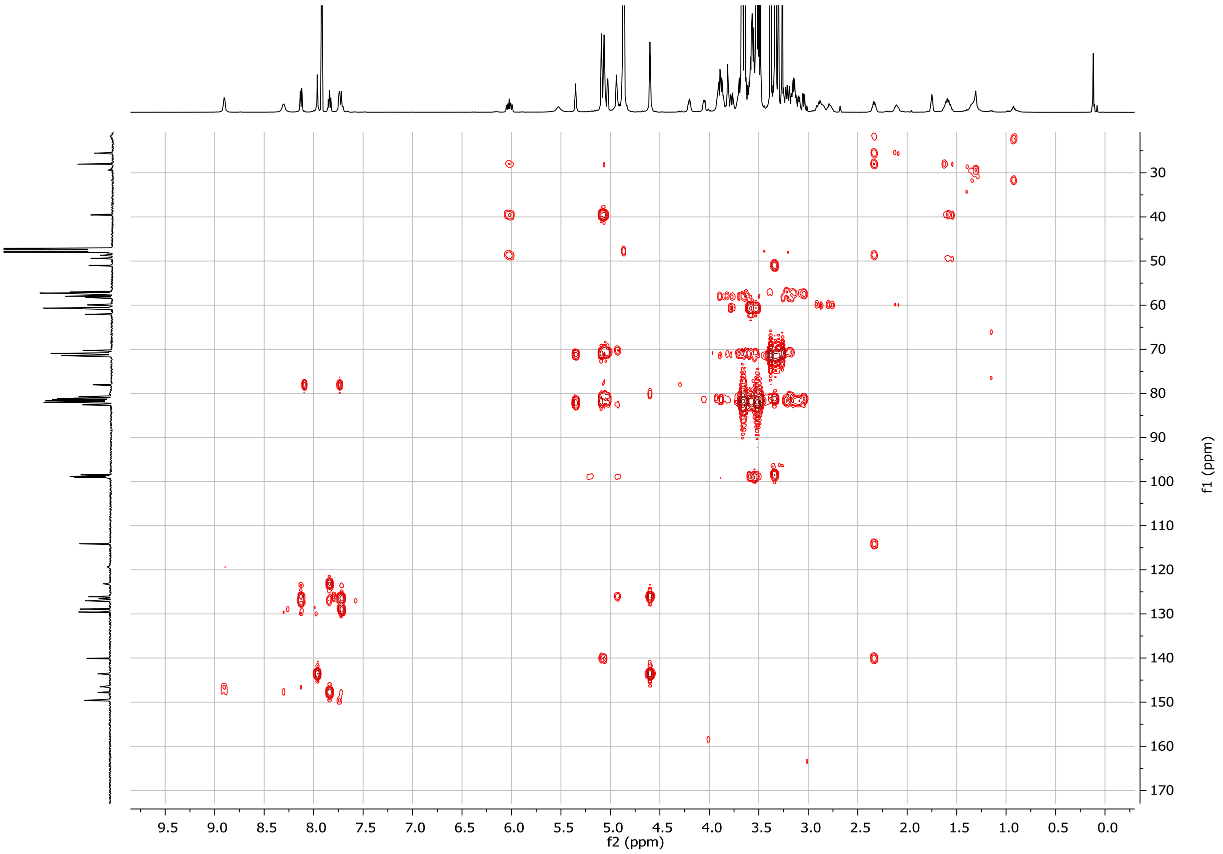

Figure S8: HMBC spectrum of compound **8b**.

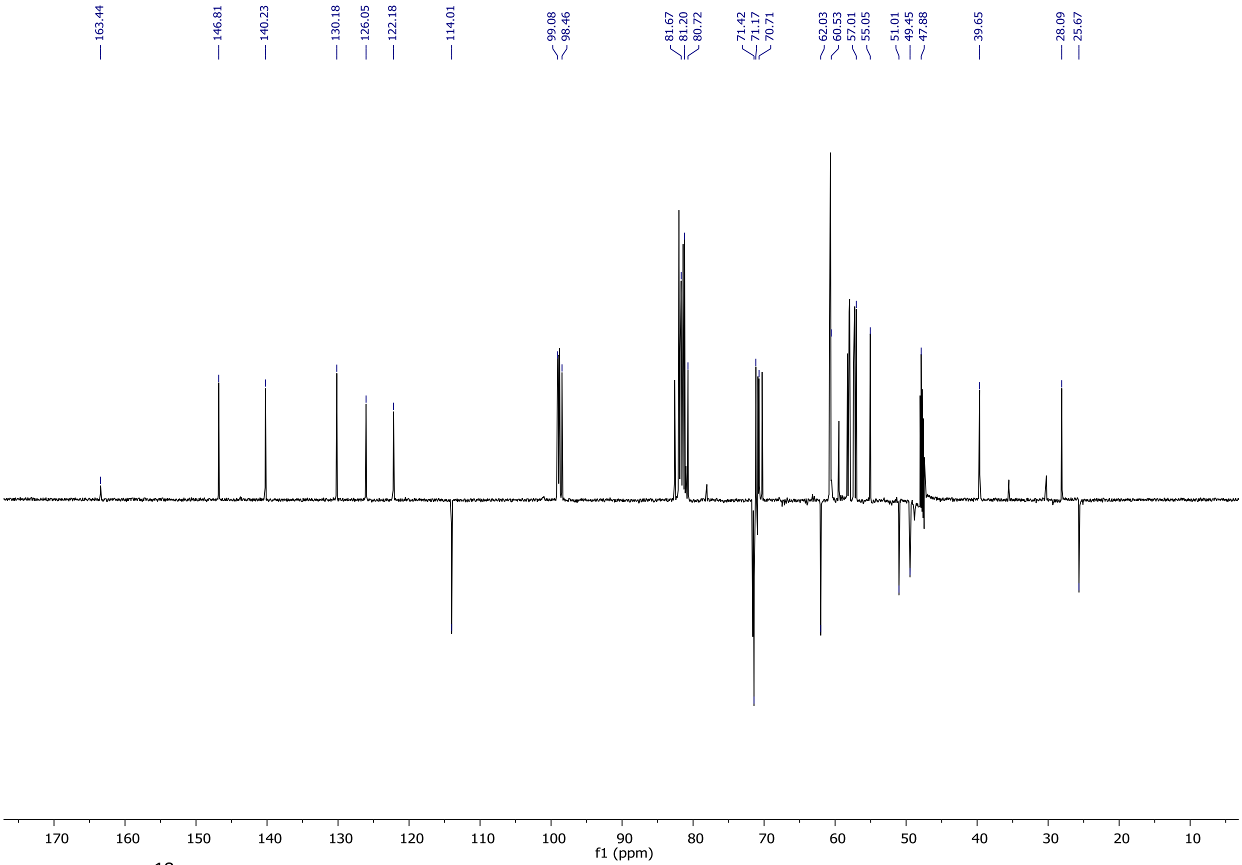

Figure S9: ^{13}C DEPT spectrum of compound **8c**.

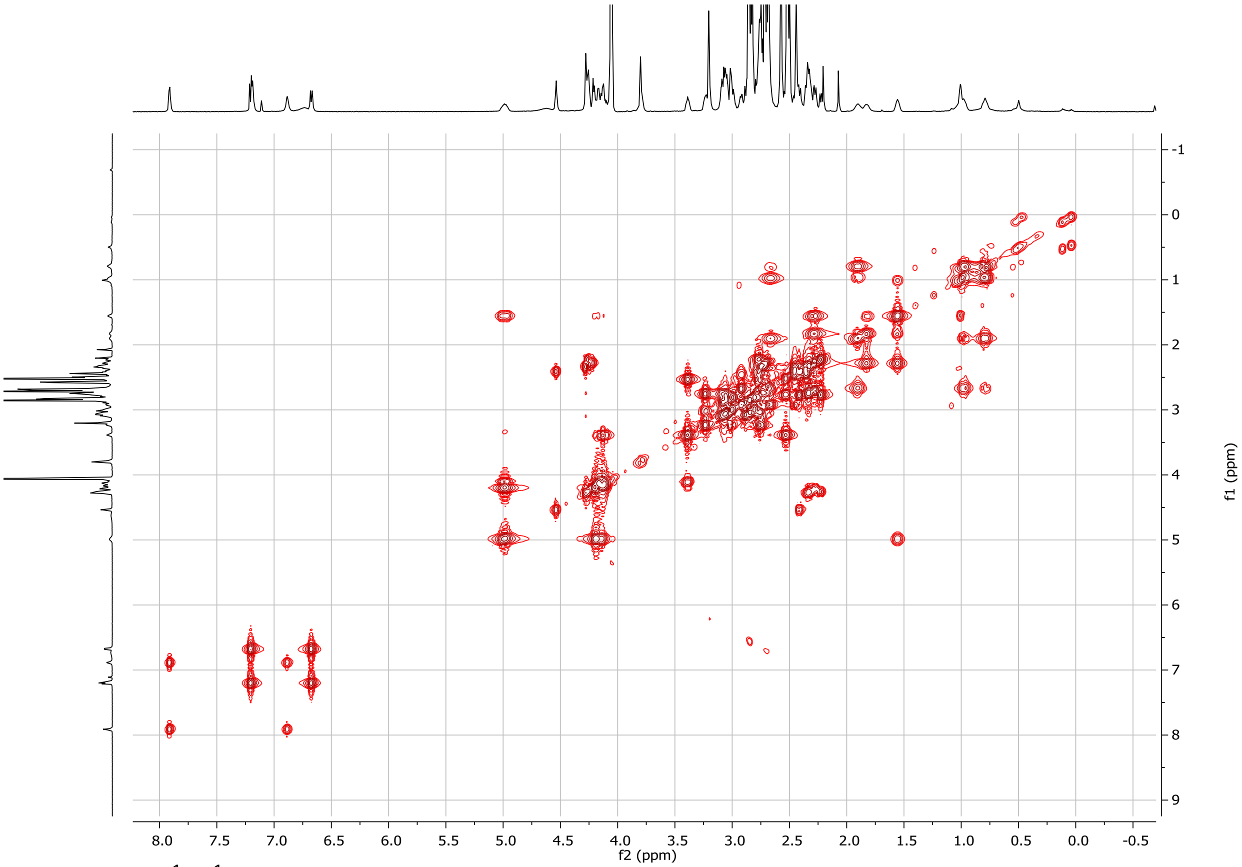

Figure S10: ^{1}H-^{1}H COSY spectrum of compound **8c**.

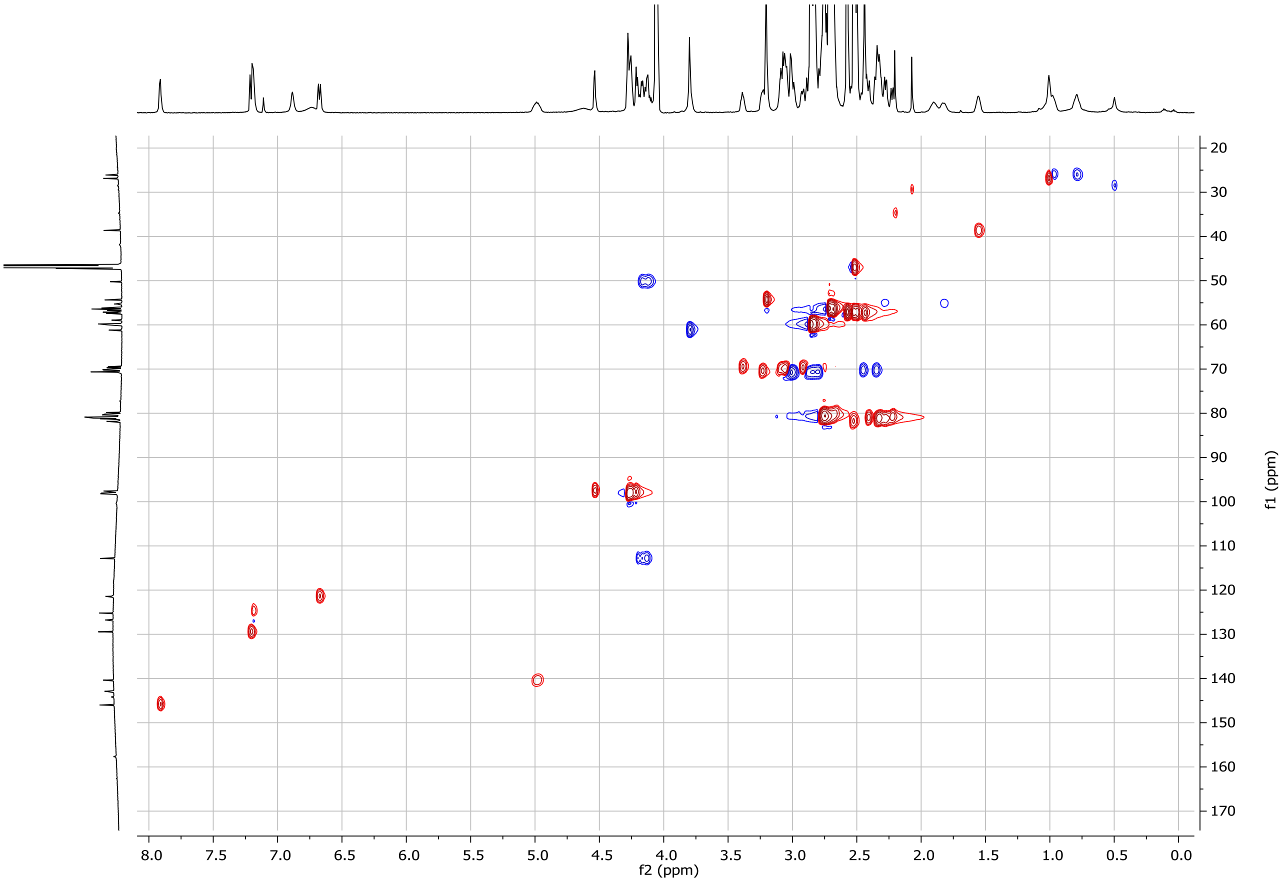

Figure S11: HSQC spectrum of compound **8c**.

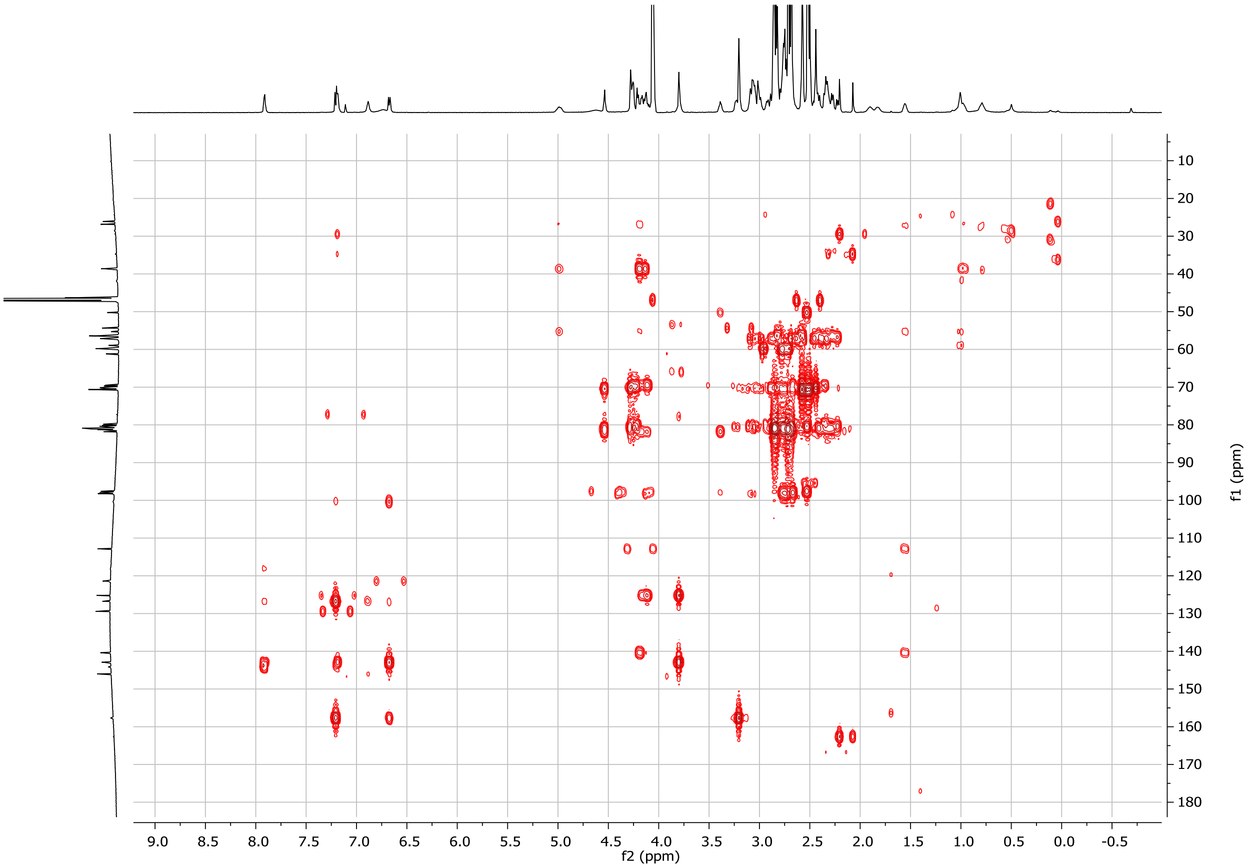

Figure S12: HMBC spectrum of compound **8c**.

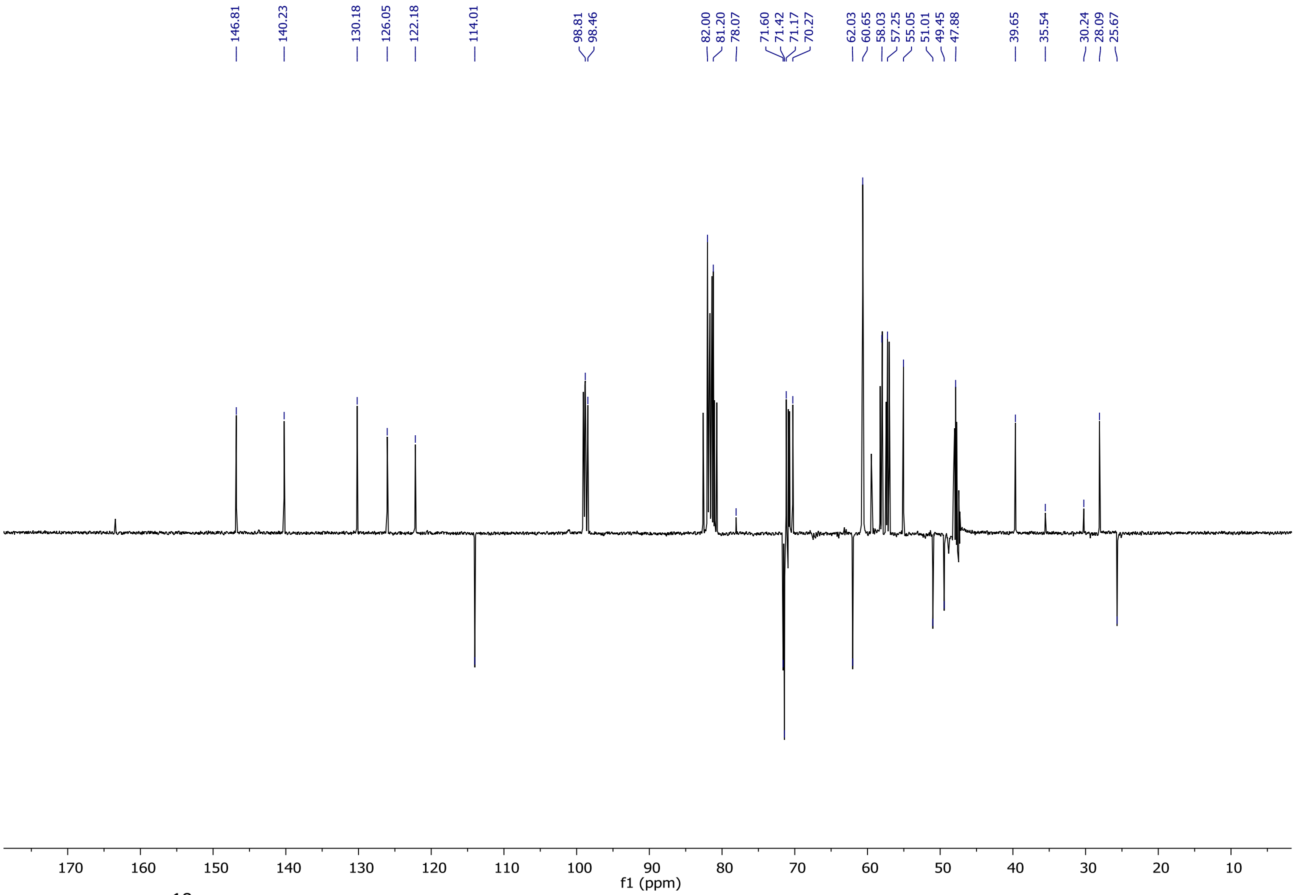

Figure S13: ^{13}C DEPT spectrum of compound **8d**.

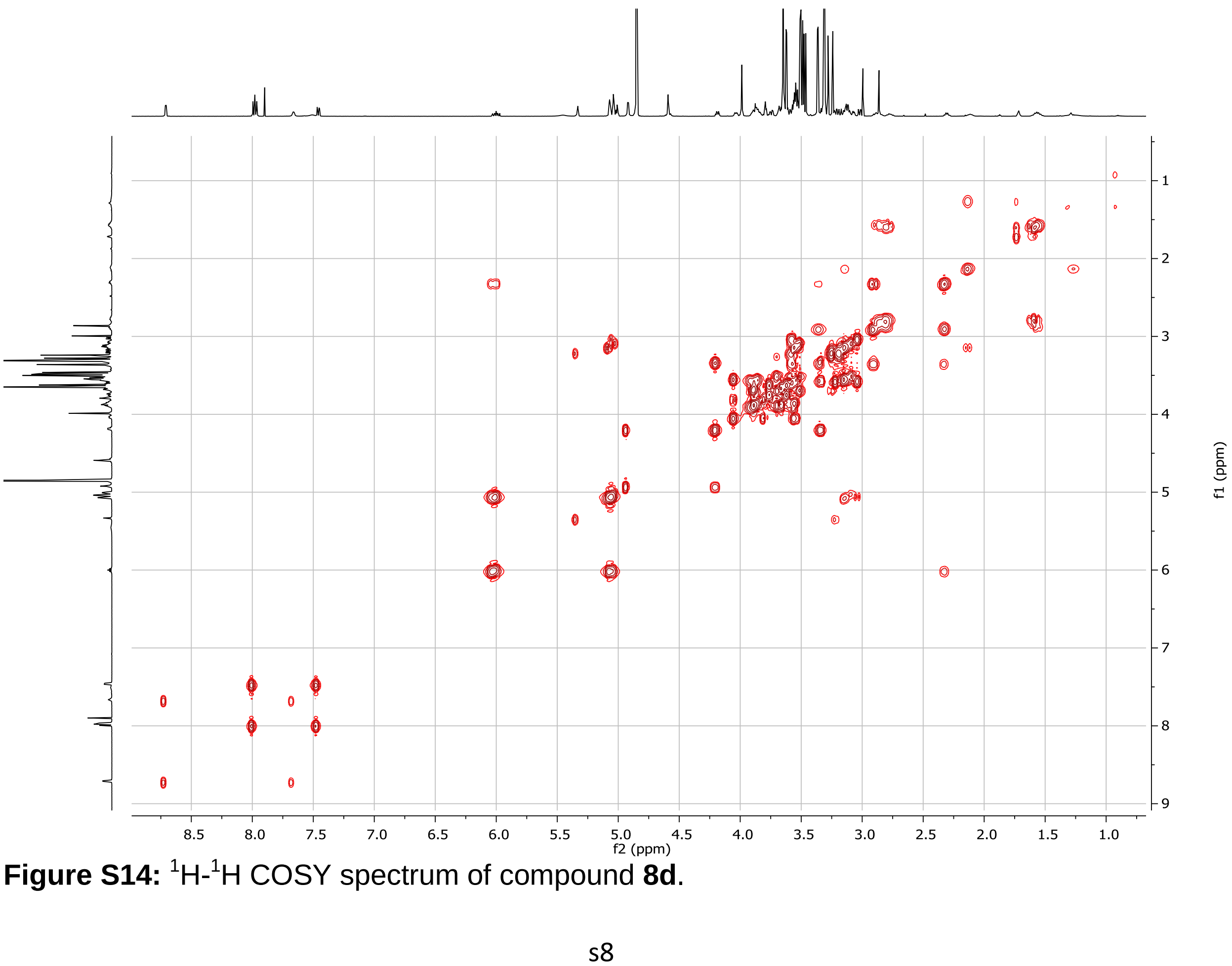

Figure S14: ^{1}H-^{1}H COSY spectrum of compound **8d**.

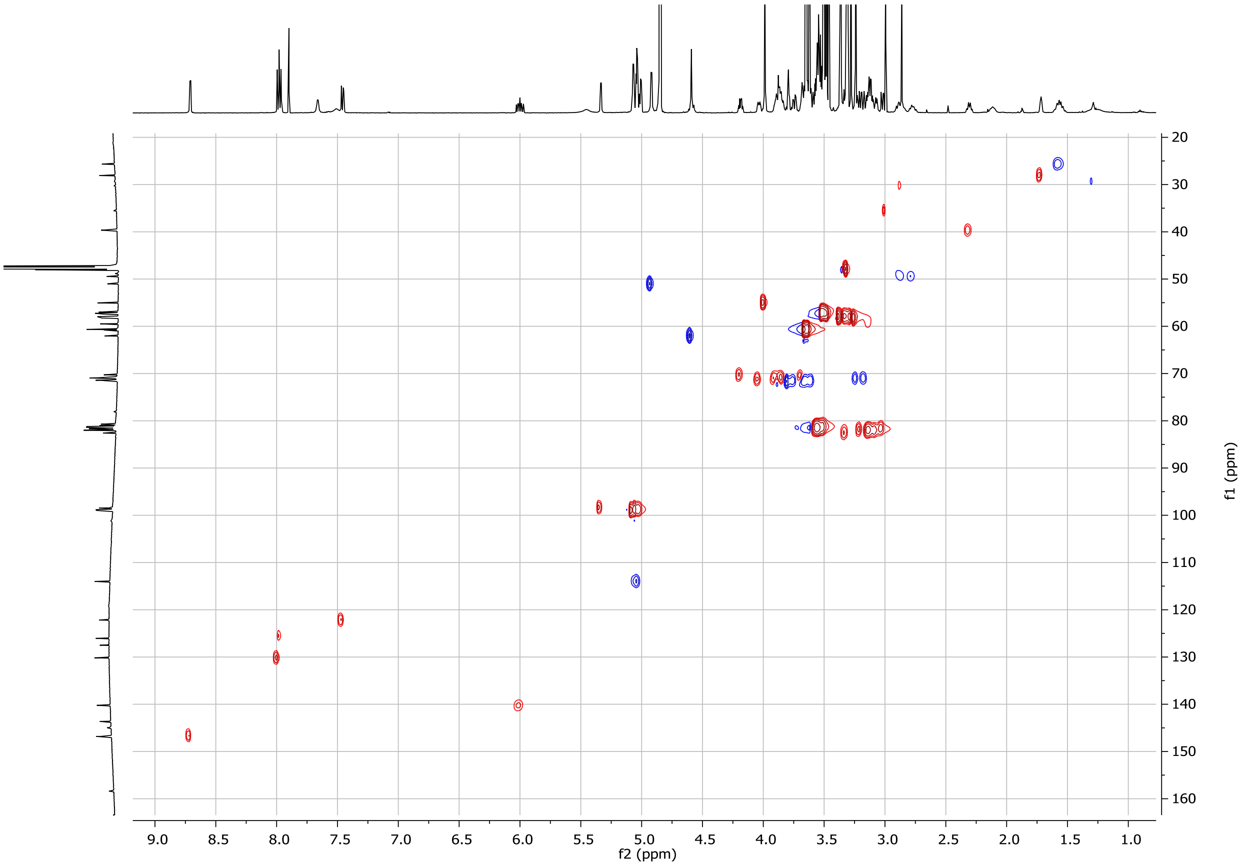

Figure S15: HSQC spectrum of spectrum of compound **8d**.

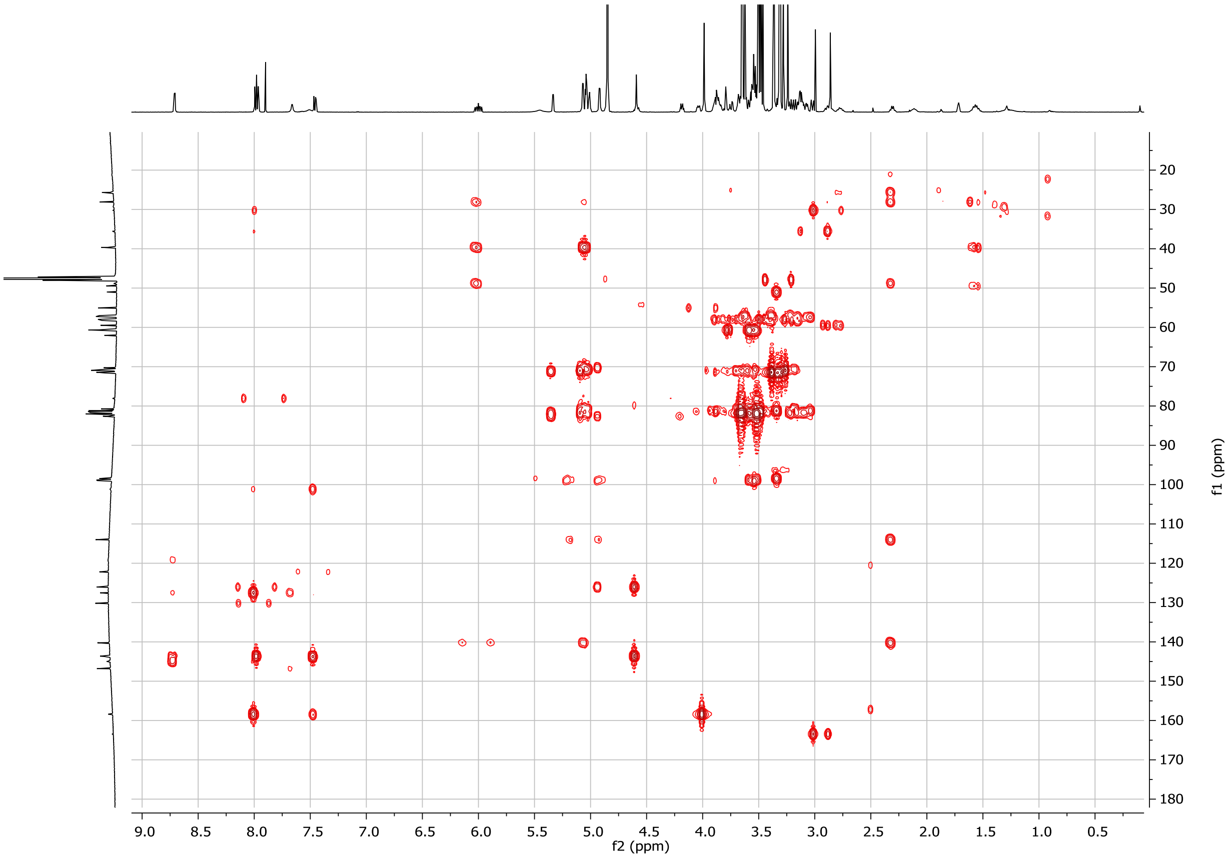

Figure S16: HMBC spectrum of spectrum of compound **8d**.

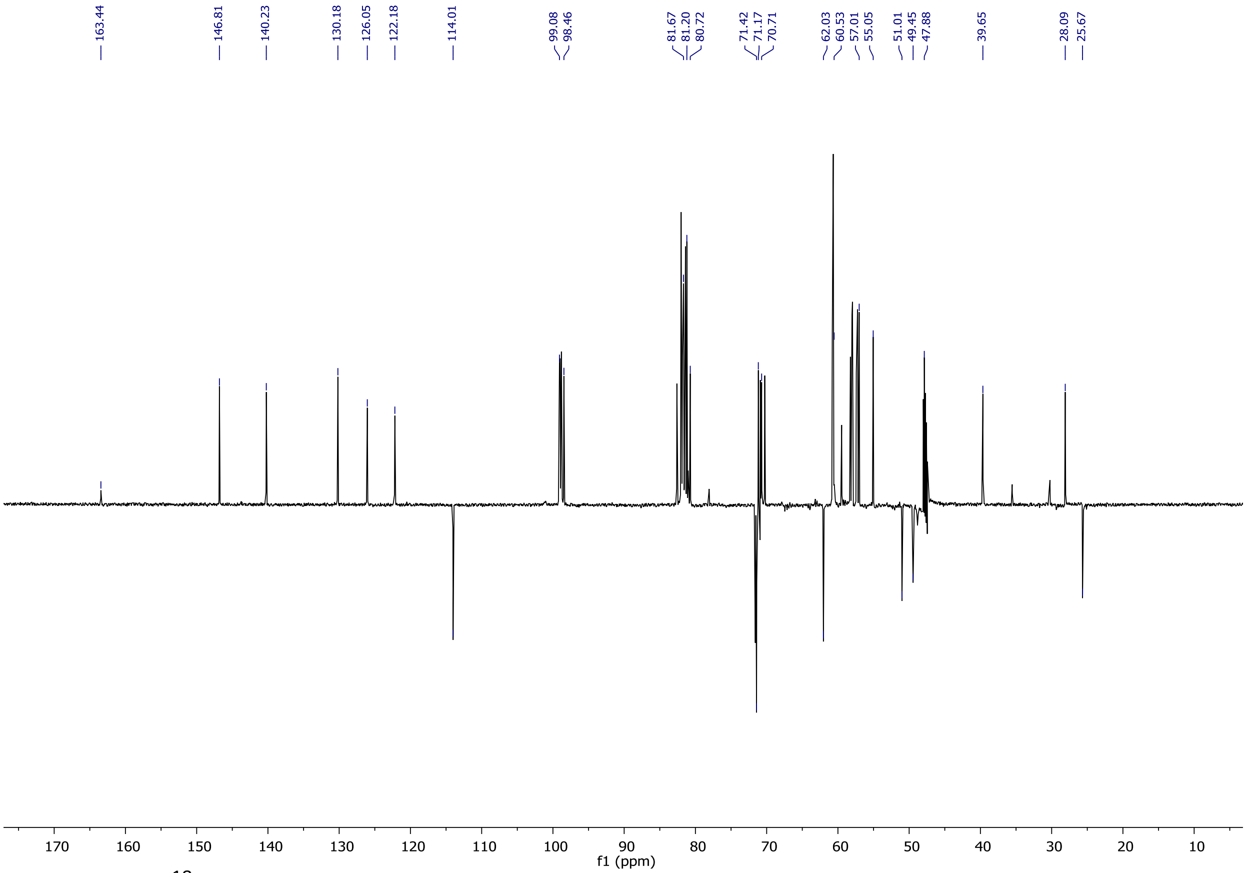

Figure S17: ^{13}C DEPT spectrum of compound **9a**.

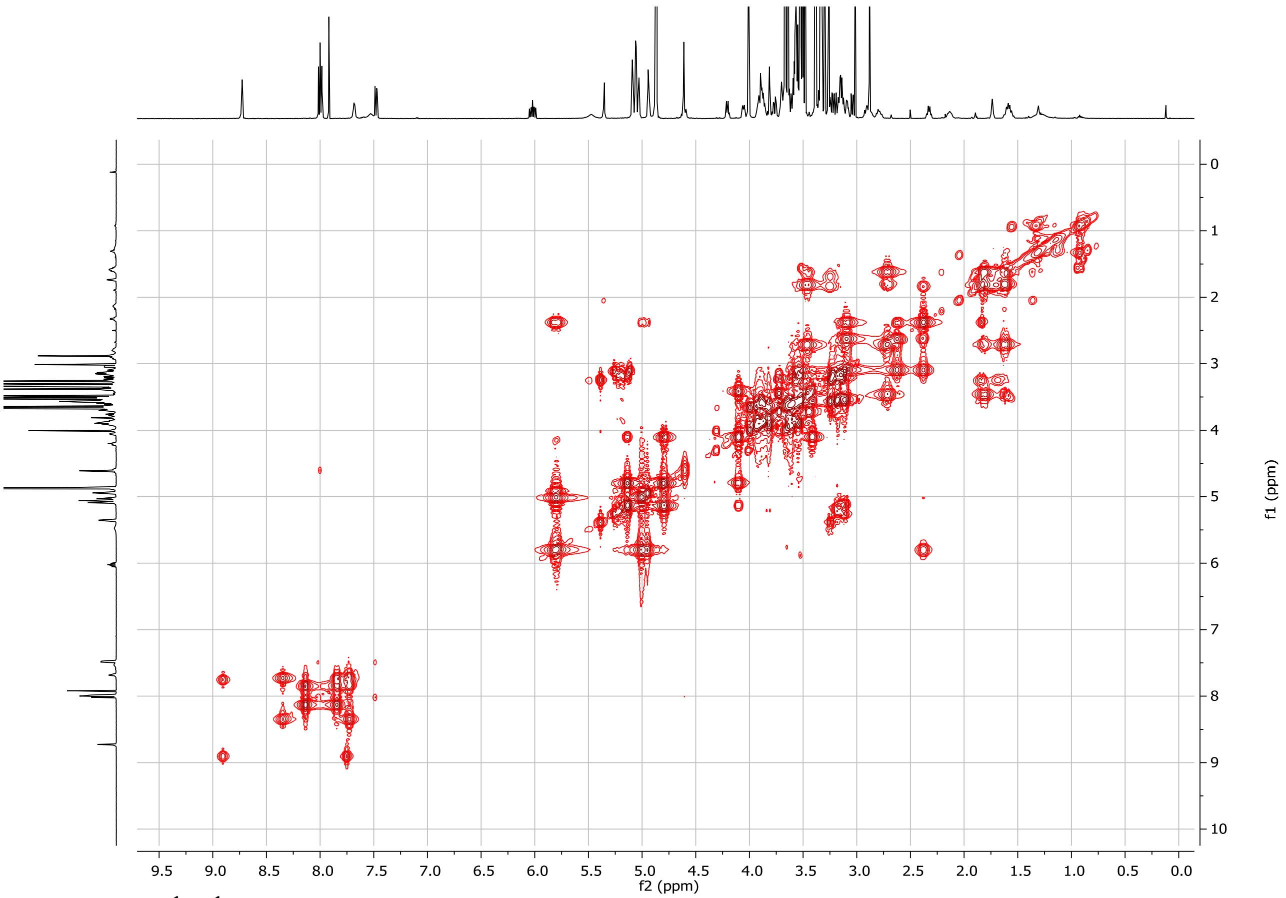

Figure S18: ^{1}H-^{1}H COSY spectrum of compound **9a**.

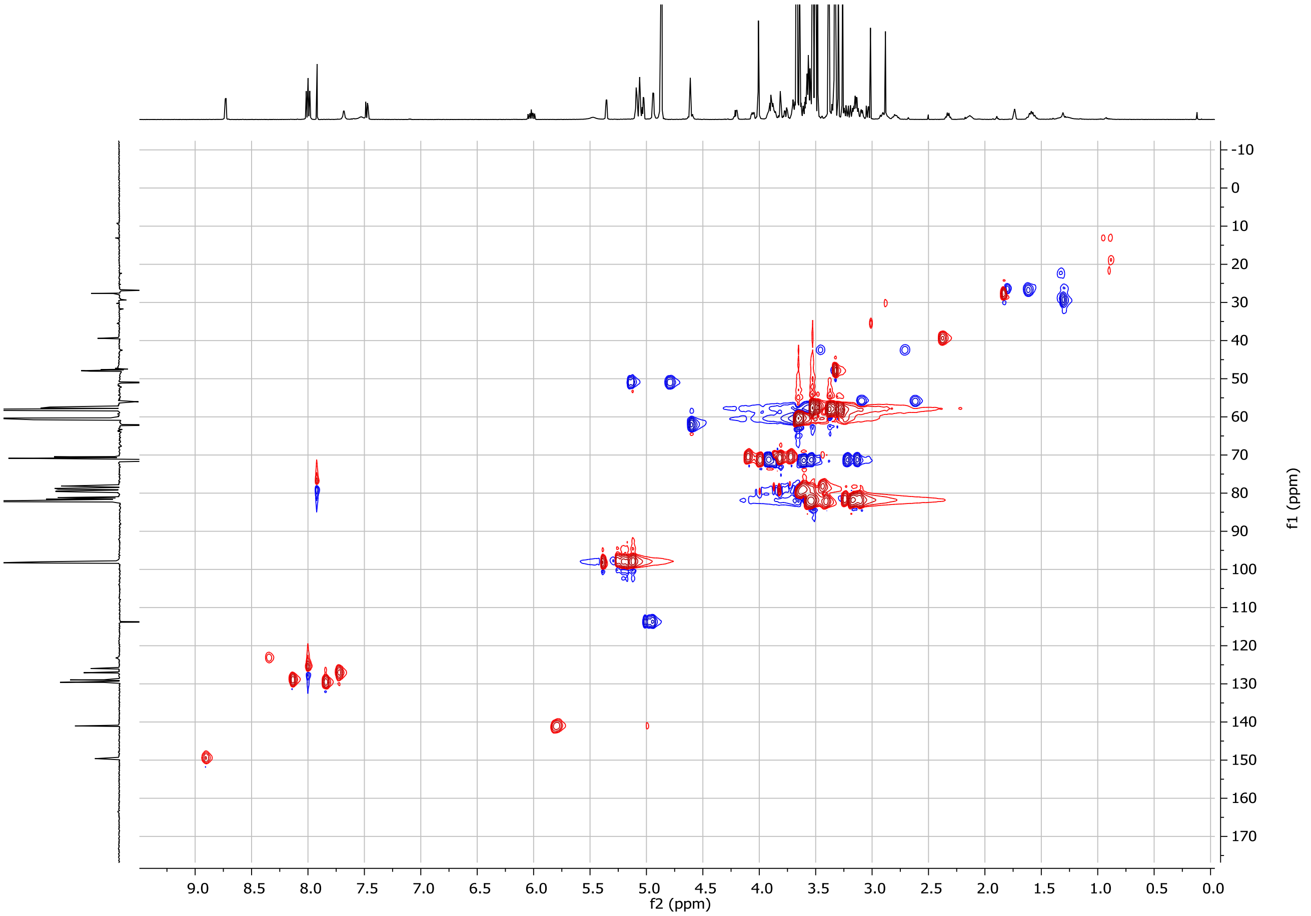

Figure S19: HSQC spectrum of compound **9a**.

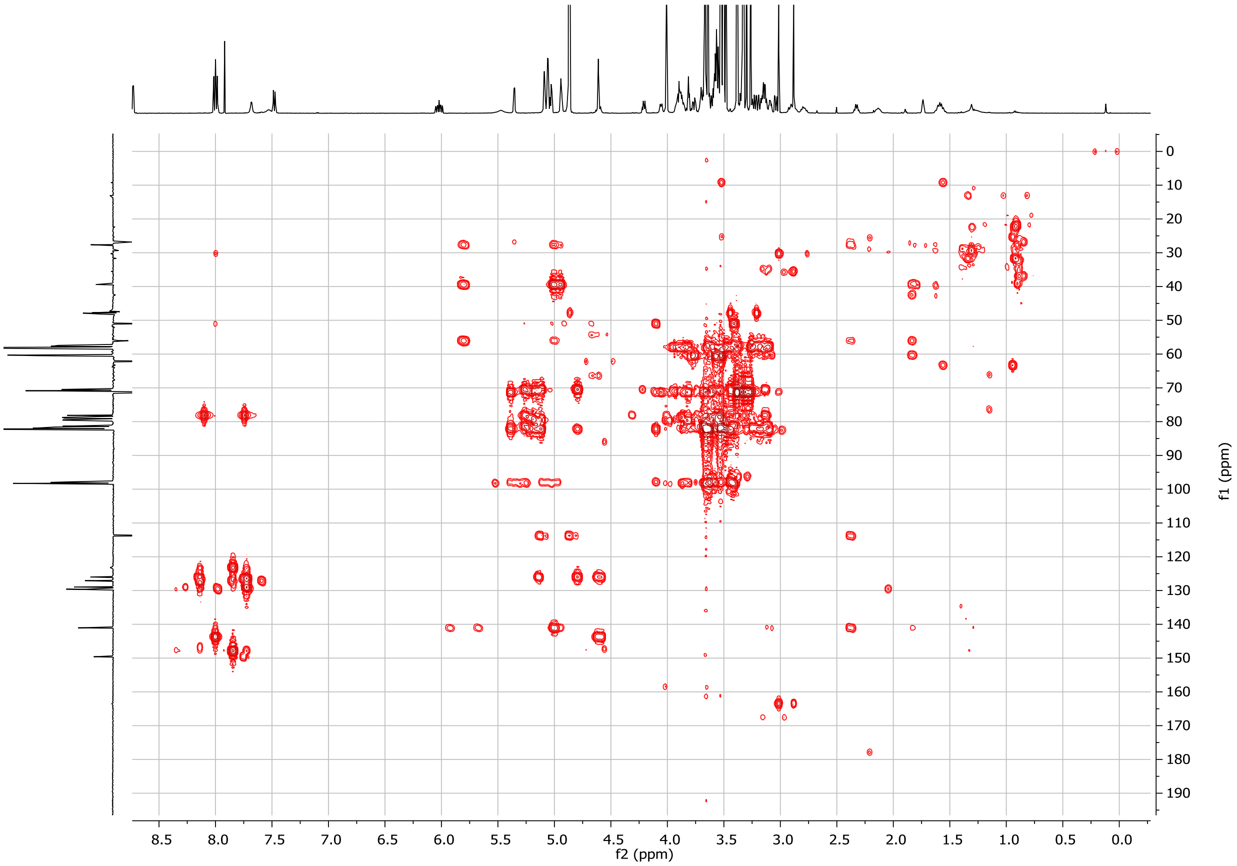

Figure S20: HMBC spectrum of compound **9a**.

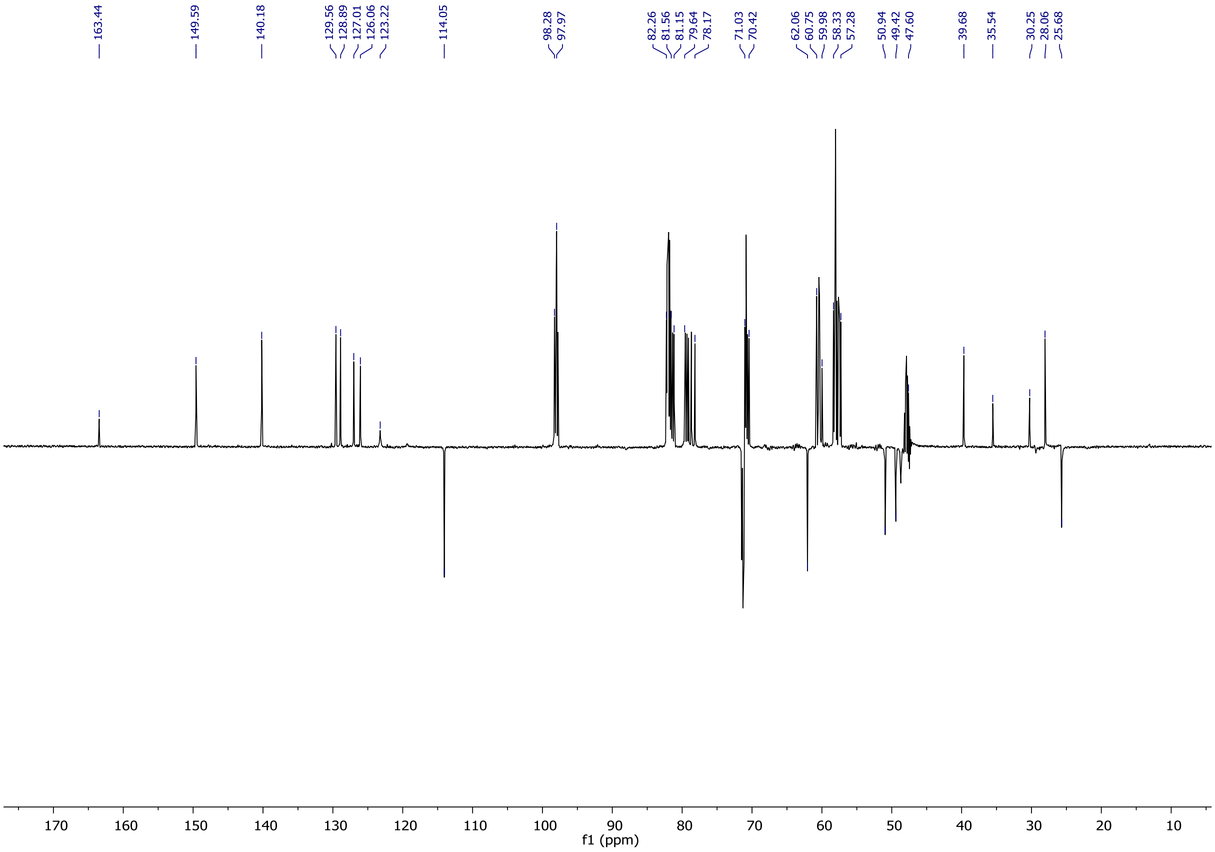

Figure S21: ^{13}C DEPT spectrum of compound **9b**.

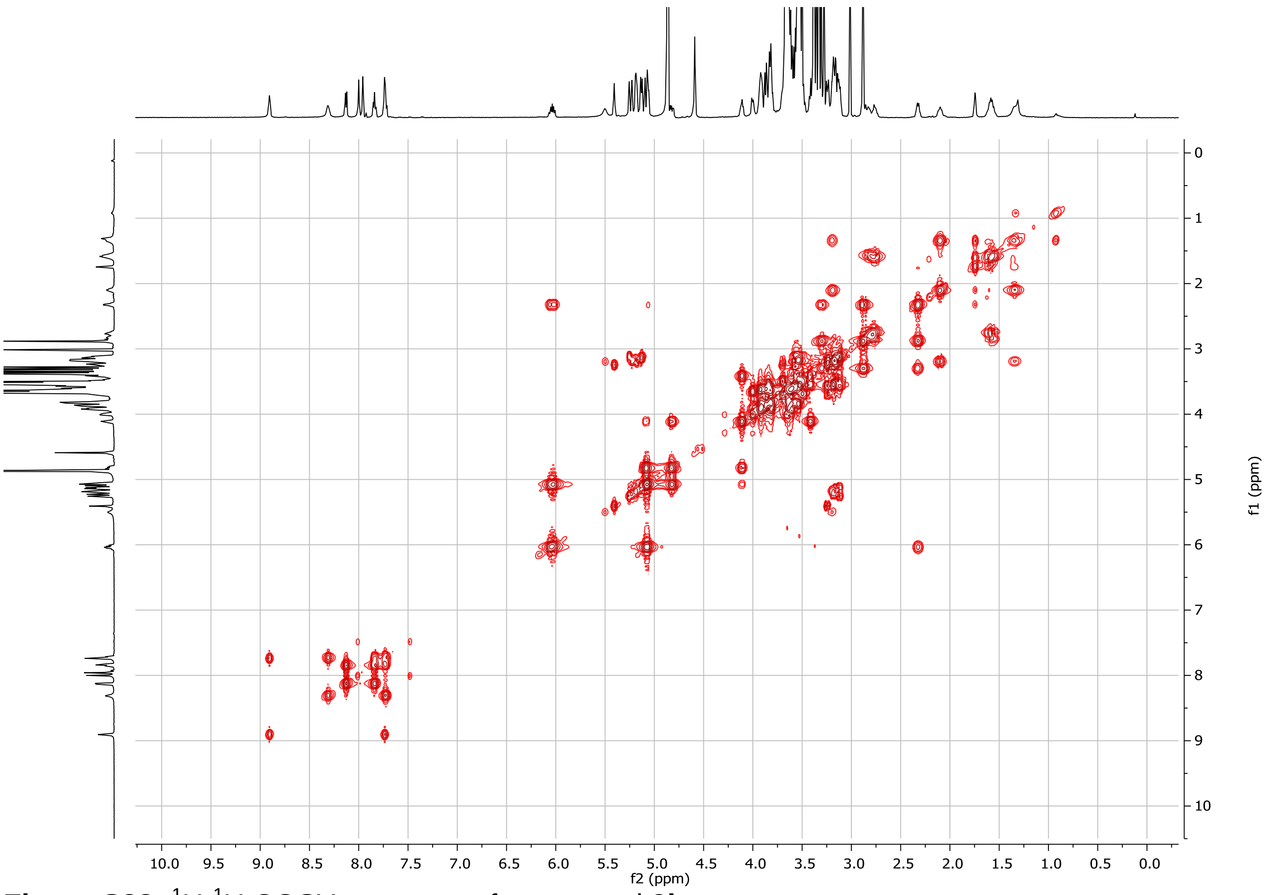

Figure S22: ^{1}H-^{1}H COSY spectrum of compound **9b**.

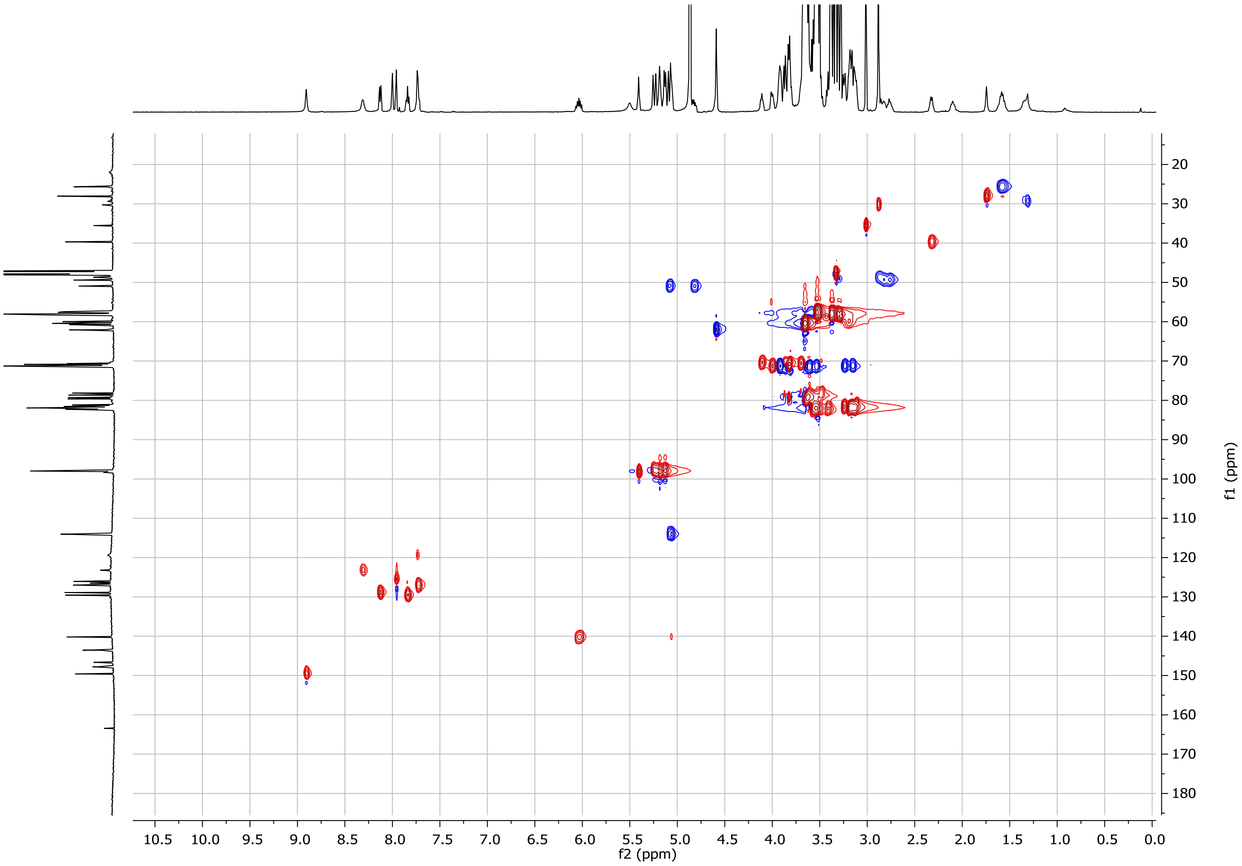

Figure S23: HSQC spectrum of compound **9b**.

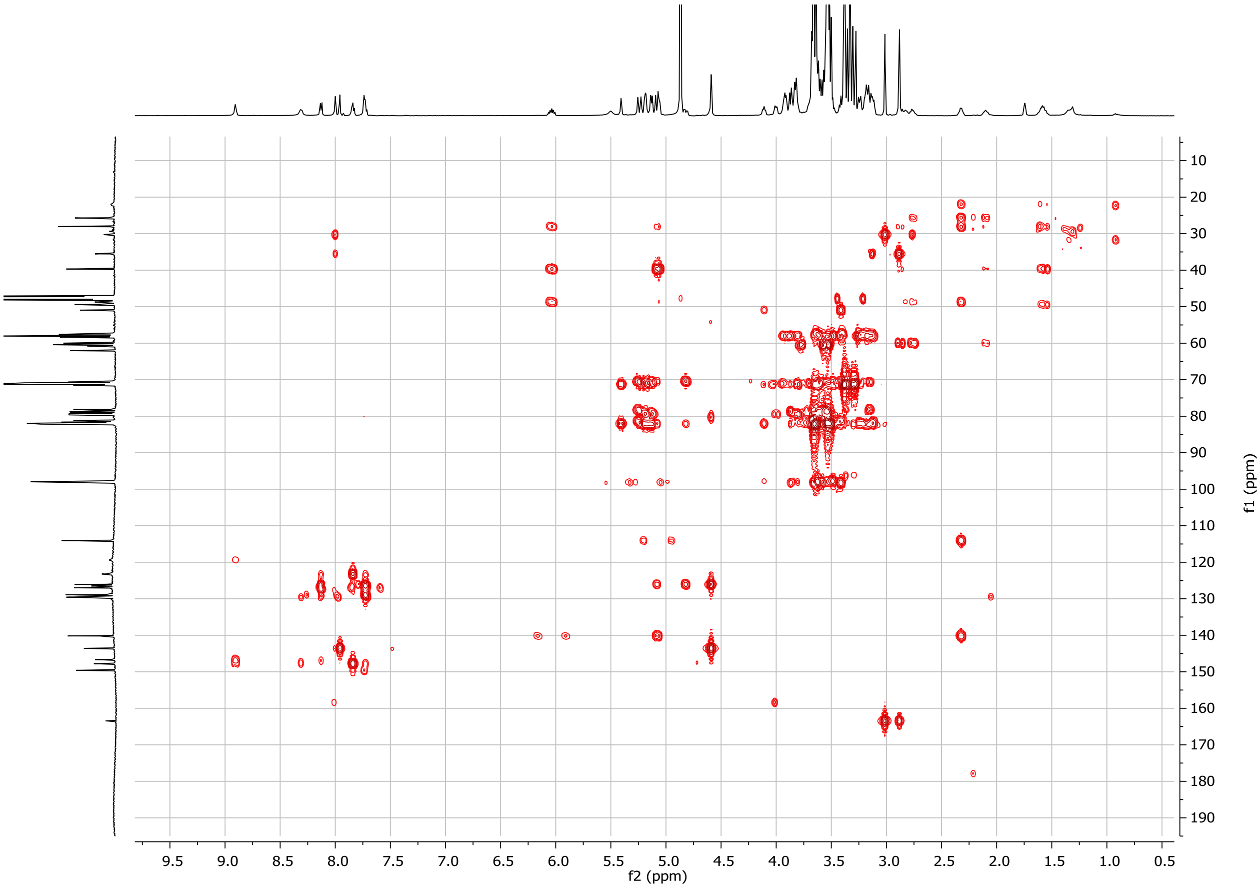

Figure S24: HMBC spectrum of compound **9b**.

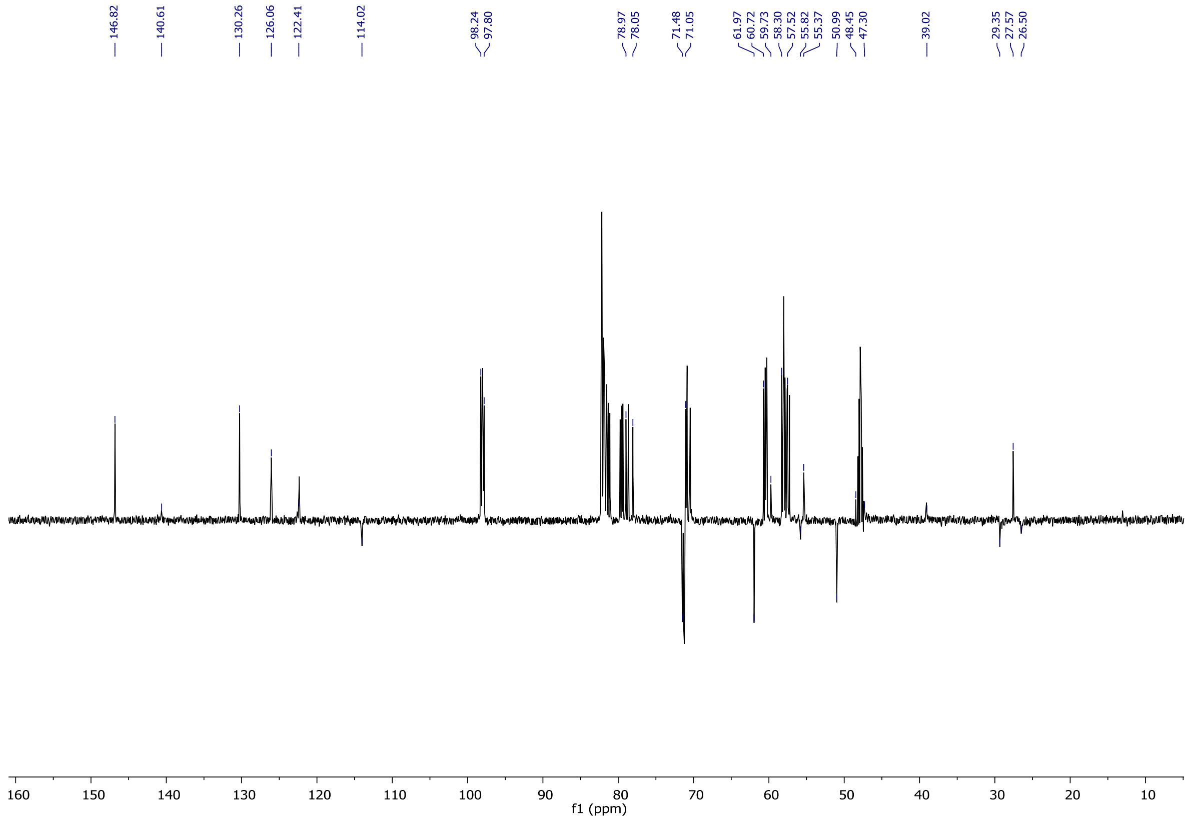

Figure S25: ^{13}C DEPT spectrum of compound **9c**.

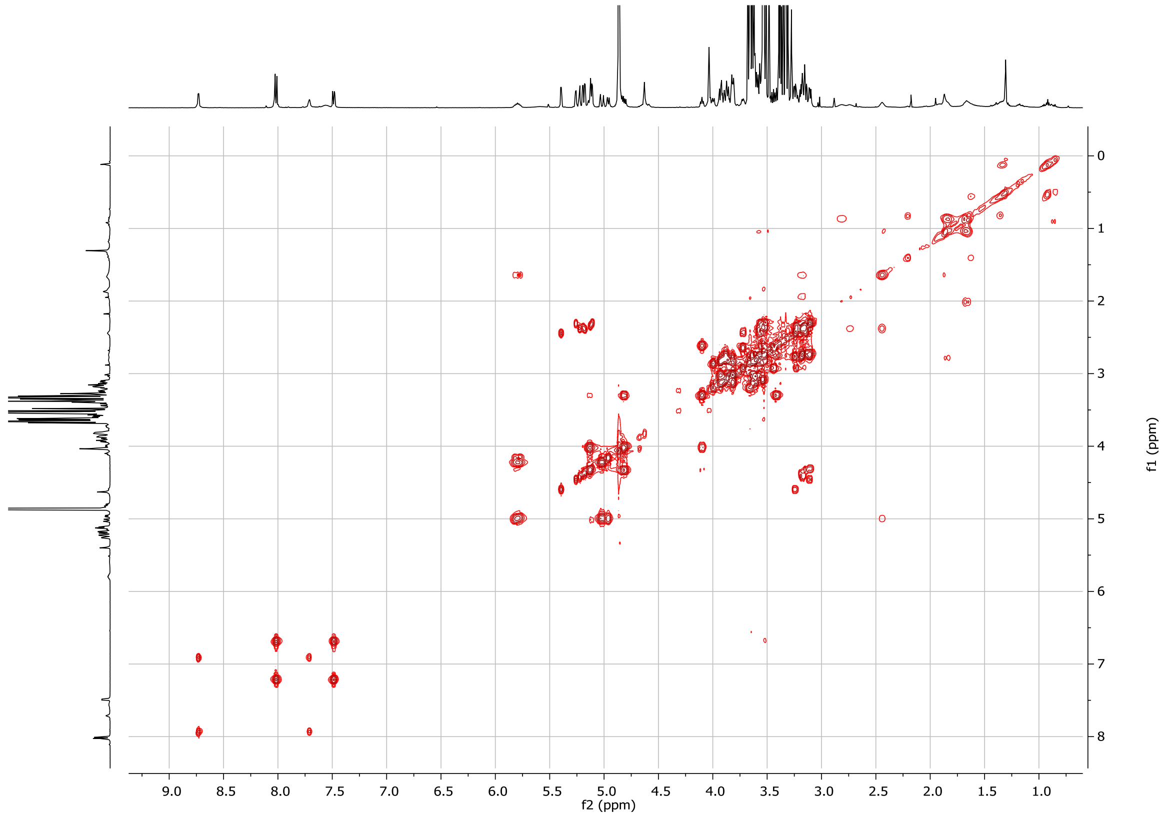

Figure S26: ^{1}H-^{1}H COSY spectrum of compound **9c**.

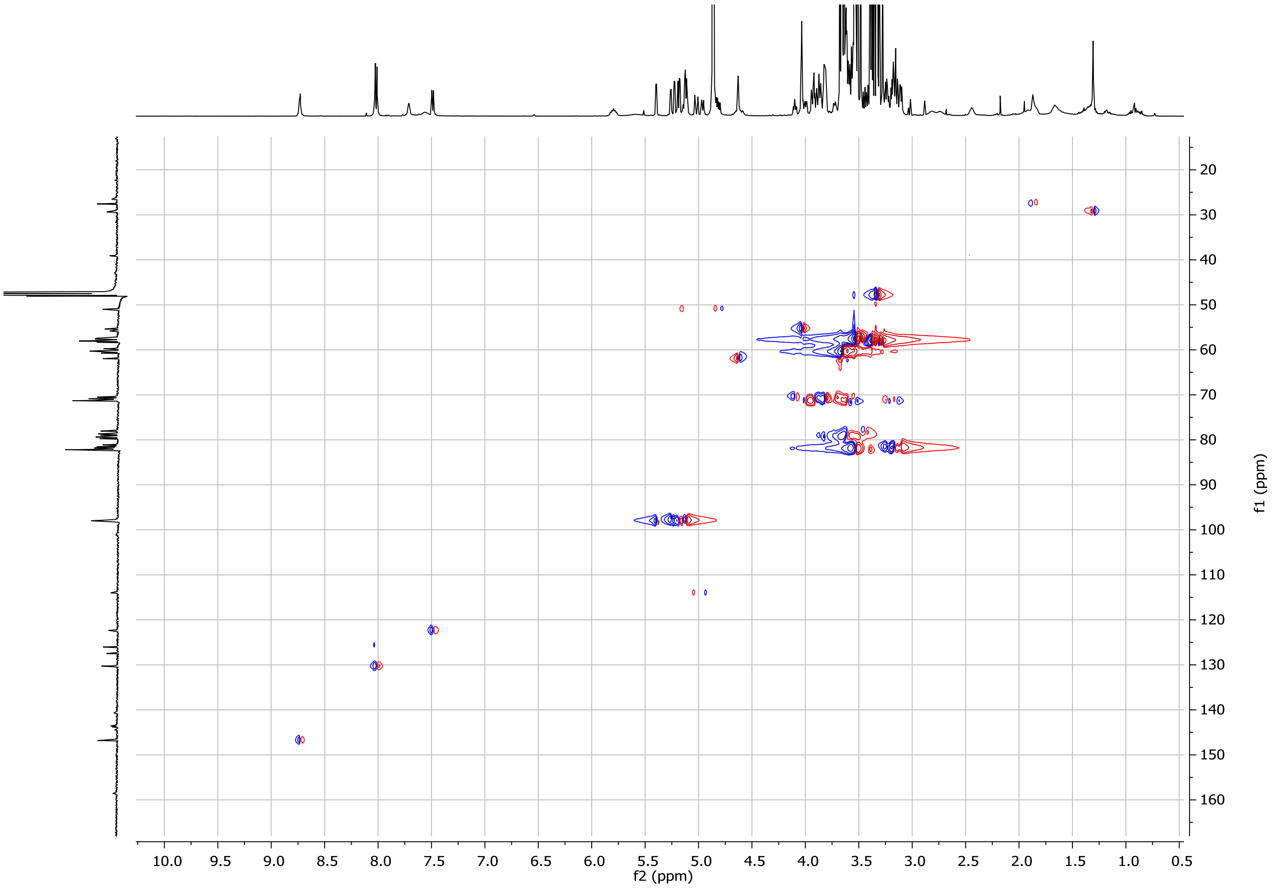

Figure S27: HSQC spectrum of compound **9c**.

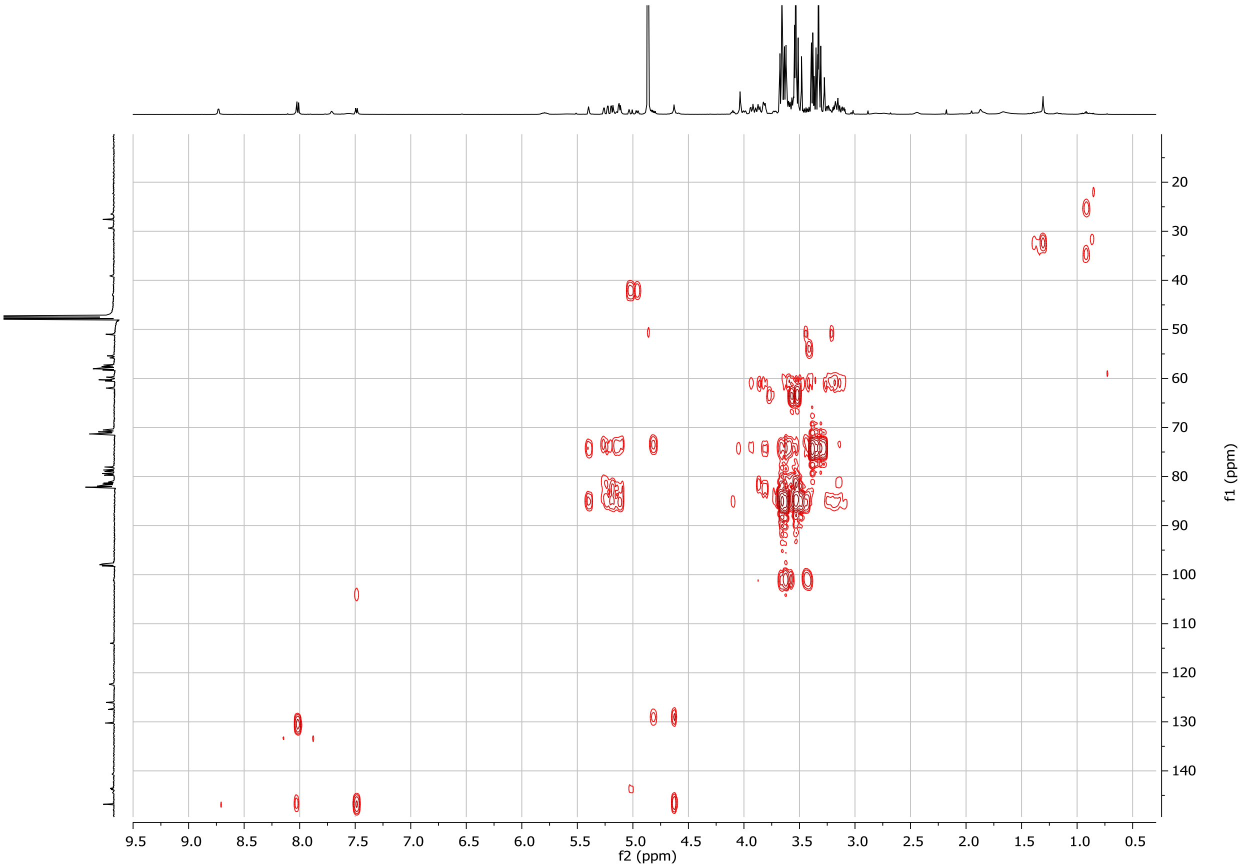

Figure S28: HMBC spectrum of compound **9c**.

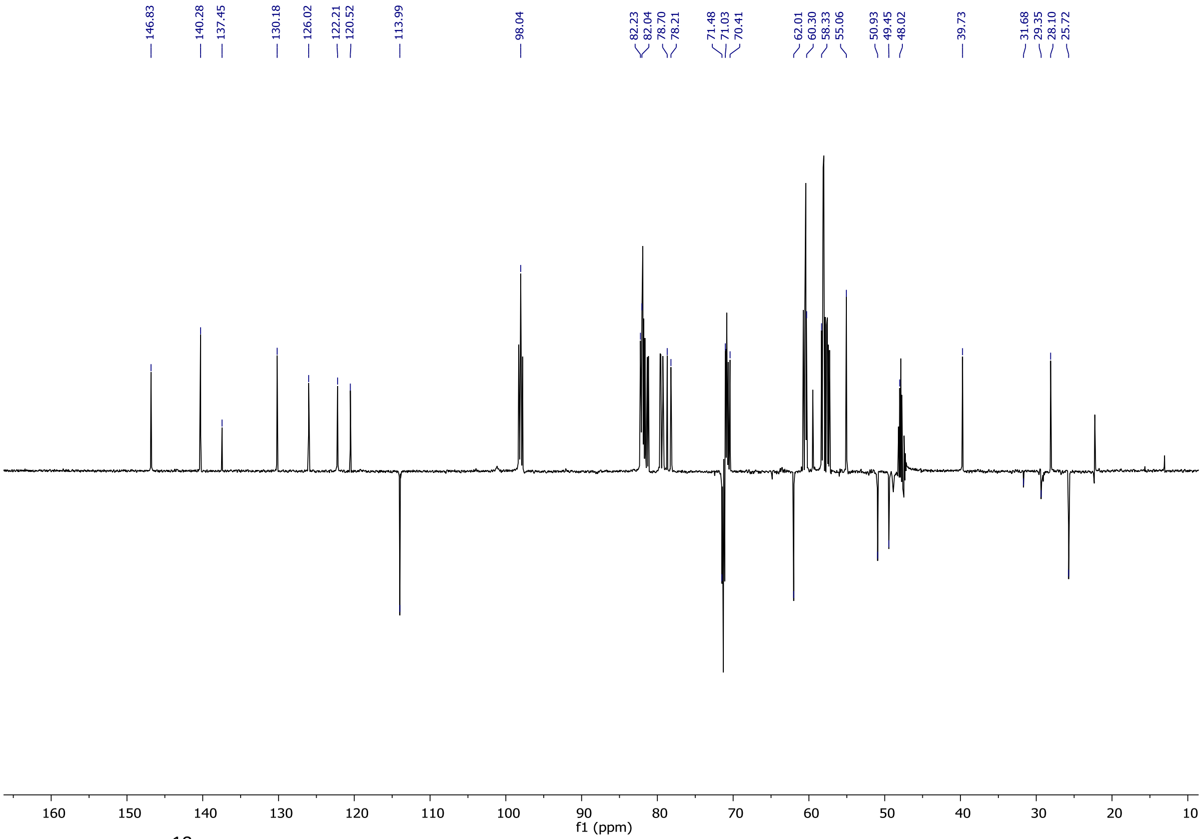

Figure S29: ^{13}C DEPT spectrum of compound **9d**.

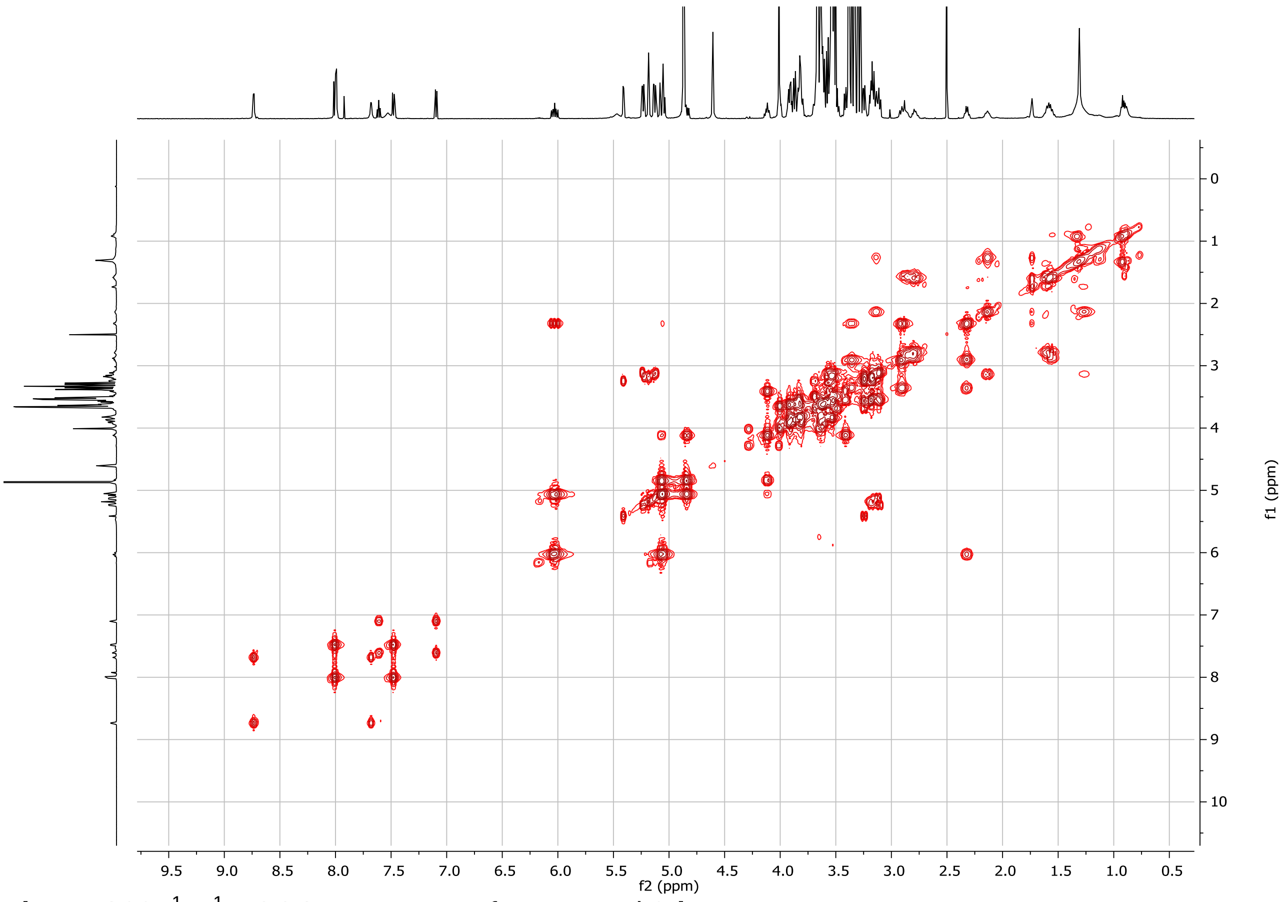

Figure S30: ^{1}H-^{1}H COSY spectrum of compound **9d**.

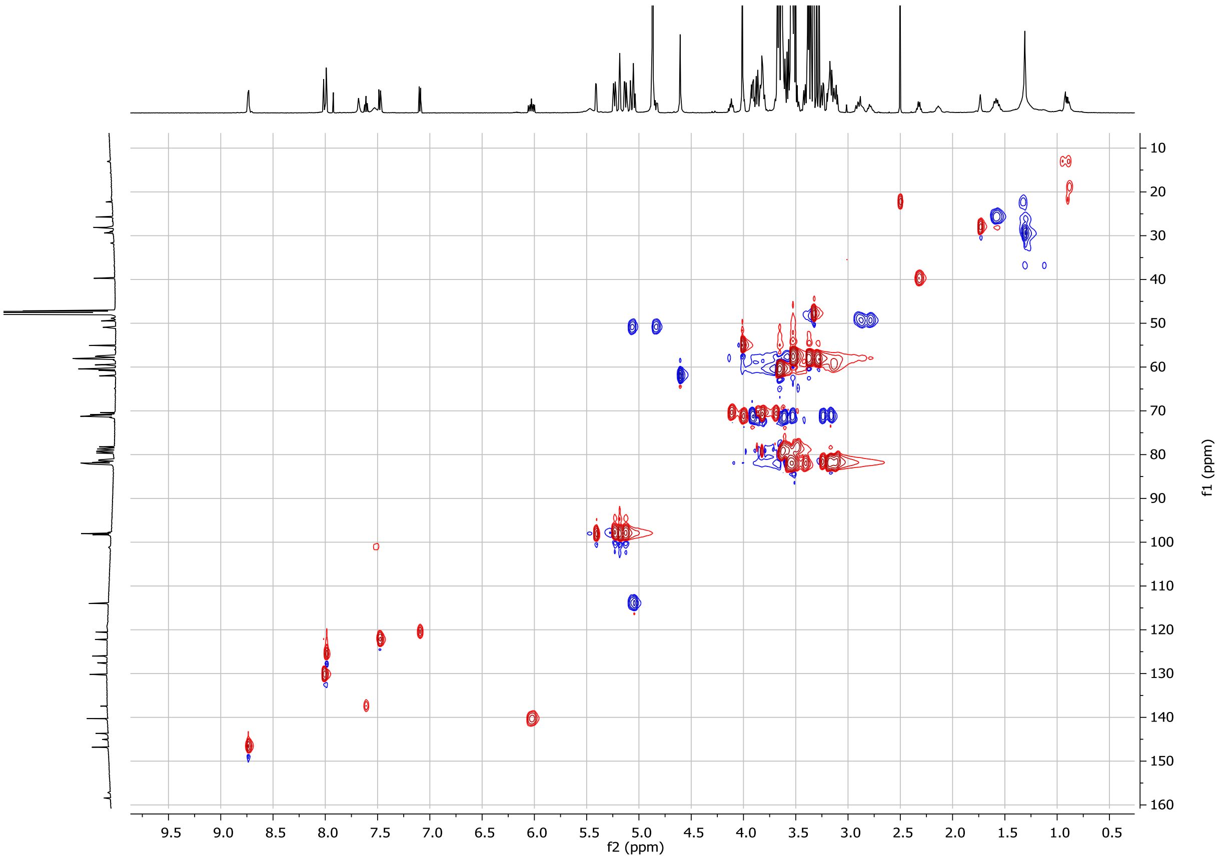

Figure S31: HSQC spectrum of compound **9d**.

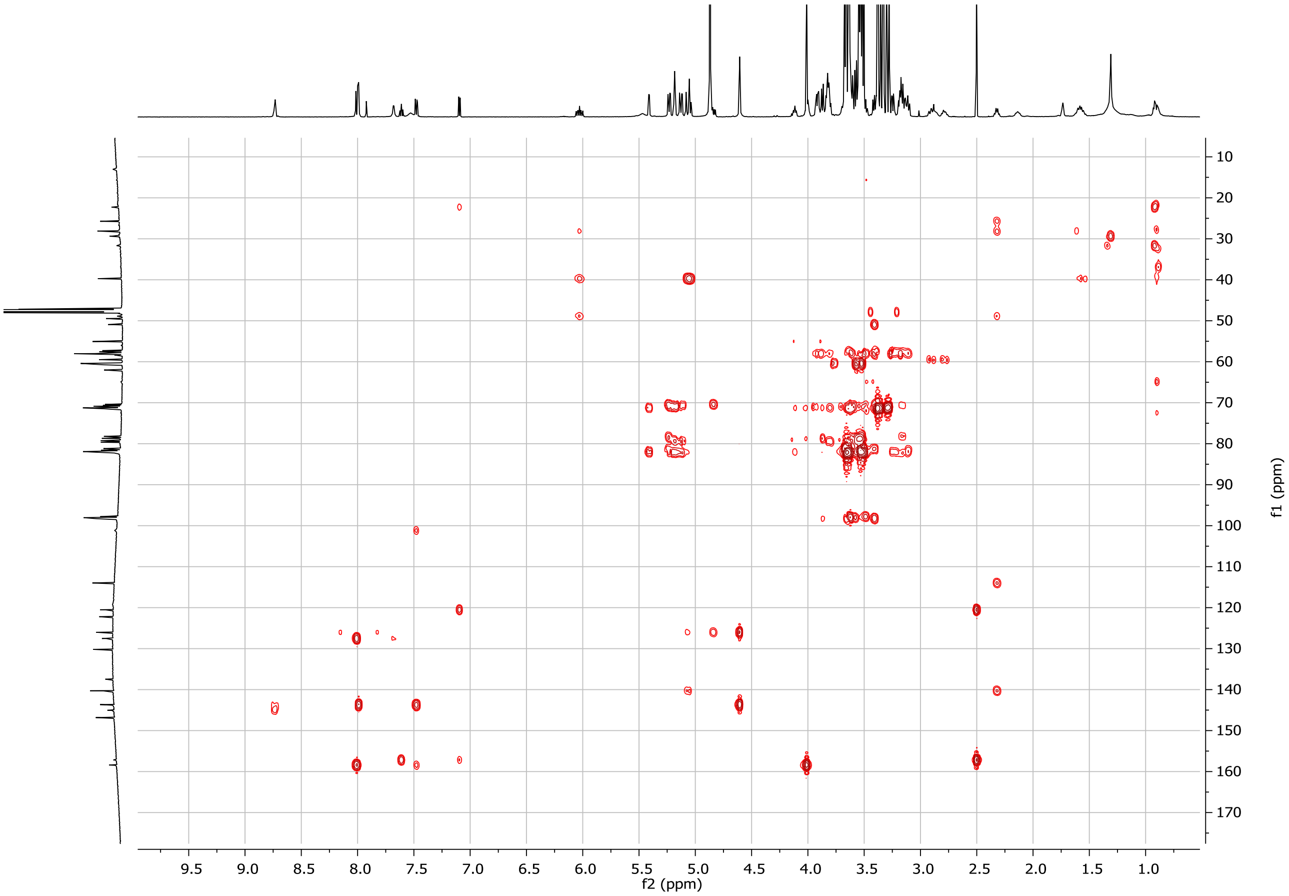

Figure S32: HMBC spectrum of compound **9d**.

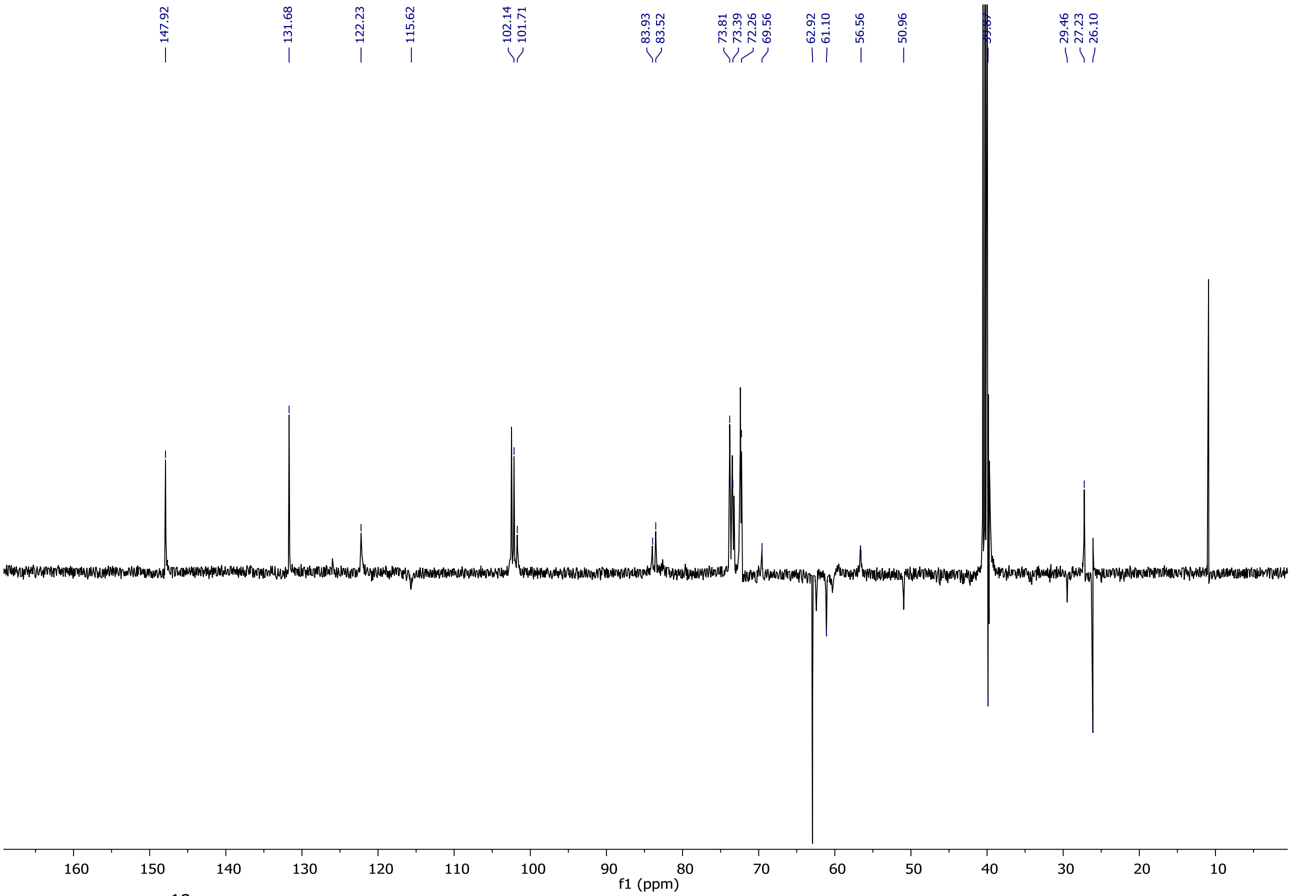

Figure S33: ^{13}C DEPT spectrum of compound **11**.

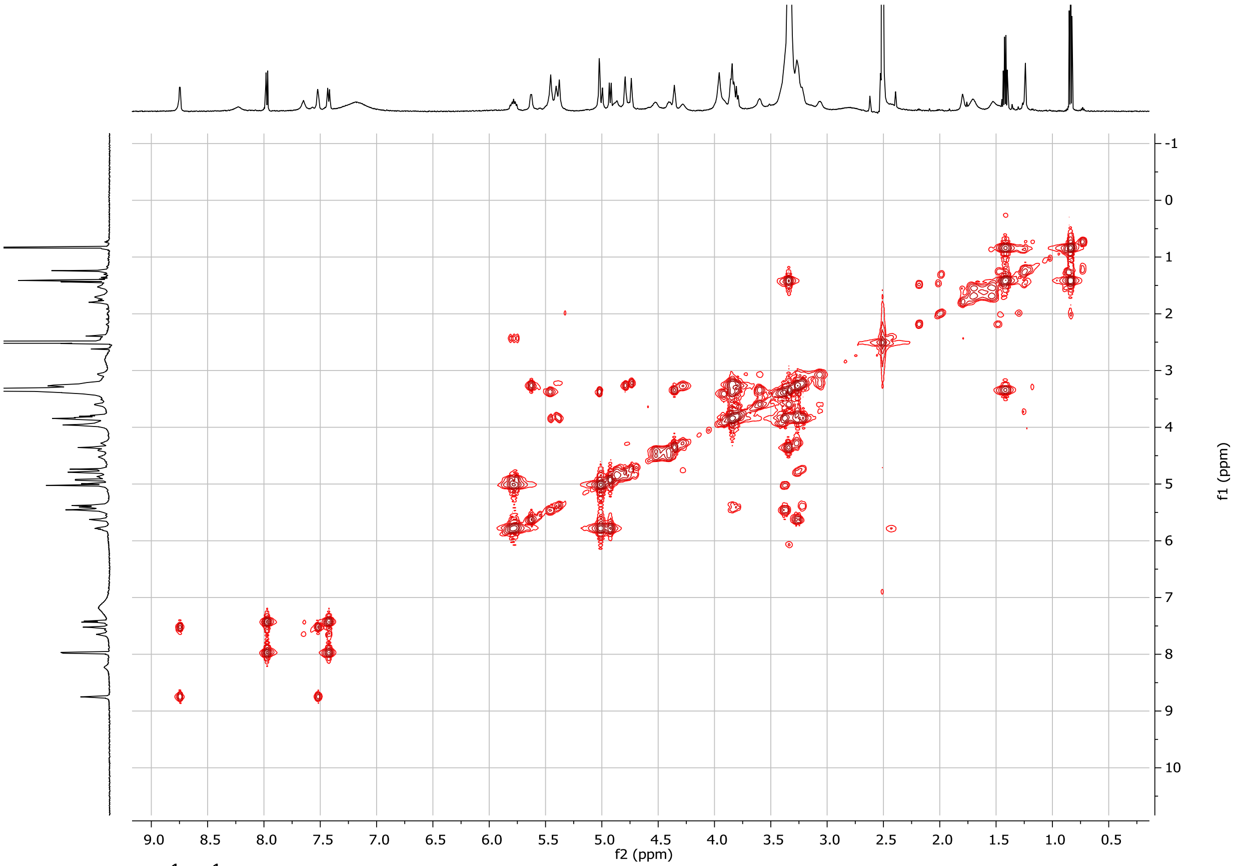

Figure S34: ^{1}H-^{1}H COSY spectrum of compound **11**.

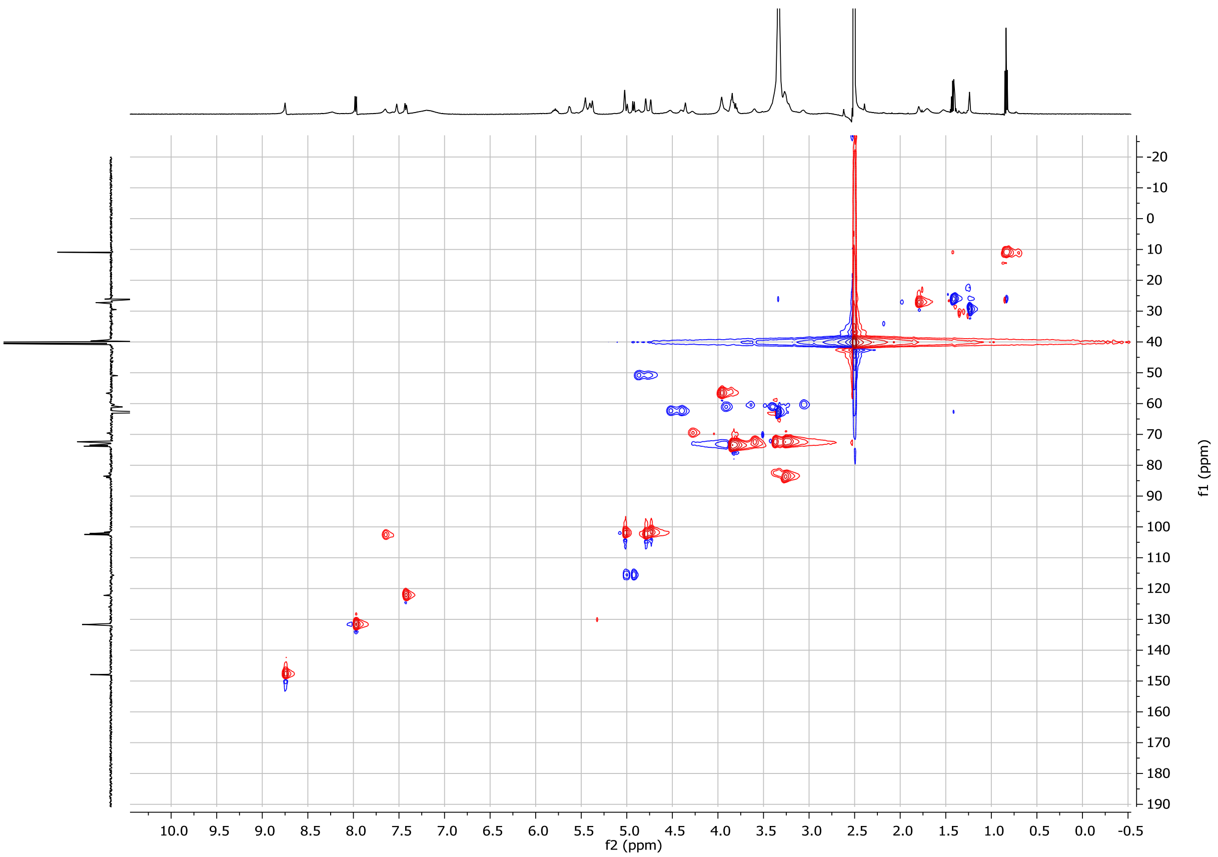

Figure S35: DEPT-edited HSQC spectrum of compound **11**.

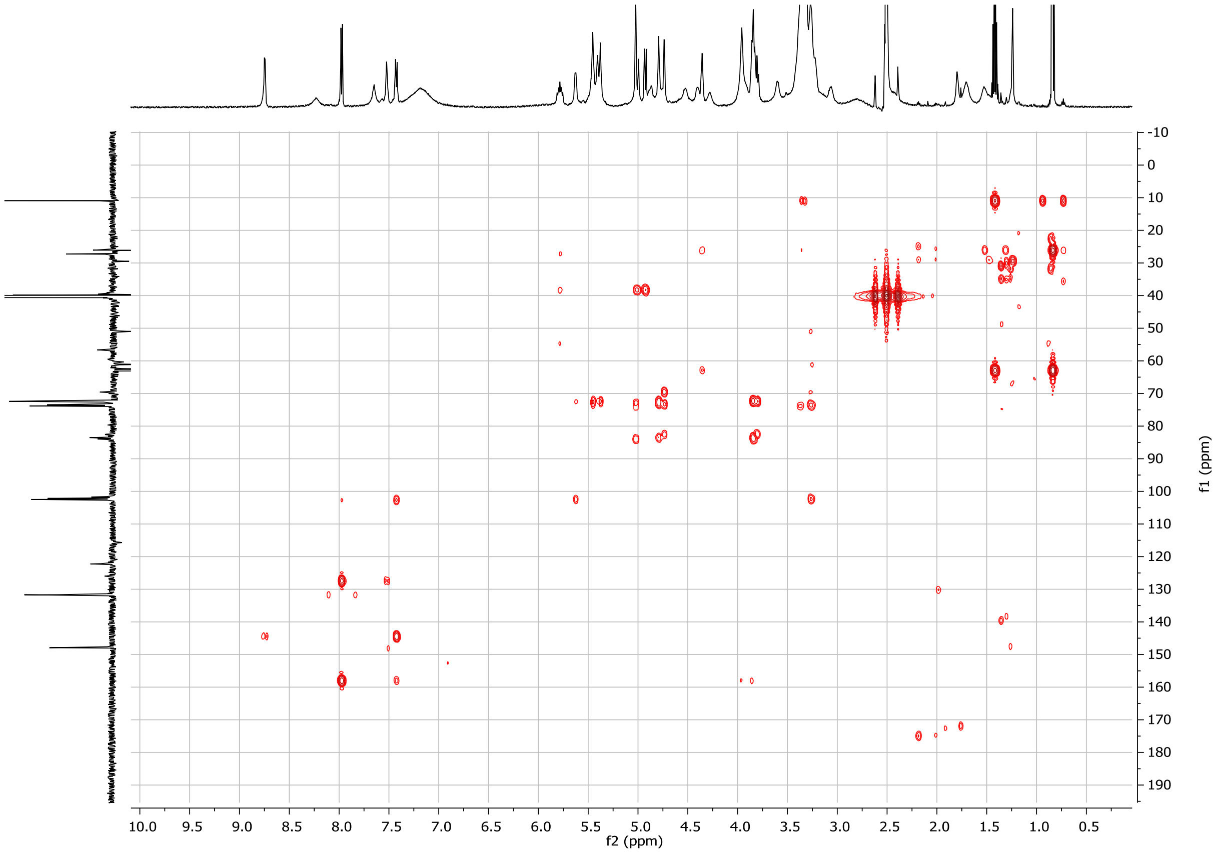

Figure S36: HMBC spectrum of compound **11**.
